# Estimates of the global, regional, and national morbidity, mortality, and aetiologies of lower respiratory infections in 195 countries, 1990–2016: a systematic analysis for the Global Burden of Disease Study 2016

**DOI:** 10.1016/S1473-3099(18)30310-4

**Published:** 2018-11

**Authors:** GBD 2016 Lower Respiratory Infections Collaborators, Christopher Troeger, Brigette Blacker, Ibrahim A Khalil, Puja C Rao, Jackie Cao, Stephanie R M Zimsen, Samuel B Albertson, Aniruddha Deshpande, Tamer Farag, Zegeye Abebe, Ifedayo Morayo O Adetifa, Tara Ballav Adhikari, Mohammed Akibu, Faris Hasan Al Lami, Ayman Al-Eyadhy, Nelson Alvis-Guzman, Azmeraw T Amare, Yaw Ampem Amoako, Carl Abelardo T Antonio, Olatunde Aremu, Ephrem Tsegay Asfaw, Solomon Weldegebreal Asgedom, Tesfay Mehari Atey, Engi Farouk Attia, Euripide Frinel G Arthur Avokpaho, Henok Tadesse Ayele, Tambe Betrand Ayuk, Kalpana Balakrishnan, Aleksandra Barac, Quique Bassat, Masoud Behzadifar, Meysam Behzadifar, Soumyadeep Bhaumik, Zulfiqar A Bhutta, Ali Bijani, Michael Brauer, Alexandria Brown, Paulo A M Camargos, Carlos A Castañeda-Orjuela, Danny Colombara, Sara Conti, Abel Fekadu Dadi, Lalit Dandona, Rakhi Dandona, Huyen Phuc Do, Eleonora Dubljanin, Dumessa Edessa, Hajer Elkout, Aman Yesuf Endries, Daniel Obadare Fijabi, Kyle J Foreman, Mohammad H Forouzanfar, Nancy Fullman, Alberto L Garcia-Basteiro, Bradford D Gessner, Peter W Gething, Rahul Gupta, Tarun Gupta, Gessessew Bugssa Hailu, Hamid Yimam Hassen, Mohammad T Hedayati, Mohsen Heidari, Desalegn Tsegaw Hibstu, Nobuyuki Horita, Olayinka S Ilesanmi, Mihajlo B Jakovljevic, Amr A Jamal, Amaha Kahsay, Amir Kasaeian, Dessalegn Haile Kassa, Yousef Saleh Khader, Ejaz Ahmad Khan, Md Nuruzzaman Khan, Young-Ho Khang, Yun Jin Kim, Niranjan Kissoon, Luke D Knibbs, Sonali Kochhar, Parvaiz A Koul, G Anil Kumar, Rakesh Lodha, Hassan Magdy Abd El Razek, Deborah Carvalho Malta, Joseph L Mathew, Desalegn Tadese Mengistu, Haftay Berhane Mezgebe, Karzan Abdulmuhsin Mohammad, Mohammed A Mohammed, Fatemeh Momeniha, Srinivas Murthy, Cuong Tat Nguyen, Katie R Nielsen, Dina Nur Anggraini Ningrum, Yirga Legesse Nirayo, Eyal Oren, Justin R Ortiz, Mahesh PA, Maarten J Postma, Mostafa Qorbani, Reginald Quansah, Rajesh Kumar Rai, Saleem M Rana, Chhabi Lal Ranabhat, Sarah E Ray, Mohammad Sadegh Rezai, George Mugambage Ruhago, Saeid Safiri, Joshua A Salomon, Benn Sartorius, Miloje Savic, Monika Sawhney, Jun She, Aziz Sheikh, Mekonnen Sisay Shiferaw, Mika Shigematsu, Jasvinder A Singh, Ranjani Somayaji, Jeffrey D Stanaway, Muawiyyah Babale Sufiyan, Getachew Redae Taffere, Mohamad-Hani Temsah, Matthew J Thompson, Ruoyan Tobe-Gai, Roman Topor-Madry, Bach Xuan Tran, Tung Thanh Tran, Kald Beshir Tuem, Kingsley Nnanna Ukwaja, Stein Emil Vollset, Judd L Walson, Fitsum Weldegebreal, Andrea Werdecker, T Eoin West, Naohiro Yonemoto, Maysaa El Sayed Zaki, Lei Zhou, Sanjay Zodpey, Theo Vos, Mohsen Naghavi, Stephen S Lim, Ali H Mokdad, Christopher J L Murray, Simon I Hay, Robert C Reiner

## Abstract

**Background:**

Lower respiratory infections are a leading cause of morbidity and mortality around the world. The Global Burden of Diseases, Injuries, and Risk Factors (GBD) Study 2016, provides an up-to-date analysis of the burden of lower respiratory infections in 195 countries. This study assesses cases, deaths, and aetiologies spanning the past 26 years and shows how the burden of lower respiratory infection has changed in people of all ages.

**Methods:**

We used three separate modelling strategies for lower respiratory infections in GBD 2016: a Bayesian hierarchical ensemble modelling platform (Cause of Death Ensemble model), which uses vital registration, verbal autopsy data, and surveillance system data to predict mortality due to lower respiratory infections; a compartmental meta-regression tool (DisMod-MR), which uses scientific literature, population representative surveys, and health-care data to predict incidence, prevalence, and mortality; and modelling of counterfactual estimates of the population attributable fraction of lower respiratory infection episodes due to *Streptococcus pneumoniae, Haemophilus influenzae* type b, influenza, and respiratory syncytial virus. We calculated each modelled estimate for each age, sex, year, and location. We modelled the exposure level in a population for a given risk factor using DisMod-MR and a spatio-temporal Gaussian process regression, and assessed the effectiveness of targeted interventions for each risk factor in children younger than 5 years. We also did a decomposition analysis of the change in LRI deaths from 2000–16 using the risk factors associated with LRI in GBD 2016.

**Findings:**

In 2016, lower respiratory infections caused 652 572 deaths (95% uncertainty interval [UI] 586 475–720 612) in children younger than 5 years (under-5s), 1 080 958 deaths (943 749–1 170 638) in adults older than 70 years, and 2 377 697 deaths (2 145 584–2 512 809) in people of all ages, worldwide. *Streptococcus pneumoniae* was the leading cause of lower respiratory infection morbidity and mortality globally, contributing to more deaths than all other aetiologies combined in 2016 (1 189 937 deaths, 95% UI 690 445–1 770 660). Childhood wasting remains the leading risk factor for lower respiratory infection mortality among children younger than 5 years, responsible for 61·4% of lower respiratory infection deaths in 2016 (95% UI 45·7–69·6). Interventions to improve wasting, household air pollution, ambient particulate matter pollution, and expanded antibiotic use could avert one under-5 death due to lower respiratory infection for every 4000 children treated in the countries with the highest lower respiratory infection burden.

**Interpretation:**

Our findings show substantial progress in the reduction of lower respiratory infection burden, but this progress has not been equal across locations, has been driven by decreases in several primary risk factors, and might require more effort among elderly adults. By highlighting regions and populations with the highest burden, and the risk factors that could have the greatest effect, funders, policy makers, and programme implementers can more effectively reduce lower respiratory infections among the world's most susceptible populations.

**Funding:**

Bill & Melinda Gates Foundation.

## Introduction

Lower respiratory tract infections, defined in the Global Burden of Diseases, Injuries, and Risk Factors Study (GBD) as pneumonia or bronchiolitis, are a leading cause of mortality and morbidity worldwide. Nearly 2·38 million deaths resulted from lower respiratory infections in 2016, making lower respiratory infections the sixth leading cause of mortality for all ages and the leading cause of death among children younger than 5 years.^1^ Historically, lower respiratory infection burden varies substantially across the globe, disproportionately affecting the young and the impoverished.^2^ People at highest risk for contracting or dying from lower respiratory infections often come from households with inconsistent or insufficient access to adequate nutrition, clean cooking fuel, vaccines, and sanitation, or have immunocompromising conditions.^3^ Although there has been a substantial decrease in lower respiratory infection mortality since 1990 (23·0% decrease, 95% uncertainty interval [UI] 15·7–29·4),^1^ the majority of the remaining deaths could be avertable and require increased global investment in prevention and treatment interventions.

**Research in context**

**Evidence before this study**

Lower respiratory infections are responsible for a substantial number of deaths, particularly among children younger than 5 years (under-5). Most other studies that have attempted to quantify the burden of lower respiratory infections have focused on children younger than 5 years or on subpopulations, such as immunocompromised children and adults. Other studies have attempted to quantify respiratory pathogens, such as *Streptococcus pneumoniae* or influenza, and have found a wide range of results depending on modelling assumptions and input data. The Global Burden of Diseases, Injuries, and Risk Factors Study (GBD) 2015, showed lower respiratory infections being responsible for over 2·74 million deaths (95% uncertainty interval [UI] 2·50–2·86) in 2015, and the third leading cause of under-5 mortality worldwide. Furthermore, an estimated 291·8 million episodes (276·3–307·0) of lower respiratory infections occurred in 2015, and were the second leading cause of disability-adjusted life-years (103 million DALYs; 95% UI 96·1–109·1).

**Added value of this study**

This GBD analysis includes new data sources, new covariates, and various advances in the modelling methods. Furthermore, we produce estimates of hospitalisations due to lower respiratory infection (for all ages), highlight the underappreciated and growing burden of lower respiratory infections on elderly populations, and do a novel analysis of the effect of changes in risk factors over time on the lower respiratory infection mortality rate, allowing for targeted strategies unique to each location to reduce lower respiratory infection risk and mortality.

**Implications of all the available evidence**

The epidemiology of lower respiratory infections is changing, and large reductions in lower respiratory infection mortality, particularly among young children have occurred in the past 16 years. Still, air pollution, childhood undernutrition, and access to timely and appropriate health care represent challenges and opportunities for continuing to avert mortality due to this preventable cause of death.

A handful of global initiatives have been developed to combat lower respiratory infections and other common illnesses, including the Global Action Plan for Pneumonia and Diarrhoea (GAPPD), Every Breath Counts, and the Stop Pneumonia Initiative. The GAPPD, created by WHO and UNICEF in 2013, is a global initiative focusing on the prevention and treatment of lower respiratory infections.^4^ Among other initiatives, the GAPPD promotes interventions to address the most evident risks for contracting and dying from lower respiratory infections. It aims to reduce the burden of lower respiratory infection in every country by 2025, by lowering mortality to three deaths per 1000 people and lower respiratory infection incidence to 75% of the country-specific levels in 2010. Such goals were developed with an emphasis on childhood mortality and the substantial burden of lower respiratory infections among adults might not be fully appreciated. Understanding and quantifying trends in lower respiratory infection burden among people of all ages is crucial to develop plans that accelerate improvement and to provide timely and appropriate investment in interventions to reduce lower respiratory infection burden and to track progress toward global goals.

Estimates of the burden of lower respiratory infections and their aetiologies are produced annually as part of the GBD Study. This paper presents the results of GBD 2016 for lower respiratory infections and four high-burden aetiologies (*Haemophilus influenza* type b [Hib], *Streptococcus pneumoniae*, influenza, and respiratory syncytial virus [RSV]), including deaths, episodes, disability-adjusted life-years (DALYs), and risk factors, the relationship between lower respiratory infections and social development, and intervention strategies for 195 countries, from 1990 to 2016, by age, for men and women. We build on previous descriptions of the global burden of lower respiratory infection by incorporating more than 287 000 additional cause of death and non-fatal datapoints. Further, this study provides greater emphasis on exploring the trends in morbidity and mortality, such as the relationships between cases and fatality, by highlighting the burden across all ages, and by identifying risks and interventions that have the greatest potential to reduce lower respiratory infection disease burden.

## Methods

### Overview

Detailed methods for the GBD study and on lower respiratory infection estimation in GBD have been previously published.^1^, ^5^ We describe these methods briefly, focusing on changes from previous GBD methods. There were no substantial modelling changes between GBD 2015 and GBD 2016. lower respiratory infections are defined as diseases of the lower airways, including pneumonia and bronchiolitis. Uncertainty in the lower respiratory infection estimates are maintained through the modelling process using draws, and are reflected as the 2·5 and 97·5 percentiles of the distribution. In compliance with the Guidelines for Accurate and Transparent Health Estimates Reporting,^6^ data and code for the GBD 2016 cycle will be made publicly available. The appendix shows more information about the data included in this study (pp 3–5, 9–13).

### Modelling

We modelled lower respiratory infection mortality using the Cause of Death Ensemble model (CODEm) platform. CODEm is a Bayesian, hierarchical, space-time, ensemble model designed to predict cause-specific mortality by age, sex, location, and year.^1^, ^7^ CODEm produces a wide variety of submodels designed to explore a diverse set of covariates (eg, childhood undernutrition, air pollution, and Socio-demographic Index) and model types. We added several new covariates for GBD 2016, including prevalence of childhood wasting (low weight-for-height) and underweight (low weight-for-age), vitamin A deficiency, zinc deficiency, health-care access and quality, and safe handwashing. A full list of covariates used in the CODEm modelling can be found in the appendix (pp 7–8). Each submodel is weighted on the basis of out-of-sample predictive validity, and contributes to a final set of 1000 draws that is composed of draws sampled from the submodels based on each model's statistical performance. These predictive regression models produce estimates of lower respiratory infection mortality for age, sex, location, and year on the basis of vital registration, verbal autopsy data, and surveillance system data.

The GBD 2016 used more than 30 000 datapoints from more than 700 sources. Compared with the GBD 2015, the GBD 2016 cycle expanded its data sources by adding 169 country-years of vital registration and 24 new verbal autopsy studies, including Sample Registration System data from the Government of India for each state, stratified by urban or rural residence.^1^ Detailed information about the International Classification of Disease (ICD) codes that were used to identify lower respiratory infections is available in the appendix (p 3). To ensure consistency and interpretability across GBD cause-specific estimations, we adjusted the sum of the all-cause-specific mortality estimates to equal the all-cause mortality estimate. We did this using CoDCorrect, in which the modelled values for lower respiratory infection and all other causes of mortality were scaled on the basis of the uncertainty of those values. A new process in GBD 2016 reassigned some lower respiratory infection deaths for which the underlying cause of death was because of disorders in elderly adults, such as Alzheimer's disease and Parkinson's disease, reducing the number of deaths among people aged 65 years and older.^1^ This reassignment was based on the prevalence and mortality of those diseases for each location, year, sex, and people older than 65 years, and was designed to correct for systematic under-reporting of dementias as the underlying cause of death in the ICD death registry data.

We modelled lower respiratory infection incidence in DisMod-MR version 2.1 (DisMod). DisMod is a Bayesian, hierarchical meta-regression tool.^8^ Similar to CODEm, DisMod uses space-time information and country-level covariates to produce modelled estimates for each age, year, location, and sex. DisMod also contains a compartmental model that enforces a consistent re-lationship between incidence, prevalence, and mortality in a series of ordinary differential equations. Input data for these models include scientific literature, population representative surveys, and hospital and health-care utilisation records. We also modelled hospital admissions due to lower respiratory infection in DisMod using inpatient records exclusively. We expanded the database for lower respiratory infection modelling in the 2016 cycle to include 133 of these new sources and 9844 new datapoints by updating our systematic review from Jan 1, 2016, to May 31, 2017. Because lower respiratory infection is seasonal in many places, we introduced a method to adjust for data sources that were shorter than a year by fitting a sine and cosine model with a period of 6 months for each GBD region and adjusting the lower respiratory infection prevalence on the basis of the predicted deviation from the mean. More information on this process can be found in the appendix (pp 14–18).

We attributed lower respiratory infection episodes and deaths to four aetiologies: *S pneumoniae* (pneumococcal pneumonia), Hib, influenza, and RSV, which were identified by expert review for GBD 2010 on the basis of the aetiological burden and the available data. Aetiologies were estimated population attributable fractions (PAFs), which represent the proportional reduction in lower respiratory infection morbidity or mortality that would be observed if the exposure to the pathogen was zero. Aetiological attribution, based on counterfactual estimates, accounted for co-infection between pathogens and for the distribution of pathogens in healthy individuals. Our conterfactual approach to aetiological distribution allowed for co-attribution between aetiologies, and so PAFs do not sum to 100%. We did not attribute aetiologies to neonatal lower respiratory infection cases or deaths because of an absence of reliable data in this age group, and we did not consider Hib in age groups older than 5 years old because there were no data on vaccine effectiveness for the Hib vaccine in children older than 5 years.

### Aetiological attribution

We used a vaccine probe design to estimate the PAF for pneumococcal pneumonia and Hib by first calculating the ratio of vaccine effectiveness against non-specific pneumonia to pathogen-specific pneumonia at the study level.^9^, ^10^, ^11^ We then adjusted this estimate by vaccine coverage, and for pneumococcal pneumonia we adjusted for the proportion of pneumococcal serotypes covered by the pneumococcal conjugate vaccine (ten-valent and 13-valent), and vaccine effectiveness to estimate country-specific and year-specific PAF values.^12^, ^13^ A detailed explanation of these calculations is provided in the appendix (pp 23, 24). We did not account for herd immunity in our estimates. We used separate pneumococcal pneumonia and Hib age distributions, modelled in DisMod, to determine the PAF by age. Finally, we estimated PAFs for location and year using vaccine coverage modelled estimates.

We estimated the PAF for influenza and respiratory syncytial virus by calculating an attributable fraction using the following equation:

$$\text{PAF}=\text{Proportion}\times \left(1-\frac{1}{\text{OR}}\right)$$

where OR is the odds ratio of lower respiratory infection given pathogen detection^14^ and Proportion is the percentage of lower respiratory infection episodes that test positive for influenza (A and B) or RSV.^15^ We used both PCR and non-PCR diagnostic data, and adjusted the non-PCR data to be comparable with the PCR data in the modelling process (appendix pp 29, 30). We updated a systematic literature review to include all data from GBD 2015 and for papers published between Jan 1, 2015, and Dec 31, 2016, and used DisMod to model the proportion of lower respiratory infection cases that are positive for influenza and RSV, separately, by location, year, age, and sex. Because the case fatality ratio for viral lower respiratory infection is lower than for bacterial lower respiratory infection, we determined a scalar of this relationship and adjusted the PAFs accordingly (appendix pp 27–30).

### Risk factor attribution and decomposition

Methods for risk factor attribution to lower respiratory infection are described in detail elsewhere.^16^, ^17^ Briefly, risk factors also followed a PAF counterfactual approach, in which we modelled the prevalence of exposure using scientific literature and population representative surveys, and we modelled the relative risk of lower respiratory infection given exposure to that risk factor using published meta-analyses. We modelled the exposure level in a population for a given risk factor using DisMod and a spatiotemporal Gaussian process regression, depending on the risk factor.

To assess the effectiveness of targeted interventions for each risk factor among children younger than 5 years, we took advantage of the counterfactual definition of risk factor burden such that the lower respiratory infection mortality rate due to each risk factor was equivalent to the reduction expected in the complete absence of the risk factor. The number needed to treat is an epidemiological concept in which the rate of disease in two populations is compared.^18^ Since the counterfactual rate of disease is simply the difference between the lower respiratory infection mortality rate and the mortality rate due to the risk factor, the number needed to treat is the inverse of the lower respiratory infection mortality rate due to that risk factor (ie, the difference between the observed and counterfactual lower respiratory infection mortality rate in the absence of the risk factor). We did a decomposition analysis of the change in lower respiratory infection deaths from 2000–16 using the 12 risk factors associated with lower respiratory infection in GBD 2016. We chose this time period to show recent changes. We decomposed each risk factor independently; the decomposition assessed the change in lower respiratory infection mortality due to the risk factor, population growth, population ageing, and any remaining unexplained change in the lower respiratory infection mortality rate. This assumed independence overlooks well known correlation between risk factors. A combinatorial process determined the relative contribution of each component to the change in lower respiratory infection DALYs.^5^, ^16^, ^19^ We did not incorporate uncertainty into our risk factor decomposition.

### Role of the funding source

The funder of the study had no role in study design, data collection, data analysis, data interpretation, or writing of the report. The corresponding author had full access to all the data in the study and had final responsibility for the decision to submit for publication.

## Results

We estimated that in 2016, lower respiratory infections were a leading infectious cause of mortality worldwide in children younger than 5 years (under-5; 652 572 deaths, 95% UI 586 475–720 612), in adults older than 70 years (1 080 958 deaths, 943 749–1 170 638), and in people of all ages (2 377 697 deaths, 2 145 584–2 512 809; table 1). In 2016, lower respiratory infections caused 13·1% of all deaths in children younger than 5 years (95% UI 11·8–14·3) and 4·4% of all deaths in people of all ages (95% UI 3·9–4·6). Most of the deaths due to lower respiratory infection in children younger than 5 years occurred in the first year of life (491 900 deaths, 95% UI 444 000–541 400). Among children younger than 5 years, we estimated that there were 68·06 million episodes (95% UI 55·29–82·72) in 2016, equivalent to 0·11 cases per child-year (95% UI 0·09–0·13; table 1). We estimated there were 5 133 000 hospital admissions because of lower respiratory infections in 2016, among children younger than 5 years (95% UI 3 860 800–6 260 200), and 65 982 807 hospital admissions due to lower respiratory infections among all ages 56 813 819–75 511 038). The highest incidence of lower respiratory infection episodes among children younger than 5 years occurred in Oceania (171·5 per 1000 children, 95% UI 132·2–217·9; table 1) and the greatest number of lower respiratory infection episodes among children younger than 5 years occurred in south Asia (18·76 million [95% UI 15·58–22·04]). For people of all ages, we estimated that there were 336·46 million episodes of lower respiratory infection (95% UI 313·08–361·62) in 2016 (table 1). Lower respiratory infections were responsible for 56 107 300 DALYs (95% UI 50 447 100–61 890 000) among children younger than 5 years, corresponding to 61% of the total 91 844 600 DALYs (84 674 400–98 252 600) attributable to lower respiratory infections across all ages in 2016. The number of deaths due to lower respiratory infections decreased by 54·1% (95% UI 48·7–58·9) among children younger than 5 years and 13·4% among all ages (7·8–13·4) between 2000 and 2016.

**Table 1:** Episodes and deaths attributable to lower respiratory infections in 2016, by location

		**All ages**				**Children younger than 5 years**				**Adults older than 70 years**			
		Deaths (95% UI)	Deaths per 100 000 people (95% UI)	Millions of episodes (95% UI)	Episodes per 1000 people (95% UI)	Deaths (95% UI)	Deaths per 100 000 people (95% UI)	Millions of episodes (95% UI)	Episodes per 1000 people (95% UI)	Deaths (95% UI)	Deaths per 100 000 people (95% UI)	Millions of episodes (95% UI)	Episodes per 1000 people (95% UI)
Global		2 377 697 (2 145 584–2 512 809)	32·2 (29·0–34·0)	336·46 (313·08–361·62)	45·5 (42·4–48·9)	652 572 (586 475–720 612)	103·3 (92·8–114·0)	68·06 (55·29–82·72)	107·7 (87·5–130·9)	1 080 958 (943 749–1 170 638)	267·4 (233·4–289·6)	62·84 (57·15–68·75)	155·4 (141·4–170·1)
High income		392 940 (362 375–425 101)	37·0 (34·1–40·0)	38·13 (35·84–40·52)	35·9 (33·8–38·2)	1905 (1749–2105)	3·3 (3·0–3·6)	2·58 (2·05–3·25)	44·6 (35·5–56·3)	337 271 (307 354–367 534)	252·5 (230·1–275·1)	13·49 (12·32–14·75)	101·0 (92·2–110·4)
	High-income North America	105 127 (98 114–112 332)	29·3 (27·3–31·3)	13·93 (13·12–14·73)	38·8 (36·5–41·0)	669 (613–730)	3·1 (2·8–3·4)	1·19 (0·97–1·46)	55·1 (44·8–67·5)	81 270 (74 817–87 883)	227·6 (209·6–246·2)	2·61 (2·38–2·84)	73·2 (66·7–79·5)
	Australasia	5164 (4532–5848)	18·0 (15·8–20·4)	1·26 (1·17–1·36)	43·9 (40·7–47·5)	36 (30–44)	2·0 (1·7–2·5)	0·11 (0·08–0·14)	60·5 (46·4–77·8)	4623 (3999–5305)	161·8 (140·0–185·6)	0·47 (0·42–0·53)	165·6 (147·4–184·8)
	High-income Asia Pacific	109 683 (98 788–121 621)	60·8 (54·8–67·4)	8·12 (7·56–8·73)	45·0 (41·9–48·4)	194 (171–220)	2·6 (2·3–3·0)	0·48 (0·36–0·62)	64·6 (48·9–84·8)	100 501 (90 040–112 207)	337·2 (302·1–376·4)	3·59 (3·25–3·96)	120·6 (109·1–132·9)
	Western Europe	138 945 (126 055–152 808)	32·4 (29·4–35·7)	12·28 (11·47–13·20)	28·7 (26·8–30·8)	379 (345–425)	1·7 (1·6–1·9)	0·51 (0·39–0·67)	23·4 (17·9–30·5)	124 805 (112 377–138 463)	207·4 (186·7–230·1)	6·10 (5·56–6·67)	101·3 (92·4–110·8)
	Southern Latin America	34 021 (30 754–37 110)	52·1 (47·1–56·8)	2·54 (2·38–2·74)	38·9 (36·4–41·9)	626 (513–761)	12·5 (10·2–15·2)	0·29 (0·23–0·36)	57·8 (46·5–72·0)	26 071 (23 187–28 951)	516·2 (459·1–573·3)	0·71 (0·63–0·79)	140·6 (125·2–156·5)
Central Europe, eastern Europe, and central Asia		97 153 (86 019–110 400)	23·4 (20·7–26·6)	27·14 (25·20–29·20)	65·3 (60·6–70·3)	17 025 (13 377–22 085)	60·4 (47·4–78·3)	3·02 (2·41–3·71)	107·1 (85·4–131·7)	31 483 (28 367–34 849)	85·2 (76·7–94·3)	6·32 (5·59–7·09)	171·1 (151·1–191·9)
	Eastern Europe	46 126 (36 064–58 694)	21·8 (17·1–27·8)	17·18 (15·84–18·62)	81·2 (74·9–88·0)	1791 (1454–2219)	13·8 (11·2–17·1)	1·46 (1·15–1·83)	112·6 (88·5–141·5)	11 363 (9178–14 151)	54·8 (44·3–68·3)	4·21 (3·67–4·78)	203·0 (177·0–230·7)
	Central Europe	26 325 (24 435–28 353)	22·8 (21·1–24·5)	4·79 (4·48–5·13)	41·4 (38·7–44·4)	795 (674–939)	14·2 (12·1–16·8)	0·54 (0·43–0·67)	96·7 (76·9–119·7)	17 366 (15 787–19 073)	134·3 (122·1–147·5)	1·56 (1·42–1·73)	120·6 (109·6–133·4)
	Central Asia	24 702 (20 925–29 866)	28·0 (23·7–33·8)	5·17 (4·77–5·59)	58·5 (54·1–63·3)	14 439 (10 821–19 609)	149·7 (112·2–203·3)	1·02 (0·81–1·24)	105·7 (84·0–128·2)	2753 (2488–3027)	82·9 (74·9–91·2)	0·56 (0·50–0·62)	168·5 (150·3–188·3)
Latin America and Caribbean		160 842 (150 906–170 365)	27·9 (26·2–29·5)	23·60 (22·05–25·23)	40·9 (38·2–43·8)	21 838 (19 982–24 428)	44·0 (40·3–49·2)	4·71 (3·82–5·70)	94·9 (77·0–114·8)	89 805 (81 557–97 556)	326·2 (296·2–354·3)	6·45 (5·91–7·05)	234·4 (214·6–256·0)
	Central Latin America	43 191 (40 602–46 405)	17·0 (15·9–18·2)	5·30 (4·89–5·75)	20·8 (19·2–22·6)	9162 (8326–10 403)	40·1 (36·4–45·5)	1·32 (1·06–1·64)	57·8 (46·4–71·7)	19 219 (17 262–21 205)	175·3 (157·5–193·5)	1·22 (1·10–1·34)	111·2 (100·6–122·1)
	Andean Latin America	28 653 (24 397–32 571)	47·9 (40·7–54·4)	3·76 (3·50–4·03)	62·8 (58·4–67·4)	4813 (3939–5793)	72·2 (59·1–86·9)	0·77 (0·62–0·93)	115·5 (93·8–140·1)	17 032 (13 894–19 794)	636·5 (519·2–739·7)	1·09 (0·98–1·19)	406·5 (365·1–446·1)
	Caribbean	16 895 (15166–18 887)	36·9 (33·2–41·3)	2·81 (2·60–3·03)	61·4 (56·9–66·3)	3128 (2046–4702)	78·4 (51·3–117·9)	0·60 (0·48–0·74)	150·1 (119·3–185·9)	9871 (8822–10 866)	344·3 (307·6–378·9)	0·83 (0·74–0·92)	288·9 (258·3–320·1)
	Tropical Latin America	71 338 (66 753–75 856)	33·0 (30·8–35·0)	11·74 (10·99–12·54)	54·2 (50·7–57·9)	4639 (4197–5173)	28·8 (26·1–32·1)	2·02 (1·64–2·44)	125·3 (101·8–151·5)	43 198 (39 301–46 840)	391·7 (356·3–424·7)	3·32 (3·03–3·61)	301·0 (274·8–327·7)
Southeast Asia, east Asia, and Oceania		380 060 (328 336–412 923)	18·2 (15·8–19·8)	75·11 (69·18–81·50)	36·1 (33·2–39·1)	60 156 (53 483–67 970)	48·9 (43·5–55·2)	14·82 (11·75–18·29)	120·4 (95·5–148·6)	214 284 (179 802–237 500)	190·3 (159·6–210·9)	14·73 (13·26–16·29)	130·8 (117·7–144·6)
	East Asia	171 297 (148 361–203 061)	12·1 (10·5–14·3)	44·22 (40·74–48·08)	31·2 (28·7–33·9)	17 022 (14 554–19 437)	26·3 (22·5–30·1)	6·94 (5·43–8·64)	107·4 (84·0–133·6)	115 743 (97 715–137 327)	132·3 (111·7–157·0)	10·15 (9·15–11·23)	116·0 (104·7–128·4)
	Southeast Asia	201 790 (170 920–218 582)	30·8 (26·1–33·4)	30·04 (27·56–32·83)	45·9 (42·1–50·1)	40 181 (34 696–46 626)	70·5 (60·8–81·8)	7·63 (6·10–9·41)	133·9 (107·0–165·0)	97 141 (80 361–107 598)	389·8 (322·4–431·7)	4·51 (4·02–5·00)	181·0 (161·5–200·4)
	Oceania	6551 (4941–8959)	58·5 (44·1–79·9)	0·85 (0·77–0·94)	75·8 (68·3–83·8)	2907 (1640–4925)	205·7 (116·1–348·6)	0·24 (0·19–0·31)	171·5 (132·2–217·9)	1129 (887–1467)	452·9 (355·9–588·5)	0·07 (0·06–0·08)	295·0 (252·2–337·3)
North Africa and Middle East		106 410 (93 839–120 968)	18·5 (16·3–21·0)	32·49 (29·41–35·79)	56·5 (51·2–62·3)	39 718 (30 676–50 233)	62·9 (48·5–79·5)	8·42 (6·67–10·43)	133·2 (105·6–165·1)	33 134 (28 168–40 097)	187·8 (159·7–227·3)	4·35 (3·81–4·89)	246·6 (216·3–277·4)
South Asia		589 653 (496 203–642 836)	34·7 (29·2–37·8)	82·97 (78·21–88·05)	48·8 (46·0–51·8)	199 513 (175 850–223 300)	129·9 (114·5–145·4)	18·76 (15·58–22·04)	122·1 (101·4–143·5)	236 883 (173 779–269 243)	408·8 (299·9–464·6)	13·37 (12·46–14·35)	230·7 (214·9–247·7)
Sub–Saharan Africa		650 639 (582 183–720 960)	66·4 (59·4–73·6)	57·02 (52·29–62·28)	58·2 (53·4–63·5)	312 417 (266 256–361 152)	199·5 (170·0–230·6)	15·76 (12·72–19·44)	100·6 (81·3–124·2)	138 099 (116 652–157 256)	768·7 (649·3–875·3)	4·12 (3·63–4·66)	229·3 (201·8–259·1)
	Southern sub–Saharan Africa	47 384 (41 130–54 257)	61·6 (53·4–70·5)	5·52 (5·11–5·97)	71·7 (66·4–77·5)	10 819 (8731–13 304)	125·7 (101·4–154·5)	0·86 (0·69–1·05)	99·6 (79·8–121·7)	14 170 (11 840–16 383)	579·0 (483·8–669·4)	0·55 (0·49–0·61)	224·2 (198·7–250·2)
	Western sub–Saharan Africa	273 944 (236 313–315 218)	68·8 (59·3–79·2)	20·43 (18·74–22·25)	51·3 (47·0–55·9)	138 330 (110 473–170 088)	214·1 (171·0–263·2)	4·88 (3·94–5·98)	75·5 (61·0–92·6)	54 205 (44 239–64 416)	859·3 (701·3–1021·1)	1·52 (1·32–1·73)	241·3 (210·0–273·7)
	Eastern sub–Saharan Africa	251 054 (224 563–279 640)	64·9 (58·0–72·2)	23·82 (21·75–26·14)	61·5 (56·2–67·5)	121 120 (103 742–138 312)	193·6 (165·8–221·1)	7·61 (6·11–9·31)	121·6 (97·6–148·9)	55 332 (47 039–64 979)	770·0 (654·6–904·2)	1·59 (1·40–1·79)	221·5 (194·8–249·4)
	Central sub–Saharan Africa	78 087 (61 096–99 630)	66·3 (51·9–84·6)	7·25 (6·54–8·05)	61·6 (55·5–68·4)	42 084 (27 228–62 102)	202·4 (130·9–298·6)	2·41 (1·93–3·00)	116·1 (92·9–144·4)	14 338 (11 285–18 207)	708·5 (557·7–899·7)	0·46 (0·39–0·52)	225·8 (193·0–258·6)

Because of rounding, some values in the table are zero. UI=uncertainty interval.

**Figure 1:**
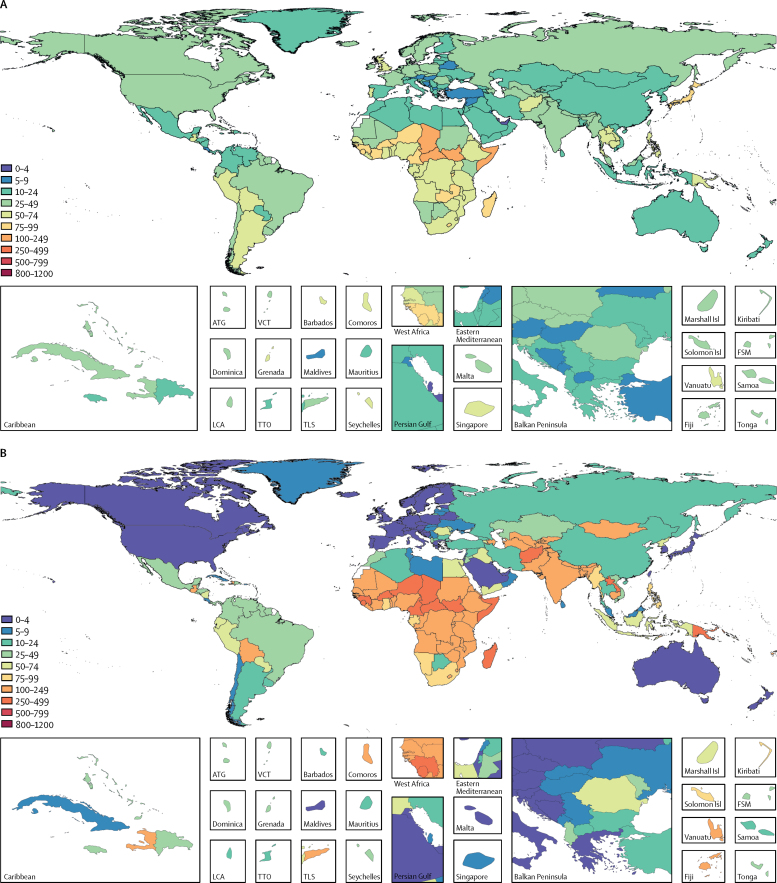

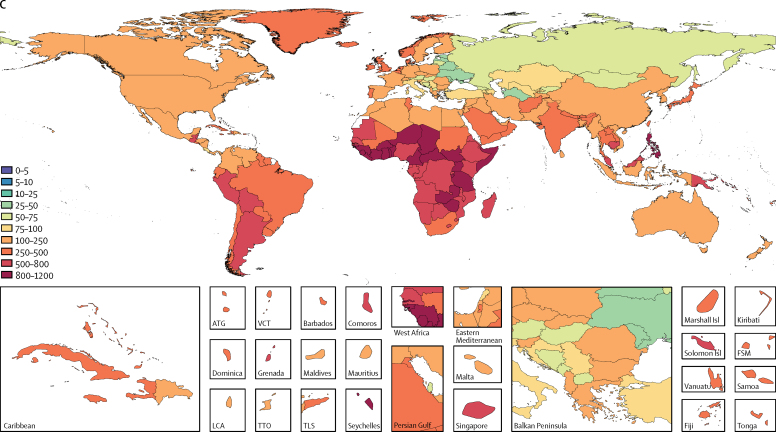
Global distribution of LRI mortality Lower respiratory infection mortality rate per 100 000 people for all ages (A), children younger than 5 years (B), and elderly adults (>70 years; C), in 2016. ATG=Antigua and Barbuda. VCT=Saint Vincent and the Grenadines. LCA=Saint Lucia. TTO=Trinidad and Tobago. Isl=Islands. FSM=Federated States of Micronesia. TLS=Timor-Leste.

Lower respiratory infection mortality in all ages was highest in sub-Saharan Africa, south Asia, and southeast Asia (figure 1). The highest rates of lower respiratory infection mortality among children younger than 5 years, in 2016, were in the Central African Republic (460 per 100 000 people, 95% UI 300–688), Chad (425 per 100 000 people, 305–576), and Somalia (417 per 100 000 people, 257–613; appendix p 45). Because of their large populations and high absolute mortality, nearly a third of all lower respiratory infection deaths in children younger than 5 years occurred in India (149 826 deaths, 95% UI 132 370–167 643) and Nigeria (57 446, 39 276–82 081; appendix p 43). The global lower respiratory infection mortality rate among children younger than 5 years decreased by 57·1% since 2000 (95% UI 52·0–61·5), while the incidence of lower respiratory infection decreased by 21·4% (18·8–24·3%) over the same time period.

Lower respiratory infection mortality is also high in the elderly (table 1, figure 1C). The number of lower respiratory infection deaths among adults aged 70 years and older increased from 746 700 (95% UI 672 300 to 806 700) in 2000, to 1 080 958 (943 749 to 1 170 638) in 2016. However, this increase has not been due to an increase in the lower respiratory infection mortality rate, which remained relatively constant in this age group between 2000 and 2016 (278·1 deaths per 100 000 people, 95% UI 250·4 to 300·5 in 2000; 267·4 deaths per 100 000 people, 233·4 to 290·6 in 2016). Globally, the number of adults older than 70 years increased by 50·6% between 2000 and 2016, from 268 522 838 to 404 287 666. Although the mortality rate in this age group has not changed appreciably in places like Brazil (18·1% increase, 95% UI 9·5 to 26·9) and India (7·1% increase, −9·3 to 25·2), or substantially in Thailand (78·5% increase, −25·9 to 161·8), the number of adults older than 70 years increased by 95·8% in Brazil, 71·2% in India, and 89·1% in Thailand between 2000, and 2016, and lower respiratory infection death counts have increased substantially in the past 16 years (131·2% in Brazil, 95% UI 114·5 to 148·5; 83·4% in India, 55·3 to 114·4; and 237·5% in Thailand, 40·1 to 395·0). As countries transition from low-middle to high-middle sociodemographic index, the under-5 lower respiratory infection mortality rate decreases, but a parallel improvement in lower respiratory infection mortality rate among the elderly is generally not observed.^20^

**Figure 2:**
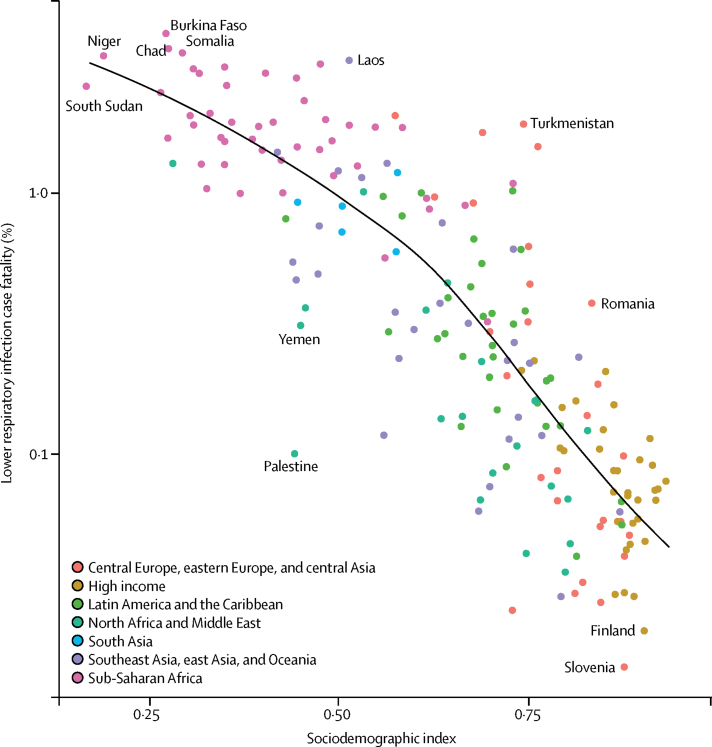
Fatality ratio and sociodemographic index for children younger than 5 years, 2016 The black line shows a smoothed loess curve for the relationship between sociodemographic index and lower respiratory infection case fatality ratio.

At the global level, the case fatality ratio due to lower respiratory infections decreased sharply among under 5s, from 2·33% (95% UI 2·12–2·55) in 1990, to 0·96% (0·87–1·06) in 2016. The countries with the highest case fatality ratios were western sub-Saharan Africa (Burkina Faso 4·11%, 95% UI 3·29–4·71; Chad 3·46%, 3·12–3·79) and eastern sub-Saharan Africa (Somalia 3·60%, 2·82–4·24), whereas the lowest occurred in the Balkan countries and a few high-income countries (Slovenia 0·015%, 0·014–0·016; Bosnia and Herzegovina 0·025%, 0·023–0·027%; and Croatia 0·027%, 0·024–0·029; figure 2).

**Figure 3:**
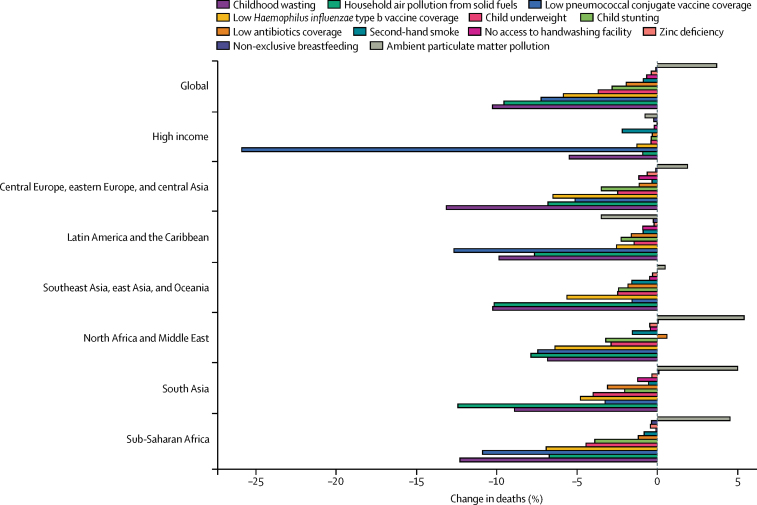
Percent change in deaths in children younger than 5 years, 2000–16

Our analysis showed that, of the four aetiologies studied, pneumococcal pneumonia was the leading cause of lower respiratory infection morbidity and mortality globally and caused more deaths than all other aetiologies combined: it was responsible for 1 189 937 deaths (95% UI 690 445–1 770 660) and 197·05 million episodes (112·83–287·64) in 2016 (table 2). RSV was the second leading aetiology of lower respiratory infection deaths overall (76 612, 95% UI 55 121–103 503) and 54% of lower respiratory infection deaths attributable to RSV occurred in children younger than 5 years (41 026, 22 922–65 851), similar to the number of under-5 lower respiratory infection deaths attributable to Hib (48 011, 13 404–88 744; table 2). Hib was not attributed to deaths or episodes in people older than 5 years. Influenza is more frequently associated with non-fatal lower respiratory infection episodes than with fatal episodes. It was responsible for the second fewest number of deaths but it was the second most common aetiology among LRI episodes (39·1 million episodes, 95% UI 30·5–48·4; table 2). Hib was not attributed to deaths or episodes in people older than 5 years because of insufficient data in these age groups. Because of expanded coverage of Hib and pneumococcal conjugate vaccines, in children younger than 5 years lower respiratory infection mortality due to Hib decreased by 5·86% and LRI mortality due to pneumococcal pneumonia decreased by 7·24% worldwide, with variation in super-region depending on the introduction and coverage of the vaccine (figure 3).

**Table 2:** Global deaths, fatal attributable fraction, and episodes due to each LRI aetiology, 2016

	**Deaths (95% UI)**	**Fatal attributable fraction (95% UI)**	**Deaths per 100 000 people (95% UI)**	**Millions of episodes (95% UI)**	**Incidence per 1000 people (95% UI)**
**All ages**					
*Streptococcus pneumoniae*	118 9937 (690445–1770660)	50·05% (29·22–73·94)	16·1 (9·3–24·0)	197·05 (112·83–287·64)	26·7 (15·3–38·9)
Respiratory syncytial virus	76 612 (55121–103503)	3·22% (2·32–4·40)	1·0 (0·7–1·4)	24·83 (19·65–31·42)	3·4 (2·7–4·3)
*Haemophilus influenzae* type b	48 011 (13404–88744)	2·02% (0·57–3·70)	0·6 (0·2–1·2)	6·73 (1·73–13·55)	0·9 (0·2–1·8)
Influenza	58 193 (43953–74175)	2·45% (1·86–3·10)	0·8 (0·6–1·0)	39·14 (30·54–48·44)	5·3 (4·1–6·6)
**Children younger than 5 years**					
*Streptococcus pneumoniae*	341 029 (195289–493551)	52·25% (29·95–73·00)	54·0 (30·9–78·1)	44·69 (20·87–73·68)	70·7 (33·0–116·6)
Respiratory syncytial virus	41 026 (22922–65851)	6·29% (3·58–10·17)	6·5 (3·6–10·4)	10·74 (6·70–16·56)	17·0 (10·6–26·2)
*Haemophilus influenzae* type b	48 011 (13404–88744)	7·36% (2·06–13·63)	7·6 (2·1–14·0)	6·08 (1·39–13·72)	9·6 (2·2–21·7)
Influenza	8360 (4905–13806)	1·28% (0·77–2·09)	1·3 (0·8–2·2)	5·75 (3·42–9·34)	9·1 (5·4–14·8)
**Elderly adults (>70 years)**					
*Streptococcus pneumoniae*	494 340 (209900–896430)	45·74% (19·50–82·10)	122·3 (51·9–221·7)	29·43 (11·34–57·02)	72·8 (28·1–141·0)
Respiratory syncytial virus	22 009 (15705–30787)	2·04% (1·46–2·82)	5·4 (3·9–7·6)	2·54 (1·98–3·15)	6·3 (4·9–7·8)
*Haemophilus influenzae* type b*	..	..	..	..	..
Influenza	24 803 (16704–34251)	2·29% (1·59–3·17)	6·1 (4·1–8·5)	6·37 (4·79–8·16)	15·8 (11·8–20·2)

UI=uncertainty interval.

* *Haemophilus influenzae* type b was not attributed to deaths or episodes in people older than 5 years.

Childhood wasting remains the leading risk factor for lower respiratory infection mortality among children younger than 5 years, responsible for 57·1% of lower respiratory infection deaths (95% UI 30·1–66·7) in 2016 (appendix p 65). However, between 2000, and 2016, a decomposition of the contribution of the leading risk factors for lower respiratory infection mortality among children younger than 5 years showed that the largest reduction in lower respiratory infection mortality was due to improvements in childhood wasting, which was responsible for a 10·3% decrease (146 000 fewer deaths than if the prevalence of wasting had remained at the same level as in 2000; figure 3). Prevalence of childhood wasting improved in most locations; the largest decreases occurred in Algeria (where a 32·2% decrease in wasting prevalence from 2000 to 2016, resulted in 800 fewer deaths than if no decrease had occurred) and the Democratic Republic of the Congo (31·1% decrease, 10 700 fewer deaths; appendix p 66). We found an exception in Madagascar, where a greater prevalence of wasting contributed to a 36·0% increase in deaths (4100 excess deaths; appendix p 66). The second leading risk factor contributing to the reduction of under-5 lower respiratory infection mortality was a reduction in household and solid fuel air pollution, which were responsible for a 9·6% global decrease in deaths (135 000 fewer deaths; appendix p 66). Despite this decrease, this shift coincided in many locations with an increased exposure to ambient particulate matter pollution, which was responsible for a 3·7% increase in under-5 lower respiratory infection mortality (figure 3). In several rapidly developing countries in south Asia (Bangladesh, India, and Nepal), reduced household pollution contributed to a decrease in lower respiratory infection mortality, but ambient particulate matter pollution contributed to an increase in lower respiratory infection mortality (figure 3; appendix p 66). The region as a whole reflected this (5·0% increase due to particulate matter pollution and 12·4% decrease due to reduced indoor air pollution), with large differences in Bangladesh (7·3% increase due to particulate matter pollution and 7·5% decrease due to indoor air pollution) and India (5% increase due to particulate air pollution and 13·4% decrease due to indoor air pollution).

**Figure 4:**
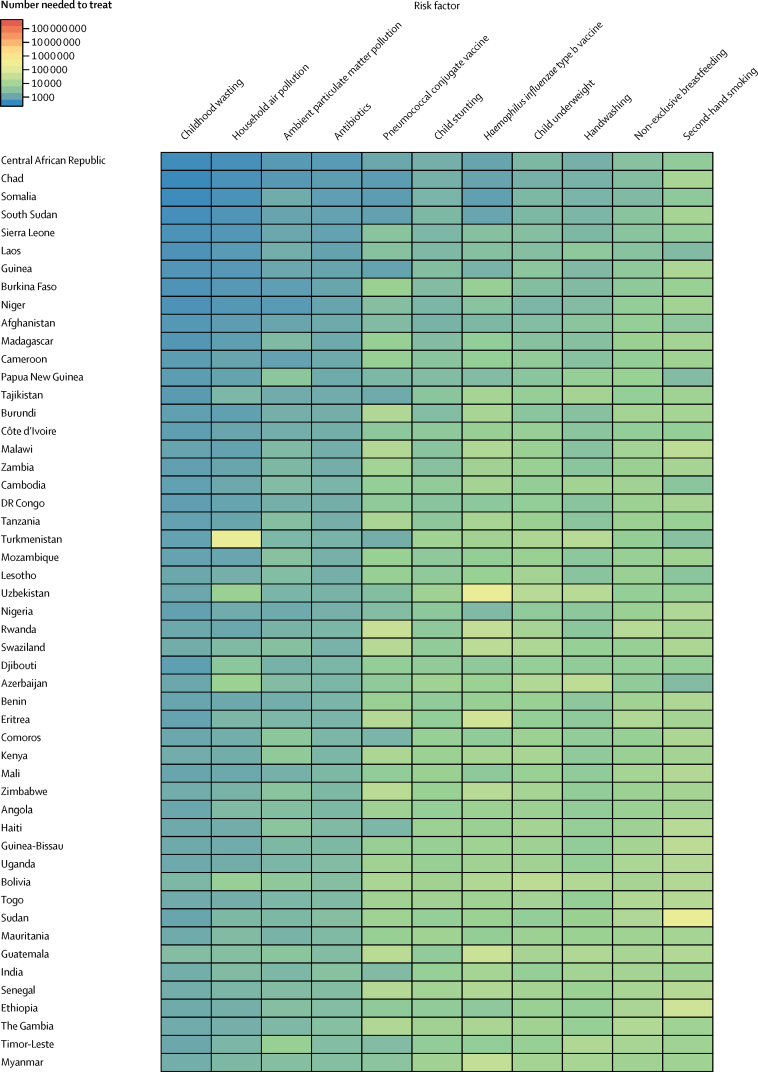

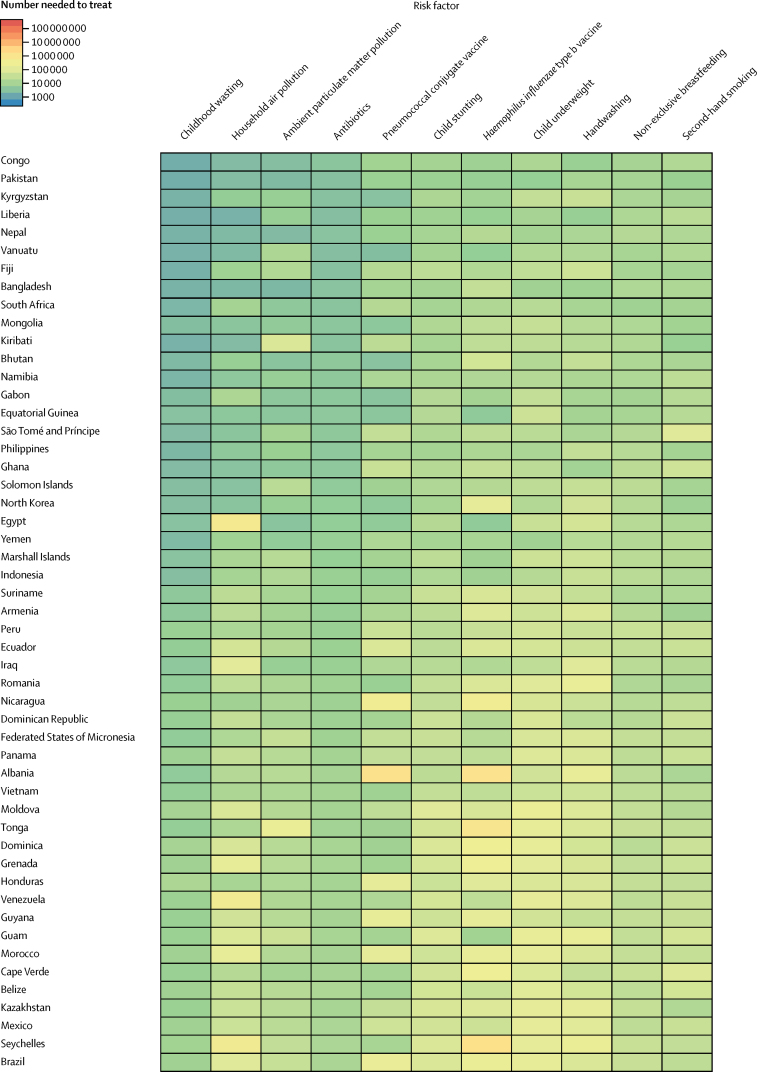

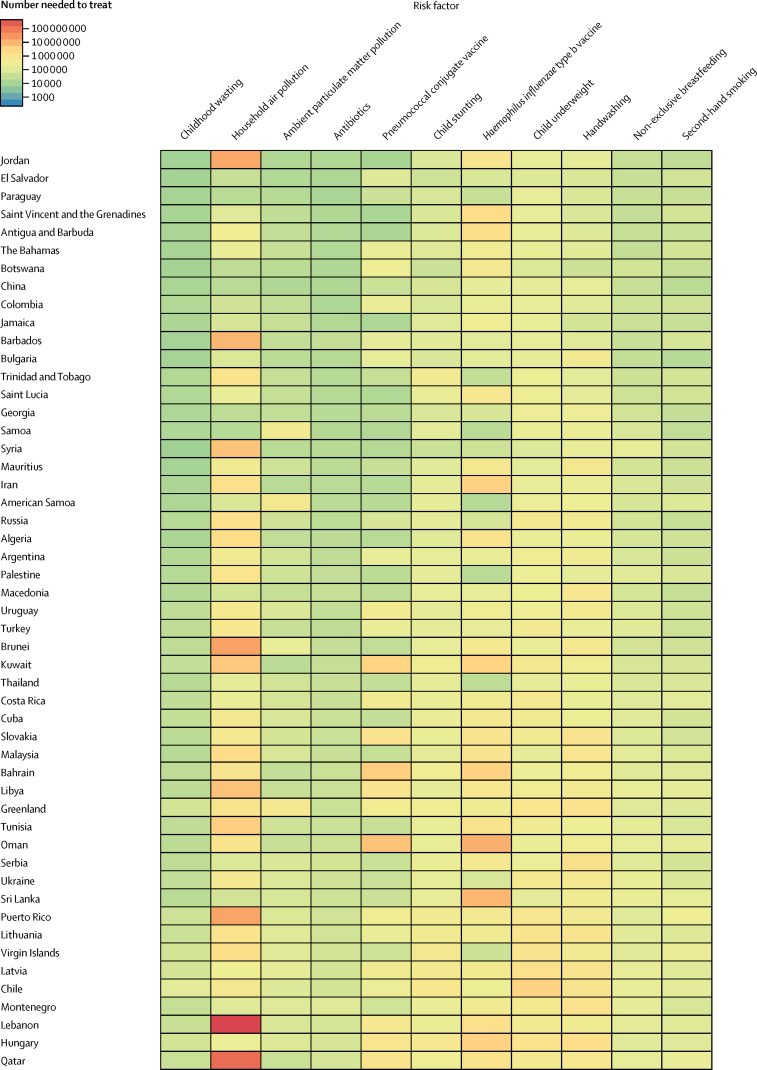

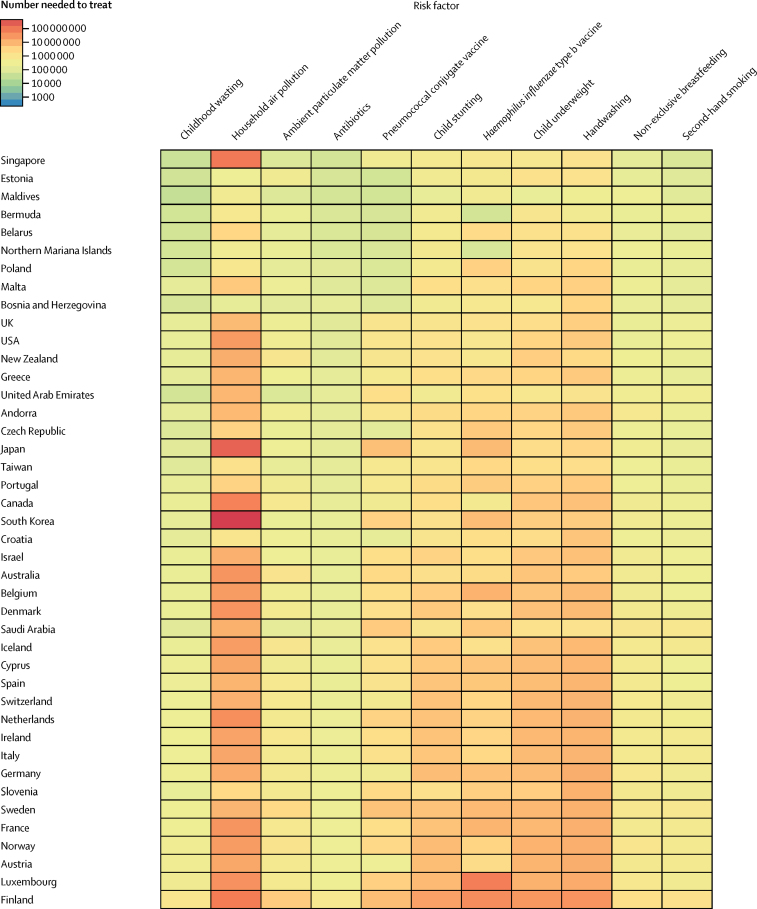
Number of children younger than 5 years needed to treat to prevent a death due to lower respiratory infection, 2016

Among the countries with the highest lower respiratory infection mortality rate, interventions to reduce childhood wasting prevalence, improve household and ambient particulate matter pollution, and expand antibiotic treatment could avert an under-5 death due to lower respiratory infection for every 4000 children treated (figure 4; appendix p 45). Specifically, for every 1576 children who improve their weight-for-height *Z* score to the global mean, one death due to lower respiratory infection could be averted (95% UI 1319–2117; figure 4). Household air pollution has the second lowest number needed to treat globally (2527 needed to treat to avert one death, 95% UI 1999–3410; figure 4). Despite the fact that antibiotic treatment improved in most locations, being responsible for a 1·94% global decrease in under-5 lower respiratory infection mortality between 2000, and 2016, 30·2% of lower respiratory infection deaths among children younger than 5 years were attributable to lack of antibiotic treatment (95% UI 14·4–43·8; appendix p 65), and appropriate antibiotic treatment could avert one under-5 death due to lower respiratory infection for every 3200 children with lower respiratory infection who are treated (95% UI 2000–7470; figure 4; appendix p 75). Although the pneumococcal conjugate vaccine prevented 52 000 under-5 lower respiratory infection deaths in 2016, the large remaining burden of pneumococcal pneumonia reflects the vaccine's low coverage in high-burden locations and the large attributable fraction of pneumococcal pneumonia in nearly every geography. Although reducing non-exclusive breastfeeding and exposure to second-hand tobacco smoke are the least efficient interventions at the global level, they are more efficient than targeting stunting, underweight, and handwashing in central Europe, central Asia, east Asia, and Latin America and the Caribbean (figure 4).

## Discussion

### Summary of findings

Substantial progress has been made to reduce the global burden of lower respiratory infections. However, this reduction has not been equal across locations, has been driven by decreases in several primary risk factors, and the burden in elderly adults could be growing and requires attention.

Our results suggest that the case fatality ratio among children younger than 5 years decreases rapidly with increasing sociodemographic development. There are several possible explanations for this observation, including improved access to health care, decreased exposure to disease risk, improved nutrition, and possibly improved case management.^21^ A key goal of the GAPPD was universal access to appropriate lower respiratory infection case management.^3^ More than 50% of lower respiratory infection deaths in 2016 were attributable to bacterial aetiologies. We found that antibiotic use has increased in most locations and contributed to a 1·94% decrease in under-5 lower respiratory infection mortality. Since the global number needed to treat is relatively small (3200), increased access to appropriate antibiotics could be an efficient intervention in many countries. We estimate that increased antibiotic availability could still prevent 30·2% (95% UI 14·4–43·8) of lower respiratory infection deaths. However, appropriate antibiotic use remains challenging—in large part because of the absence of reliable clinical signs and symptoms that can accurately predict bacterial pneumonia^22^, ^23^—and universal use of antibiotics for lower respiratory infections could lead to antimicrobial resistance.^24^ Although interventions to expand judicious antibiotic use and improve diagnostic accuracy might help to mitigate this risk, these efforts must be accompanied by interventions to prevent cases of lower respiratory infection.

Pneumococcal pneumonia is by far the leading aetiology responsible for lower respiratory infection incidence and mortality in children and adults. There is increasing evidence that *S pneumoniae* might be a co-infection or a follow-up infection to viral infections.^25^ However, given our counterfactual approach to aetiological attribution, it is possible to interpret our results as the expected reduction in lower respiratory infection burden in the total absence of an aetiology. Because our approach treats aetiological attribution independently, it is difficult to assess co-infections. The vaccine probe approach to aetiological attribution might miss indirect effects of the vaccine in protecting non-vaccinated people, a phenomenon that has been observed for pneumococcal conjugate vaccine. Despite this limitation, we observed a moderate reduction in lower respiratory infection mortality in children younger than 5 years after the introduction of pneumococcal conjugate vaccine in many countries, and the results suggest that a great deal of lower respiratory infection burden could be avertable if pneumococcal conjugate vaccine coverage was universal.

Risk factors for childhood undernutrition (stunting, wasting, and underweight) are among the leading risk factors for lower respiratory infection mortality in children younger than 5 years, and are strongly associated with poor health outcomes.^17^, ^26^ This association is probably due to impaired immunity caused by poor micronutrition and macronutrition. There are several reasons why many children in low-income countries, born underweight or not, tend to move further away from the global mean as they age. One reason might be a positive reinforcement loop that exists between infectious disease incidence, including lower respiratory infection and diarrhoea, which leads to poor physical growth, subsequently predisposing children to future episodes of infectious diseases.^27^, ^28^, ^29^ Breaking these interactions has proved challenging, but some countries, such as Algeria, and the Democratic Republic of the Congo, have improved more than others with respect to childhood nutrition. This success might be due in part to improved maternal education, prenatal care, and interventions that target mothers and children.^30^, ^31^, ^32^ Because lower respiratory infections can predispose (through undernutrition) or be concurrent with other underlying causes of death, such as HIV, there could be an even greater burden of the disease since the GBD attributes each death to only one underlying cause, which is consistent with the core principle of ICD codes reflecting the condition responsible for the causal pathway that ends in mortality.^1^, ^20^

Exposure to indoor household solid fuel use and ambient particulate matter—the two key components of air pollution—varies widely, and they might be trending in opposite directions.^33^ As countries develop in sociodemographic index, indoor air pollution tends to decrease as cooking shifts from biofuels to natural gas and electricity.^34^ At the same time, rapid proliferation of cheap fossil-fuel-based energy has led to much greater exposure to ambient particulate matter pollution. This paradox is a challenge of development. Reducing exposure to both types of air pollution is likely to be an efficient means to reduce lower respiratory infection mortality among children younger than 5 years. These risk factors, while ubiquitous in prevalence, have small associations with lower respiratory infection risk.^33^, ^35^, ^36^, ^37^ Several studies have identified only small effects, if any, of household air pollution on lower respiratory infection risk, and trials investigating indoor air pollution interventions have not shown major reductions in lower respiratory infection incidence.^38^, ^39^, ^40^ Even if the improvements in lower respiratory infection mortality are lower than we estimated, improving air quality is a worthwhile goal for cognitive development, asthma, and other respiratory and cardiac outcomes.^33^

Our results suggest that nearly three-quarters of lower respiratory infection deaths occurred in older children and adults, with a particularly high burden in adults older than 70 years. Mortality rates in adults older than 70 years have remained remarkably consistent in most regions of the world since 1990, while the population of elderly adults has increased globally; there are twice as many adults older than 70 years in 2016, compared with 1990. Much of the global initative to reduce the lower respiratory infection burden has been focused in children younger than 5 years, but our results suggest a growing need to expand this focus to include elderly populations. Further exploration of the effect of comorbidities and other risk factors that put older adults at risk of lower respiratory infection should be investigated, and exploration of the effect of pneumococcal conjugate vaccine in elderly populations in low-income and middle-income countries LMICs is warranted.

### Comparison with other estimates

The GBD 2016 estimates of lower respiratory infection mortality in children younger than 5 years in 2015 (700 554, 95% UI 635 081–767 610) were nearly identical to the estimates produced for GBD 2015 in the same year (703 918, 651 385–763 039; appendix p 32). However, the number of lower respiratory infection deaths among people of all ages in 2015 (2 388 748, 95% UI 2 157 662–2 520 283) was smaller than the number of deaths estimated for GBD 2015 (2 736 714, 2 500 318–2 860 843; appendix p 33).^5^ This discrepancy is partly due to changes in the data sources used for lower respiratory infection mortality modelling. Notably, in India, reliable Sample Registration System data covering all states were introduced for GBD 2016. Moreover, a new process introduced in GBD 2016 to maintain consistency in estimates between cognitive diseases in elderly adults, such as Alzheimer's disease and Parkinson's disease, re-distributed more lower respiratory infection deaths in age groups of adults older than 65 years to those causes of death than previously.

Our estimates of lower respiratory infection mortality in children younger than 5 years differ from those produced by the WHO Department of Evidence, Information and Research, and the Maternal and Child Epidemiology Estimation (MCEE) group, which estimates deaths due to pneumonia. The GBD 2016 estimates for under-5 mortality due to pneumonia in 2016 (700 554, 95% UI 635 081–767 610) are lower than those from the MCEE (920 136).^41^, ^42^ The source of much of this discrepancy is due to differences in the number of deaths in the two highest burden countries, India and Nigeria, where differences arise from input data and modelling approach (appendix pp 36–38). Our estimates of the number of lower respiratory infection episodes among children under-5 in 2010 (74 130 000, 95% UI 60 610 000–89 700 000)^43^ are about half that of the numbers estimated by the MCEE group (120 400 000, 60 800 000–277 000 000) but with overlap in the 95% uncertainty intervals (appendix p 40). The estimates produced by the MCEE are informed by the incidence of pneumonia in 35 cohort studies, which informed an overall incidence of pneumonia that was related to the prevalence of five risk factors for pneumonia to estimate country-level incidence.^44^ Although there is overlap in the risk factors used in both studies, GBD 2016 used more than 30 000 datapoints from more than 700 sources to produce internally consistent estimates of lower respiratory infection incidence, prevalence, and mortality.

Estimates for the aetiologies of lower respiratory infection also differ from other recently published estimates. Shi and colleagues^45^ used a modelling strategy to estimate RSV incidence using 76 studies and several different approaches to defining a case fatality ratio. They estimated 59 600 (95% UI 48 000–74 500) deaths due to RSV among children younger than 5 years in 2015, and potentially as many as 118 200 (94 600–149 400), depending on model assumptions. By contrast, we estimated 41 000 deaths (95% UI 22 900–65 900) in 2016, with overlap in the uncertainty intervals. Iuliano and colleagues^46^ estimated between 291 200 and 645 800 deaths associated with influenza among people of all ages in 2015, much higher than the GBD 2016 estimate of 58 200 (44 000–74 200). Although there are several differences in the approach to make these estimates, a major difference is the definition of an influenza death. In GBD 2016, we report the number of lower respiratory infection deaths attributable to influenza, whereas Iuliano and colleagues reported the number of respiratory deaths associated with influenza.^46^

### Data limitations

Our estimates of lower respiratory infection burden are limited by data availability. In particular, the regions of the world with the highest lower respiratory infection morbidity and mortality tend to be the places with the poorest data coverage, particularly for lower respiratory infection mortality and aetiological attribution. A detailed analysis done for GBD 2016 found that nearly all countries in Africa had poor vital record data, as measured by indices such as completeness and detail,^1^ with only Egypt receiving a 3/5 grade (where 1 is worst and 5 is best, and 3/5 was the best grade received). Better surveillance systems, including standard reporting mechanisms and case definitions, in Africa and south and southeast Asia would substantially reduce a major source of uncertainty in the lower respiratory infection mortality estimates.^47^ The predictive modelling approaches used in GBD 2016 rely on covariates and shared information across space and time to fill in these areas and the data gaps. Uncertainty is carried through each step of the lower respiratory infection modelling process and is represented in the uncertainty intervals for the results.

In recent years, PCR has become the gold standard for respiratory pathogen detection. However, even with the improved sensitivity of PCR diagnostic techniques, data about lower respiratory infection aetiologies remain sparse, partly because of challenges in obtaining appropriate samples from the site of infection, detection in healthy individuals, and relatively few data about aetiologies in adults.^48^, ^49^ Because of an absence of data in older children and adults, we assumed that the relationship between RSV and influenza and lower respiratory infections are the same in children younger than 5 years and in adults, which might not be true. Each iteration of the GBD study adds additional data that were either previously unidentified or have been published since the previous study, and GBD 2016 had an increase of 38% in available data for RSV and 20% more for influenza. Additionally, the relationship between disease severity, disease mortality, and aetiological attribution has not been well characterised for all pathogens and settings. Additional studies on lower respiratory infection aetiologies, including those in adults, which give detailed information on diagnostics, sample site, illness severity and co-infections, and with more discrete age ranges, are needed.

### Next steps

The GBD study will be updated annually, with the most recent results superseding previous results. This approach provides a unique opportunity to be readily adaptable to changes in methods, to incorporate new or previously unidentified data sources, and to be timely with estimates of mortality and morbidity due to lower respiratory infection. Lower respiratory infections can be frequently associated with sepsis, which has not yet been estimated for the GBD. Recommended treatment for bacterial lower respiratory infections, with or without sepsis, is antibiotic therapy. Antimicrobial resistance is becoming more widely recognised as a leading global health threat, and antimicrobial resistant will be quantified.

There might be substantial subnational variation in the burden of lower respiratory infection within countries. For example, an analysis of GBD 2016 focusing on India^50^ found a seven-fold variation in lower respiratory infection mortality between states. To further investigate such variation, work to estimate the burden of lower respiratory infection at very fine spatial resolutions, similar to estimates of malaria and under-5 mortality,^51^, ^52^ will provide detailed evidence to guide policy at the local level and direct interventions to where they could affect the most change. This type of precision public health requires strong surveillance systems, sophisticated analytical approaches, and the capacity to act on the results.^53^

## Conclusions

Despite noteworthy decreases in the number of deaths due to lower respiratory infections since 2000, there remains an urgent need to accelerate efforts to reduce the burden of disease in the most susceptible populations, particularly those with high prevalence of childhood undernutrition, exposure to air pollution, and poor access to health care. The GBD study provides a comprehensive, comparable, scientific set of estimates for the burden of lower respiratory infections and these estimates provide both a description of the epidemiology of the disease and a potential roadmap for continuing to reduce the burden of the disease.

For more on the **GBD 2016 data and code** see https://ghdx.healthdata.org/
