# Supplementary material for: Estimates of the global, regional, and national morbidity, mortality, and aetiologies of lower respiratory infections in 195 countries, 1990–2016: a systematic analysis for the Global Burden of Disease Study 2016
Source: Lancet Infect Dis. 2018 Nov;18(11):1191–210. doi: 10.1016/S1473-3099(18)30310-4 (PMC6202443; doi:10.1016/S1473-3099(18)30310-4)
Supplement: Supplementary appendix [file mmc1.pdf]

THE LANCET
Infectious Diseases

Supplementary appendix

This appendix formed part of the original submission and has been peer reviewed. We post it as supplied by the authors.

Supplement to: GBD 2016 Lower Respiratory Infections Collaborators. Estimates of the global, regional, and national morbidity, mortality, and aetiologies of lower respiratory infections in 195 countries, 1990–2016: a systematic analysis for the Global Burden of Disease Study 2016. *Lancet Infect Dis* 2018; published online Sept 19. http://dx.doi.org/10.1016/S1473-3099(18)30310-4.

Appendix to: The global burden of lower respiratory infections: results from the Global Burden of Diseases, Injuries, and Risk Factors (GBD) 2016 Study

This appendix provides methodological detail, supplemental figures, and comprehensive information on input data and data transformation.

Contents

Tables, Figures, and Flowcharts 2

Summary of LRI mortality modelling 3

Summary of LRI morbidity modelling 8

Summary of aetiology population attributable fraction strategy 20

Pneumococcal pneumonia and Hib 20

Influenza and RSV 27

Comparison to GBD 2015 32

Comparison with other estimates 36

References 40

Supplementary Results 42

Tables, Figures, and Flowcharts

Tables

Appendix Table 1. Summary of cause-specific mortality modelling input data 5
Appendix Table 2. Covariates in CODEm 6
Appendix Table 3. Severity definitions 9
Appendix Table 4. The number of data points by source type used in GBD 2016 9
Appendix Table 5. Case definitions and adjustments for LRI 13
Appendix Table 6. Covariates in the LRI DisMod model 19
Appendix Table 7. Sources in the Hib and Streptococcus pneumoniae vaccine efficacy meta-analysis 21
Appendix Table 8. Summary of LRI aetiology data used in attributable fraction estimation 29
Appendix Table 9. The adjustments for non-reference case definition used in modelling influenza and RSV 29
Appendix Table 10. The median values for the ratio of case fatality for viral to bacterial pneumonia 30
Appendix Table 11. The number of deaths due to lower respiratory infections 39
Appendix Table 12. Episodes and deaths among all ages, children under 5, and adults over 70 in 2016 by geography 45
Appendix Table 13. The population attributable fraction for risk factors associated with LRI deaths, globally, among children under-5 in 2000 and 2016. 65
Appendix Table 14. The percent change in under-5 LRI mortality attributable to change in risk factors between 2000 and 2016 by GBD region and country. 66
Appendix Table 15. The number needed to treat to prevent one under-5 death due to LRI in 2016 76

Figures

Appendix Figure 1. LRI mortality data geographic distribution 5
Appendix Figure 2. Geographic distribution of LRI morbidity modelling 10
Appendix Figure 3. LRI seasonality adjustment 14
Appendix Figure 4. The seasonality sinusoidal regression fit is shown for each GBD super-region 15
Appendix Figure 5. Age distribution of the pneumococcal pneumonia base population attributable fraction 23
Appendix Figure 6. Vaccine coverage estimates for all ages 25
Appendix Figure 7. Geographic distribution of aetiology data 28
Appendix Figure 8. Scatterplot of GBD 2015 and GBD 2016 deaths by country among children under-5 in 2015 33
Appendix Figure 9. The number of deaths over time for all ages and for children under-5 in GBD 2015 and GBD 2016 34
Appendix Figure 10. Aetiologic attribution to under-5 LRI deaths in 2010 35
Appendix Figure 11. Scatterplot of under-5 LRI deaths in comparing GBD 2016 and the WHO-MCEE group final estimates.[19] 37
Appendix Figure 12. Clustered bar chart of LRI aetiologies in 2016 63

Flowcharts

Appendix Flowchart 1. Fatal LRI Modelling 3
Appendix Flowchart 2. Non-fatal LRI modelling 8

Summary of LRI mortality modelling

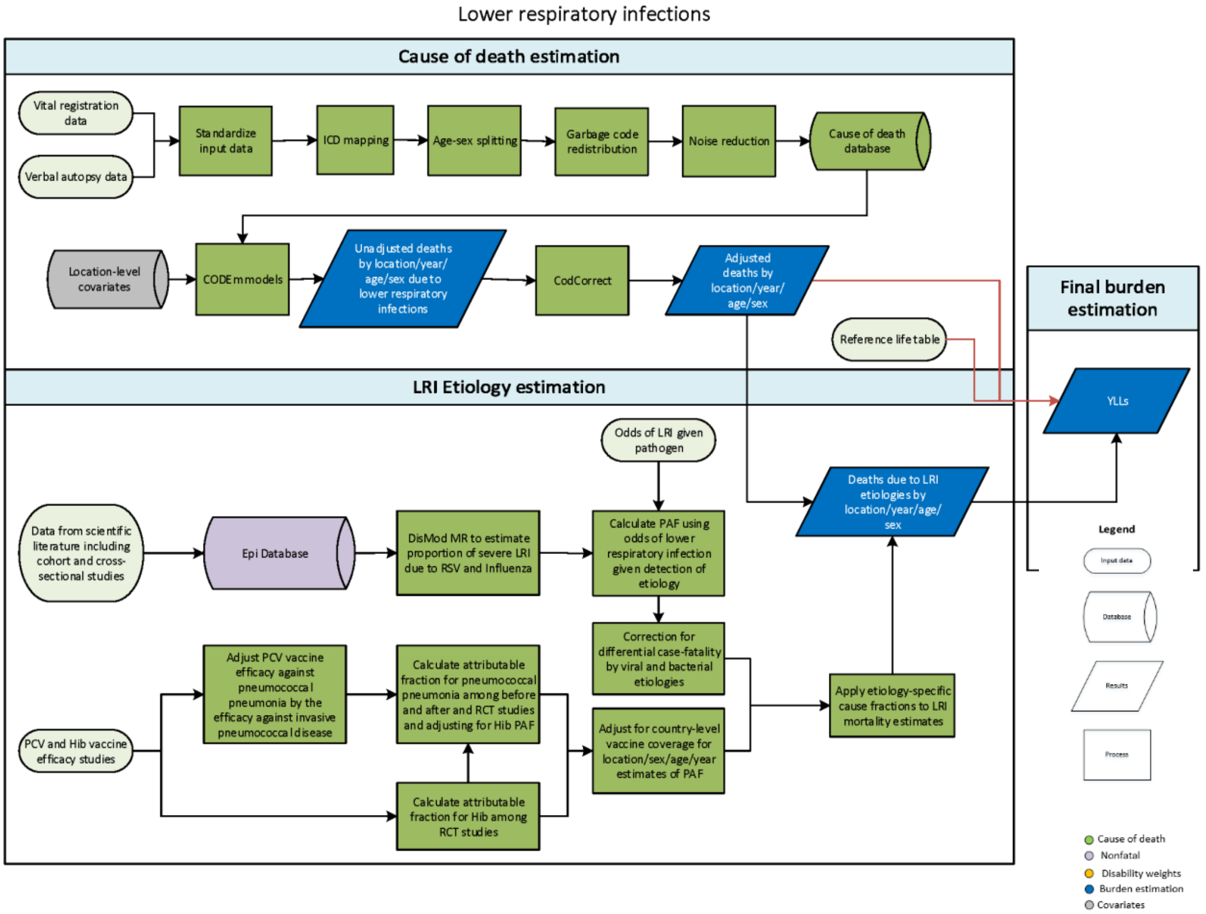

Appendix Flowchart 1. Fatal LRI Modelling

This flowchart shows the detailed analytic strategy for the fatal LRI modelling including the aetiologic attribution to LRI morbidity. Each of these steps will be discussed in greater detail in the section that follows.

Input data were all available data from vital registration systems, surveillance systems, and verbal autopsy. We first identified verbal autopsy studies, irrespective of cause, by searching PubMed and Google Scholar for all studies with the term "verbal autopsy", and did country-specific searches on Google using the country name and "verbal autopsy". LRI mortality was identified by ICD9 and ICD10 codes (ICD9 Codes included 073·0-073·6, 079·82, 466-469, 480-489, 513·0, and 770·0. ICD10 codes included A48·1, J09-J22, J85·1, P23-P23·9, and U04). We included studies that used verbal autopsy, had over 50 deaths, provided the number of deaths with LRI as the underlying cause, and were conducted for at least one year to account for seasonality**.** There were 801,600 data points on LRI mortality that were used in the modelling (**Appendix Figure 1 and Appendix Table 1**). We checked for and excluded outliers from our data by country or region. We excluded ICD9-coded mortality data in Sri Lanka (1982, 1987–1992), ICD9-coded neonatal mortality data in Guatemala (1980, 1981, 1984, 2000–2004), and Civil Registration System data in some Indian states (1986–1995). Overall, 3013 data points were excluded or outliered (0·6% of data points).

A key component of cause of death modelling in GBD is the redistribution of poorly coded causes of death such as “infection”, “fever”, or “dehydration” to specific causes of death.[1] This processing of *garbage* codes, causes of death that cannot or should not be considered underlying causes of death, reallocates a number of deaths from these non-specific causes to LRI. The garbage code redistribution was informed by an IHME expert review of the data and subsequent modelling.[1] Data points were split into GBD age groups using the global mortality age pattern for LRI. An overall mortality envelope and population estimates by age, sex, and location were used to calculate cause fraction and mortality rate.

Lower respiratory infection mortality was estimated in the Cause of Death Ensemble model (CODEm) platform.[2,3] CODEm is a Bayesian statistical model and uses spatial priors from a hierarchical structure to inform the mortality models. CODEm is based on five general principles: identifying all available data, maximising the comparability and quality of the dataset, developing a diverse set of plausible models, assessing the predictive validity of each plausible individual model and of ensemble models, and choosing the model or ensemble model with the best performance in out-of-sample predictive analysis. CODEm produces a large suite of models based on either cause fraction or mortality rate, uses linear and space-time Gaussian process regression (ST-GPR), and a covariate selection process. Each sub-model is evaluated using out-of-sample predictive validity. Thirty percent of the data are excluded from the initial model fits and 15% are used to evaluate component models and 15% used to build the ensembles. The sub-models are ranked using 15% of the data based on their out-of-sample predictive validity. The proportion weighting of the ensemble sub-models is evaluated using the remaining 15% of the hold-out data. This weighting scheme evaluates ensemble models that are built with ranked sub-models contributing proportionally more or fewer draws to the final ensemble. The final ensemble model is evaluated against other ensemble models using the same fit statistics (in-sample, out-of-sample root mean squared error and data coverage). Detailed information on this process can be found in Foreman et al 2012[4] and in the GBD 2016 Mortality and Causes of Death manuscript.[5]

Covariates are selected independently for each sub-model and the selection is based on an algorithm that captures biologically plausible relationships between the covariates and LRI mortality and provides a diversity of possible models. A list of covariates that the models select from is provided in **Appendix Table 2**. For every covariate, the direction of effect and a level of biologic proximity to LRI mortality was defined by the modeller. Each model includes all combinations of covariates if the direction of effect is along the assumed direction and the coefficient is significant at the $p < 0{\cdot}05$ level. Also, if adding a higher level covariate changes the significance of a level one to non-significant or an implausible direction, it will be dropped from the set. The reason for this algorithm is to give priority and emphasis on covariates that are more causally and proximately related to LRI such as air pollution and malnutrition rather than more contextual and macro covariates such as education and income per capita.

LRI mortality is estimated for 23 age groups, 774 locations, both sexes, and every year from 1980-2016. We estimated LRI mortality separately for males and females and for children under 5 years and older than 5 years due to expected underlying differences in the risk of mortality between these age groups. Data-rich and data-poor geographic locations were modelled separately and these models were then hybridised for a global model. This was to maintain proper uncertainty in the models where trusted data on causes of death exist. For a detailed description of the input data coverage, completeness, and reliability of the cause of death data in GBD 2016, please refer to the scoring system introduced in the GBD 2016 Mortality Collaborators manuscript.[5]

LRI mortality estimates are then squeezed into an overall mortality envelope by age/sex/location/year in a process called CoDCorrect. This step is to ensure internal consistency among causes of death and that the sum of cause-specific mortality is the same as the estimated all-cause mortality.

Appendix Figure 1. LRI mortality data geographic distribution

The number of site-years of verbal autopsy or vital registration data for all ages and from 1980-2016 are shown. Countries in gray have no data. Input data and models can be found using the GBD visualization tools at: http://vizhub.healthdata.org/data-visualizations.

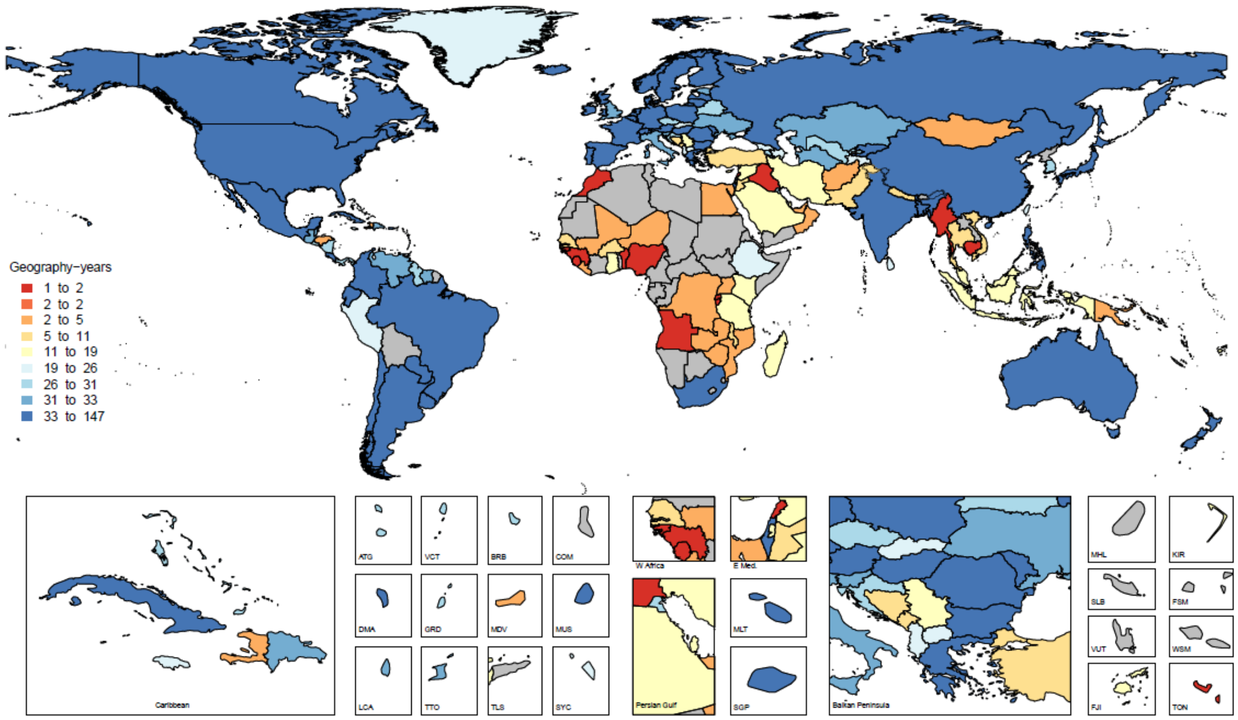

Appendix Table 1. Summary of cause-specific mortality modelling input data

**Type of data**	**Input data**
**Total data sources**	**12,155 site-years**
Vital registration data	10,312 site-years
Surveillance data	928 site-years
Verbal autopsy data	915 site-years

Appendix Table 2. Covariates in CODEm

CODEm uses a covariate selection algorithm and chooses from the covariates listed in the table below. Covariates are selected from this list while considering prior information about the strength of the association and direction of effect between the covariate and LRI mortality. The Level is ranked from 1 (in causal pathway) to 3 (likely related to LRI mortality). A) Under 5 years old model. B) Over 5 years old model.

Under 5 years old model

Covariate	Level	Direction
Hib vaccine coverage	1	-
Pneumococcal conjugate vaccine coverage*	1	-
Childhood stunted (<2SD)	1	+
Childhood underweight (<2SD)*	1	+
Childhood wasted (<2SD)*	1	+
Indoor air pollution*	1	+
LRI Summary Exposure Variable*	1	+
Breastfeeding	2	-
DTP3 vaccine coverage	2	-
Healthcare access and quality index	2	-
Outdoor air pollution (PM2.5)	2	+
Second-hand smoking prevalence	2	+
Vitamin A deficiency	2	+
Zinc deficiency	2	+
Handwashing	3	-
LDI per capita	3	-
Maternal education per capita	3	-
Socio-demographic index	3	-
Water and sanitation SEV	3	+

Over 5 years old model

Covariate	Level	Direction
Pneumococcal conjugate vaccine coverage	1	-
Indoor air pollution	1	+
LRI Summary Exposure Variable	1	+
Mean BMI	1	+
Smoking prevalence	1	+
DTP3 vaccine coverage	2	-
Healthcare access and quality index	2	-
Education per capita	3	-
LDI per capita	3	-
Socio-demographic index	3	-
Alcohol liters per capita	3	+
Outdoor air pollution (PM2.5)	3	+
Water and sanitation SEV	3	+

Summary of LRI morbidity modelling

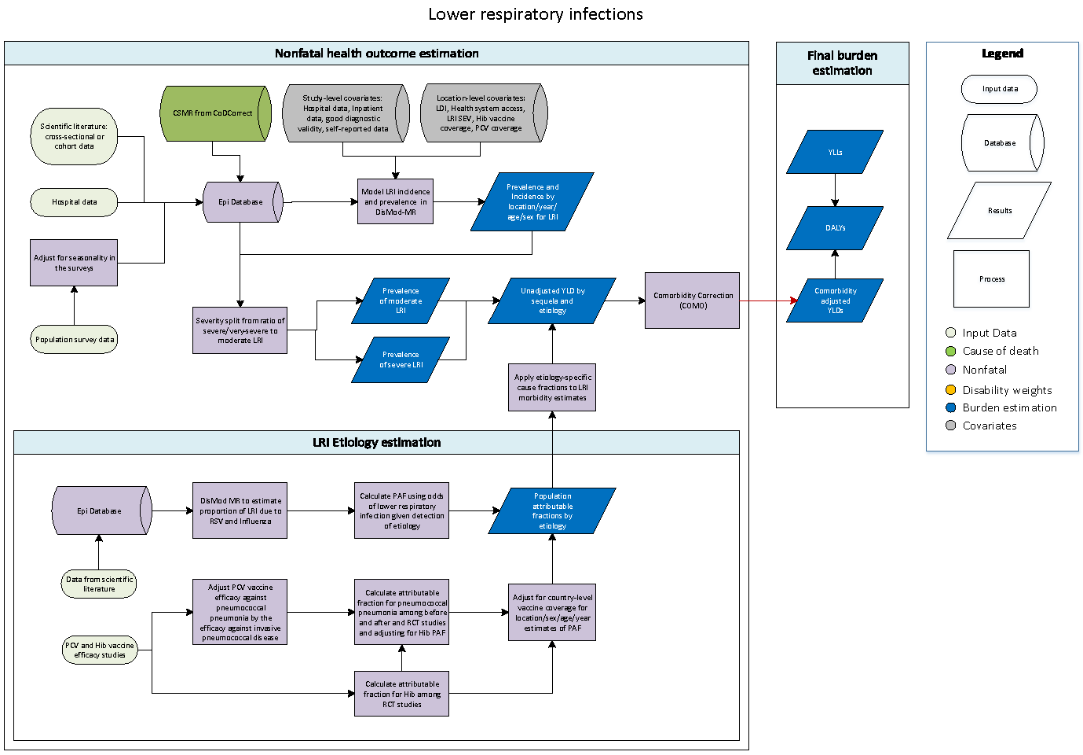

Appendix Flowchart 2. Non-fatal LRI modelling

This flowchart shows the detailed analytic strategy for the non-fatal LRI modelling including the aetiologic attribution to LRI morbidity. Each of these steps will be discussed in greater detail in the section that follows.

Lower respiratory infections (LRI) are characterised as clinician-diagnosed or x-ray confirmed pneumonia or bronchiolitis. Cases are split into moderate and severe/very severe episodes using a severity definition that closely matches the WHO Integrated Management of Childhood Illness categories of pneumonia (**Appendix Table 3**).[6]

The distribution of moderate (84·9%, 95% UI: 83·1-86·7%) and severe/very severe (15·1%, 95% UI: 13·3-16·9%) LRI is determined by a random effects meta-analysis of the ratio of severe to all LRI from 15 studies that report the incidence of moderate and severe LRI. Years lived with disability (YLDs) are calculated using the severity distribution of LRI cases and the disability weights associated with them. The disability weights were separately estimated by the Disability Weights Survey portion of the GBD study and were systematically constructed based on responses from more than 6,000 survey respondents.[7]

Appendix Table 3. Severity definitions

Severity level	Lay description	Disability Weight (95% CI)	Percent of episodes (95% CI)
Moderate	Cough or difficulty breathing with rapid breathing<br>Has a fever and aches and feels weak which causes some difficulty with daily activities	0·051 (0·032-0·074)	84·9% (83·1-86·7%)
Severe/very severe	Cough or difficulty breathing with lower chest wall indrawing, central cyanosis, or the inability to drink<br>Has a high fever and pain and feels very weak, which causes great difficulty with daily activities	0·133 (0·088-0·19)	15·1% (13·3-16·9%)

The non-fatal LRI burden, including incidence and prevalence, is modelled in DisMod-MR 2·1 (DisMod). DisMod is a Bayesian, hierarchical, age-integrating meta-regression tool that relates incidence, prevalence, recovery, and mortality. Input data are from a systematic literature review of cross-sectional and cohort studies, hospital inpatient and outpatient data (ICD9 Codes included 073·0-073·6, 079·82, 466-469, 480-489, 513·0, and 770·0. ICD10 codes included A48·1, J09-J22, J85·1, P23-P23·9, and U04), MarketScan healthcare utilization data (USA only), and population-representative surveys. To make the data more consistent for covariates and adjustments in the modelling process, we converted all incidence data to prevalence data using an average duration of illness of 7.8 days. Input data include all data used in GBD 2015 and a new review of data sources from January 2016-May 2017 (**Appendix Table 4, Appendix Figure 2**).

Appendix Table 4. The number of data points by source type used in GBD 2016

Type of data	Data points (#)
Facility - inpatient	15,258
Facility - other/unknown	6,411
Survey - cross-sectional	7,772
Other	909
**Total**	**30,350**

Appendix Figure 2. Geographic distribution of LRI morbidity modelling

The number of source-years by country is shown for A) All data, B) Survey data, C) Hospital data. Overall, there are 30,350 data points from 718 unique sources. Input data and models can be found using the GBD visualization tools at: http://vizhub.healthdata.org/data-visualizations.

A) All Data

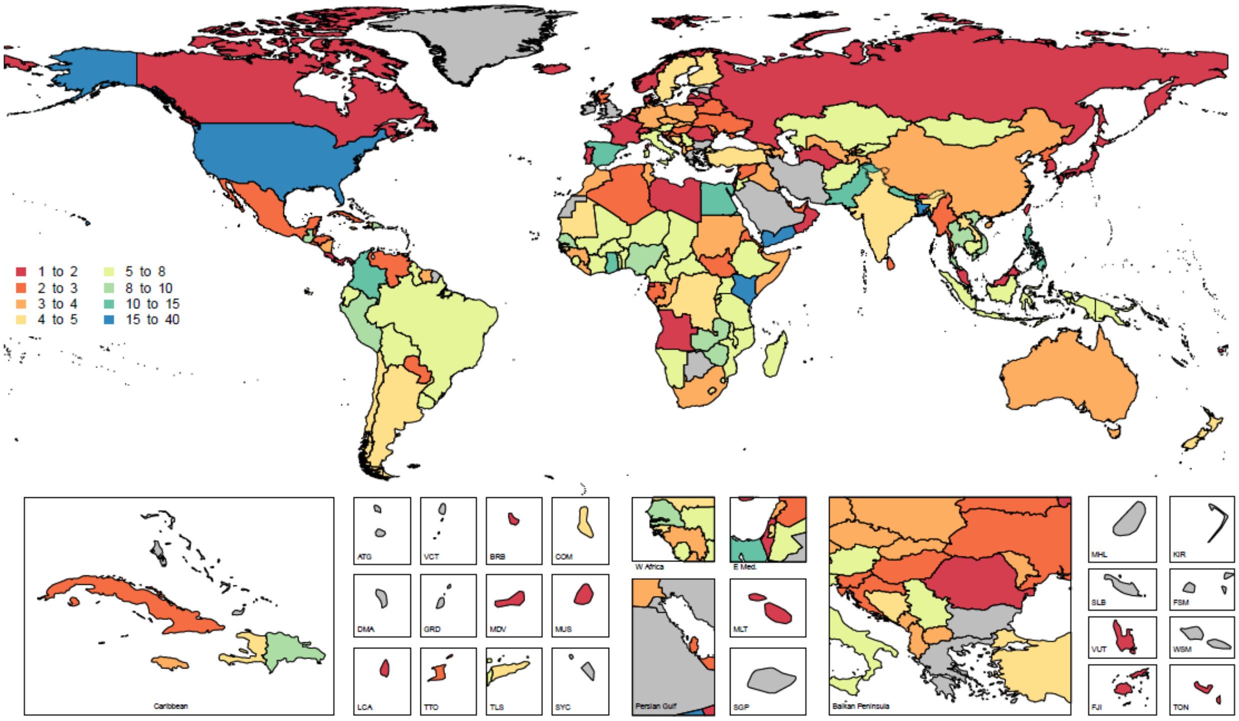

B) Survey data

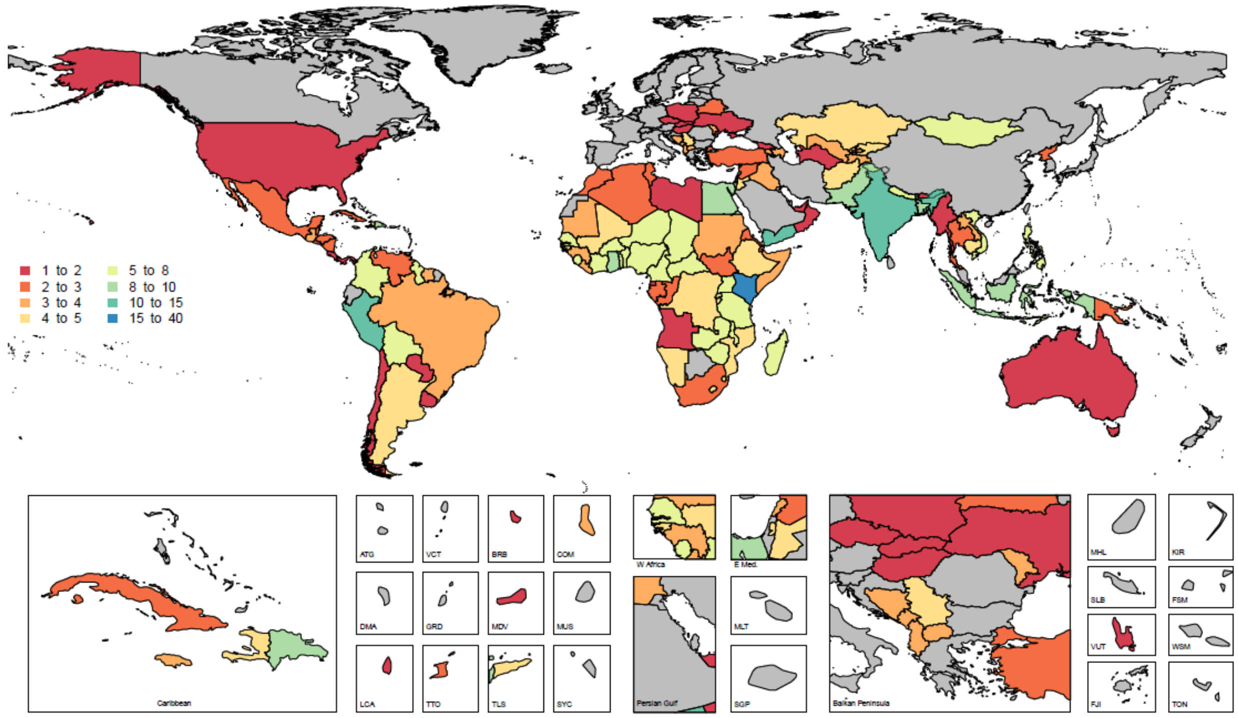

C) Hospital data

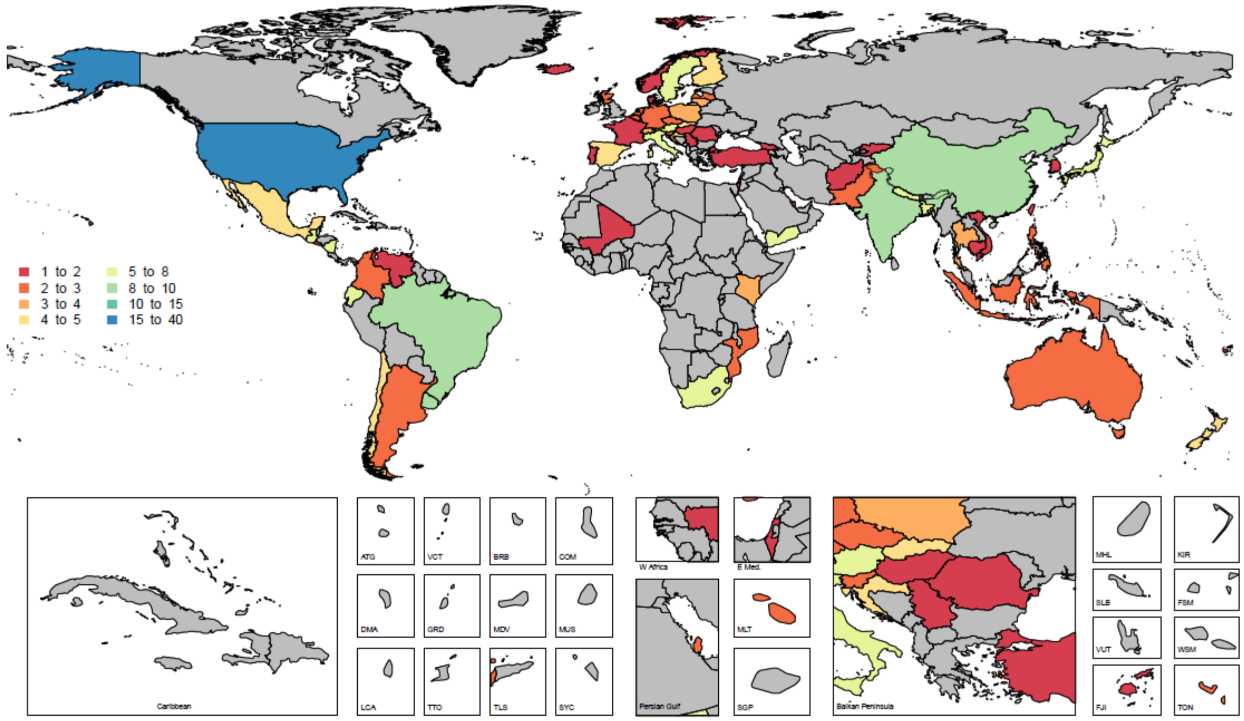

Input data are adjusted to our standard case definition. Data are adjusted by study-level binary covariates which describe if the source is a hospital or inpatient sample and if the data come from a self-reported survey (**Appendix Table 5**). Self-reported prevalence of LRI symptoms from population-representative surveys such as the Demographic and Health Survey (DHS) and the Multiple Indicator Cluster Survey (MICS) is used. Our case definition from symptom-based prevalence estimates is children in the last two weeks with fever and cough with difficulty breathing and symptoms located in the chest and/or chest and nose. This is consistent with the WHO Integrated Management of Childhood Illness guidelines definition of pneumonia and with the DHS and MICS definition of acute respiratory infection (ARI).[6] We extracted the prevalence of children under 5 years old that had fever and cough with difficulty breathing and included an indicator for this less-specific definition.

Some surveys did not include the prevalence of LRI symptoms *and* fever so we adjusted survey prevalence estimates that did not include fever by studies that did based on a logistic regression. These data were converted from two-week period prevalence to point prevalence using a mean duration of illness of 7.8 days. The illness duration was based on a review of available data on LRI symptom duration including cough, difficulty breathing, and fever. Where applicable, period prevalence was converted to point prevalence using the following formula:

$$Point\ Prevalence = \frac{Period\ Prevalence\ *\ Duration}{(Recall\ Period + Duration - 1)}$$

Appendix Table 5. Case definitions and adjustments for LRI

A summary of the acceptable case definitions from self-reported, population-representative surveys for symptoms of lower respiratory infections and the data adjustments that are performed to make these responses comparable to our case definition of physician-diagnosed pneumonia or bronchiolitis.

Definition	Adjustment for missing fever+	Adjustment for missing chest symptoms	Adjustment for self-reported data	Overall adjustment
Fever with cough and difficulty breathing & symptoms in chest	None	None	0.25 (0.23-0.26)	0.25*
Fever with cough and difficulty breathing	None	0.58 (0.55-0.63)	0.25 (0.23-0.26)	0.145^
Cough and difficulty breathing & symptoms in chest	0.8	None	0.25 (0.23-0.26)	0.2
Cough and difficulty breathing	0.58	0.58 (0.55-0.63)	0.25 (0.23-0.26)	0.084

*Hazir et al. 2013 found that the accuracy of DHS suspected pneumonia + fever was between 5.1 and 33.9% for clinician-diagnosed pneumonia in Bangladesh and Pakistan and the specificity was 63.8-85.7%
+Rambaud-Althaus et al. 2015 found that fever was present in 94% of pneumonia episodes
^Rambaud-Althaus et al. 2015 found that accuracy of difficulty breathing was 12%

As surveys are frequently conducted over a period of less than a year, these data are potentially biased by the seasonality of respiratory infections. To account for this variation, we implemented an adjustment factor for survey data where a sine-cosine regression with a period of 6 months was fit to each GBD region, weighted by the standard error of the input data. The percent difference in the periodic regression fit and the mean respiratory infection symptom prevalence in each region was used as a scalar to adjust the survey prevalence input data based on the month of the survey for each individual (**Appendix Figures 3 and 4**). After applying this scalar, the mean LRI 2-week prevalence from each survey by 1-year age group and by child sex was extracted and prepared for use in the LRI non-fatal modelling.

Appendix Figure 3. LRI seasonality adjustment

This plot illustrates the seasonal adjustment performed for LRI survey data to account for seasonal biases. The relative difference between the fitted sinusoidal curve and the horizontal dashed line is the monthly scalar. This regression is performed for each GBD region and adjusted for survey-year and the case definition in the survey.

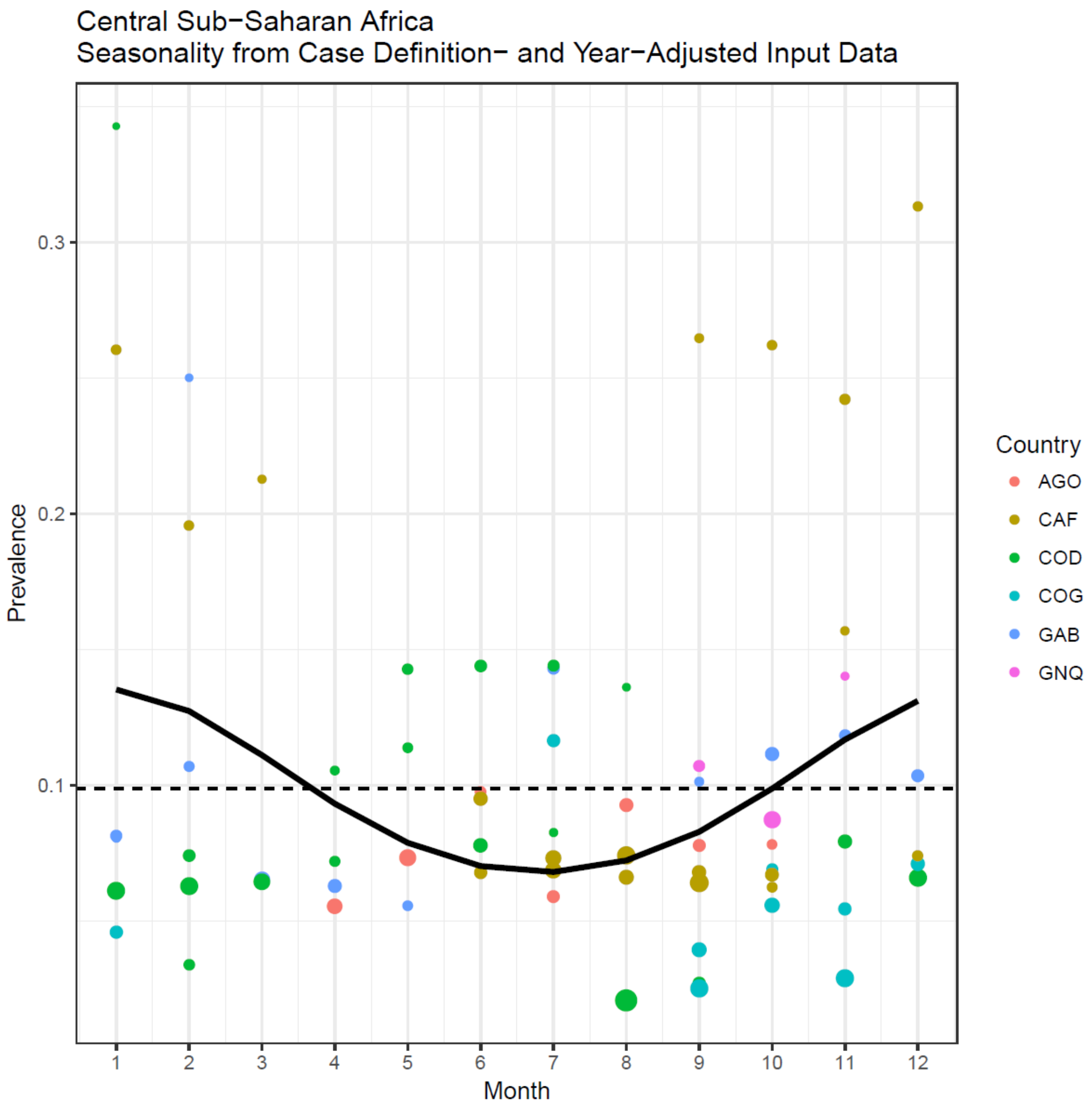

Appendix Figure 4. The seasonality sinusoidal regression fit is shown for each GBD super-region

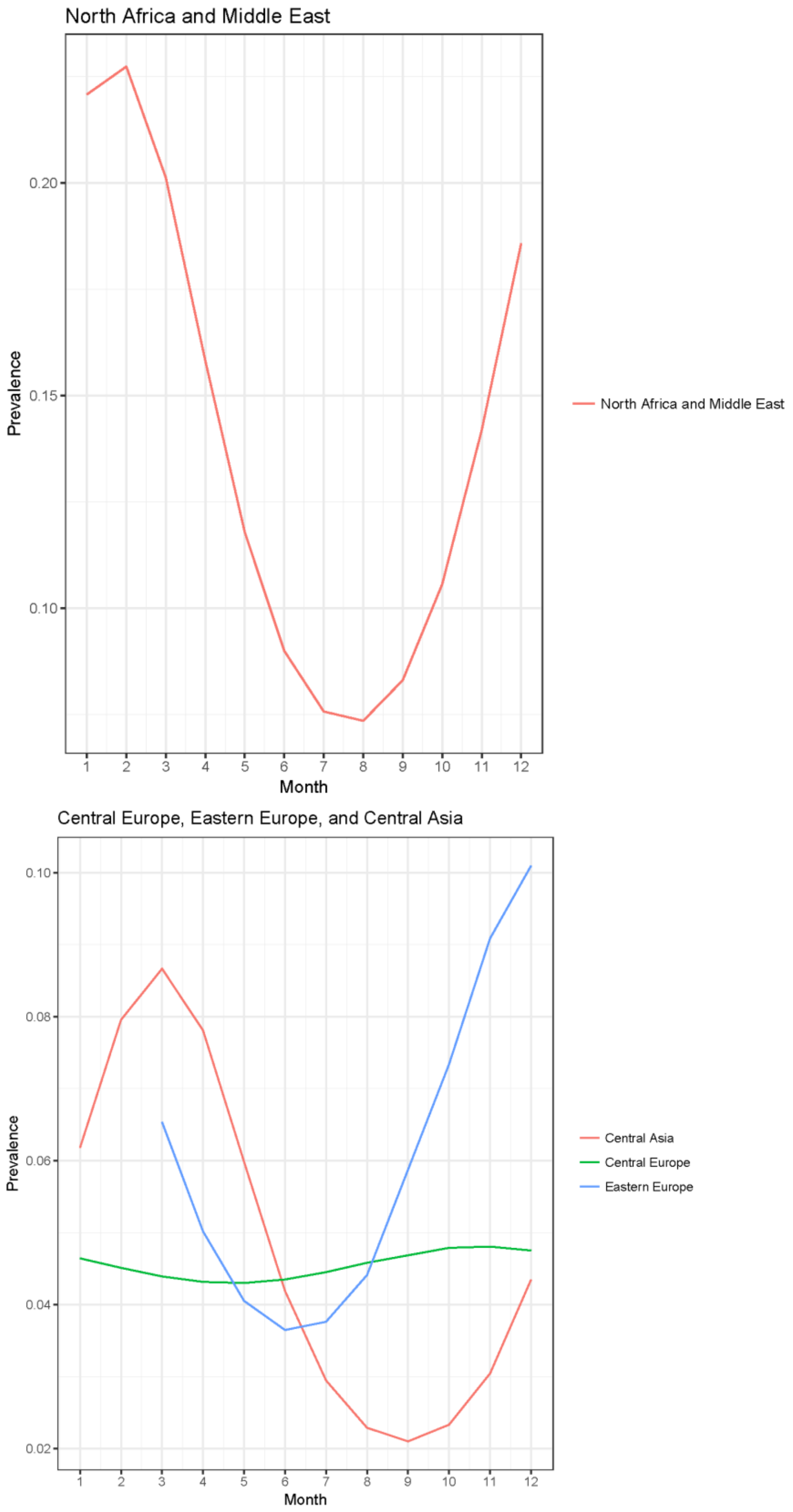

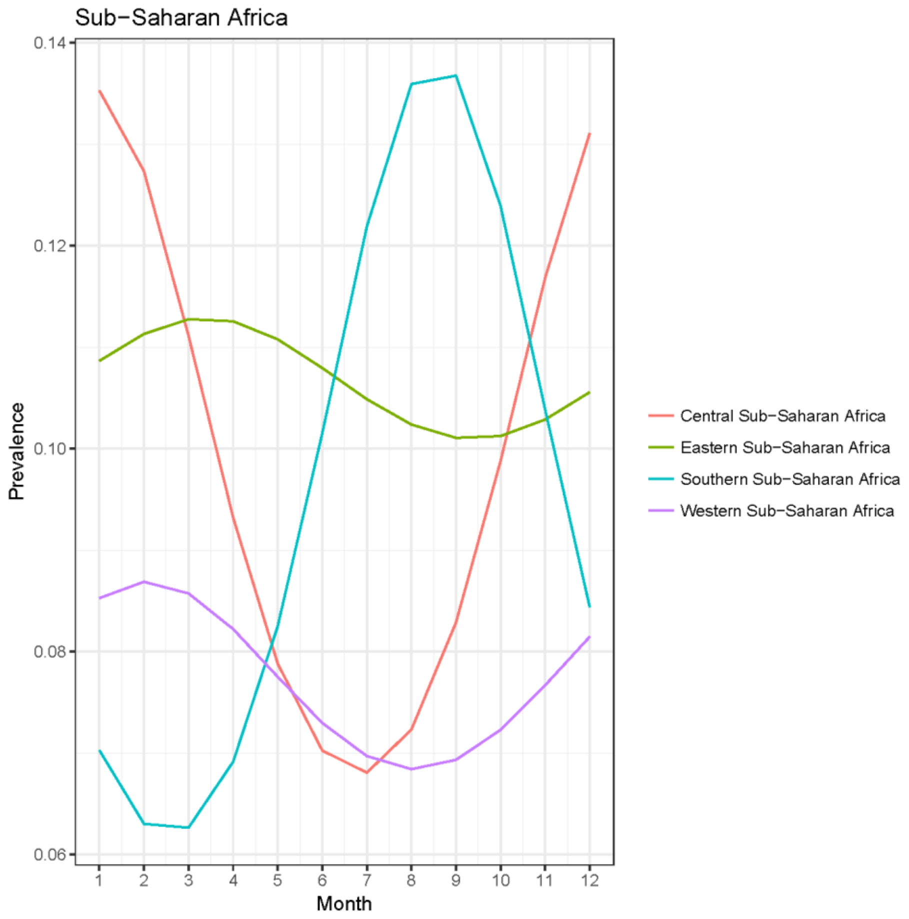
Sub−Saharan Africa
Prevalence
Month
0.14
0.12
0.10
0.08
0.06
1 2 3 4 5 6 7 8 9 10 11 12
Central Sub−Saharan Africa
Eastern Sub−Saharan Africa
Southern Sub−Saharan Africa
Western Sub−Saharan Africa

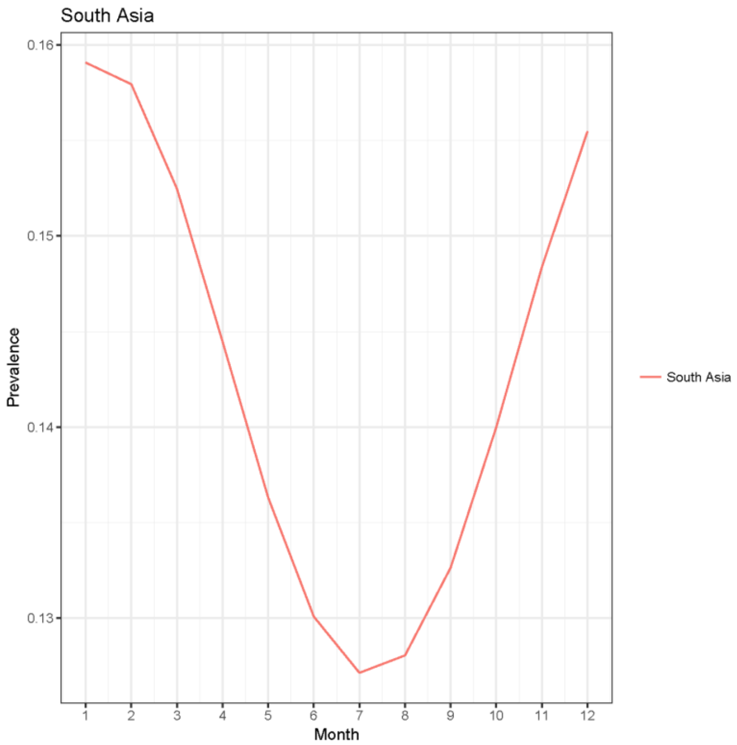
South Asia
Prevalence
Month
0.16
0.15
0.14
0.13
1 2 3 4 5 6 7 8 9 10 11 12
South Asia

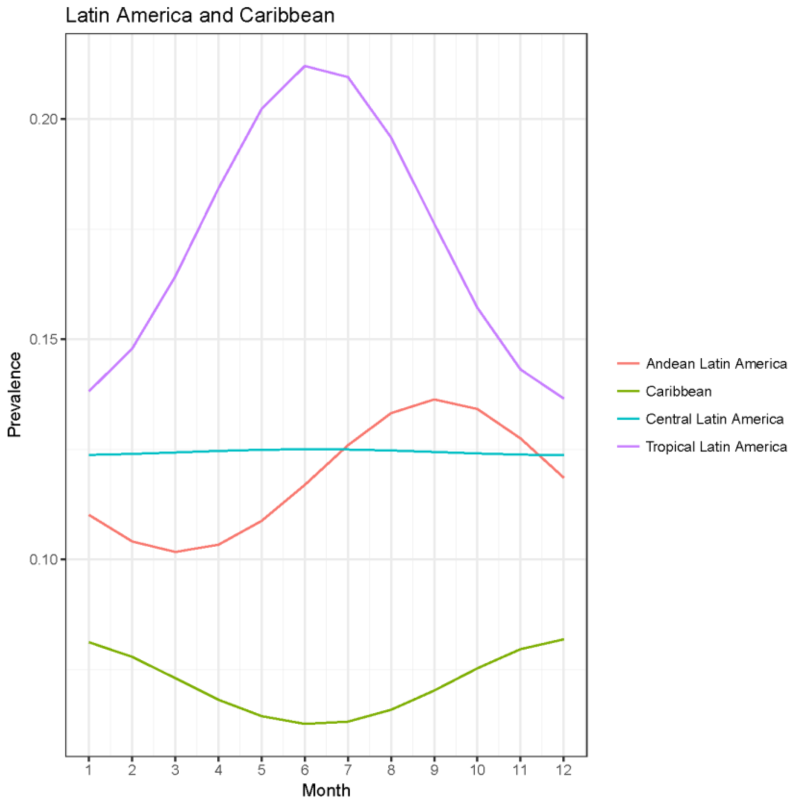
Latin America and Caribbean
Prevalence
0.20
0.15
0.10
1
2
3
4
5
6
7
8
9
10
11
12
Month
Andean Latin America
Caribbean
Central Latin America
Tropical Latin America

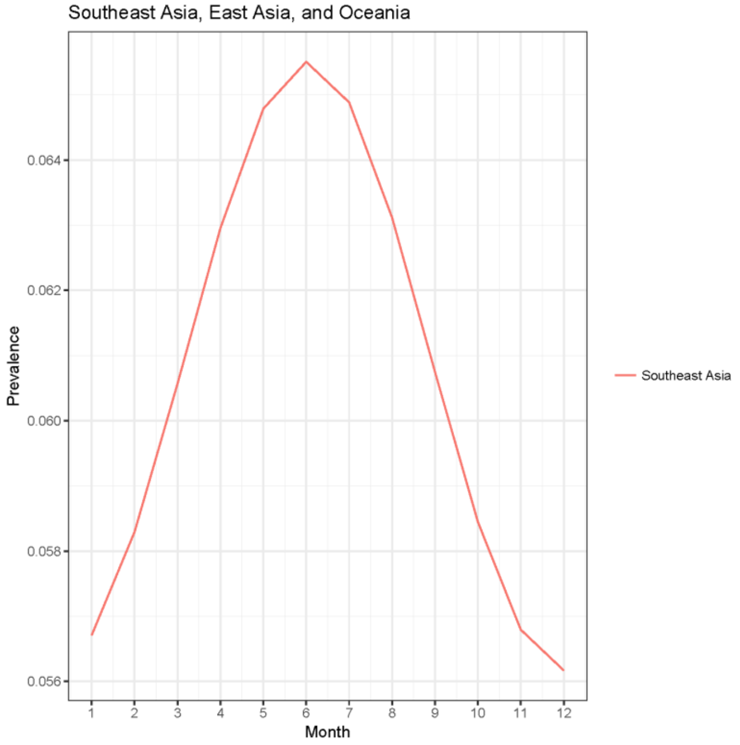
Southeast Asia, East Asia, and Oceania
Prevalence
0.064
0.062
0.060
0.058
0.056
1
2
3
4
5
6
7
8
9
10
11
12
Month
Southeast Asia

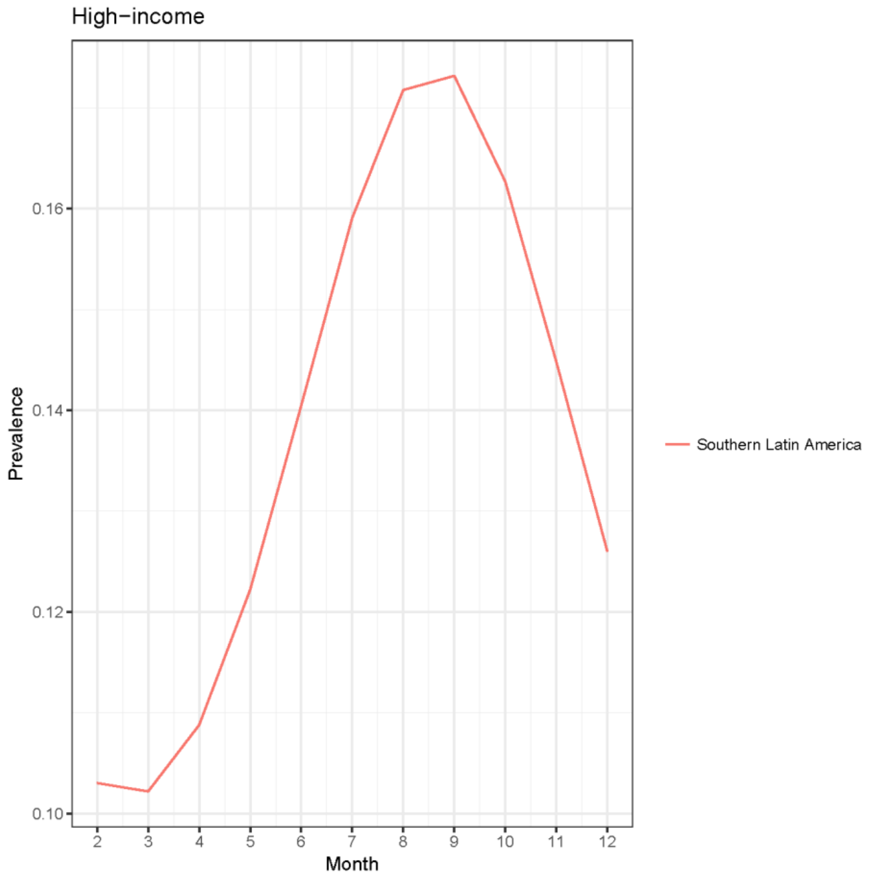
High−income
Prevalence
0.16
0.14
0.12
0.10
2
3
4
5
6
7
8
9
10
11
12
Month
Southern Latin America

Country-level covariates also inform the model. These include Hib and pneumococcal conjugate vaccine coverage, health system access, income per capita, and the SEV for LRI (**Appendix Table 6**). In addition, the model is informed by cause-specific mortality estimates from a different process for mortality and cause of death in GBD 2016. PCV and Hib vaccine coverage are modelled as covariates for GBD 2016. The estimates of the vaccine coverage are modelled using spatio-temporal Gaussian Process Regression, a Bayesian modelling approach to leverage space-time variation, using input data from surveys, delivery campaigns, and information about national vaccination infrastructure.[8]

Appendix Table 6. Covariates in the LRI DisMod model

Study covariate	Parameter	Exponentiated beta (95% UI)
Hospital inpatient population	Prevalence	0.25 (0.23-0.27)
Hospital data from middle- or low-income country	Prevalence	0.17 (0.16-0.19)
Self-reported	Prevalence	4.05 (3.81-4.27)
Poor diagnostic specificity	Prevalence	1.72 (1.63-1.81)
Hib vaccine coverage	Prevalence	0·73 (0.70-0.76)
Socio-demographic Index	Prevalence	0.77 (0.59-1.06)
LRI SEV	Prevalence	0.98 (0.91-1.07)
Healthcare access and quality index	Excess mortality	0·96 (0.96-0.96)

Summary of aetiology population attributable fraction strategy

We estimated LRI aetiologies separately from overall LRI mortality using two distinct counterfactual modelling strategies based on population attributable fractions (PAFs), described in detail below. We did not attribute aetiologies to neonatal pneumonia due to a dearth of reliable data in this age group. We calculated uncertainty of our PAF estimates from 1,000 draws of each parameter using normal distributions in log space.

Pneumococcal pneumonia and Hib

For *Streptococcus pneumoniae* (pneumococcal pneumonia) and *Haemophilus influenzae* type B (Hib), we calculated the population attributable fraction using a vaccine probe design.[9,10] The ratio of vaccine effectiveness against nonspecific pneumonia to pathogen-specific disease represents the fraction of pneumonia cases attributable to each pathogen.

To estimate the PAF for Hib and pneumococcal pneumonia, we calculated the ratio of vaccine effectiveness against nonspecific pneumonia to pathogen-specific pneumonia (Equations 1 and 3). We estimated a study-level estimate of PAF from a meta-analysis of these ratios (sources provided in **Appendix Table 7**). To estimate the PAF for Hib, we only used randomised controlled trials because of implausibly high values of vaccine efficacy in case-control studies. To estimate the PAF for pneumococcal pneumonia, we included RCTs and before and after vaccine introduction longitudinal studies.

We adjusted the study-level PAF estimate by vaccine coverage and expected vaccine performance to estimate country- and year-specific PAF values. For pneumococcal pneumonia, we adjusted the PAF by the final Hib PAF estimate and by vaccine serotype coverage.[11] Finally, we used an age distribution of PAF modelled in DisMod to determine the PAF by age (**Appendix Figure 5**). Because of an absence of data describing vaccine efficacy against Hib in children older than 2 years of age, we did not attribute Hib to episodes of LRI in ages 5 years and older.

Appendix Table 7. Sources in the Hib and Streptococcus pneumoniae vaccine efficacy meta-analysis

Citation	Year	Location
**Pneumococcal pneumonia**		
Ansaldi F, Sticchi L, Durando P, Carloni R, Oreste P, Vercelli M, Crovari P, Icardi G. Decline in pneumonia and acute otitis media after the introduction of childhood pneumococcal vaccination in Liguria, Italy. *J Int Med Res*. 2008; 36(6): 1255-60.	2008	Italy
Bonten MJM, Huijts SM, Bolkenbaas M, Webber C, Patterson S, Gault S, van Werkhoven CH, van Deursen AMM, Sanders EAM, Verheij TJM, Patton M, McDonough A, Moradoghli-Haftvani A, Smith H, Mellelieu T, Pride MW, Crowther G, Schmoele-Thoma B, Scott DA, Jansen KU, Lobatto R, Oosterman B, Visser N, Caspers E, Smorenburg A, Emini EA, Gruber WC, Grobbee DE. Polysaccharide conjugate vaccine against pneumococcal pneumonia in adults. *N Engl J Med*. 2015; 372(12): 1114–25	2015	Netherlands
Cutts F, Zaman SM, Enwere G, Jaffar S, Levine O, Okoko J, Oluwalana C, Vaughan A, Obaro S, Leach A, McAdam K, Biney E, Saaka M, Onwuchekwa U, Yallop F, Pierce N, Greenwood B, Adegbola R. Efficacy of nine-valent pneumococcal conjugate vaccine against pneumonia and invasive pneumococcal disease in The Gambia: randomised, double-blind, placebo-controlled trial. *Lancet*. 2005; 365(9465): 1139-46	2005	The Gambia
Grijalva CG, Nuorti JP, Arbogast PG, Martin SW, Edwards KM, Griffin MR. Decline in pneumonia admissions after routine childhood immunisation with pneumococcal conjugate vaccine in the USA: a time-series analysis. *Lancet*. 2007; 369(9568): 1179-86	2007	United States
Hansen J, Black S, Shinefield H, Cherian T, Benson J, Fireman B, Lewis E, Ray P, Lee J. Effectiveness of Heptavalent Pneumococcal Conjugate Vaccine in Children Younger Than 5 Years of Age for Prevention of Pneumonia: Updated Analysis Using World Health Organization Standardized Interpretation of Chest Radiographs. *Pediatr Infect Dis J*. 2006; 25(9): 779-81	2006	United States
Jardine A, Menzies RI, McIntyre PB. Reduction in hospitalizations for pneumonia associated with the introduction of a pneumococcal conjugate vaccination schedule without a booster dose in Australia. *Pediatr Infect Dis J*. 2010; 29(7): 607-12.	2010	Australia
Klugman KP, Madhi SA, Huebner RE, Kohberger R, Mbelle N, Pierce N, Vaccine Trialists Group. A trial of a 9-valent pneumococcal conjugate vaccine in children with and those without HIV infection. *N Engl J Med*. 2003; 349(14): 1341-8	2003	South Africa
Lucero MG, Tallo V, Lupisan S, Sanvictores D, Ugpo J, Lechago M, Abucejo-Ladesma E, Sombrero L, Nohynek H, Puumalainen T, Nissinen A, Soininen A, Ruutu P, Makela HP, Williams G, Forsyth S, De Campo M, Riley I, Simoes EAF. Efficacy of an 11-valent pneumococcal conjugate vaccine against radiologically confirmed pneumonia among children less than 2 years of age in the Philippines: A randomized, double-blind, placebo-controlled trial. *Pediatr Infect Dis J*. 2009; 28(6): 455-62	2009	Philippines
Martinelli D, Pedalino B, Cappelli MG, Caputi G, Sallustio A, Fortunato F, Tafuri S, Cozza V, Germinario C, Chironna M, Prato R. Towards the 13-valent pneumococcal conjugate universal vaccination: effectiveness in the transition era between PCV7 and PCV13 in Italy, 2010-2013. *Hum Vaccin Immunother*. 2014; 10(1): 33-9	2014	Italy
Maruyama T, Taguchi O, Niederman MS, Morser J, Kobayashi H, Kobayashi T, D'Alessandro-Gabazza C, Nakayama S, Nishikubo K, Noguchi T, Takei Y, Gabazza EC. Efficacy of 23-valent pneumococcal vaccine in preventing pneumonia and improving survival in nursing home residents: double blind, randomised and placebo controlled trial. BMJ. 2010; 340: c1004.	2010	Japan
O'Brien KL, Millar EV, Zell ER, Bronsdon M, Weatherholtz R, Reid R, Becenti J, Kvamme S, Whitney CG, Santosham M. Effect of pneumococcal conjugate vaccine on nasopharyngeal colonization among immunized and unimmunized children in a community-randomized trial. J Infect Dis. 2007; 196(8): 1211-20	2007	United States
Simonsen L, Taylor RJ, Young-Xu Y, Haber M, May L, Klugman KP. Impact of Pneumococcal Conjugate Vaccination of Infants on Pneumonia and Influenza Hospitalization and Mortality in All Age Groups in the United States. *MBio*. 2011; 2(1): e00309-10	2011	United States
Tregnaghi MW, Sáez-Llorens X, López P, Abate H, Smith E, Pósleman A, et al. Evaluating the efficacy of 10-valent pneumococcal non-typeable Haemophilus influenzae protein-D conjugate vaccine (PHiD-CV) against community-acquired pneumonia in Latin America [abstract]. In: Abstracts of the 29th Annual Meeting of the European Society for Paediatric Infectious Diseases (ESPID); 2011 June 7-11; The Hague, The Netherlands	2011	Panama
Zhou F, Kyaw MH, Shefer A, Winston CA, Nuorti J. Health care utilization for pneumonia in young children after routine pneumococcal conjugate vaccine use in the United States. *Arch Pediatr Adolesc Med*. 2007; 161(12): 1162-8	2007	United States

**Hib**		
Baqui AH, El Arifeen S, Saha SK, Persson L, Zaman K, Gessner BD, Moulton LH, Black RE, Santosham M. Effectiveness of Haemophilus influenzae type B conjugate vaccine on prevention of pneumonia and meningitis in Bangladeshi children: a case-control study. *Pediatr Infect Dis J*. 2007; 26(7): 565-71	2007	Bangladesh
Gessner BD, Sutanto A, Linehan M, Djelantik IG, Fletcher T, Gerudug IK, Ingerani, Mercer D, Moniaga V, Moulton LH, Moulton LH, Mulholland K, Nelson C, Soemohardjo S, Steinhoff M, Widjaya A, Stoeckel P, Maynard J, Arjoso S. Incidences of vaccine-preventable Haemophilus influenzae type b pneumonia and meningitis in Indonesian children: hamlet-randomised vaccine-probe trial. *Lancet*. 2005; 365(9453): 43-52	2005	Indonesia
Levine OS, Lagos R, Muñoz A, Villaroel J, Alvarez AM, Abrego P, Levine MM. Defining the burden of pneumonia in children preventable by vaccination against Haemophilus influenzae type b. *Pediatr Infect Dis J*. 1999; 18(12): 1060-4	1999	Chile
Mulholland K, Hilton S, Adegbola R, Usen S, Oparaugo A, Omosigho C, Weber M, Palmer A, Schneider G, Jobe K, Lahai G, Jaffar S, Secka O, Lin K, Ethevenaux C, Greenwood B. Randomised trial of Haemophilus influenzae type-b tetanus protein conjugate vaccine corrected for prevention of pneumonia and meningitis in Gambian infants. *Lancet*. 1997; 349(9060): 1191-7	1997	The Gambia

Appendix Figure 5. Age distribution of the pneumococcal pneumonia base population attributable fraction

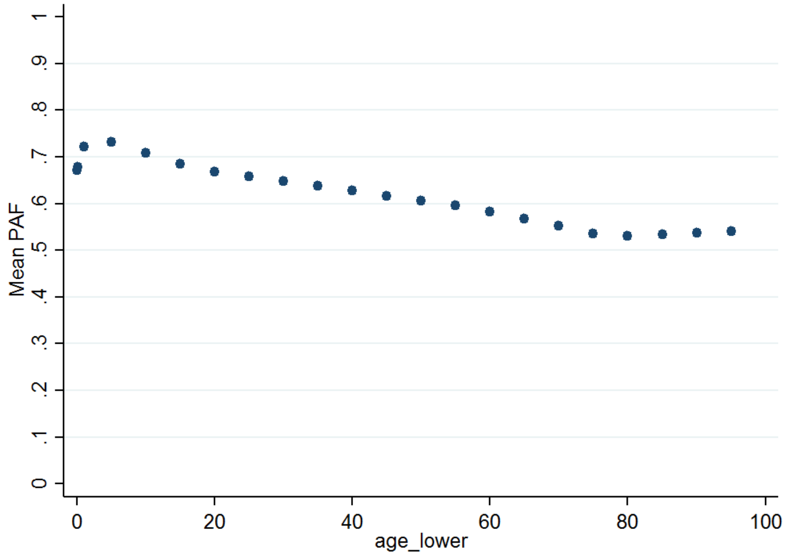

We used a vaccine probe design to estimate the PAF for pneumococcal pneumonia and (Hib) by first calculating the ratio of vaccine effectiveness against nonspecific pneumonia to pathogen-specific pneumonia at the study level (Equations 1 and 2).[9,10,12] We then adjusted this estimate by vaccine coverage and expected vaccine performance to estimate country- and year-specific PAF values (Equations 3 and 4).

$$1)\quad HibPAF_{Base} = \frac{VE_{Pneumonia}}{VE_{Hib}}$$

$$2)\quad PneumoPAF_{Base} = \frac{VE_{Pneumonia} * (1 - PAF_{Hib} * VE_{Hib\ Optimal})}{VE_{Streptococcus} * Cov_{Serotype}}$$

$$3)\quad PAF_{Hib} = PAF_{Base} * \frac{(1 - Cov_{Hib} * VE_{Hib\ Optimal})}{(1 - PAF_{Base} * Cov_{Hib} * VE_{Hib\ Optimal})}$$

$$4)\quad PAF_{Pneumo} = \frac{PAF_{Base} * (1 - Cov_{PCV} * VE_{PCV\ Optimal})}{(1 - PAF_{Hib} * Cov_{Hib} * VE_{Hib\ Optimal}) * \left(1 - \frac{PAF_{Base} Cov_{PCV} * VE_{PCV\ Optimal}}{(1 - PAF_{Hib} * Cov_{Hib} * VE_{Hib\ Optimal})}\right)}$$

Where $VE_{Pneumonia}$ is the vaccine efficacy against nonspecific pneumonia, VE_{Hib} is the vaccine efficacy against invasive Hib disease, $VE_{Streptococcus}$ is the vaccine efficacy against serotype-specific pneumococcal pneumonia, $Cov_{serotype}$ is the serotype-specific vaccine coverage for PCV,[11] $VE_{Hib\ Optimal}$ is the Hib effectiveness in the community (0·8)[13], PAF_{Hib} is the final PAF for Hib, Cov_{PCV} is the PCV coverage, Cov_{Hib} is the Hib coverage by country, and $VE_{PCV\ Optimal}$ is the vaccine

effectiveness in the community (0·8).[14] **Appendix Figure 6** shows the global maps for PCV and Hib vaccine coverage estimates among all ages in 2005 and 2016.

For Hib, we assumed that the vaccine efficacy against invasive Hib disease is the same against Hib pneumonia. For pneumococcal pneumonia, a recent study in adults [15] found that the vaccine efficacy against invasive pneumococcal disease may be significantly higher than against pneumococcal pneumonia. We used this ratio to adjust estimates of vaccine efficacy against invasive pneumococcal disease from other studies. However, recognising that the study is unique in that it uses a urine antigen test among adults, we added uncertainty around our adjustment using a wide uniform distribution (median 0·65, 0·3-1·0). This has increased the estimates of pneumococcal pneumonia mortality in a meaningful way.

Appendix Figure 6. Vaccine coverage estimates for all ages

Vaccine coverage estimates are modelled as part of the covariate models for GBD 2016. **A)** Hib coverage in 2005, **B)** Hib coverage in 2016, **C)** PCV coverage in 2005, & **D)** PCV coverage in 2016.

380**A)**

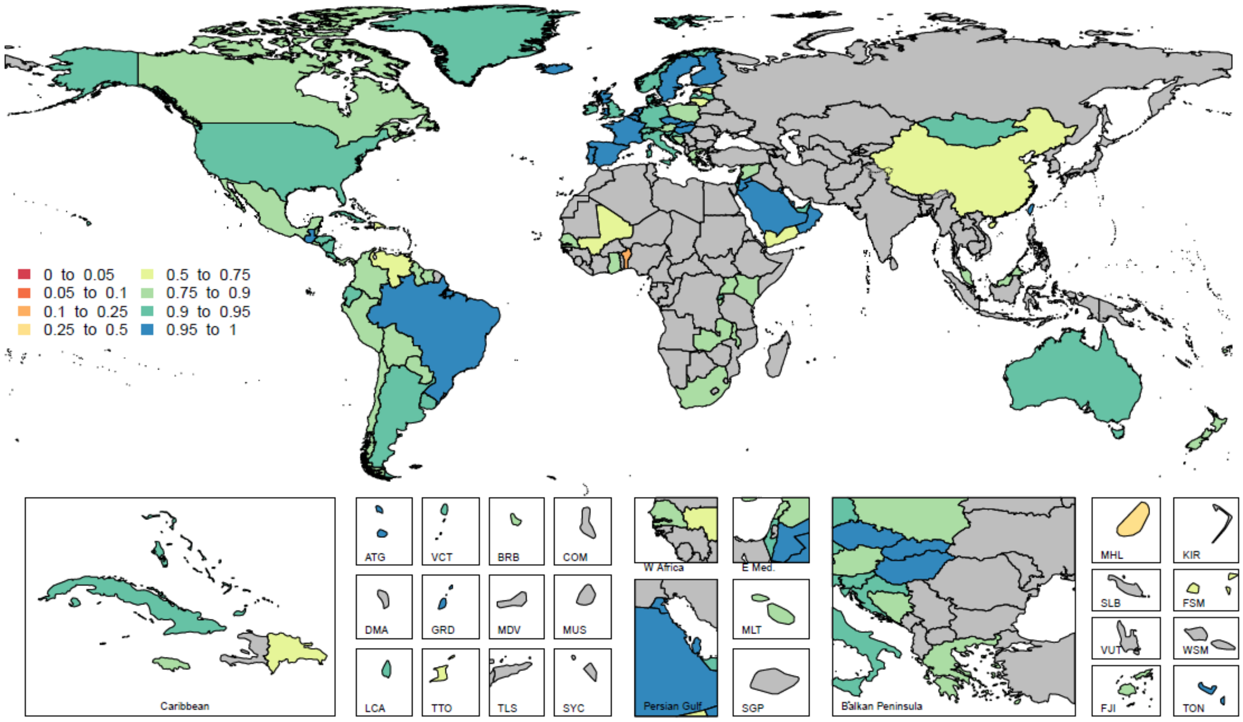

B)

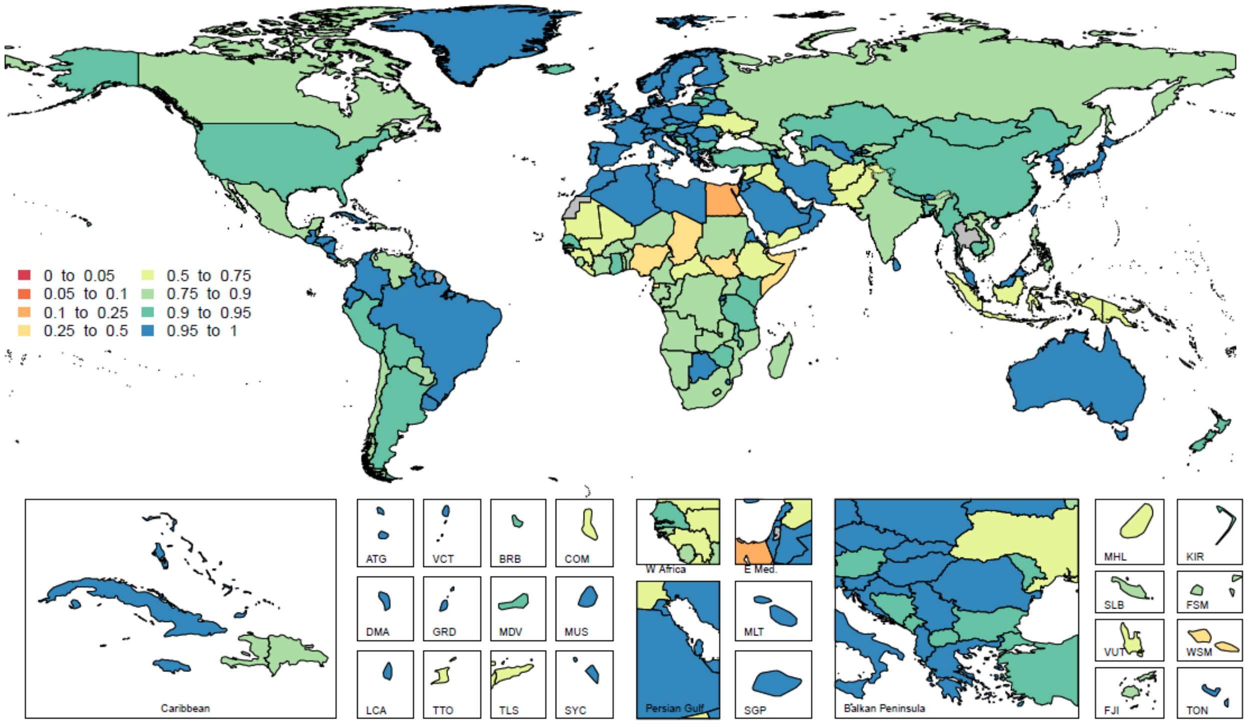

C)

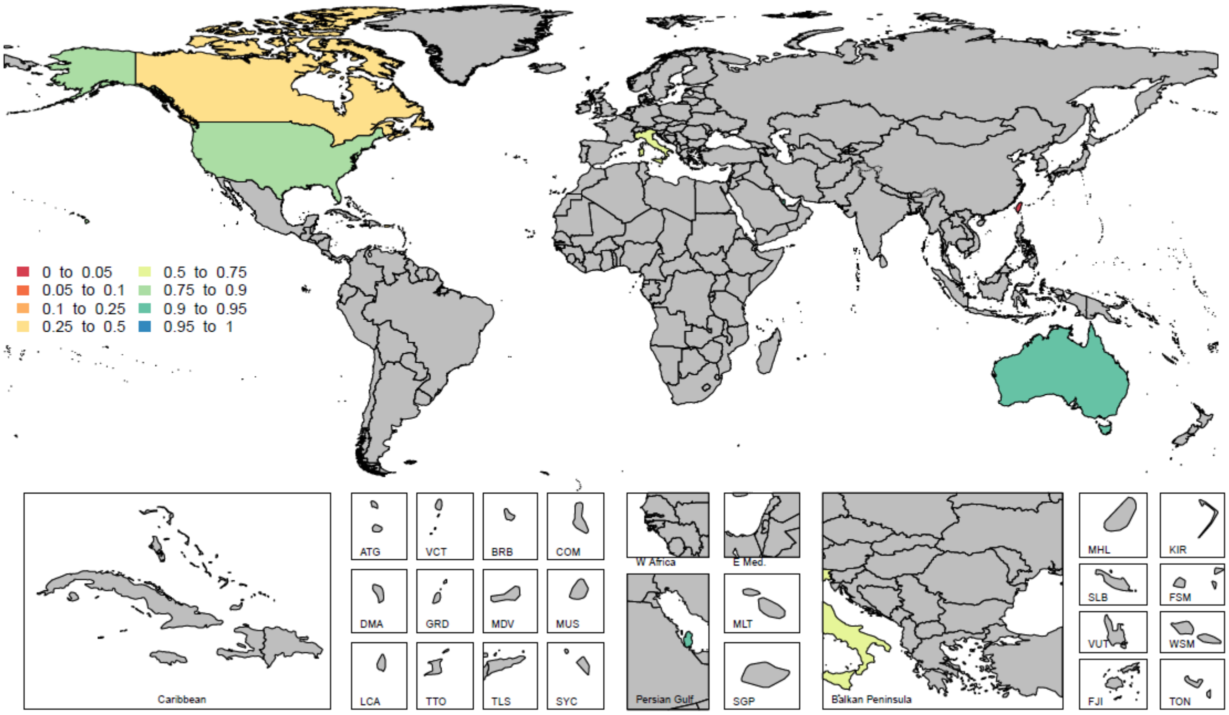

0 to 0.05
0.05 to 0.1
0.1 to 0.25
0.25 to 0.5
0.5 to 0.75
0.75 to 0.9
0.9 to 0.95
0.95 to 1
Caribbean
ATG
VCT
BRB
COM
DMA
GRD
MDV
MUS
LCA
TTO
TLS
SYC
W Africa
E Med.
Persian Gulf
MLT
SGP
Balkan Peninsula
MHL
KIR
SLB
FSM
VUT
WSM
FJI
TON

D)

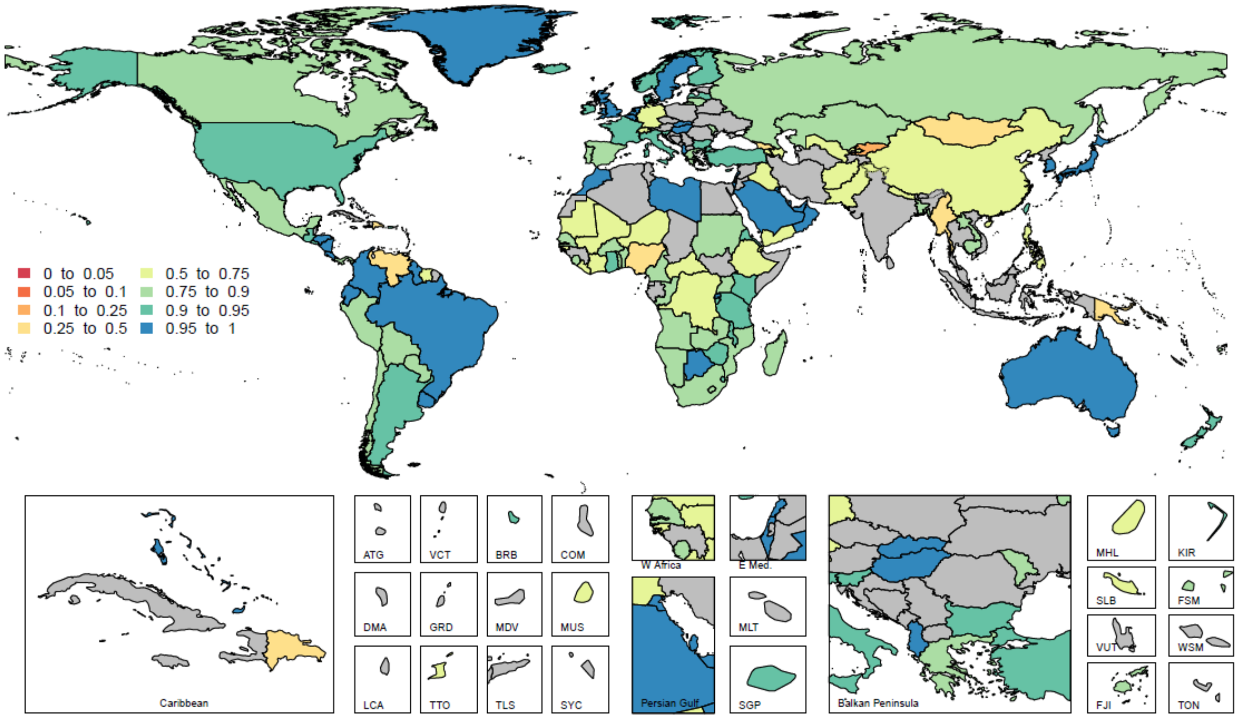

0 to 0.05
0.05 to 0.1
0.1 to 0.25
0.25 to 0.5
0.5 to 0.75
0.75 to 0.9
0.9 to 0.95
0.95 to 1
Caribbean
ATG
VCT
BRB
COM
DMA
GRD
MDV
MUS
LCA
TTO
TLS
SYC
W Africa
E Med.
Persian Gulf
MLT
SGP
Balkan Peninsula
MHL
KIR
SLB
FSM
VUT
WSM
FJI
TON

Influenza and RSV

The population attributable fractions (PAFs) for influenza and respiratory syncytial virus (RSV) are estimated using the following formula:[16]

$$5)\quad PAF = Proportion * (1 - \frac{1}{OR})$$

Where *Proportion* is the proportion of LRI cases that test positive for influenza or RSV and *OR* is the odds ratio of LRI given the presence of the pathogen. We used an odds ratio of 5·1 (3·19 – 8·14) for influenza and 9·79 (4·98 – 19·27) for RSV from a recently published meta-analysis.[17]

The *Proportion* values are modelled estimates. We used the meta-regression tool DisMod-MR to estimate the proportion of LRI cases that are positive for influenza and RSV, separately, by location/year/age/sex. These models are informed by data from a systematic review of cohort, cross-sectional, and clinical trial studies (**Search string 2, Appendix Table 8, Appendix Figure 7**). Our inclusion criteria were studies that were published between January 1990 and June 2016, a sample size of at least 100, at least one year in duration, and with LRI, pneumonia, or bronchiolitis as the case definition. We excluded studies that described pandemic H1N1 influenza solely and studies that used influenza-like illness as the case definition. If the ages of the study participants were not reported, we assigned an age range based on the prevalence-weighted mean age of LRI, from the LRI prevalence DisMod model, for the appropriate year/sex/location. Input data from non-molecular diagnostics such as enzyme-linked immuosorbent assay (ELISA) or viral culture were adjusted to be comparable with our diagnostic case definition based on a polymerase chain reaction (PCR) diagnostic (**Appendix Table 9).** The scalar for our diagnostic methods are based on a mixed-effects regression of the ratio of detection in the non-molecular to the molecular test results.

Appendix Figure 7. Geographic distribution of aetiology data

Number of data points for influenza (649)

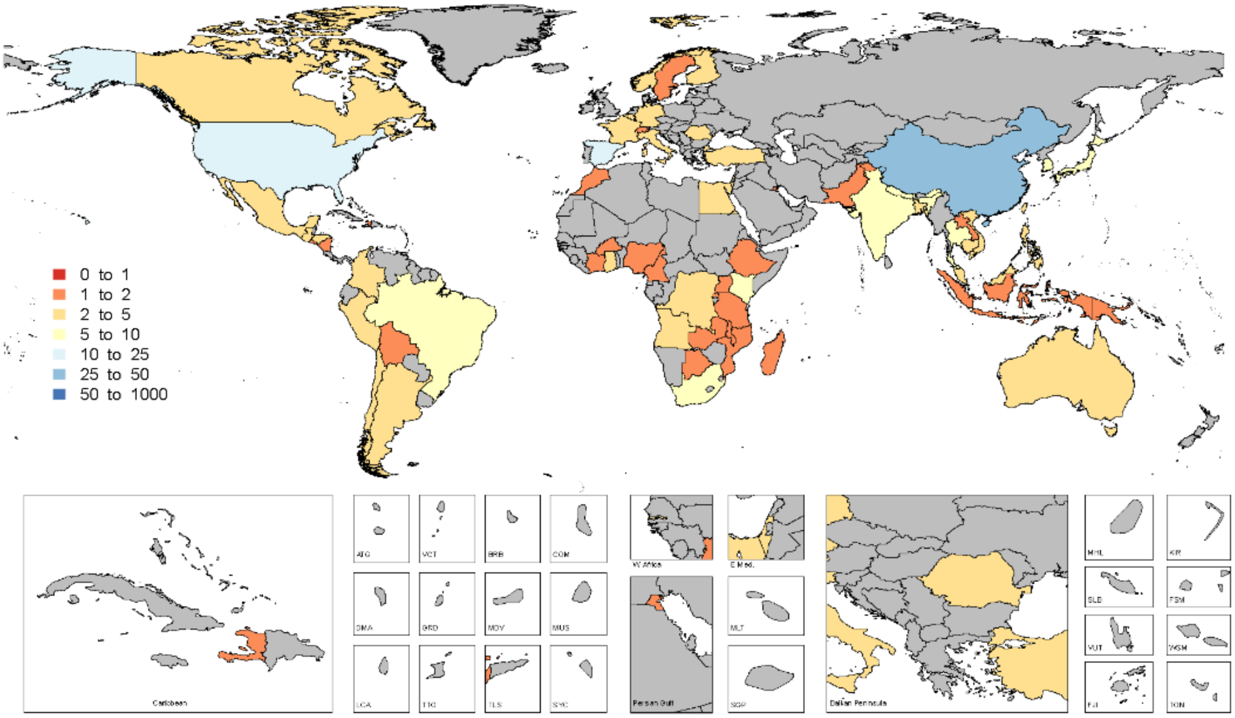

Number of data points for respiratory syncytial virus (483)

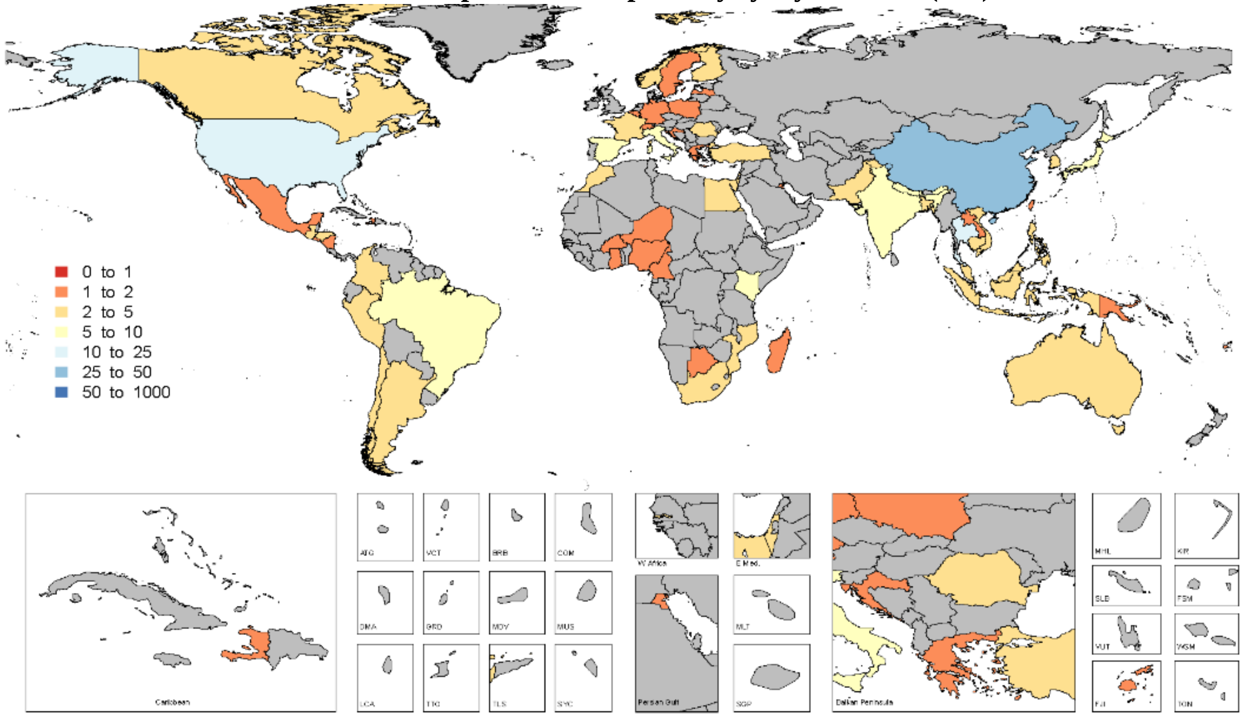

Appendix Table 8. Summary of LRI aetiology data used in attributable fraction estimation

The number of data points does not necessarily indicate the number of unique sources used in the modelling. Data in the modelling are included at the most detailed possible level based on age, year, sex, and geography. More information including meta-data on the sources used in the aetiology modelling can be found on the Global Health Data Exchange: http://ghdx.healthdata.org/gbd-2016

Aetiology	Total data points	Data points new to GBD 2016	Number (percent) from Inpatient population	Number (percent) using PCR diagnostic
Respiratory syncytial virus	483	51 (10.6%)	199 (41.0%)	335 (69.4%)
Influenza	649	167 (25.7%)	349 (53·8%)	392 (60.4%)

There are separate PAF values for nonfatal and fatal LRI episodes. Fatal PAFs are adjusted using a scalar from the DisMod proportion models that represents the relative frequency of detection in inpatient versus non-inpatient sample populations. The value of these scalars are 0·75 (95% UI: 0·65-0·88) for influenza and 1·33 (95% UI: 1·17-1·52) for RSV (**Appendix Table 9**). In addition, as the case-fatality of viral causes of pneumonia is lower than for bacterial causes, we adjusted the fatal PAF estimates by determining the ratio of case-fatality among viral to bacterial causes of pneumonia from hospital data coded specifically to these causes. Hospital data were limited to the USA, Austria, Brazil, and Mexico. We generated an age-specific ratio of case-fatality between viral and bacterial causes of LRI using DisMod-MR (**Appendix Table 10**).

Appendix Table 9. The adjustments for non-reference case definition used in modelling influenza and RSV

Aetiology	Hospitalization scalar	PCR diagnostic scalar
Influenza	0.75 (0.65-0.88)	0.77 (0.65-0.90)
RSV	1.33 (1.17-1.52)	1.2 (1.01-1.42)

Appendix Table 10. The median values for the ratio of case fatality for viral to bacterial pneumonia

These estimates are modelled using hospital-based, ICD-coded admissions and mortality for viral and bacterial pneumonia. Values in parentheses represent the 95% uncertainty interval.

Age Group	Ratio
Early Neonatal	0·34 (0·19-0·58)
Late Neonatal	0·34 (0·19-0·58)
Post Neonatal	0·34 (0·19-0·58)
1 to 4	0·28 (0·16-0·44)
5 to 9	0·31 (0·15-0·56)
10 to 14	0·33 (0·19-0·53)
15 to 19	0·37 (0·2-0·64)
20 to 24	0·46 (0·12-1·16)
25 to 29	0·44 (0·17-0·93)
30 to 34	0·46 (0·22-0·83)
35 to 39	0·5 (0·22-1)
40 to 44	0·61 (0·13-1·75)
45 to 49	0·5 (0·21-0·99)
50 to 54	0·44 (0·23-0·74)
55 to 59	0·42 (0·21-0·75)
60 to 64	0·42 (0·15-0·95)
65 to 69	0·39 (0·19-0·7)
70 to 74	0·38 (0·21-0·61)
75 to 79	0·37 (0·2-0·62)
80 plus	0·37 (0·17-0·71)

Search Strings

1. ('lower respiratory'[title/abstract] OR pneumonia[title/abstract] AND ('2015/01/01'[PDat] : '2016/12/31'[PDat])AND Humans[MeSH Terms] NOT(autoimmune[title/abstract] OR COPD [title/abstract] OR 'cystic fibrosis'[title/abstract])
2. (“lower respiratory”[title/abstract] OR pneumonia[title/abstract] AND (influenza[title/abstract] OR influenza[MeSH Terms] OR “respiratory syncytial”[title/abstract] OR etiolog*[title/abstract]) AND ("2015/01/01"[PDat] : "2016/12/31"[PDat])AND Humans[MeSH Terms]
3. (“haemophilus influenzae type b” OR “haemophilus influenzae b”) AND vaccine AND (efficacy OR effectiveness) AND ("2015/01/01"[PDat] : "2016/12/31"[PDat])AND Humans[MeSH Terms] NOT(autoimmune[title/abstract] OR COPD [title/abstract] OR "cystic fibrosis"[title/abstract])
4. ('streptococcus pneumoniae' OR pneumococcus OR pneumococcal) AND ('conjugate vaccine' OR 'polysaccharide vaccine') AND (efficacy OR effectiveness) AND ('2015/01/01'[PDat] : '2016/12/31'[PDat])AND Humans[MeSH Terms]

Information on the sources for aetiology modelling in GBD 2016 can be found on the Global Health Data Exchange (http://internal-ghdx.healthdata.org/gbd-2016).

Comparison to GBD 2015

The differences in final estimates for LRI mortality between GBD 2015 and GBD 2016 are shown in **Appendix Figure 8.** The number of LRI deaths in children under 5 and in all ages in the year 2015 is very similar between GBD 2015 and GBD 2016 (**Appendix Figure 9**). The main difference in children under-5 occurred in India where a new data source, the Sample Registration System, was included for modelling in GBD 2016. These data are from a large, state-representative survey of deaths and are viewed as reliable and consistent across states. The aetiologic attribution among children under-5 is shown in **Appendix Figure 10.** There has been relative consistency across the most recent iterations of the GBD cycle in terms of the global attributable fraction to influenza and respiratory syncytial virus. The attribution of pneumococcal pneumonia increased sharply from GBD 2013 to GBD 2015 due to an adjustment for invasive pneumococcal disease, described above in this appendix.

Appendix Figure 8. Scatterplot of GBD 2015 and GBD 2016 deaths by country among children under-5 in 2015

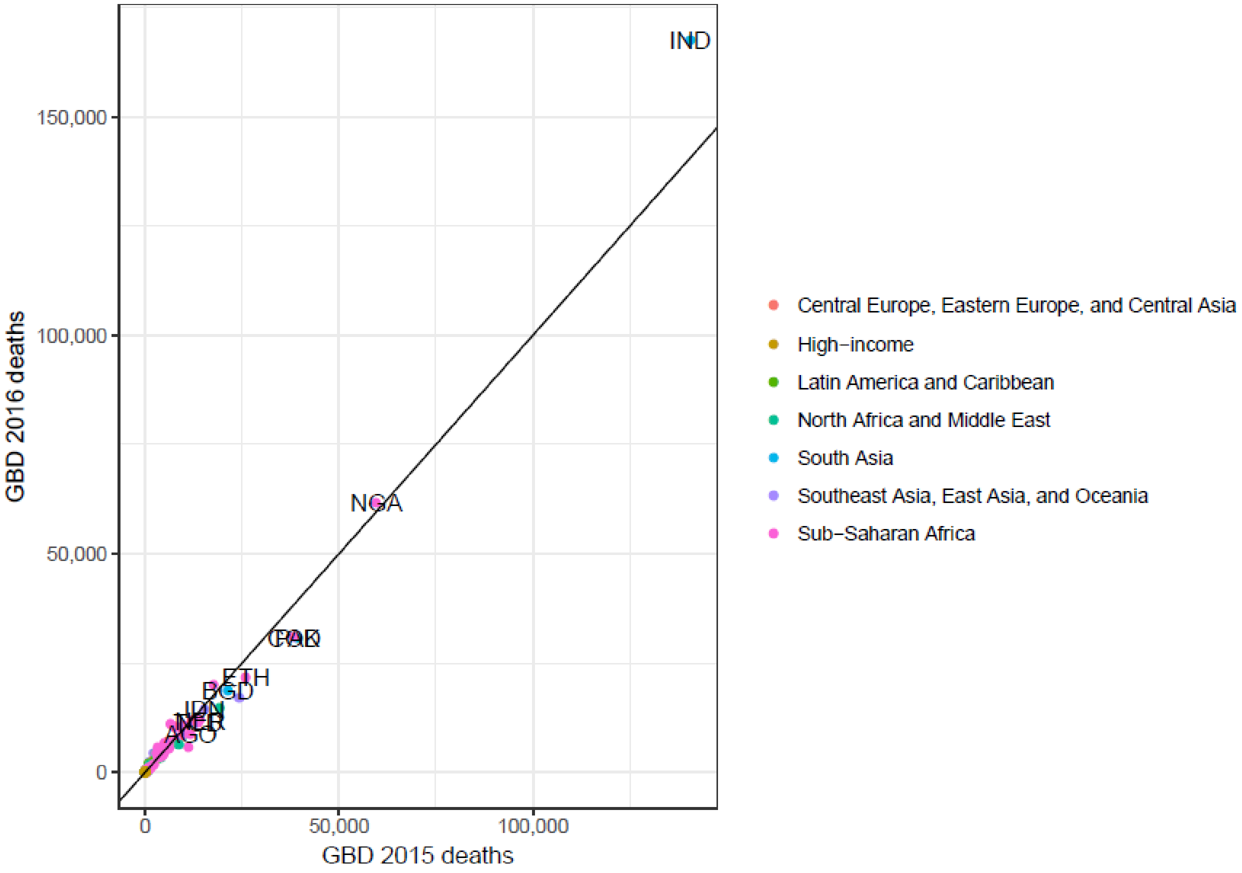

Appendix Figure 9. The number of deaths over time for all ages and for children under-5 in GBD 2015 and GBD 2016

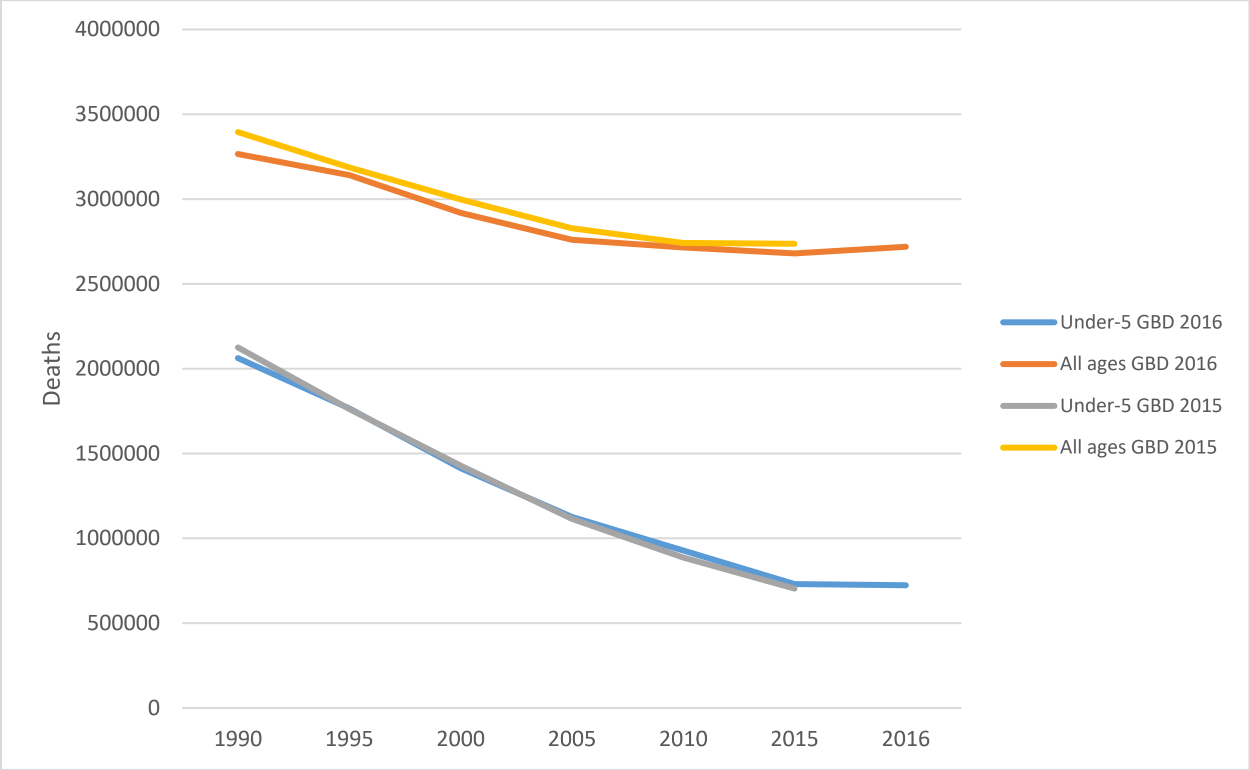

Appendix Figure 10. Aetiologic attribution to under-5 LRI deaths in 2010

The percent of under-5 LRI deaths due to each aetiology is shown in the bar charts below in GBD 2013,[18] in GBD 2015, and in GBD 2016.

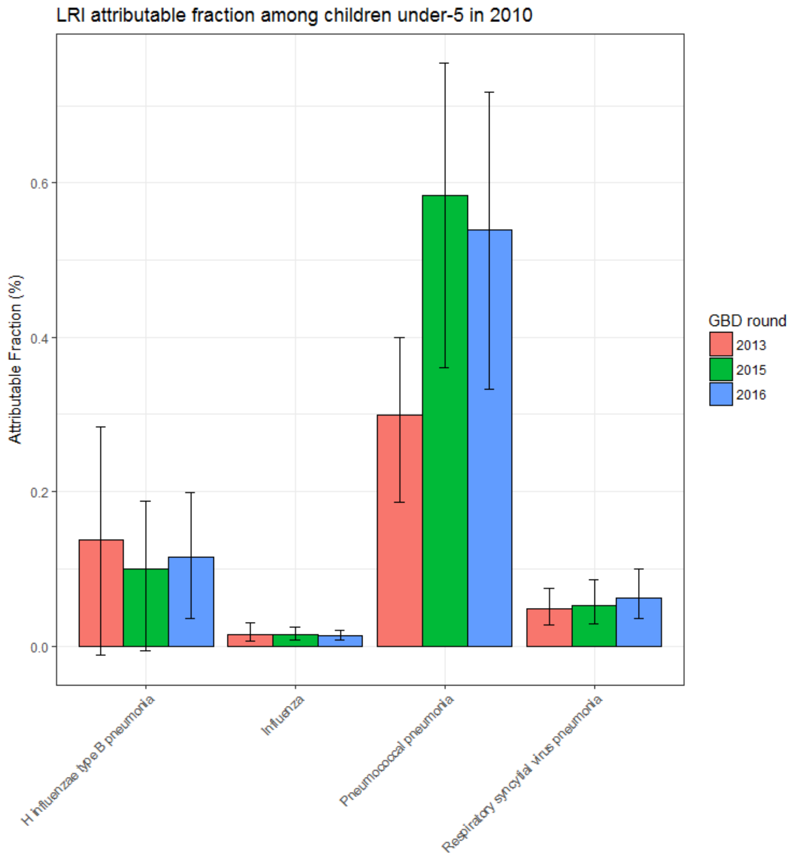

Comparison with other estimates

The World Health Organization and the Maternal and Child Epidemiology Estimation group produce global and national estimates of the number of deaths among children under-5 due to lower respiratory infections. **Appendix Figure 11** shows the number of deaths due to LRI in 2000 and 2015 among the two groups. Several select countries are shown in **Appendix Table 11.** The largest differences occurred in India, Pakistan, and Nigeria. Modelled estimates in these countries depend on covariates and input data, as well as the modelling approach, all of which are different between the WHO-MCEE estimates and the GBD 2016 estimates. For example, GBD 2016 produces estimates at the state urban/rural level in India, relying on the Sample Registration System dataset, which allows for much greater within country variation than modelling at the national level only. Estimates in Nigeria for GBD 2016 are informed by a pair of subnational verbal autopsy studies which may not be representative of the nation as a whole. Models in the GBD are strengthened by spatio-temporal patterns and by covariates. Still, there is a great deal of uncertainty around the estimates in Nigeria and Western Sub-Saharan African countries generally.

Appendix Figure 11. Scatterplot of under-5 LRI deaths in comparing GBD 2016 and the WHO-MCEE group final estimates.[19]

A) Number of deaths in 2000, B) Number of deaths in 2015.

A)

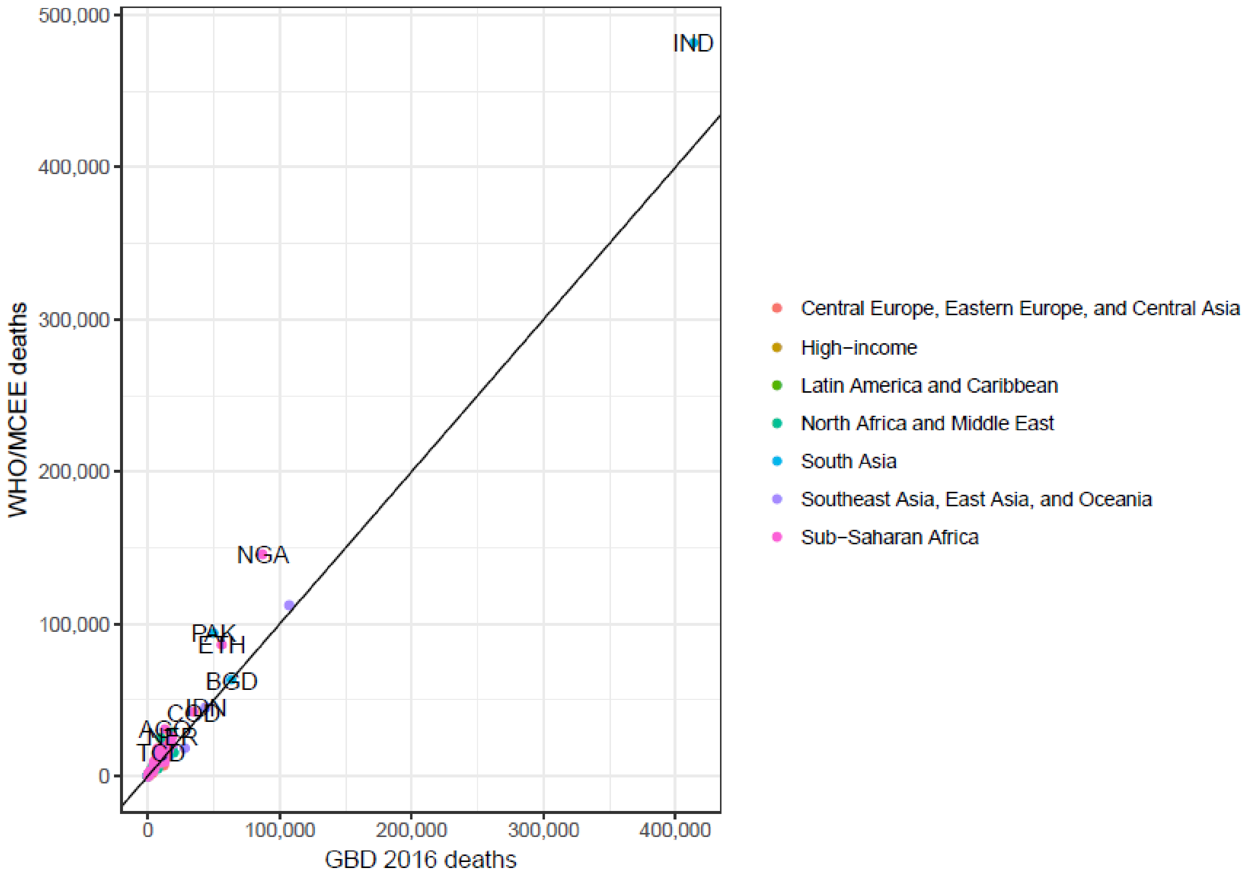

B)

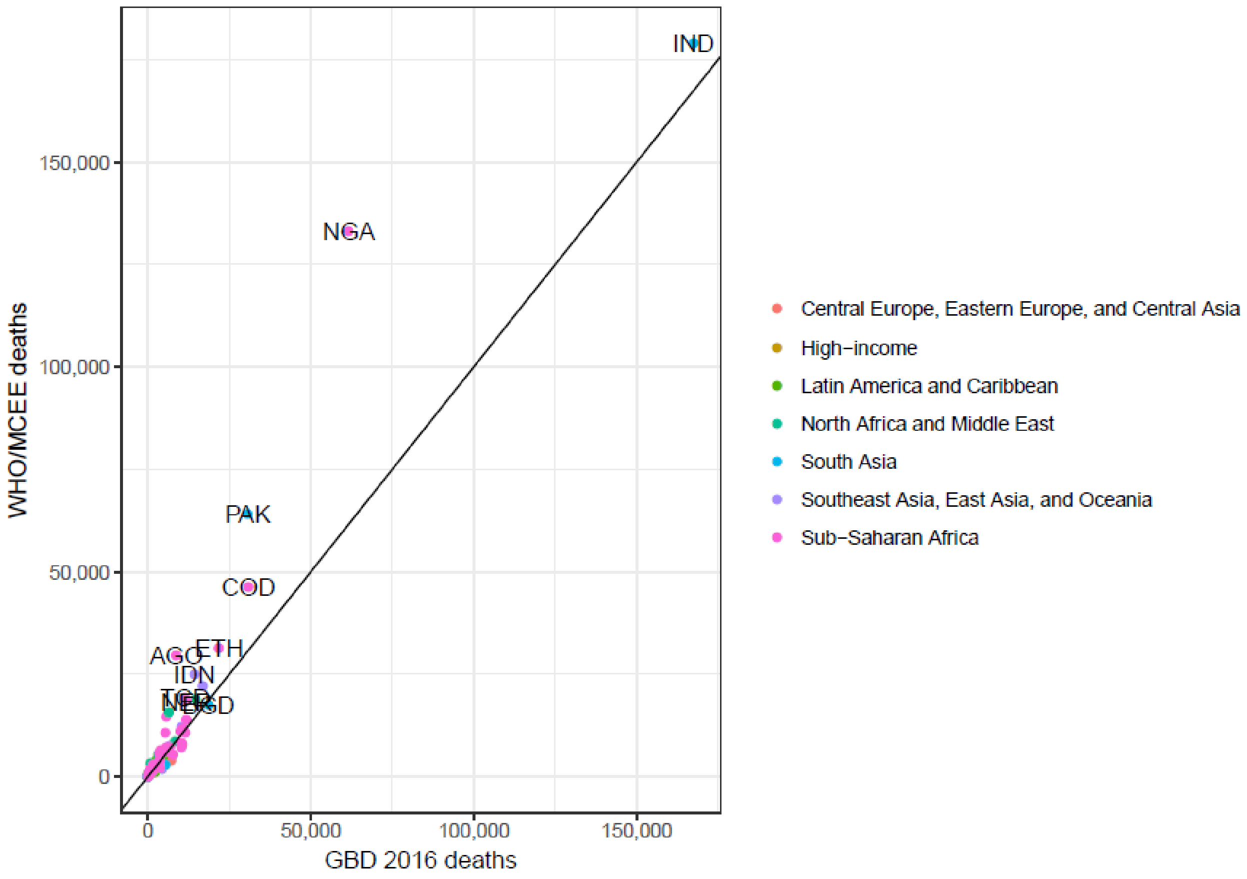
IND
NGA
PAK
COD
AGO
ETH
IDN
BGD
150,000
100,000
50,000
0
WHO/MCEE deaths
0
50,000
100,000
150,000
GBD 2016 deaths
Central Europe, Eastern Europe, and Central Asia
High−income
Latin America and Caribbean
North Africa and Middle East
South Asia
Southeast Asia, East Asia, and Oceania
Sub−Saharan Africa

Appendix Table 11. The number of deaths due to lower respiratory infections

The number of deaths due to lower respiratory infections among children under-5 in the year 2015 is shown for the WHO-MCEE group as well as for GBD 2015 and GBD 2016.

Location	WHO-MCEE	GBD 2015	GBD 2016
**Global**	**920,136**	**703,918**	**701,000**
Indonesia	25,000	15,250	14,300
India	178,994	140,649	167,500
Pakistan	63,960	39,158	30,600
Democratic Republic of the Congo	46,226	38,357	30,800
Ethiopia	31,427	25,971	21,800
Nigeria	133,239	59,645	61,600

Our estimates of the number of LRI episodes among children under-5 in 2010 (74,130,000, 95% UI 60,610,000-89,700,000) are about half of the estimates produced by the Child Health Epidemiology Research Group (CHERG) (120,400,000, 95% UI 60,800,000-277,000,000) but with overlap in the 95% uncertainty intervals.[20] The estimates produced by the MCEE are informed by the incidence of pneumonia in 35 cohort studies which informed an envelope of pneumonia incidence that was related to the prevalence of five risk factors for pneumonia to estimate country-level incidence.[21] While there is overlap in the risk factors used in both studies, GBD 2016 utilised over 30,000 data points from more than 700 sources to produce internally consistent estimates of LRI incidence, prevalence, and mortality.

References

1 Naghavi M, Makela S, Foreman K, O'Brien J, Pourmalek F, Lozano R. Algorithms for enhancing public health utility of national causes-of-death data. *Popul Health Metr* 2010; **8**: 9.

2 GBD 2015 Mortality and Causes of Death Collaborators. Global, regional, and national life expectancy, all-cause and cause-specific mortality for 249 causes of death, 1980–2015: a systematic analysis for the Global Burden of Disease Study 2015. *Lancet* 2016; **388**: 1459–544.

3 Foreman KJ, Lozano R, Lopez AD, Murray CJ. Modeling causes of death: an integrated approach using CODEm. *Popul Health Metr* 2012; **10**: 1.

4 Foreman KJ, Lozano R, Lopez AD, Murray CJ. Modeling causes of death: an integrated approach using CODEm. *Popul Health Metr* 2012; **10**: 1.

5 GBD 2016 Mortality Collaborators. Global, regional, and national under-5 mortality, adult mortality, age-specific mortality, and life expectancy, 1970-2016: a systematic analysis for the Global Burden of Disease Study 2016. *Lancet* 2017; **390**: 1084–150.

6 World Health Organization: Department of Child and Adolescent Health and Development. Handbook Integrated Management of Childhood Illness. 2005.

7 Salomon JA, Haagsma JA, Davis A, *et al.* Disability weights for the Global Burden of Disease 2013 study. *Lancet Glob Health* 2015; **3**: e712-723.

8 GBD 2015 Risk Factors Collaborators. Global, regional, and national comparative risk assessment of 79 behavioural, environmental and occupational, and metabolic risks or clusters of risks, 1990-2015: a systematic analysis for the Global Burden of Disease Study 2015. *Lancet* 2016; **388**: 1659–724.

9 Feikin DR, Scott JAG, Gessner BD. Use of vaccines as probes to define disease burden. *Lancet* 2014; **383**: 1762–70.

10 O'Brien KL, Wolfson LJ, Watt JP, *et al.* Burden of disease caused by Streptococcus pneumoniae in children younger than 5 years: global estimates. *Lancet* 2009; **374**: 893–902.

11 Johnson HL, Deloria-Knoll M, Levine OS, *et al.* Systematic evaluation of serotypes causing invasive pneumococcal disease among children under five: the pneumococcal global serotype project. *PLoS Med* 2010; **7**. DOI:10.1371/journal.pmed.1000348.

12 Watt JP, Wolfson LJ, O'Brien KL, *et al.* Burden of disease caused by Haemophilus influenzae type b in children younger than 5 years: global estimates. *Lancet* 2009; **374**: 903–11.

13 Swingler G, Fransman D, Hussey G. Conjugate vaccines for preventing Haemophilus influenzae type B infections. *Cochrane Database Syst Rev* 2007; : CD001729.

14 Lucero MG, Dulalia VE, Nillos LT, *et al.* Pneumococcal conjugate vaccines for preventing vaccine-type invasive pneumococcal disease and X-ray defined pneumonia in children less than two years of age. *Cochrane Database Syst Rev* 2009; : CD004977.

15 Bonten MJM, Huijts SM, Bolkenbaas M, *et al.* Polysaccharide conjugate vaccine against pneumococcal pneumonia in adults. *N Engl J Med* 2015; **372**: 1114–25.

16 Miettinen OS. Proportion of disease caused or prevented by a given exposure, trait or intervention. *Am J Epidemiol* 1974; **99**: 325–32.

17 Shi T, McLean K, Campbell H, Nair H. Aetiological role of common respiratory viruses in acute lower respiratory infections in children under five years: A systematic review and meta-analysis. *J Glob Health* 2015; **5**: 010408.

18 GBD 2013 Mortality and Causes of Death Collaborators. Global, regional, and national age-sex specific all-cause and cause-specific mortality for 240 causes of death, 1990-2013: a systematic analysis for the Global Burden of Disease Study 2013. *Lancet* 2015; **385**: 117–71.

19 WHO. Estimates for 2000-2015. http://www.who.int/ healthinfo/global_burden_disease/estimates_child_cod_2015/en/ (accessed Aug 25, 2016).

20 Walker CLF, Rudan I, Liu L, *et al.* Global burden of childhood pneumonia and diarrhoea. *The Lancet* 2013; **381**: 1405–16.

21 Rudan I, O'Brien KL, Nair H, *et al.* Epidemiology and etiology of childhood pneumonia in 2010: estimates of incidence, severe morbidity, mortality, underlying risk factors and causative pathogens for 192 countries. *J Glob Health* 2013; **3**. DOI:10.7189/jogh.03.010401.

22 Shi T, McAllister DA, O'Brien KL, *et al.* Global, regional, and national disease burden estimates of acute lower respiratory infections due to respiratory syncytial virus in young children in 2015: a systematic review and modelling study. *Lancet* 2017; **390**: 946–58.

23 Iuliano AD, Roguski KM, Chang HH, *et al.* Estimates of global seasonal influenza-associated respiratory mortality: a modelling study. *Lancet* 2018; **391**: 1285–300.

Supplementary Results

This section contains supplementary results for the manuscript. All models and results can be found using several online resources for a curious reader.

- **GBD Compare: https://vizhub.healthdata.org/gbd-compare/**
  - This resource provides the most current estimates for nearly all results available from the Global Burden of Disease Study and allows the user to take screenshots, download the data available in the webpage, and gives a comprehensive picture of health loss globally.

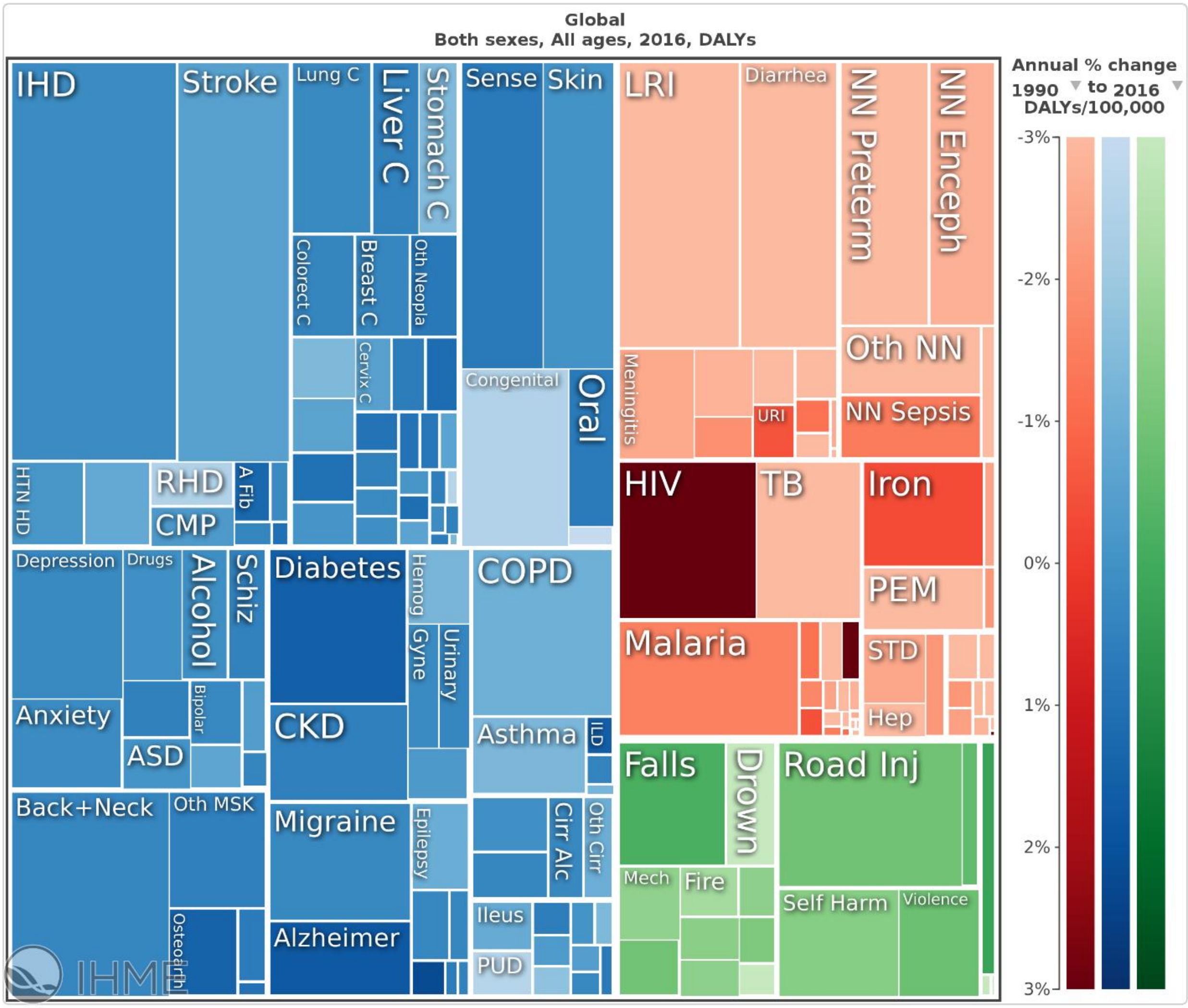

- **Global Health Data Exchange: https://ghdx.healthdata.org/**
  - This resource provides a comprehensive library of health data used in the Global Burden of Disease study and is intended to be a resource available for the entire global health community to identify data and sources.

- **Cause of Death modelling: https://vizhub.healthdata.org/cod/**
  - This tool shows the intermediate models for causes of death in the global burden of disease study, including for lower respiratory infections (**http://ihmeuw.org/4dp1)**. The tool shows the final model fits used in the GBD 2016 including input data for every location and age group.

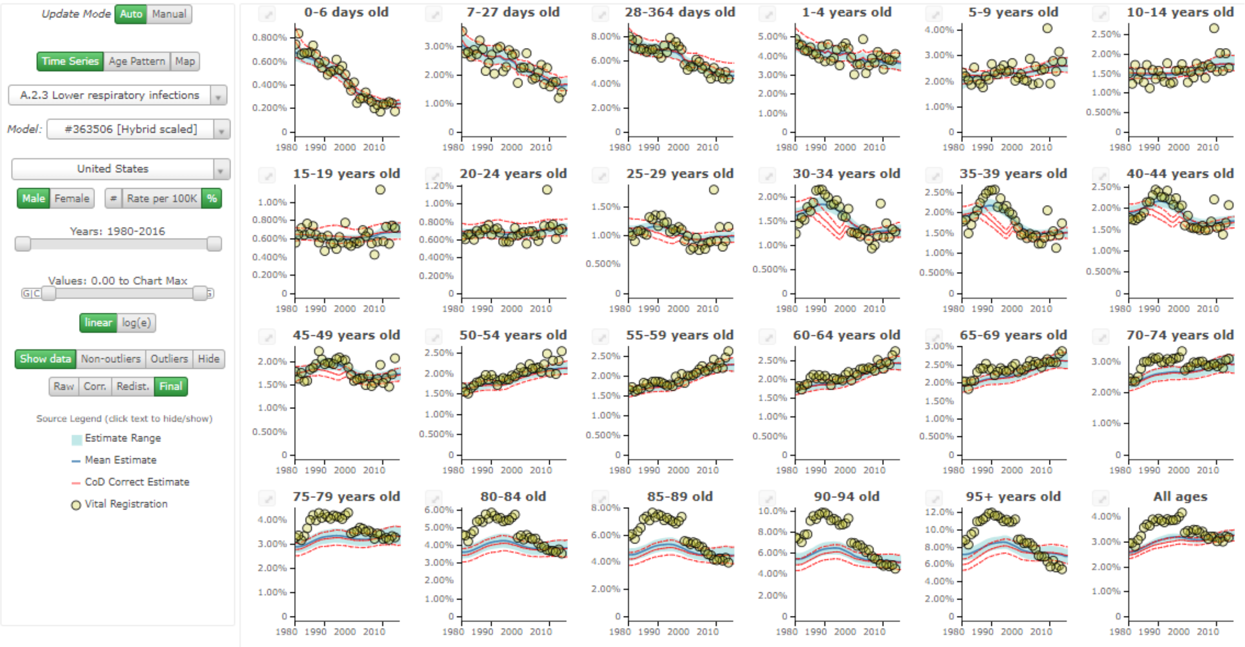

- **DisMod-MR 2.1:** https://vizhub.healthdata.org/epi/
    - This tool shows the model results for the non-fatal models used in the Global Burden of Disease study 2016, including for lower respiratory infections (http://ihmeuw.org/4dp2). The tool shows the model fits and input data.
    -

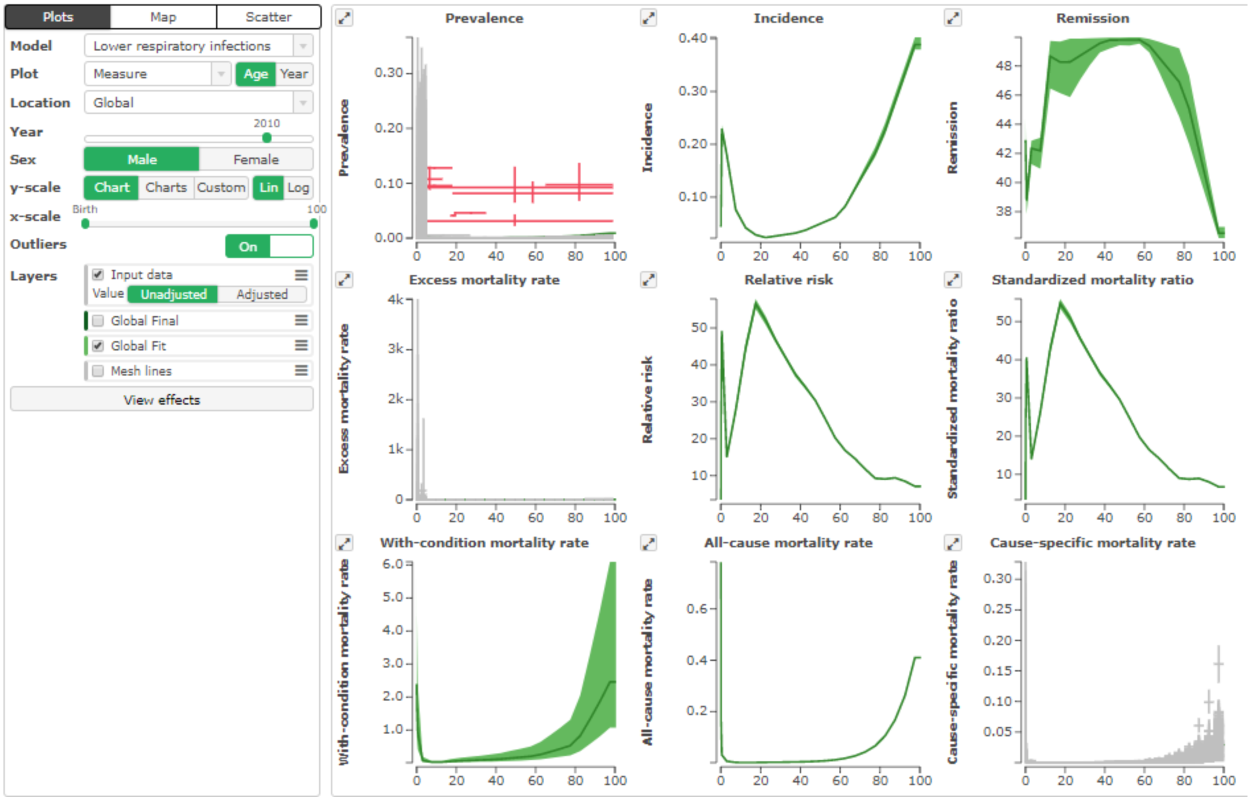

Appendix Table 12. Episodes and deaths among all ages, children under 5, and adults over 70 in 2016 by geography

Super-regions and regions have been highlighted in green. Super-regions (7) and regions (20) are groups of countries that share a geography and development status and are hypothesised to share similar disease burdens. Spatial information in the modelling for GBD is passed down an analytic cascade from global to super-region to region to country such that countries within a region are more similar to each other than to others outside that region. Due to rounding, some values in the table are zero (e.g. 0 [0-0]).

	All Ages				Children under-5				Adults over-70			
**Location**	**Deaths (95% UI)**	**Deaths per 1000 (95% UI)**	**Millions of cases (95% UI)**	**Incidence per 1000 (95% UI)**	**Deaths (95% UI)**	**Deaths per 1000 (95% UI)**	**Millions of cases (95% UI)**	**Incidence per 1000 (95% UI)**	**Deaths (95% UI)**	**Deaths per 1000 (95% UI)**	**Millions of cases (95% UI)**	**Incidence per 1000 (95% UI)**
**Global**	**2,377,697 (2,145,584-2,512,809)**	**32.2 (29.0-34.0)**	**336.46 (313.08-361.62)**	**45.5 (42.4-48.9)**	**652,572 (586,475-720,612)**	**103.3 (92.8-114.0)**	**68.06 (55.29-82.72)**	**107.7 (87.5-130.9)**	**1,080,958 (943,749-1,170,638)**	**267.4 (233.4-289.6)**	**62.84 (57.15-68.75)**	**155.4 (141.4-170.1)**
**High-income**	**392,940 (362,375-425,101)**	**37.0 (34.1-40.0)**	**38.13 (35.84-40.52)**	**35.9 (33.8-38.2)**	**1,905 (1,749-2,105)**	**3.3 (3.0-3.6)**	**2.58 (2.05-3.25)**	**44.6 (35.5-56.3)**	**337,271 (307,354-367,534)**	**252.5 (230.1-275.1)**	**13.49 (12.32-14.75)**	**101.0 (92.2-110.4)**
**High-income North America**	**105,127 (98,114-112,332)**	**29.3 (27.3-31.3)**	**13.93 (13.12-14.73)**	**38.8 (36.5-41.0)**	**669 (613-730)**	**3.1 (2.8-3.4)**	**1.19 (0.97-1.46)**	**55.1 (44.8-67.5)**	**81,270 (74,817-87,883)**	**227.6 (209.6-246.2)**	**2.61 (2.38-2.84)**	**73.2 (66.7-79.5)**
Canada	9,123 (7,934-10,482)	25.1 (21.8-28.9)	1.24 (1.14-1.35)	34.3 (31.4-37.1)	41 (32-53)	2.1 (1.7-2.7)	0.09 (0.07-0.12)	45.5 (34.3-60.4)	7,971 (6,811-9,287)	209.1 (178.7-243.6)	0.29 (0.25-0.33)	75.6 (66.2-85.3)
Greenland	12 (6-15)	23.2 (12.1-31.1)	0.00 (0.00-0.00)	35.2 (31.8-38.7)	0 (0-0)	9.0 (6.2-12.4)	0.00 (0.00-0.00)	55.8 (43.0-72.5)	7 (3-9)	325.2 (165.5-445.0)	0.00 (0.00-0.00)	89.0 (75.6-105.3)
United States	95,992 (89,607-102,347)	29.7 (27.8-31.7)	12.68 (11.96-13.40)	39.3 (37.0-41.5)	628 (575-685)	3.2 (2.9-3.5)	1.10 (0.90-1.34)	56.0 (45.7-68.3)	73,293 (67,384-79,204)	229.9 (211.4-248.5)	2.32 (2.12-2.52)	72.9 (66.6-79.2)
Australasia	5,164 (4,532-5,848)	18.0 (15.8-20.4)	1.26 (1.17-1.36)	43.9 (40.7-47.5)	36 (30-44)	2.0 (1.7-2.5)	0.11 (0.08-0.14)	60.5 (46.4-77.8)	4,623 (3,999-5,305)	161.8 (140.0-185.6)	0.47 (0.42-0.53)	165.6 (147.4-184.8)
Australia	4,424 (3,831-5,062)	18.4 (15.9-21.0)	1.12 (1.03-1.22)	46.6 (42.9-50.7)	27 (22-34)	1.8 (1.5-2.3)	0.10 (0.07-0.12)	63.8 (48.5-83.2)	3,937 (3,365-4,569)	163.9 (140.1-190.2)	0.42 (0.37-0.47)	172.9 (152.6-194.6)
New Zealand	740 (619-878)	16.3 (13.6-19.3)	0.14 (0.13-0.14)	29.7 (28.0-31.5)	9 (7-11)	3.1 (2.4-3.9)	0.01 (0.01-0.02)	42.8 (33.9-53.3)	686 (571-821)	150.9 (125.5-180.6)	0.06 (0.05-0.06)	127.4 (116.1-138.4)
**High-income Asia Pacific**	**109,683 (98,788-121,621)**	**60.8 (54.8-67.4)**	**8.12 (7.56-8.73)**	**45.0 (41.9-48.4)**	**194 (171-220)**	**2.6 (2.3-3.0)**	**0.48 (0.36-0.62)**	**64.6 (48.9-84.8)**	**100,501 (90,040-112,207)**	**337.2 (302.1-376.4)**	**3.59 (3.25-3.96)**	**120.6 (109.1-132.9)**
Brunei	63 (52-78)	14.7 (12.1-18.3)	0.01 (0.01-0.02)	35.0 (31.4-38.9)	4 (3-5)	11.4 (9.0-14.4)	0.00 (0.00-0.00)	73.2 (55.9-93.1)	37 (30-50)	328.6 (262.6-442.8)	0.00 (0.00-0.00)	118.1 (102.3-135.2)

Location	All Ages				Children under-5				Adults over-70			
	**Deaths (95% UI)**	**Deaths per 1000 (95% UI)**	**Millions of cases (95% UI)**	**Incidence per 1000 (95% UI)**	**Deaths (95% UI)**	**Deaths per 1000 (95% UI)**	**Millions of cases (95% UI)**	**Incidence per 1000 (95% UI)**	**Deaths (95% UI)**	**Deaths per 1000 (95% UI)**	**Millions of cases (95% UI)**	**Incidence per 1000 (95% UI)**
Japan	96,263 (86,782-107,148)	76.6 (69.0-85.3)	6.22 (5.78-6.70)	49.5 (46.0-53.3)	138 (122-153)	2.7 (2.4-3.0)	0.32 (0.24-0.41)	63.3 (48.3-82.5)	88,926 (79,571-99,260)	358.4 (320.7-400.1)	3.09 (2.80-3.40)	124.5 (112.8-137.0)
Singapore	2,313 (1,902-2,796)	58.8 (48.4-71.1)	0.20 (0.19-0.22)	51.4 (47.4-55.7)	10 (7-13)	5.7 (4.2-7.3)	0.01 (0.01-0.02)	78.8 (59.8-101.2)	1,866 (1,528-2,281)	573.6 (469.7-701.1)	0.06 (0.05-0.07)	180.2 (158.1-201.7)
South Korea	11,044 (7,706-14,495)	21.9 (15.3-28.8)	1.68 (1.53-1.84)	33.4 (30.5-36.5)	42 (28-59)	2.0 (1.3-2.8)	0.14 (0.11-0.19)	66.4 (49.4-88.2)	9,672 (6,664-12,797)	207.6 (143.0-274.7)	0.44 (0.39-0.50)	95.4 (82.8-107.7)
**Western Europe**	**138,945 (126,055-152,808)**	**32.4 (29.4-35.7)**	**12.28 (11.47-13.20)**	**28.7 (26.8-30.8)**	**379 (345-425)**	**1.7 (1.6-1.9)**	**0.51 (0.39-0.67)**	**23.4 (17.9-30.5)**	**124,805 (112,377-138,463)**	**207.4 (186.7-230.1)**	**6.10 (5.56-6.67)**	**101.3 (92.4-110.8)**
Andorra	38 (22-52)	47.7 (27.2-65.7)	0.00 (0.00-0.00)	31.1 (28.0-34.3)	0 (0-0)	2.8 (1.6-5.2)	0.00 (0.00-0.00)	24.6 (18.3-33.2)	35 (20-48)	311.8 (179.8-433.5)	0.00 (0.00-0.00)	116.6 (101.1-132.4)
Austria	762 (643-896)	8.8 (7.4-10.3)	0.17 (0.16-0.19)	19.6 (18.0-21.4)	4 (3-6)	1.1 (0.7-1.5)	0.01 (0.01-0.01)	19.3 (14.8-25.2)	662 (546-796)	54.6 (45.1-65.7)	0.08 (0.07-0.09)	64.1 (55.7-72.4)
Belgium	5,162 (4,399-6,059)	45.4 (38.7-53.3)	0.39 (0.36-0.42)	34.1 (31.4-37.2)	12 (9-15)	1.8 (1.4-2.3)	0.02 (0.01-0.02)	27.1 (20.5-35.1)	4,585 (3,862-5,447)	313.9 (264.4-372.9)	0.19 (0.17-0.22)	131.5 (118.1-147.4)
Cyprus	147 (118-211)	16.2 (13.0-23.2)	0.01 (0.01-0.02)	15.8 (14.5-17.1)	1 (0-1)	1.5 (1.0-2.0)	0.00 (0.00-0.00)	16.7 (12.4-21.7)	130 (101-193)	146.8 (114.6-218.1)	0.01 (0.01-0.01)	66.9 (58.7-75.2)
Denmark	2,316 (1,980-2,696)	40.5 (34.6-47.1)	0.18 (0.16-0.19)	31.0 (28.6-33.6)	5 (4-7)	1.8 (1.3-2.3)	0.01 (0.01-0.01)	23.9 (18.0-31.7)	2,117 (1,795-2,480)	290.7 (246.6-340.6)	0.09 (0.08-0.10)	120.1 (107.9-134.3)
Finland	924 (763-1,105)	16.8 (13.9-20.1)	0.13 (0.12-0.14)	23.4 (21.4-25.6)	1 (1-2)	0.5 (0.3-0.6)	0.01 (0.00-0.01)	21.2 (15.9-27.6)	833 (674-1,009)	111.8 (90.5-135.4)	0.06 (0.05-0.07)	80.1 (69.9-90.3)
France	20,666 (17,737-23,921)	31.8 (27.3-36.8)	2.75 (2.53-3.01)	42.4 (38.9-46.4)	46 (34-61)	1.2 (0.9-1.5)	0.16 (0.12-0.21)	39.7 (29.6-52.8)	18,623 (15,729-21,898)	219.4 (185.3-258.0)	1.25 (1.10-1.41)	146.8 (129.5-166.7)
Germany	22,425 (19,321-25,902)	27.3 (23.5-31.6)	2.56 (2.37-2.80)	31.2 (28.9-34.1)	42 (32-56)	1.2 (0.9-1.6)	0.09 (0.07-0.12)	27.1 (20.8-35.1)	19,390 (16,440-22,647)	141.7 (120.1-165.5)	1.33 (1.18-1.51)	97.3 (86.5-110.0)
Greece	2,675 (2,323-3,078)	24.6 (21.4-28.3)	0.28 (0.26-0.30)	25.8 (23.7-27.9)	14 (9-21)	2.9 (2.0-4.4)	0.01 (0.01-0.01)	23.0 (17.3-30.1)	2,350 (2,006-2,743)	137.9 (117.7-160.9)	0.14 (0.12-0.15)	81.5 (71.6-90.2)

	All Ages				Children under-5				Adults over-70			
**Location**	**Deaths (95% UI)**	**Deaths per 1000 (95% UI)**	**Millions of cases (95% UI)**	**Incidence per 1000 (95% UI)**	**Deaths (95% UI)**	**Deaths per 1000 (95% UI)**	**Millions of cases (95% UI)**	**Incidence per 1000 (95% UI)**	**Deaths (95% UI)**	**Deaths per 1000 (95% UI)**	**Millions of cases (95% UI)**	**Incidence per 1000 (95% UI)**
Iceland	95 (81-112)	28.8 (24.4-33.9)	0.01 (0.01-0.01)	26.9 (24.8-29.3)	0 (0-0)	1.7 (1.2-2.3)	0.00 (0.00-0.00)	23.3 (17.6-30.2)	85 (71-101)	276.2 (230.5-330.4)	0.00 (0.00-0.00)	123.9 (110.3-139.6)
Ireland	1,343 (1,133-1,591)	28.9 (24.4-34.3)	0.11 (0.10-0.12)	23.4 (21.4-25.6)	5 (3-6)	1.3 (0.9-1.9)	0.01 (0.00-0.01)	19.2 (14.2-25.2)	1,212 (1,014-1,445)	292.4 (244.8-348.8)	0.05 (0.04-0.05)	110.3 (97.9-123.4)
Israel	1,705 (1,424-2,042)	20.8 (17.4-24.9)	0.18 (0.16-0.19)	21.6 (19.8-23.6)	16 (12-20)	1.9 (1.5-2.4)	0.02 (0.01-0.02)	18.2 (13.8-23.7)	1,434 (1,174-1,726)	233.0 (190.7-280.4)	0.06 (0.06-0.07)	105.6 (92.8-118.7)
Italy	9,226 (7,913-10,907)	15.2 (13.1-18.0)	1.16 (1.07-1.26)	19.1 (17.6-20.9)	33 (22-47)	1.3 (0.9-1.9)	0.04 (0.03-0.05)	14.9 (11.4-19.1)	8,228 (6,981-9,843)	82.9 (70.3-99.2)	0.63 (0.56-0.71)	63.8 (56.4-71.3)
Luxembourg	126 (106-147)	21.8 (18.4-25.4)	0.01 (0.01-0.01)	15.3 (14.1-16.7)	0 (0-0)	1.0 (0.7-1.4)	0.00 (0.00-0.00)	13.0 (10.0-16.8)	114 (94-134)	195.7 (161.8-231.0)	0.00 (0.00-0.00)	70.9 (62.4-79.5)
Malta	124 (104-149)	29.6 (24.8-35.4)	0.01 (0.01-0.01)	21.8 (20.2-23.6)	1 (0-1)	3.5 (2.6-4.6)	0.00 (0.00-0.00)	16.8 (12.9-21.7)	109 (91-132)	202.0 (168.0-243.2)	0.00 (0.00-0.00)	79.3 (71.4-87.9)
Netherlands	7,298 (6,410-8,371)	42.6 (37.4-48.8)	0.41 (0.37-0.44)	23.8 (21.8-25.9)	12 (9-15)	1.4 (1.0-1.8)	0.01 (0.01-0.02)	14.8 (11.0-19.6)	6,670 (5,784-7,701)	330.3 (286.5-381.4)	0.20 (0.18-0.23)	101.4 (90.1-114.0)
Norway	1,767 (1,478-2,068)	33.6 (28.1-39.4)	0.12 (0.11-0.13)	23.2 (21.5-25.2)	3 (2-5)	1.2 (0.8-1.5)	0.01 (0.00-0.01)	17.2 (13.1-22.5)	1,665 (1,379-1,968)	291.5 (241.5-344.6)	0.06 (0.05-0.07)	104.9 (93.4-118.1)
Portugal	6,329 (5,590-7,264)	60.4 (53.4-69.3)	0.41 (0.38-0.44)	39.1 (35.9-42.3)	11 (9-15)	2.7 (2.1-3.4)	0.01 (0.01-0.01)	25.0 (19.0-32.7)	5,665 (4,968-6,579)	366.0 (321.0-425.0)	0.22 (0.19-0.24)	140.6 (125.7-155.6)
Spain	10,930 (9,371-12,585)	23.5 (20.2-27.1)	0.91 (0.84-0.99)	19.7 (18.1-21.4)	31 (22-42)	1.4 (1.0-2.0)	0.03 (0.02-0.04)	13.6 (10.0-18.1)	9,737 (8,242-11,308)	152.5 (129.1-177.1)	0.46 (0.41-0.52)	72.1 (64.0-81.5)
Sweden	3,079 (2,585-3,610)	31.1 (26.1-36.5)	0.26 (0.24-0.28)	26.2 (24.0-28.6)	7 (5-9)	1.2 (0.8-1.6)	0.01 (0.01-0.02)	21.5 (16.1-28.9)	2,833 (2,360-3,351)	208.6 (173.7-246.6)	0.13 (0.12-0.15)	96.9 (85.5-109.0)
Switzerland	1,805 (1,409-2,246)	21.5 (16.8-26.8)	0.17 (0.16-0.18)	20.2 (18.5-21.8)	6 (4-8)	1.4 (1.0-2.0)	0.01 (0.00-0.01)	14.7 (11.4-18.8)	1,673 (1,296-2,095)	156.1 (121.0-195.5)	0.09 (0.08-0.10)	80.7 (71.2-89.3)
United Kingdom	40,001 (36,368-43,750)	61.2 (55.6-66.9)	2.04 (1.90-2.19)	31.2 (29.1-33.5)	130 (121-138)	3.3 (3.1-3.5)	0.08 (0.06-0.10)	19.8 (15.0-26.2)	36,657 (33,088-40,365)	460.5 (415.7-507.1)	1.04 (0.94-1.14)	130.0 (117.7-143.2)

Location	All Ages				Children under-5				Adults over-70			
	Deaths (95% UI)	Deaths per 1000 (95% UI)	Millions of cases (95% UI)	Incidence per 1000 (95% UI)	Deaths (95% UI)	Deaths per 1000 (95% UI)	Millions of cases (95% UI)	Incidence per 1000 (95% UI)	Deaths (95% UI)	Deaths per 1000 (95% UI)	Millions of cases (95% UI)	Incidence per 1000 (95% UI)
England	34,016 (30,932-37,147)	61.8 (56.2-67.5)	1.65 (1.53-1.77)	29.9 (27.9-32.2)	115 (107-123)	3.5 (3.2-3.7)	0.06 (0.05-0.08)	18.4 (14.0-24.3)	31,262 (28,251-34,373)	469.7 (424.5-516.5)	0.84 (0.76-0.93)	126.7 (114.3-139.7)
Northern Ireland	1,106 (930-1,294)	59.4 (50.0-69.5)	0.06 (0.05-0.06)	31.3 (28.5-34.4)	4 (3-6)	3.5 (2.2-5.3)	0.00 (0.00-0.00)	22.7 (16.9-29.8)	1,002 (836-1,183)	492.8 (410.9-581.7)	0.03 (0.02-0.03)	138.6 (122.3-157.0)
Scotland	3,024 (2,575-3,492)	56.0 (47.7-64.6)	0.23 (0.21-0.25)	43.0 (39.6-47.0)	6 (4-8)	2.1 (1.5-2.9)	0.01 (0.01-0.01)	33.7 (25.4-45.5)	2,706 (2,275-3,153)	399.1 (335.6-465.0)	0.11 (0.10-0.13)	165.5 (148.0-184.4)
Wales	1,854 (1,557-2,190)	59.8 (50.2-70.6)	0.10 (0.09-0.11)	32.3 (29.6-35.1)	4 (3-6)	2.5 (1.9-3.3)	0.00 (0.00-0.00)	21.0 (15.9-28.1)	1,687 (1,397-2,017)	398.6 (330.1-476.6)	0.05 (0.05-0.06)	122.2 (108.9-136.9)
**Southern Latin America**	**34,021 (30,754-37,110)**	**52.1 (47.1-56.8)**	**2.54 (2.38-2.74)**	**38.9 (36.4-41.9)**	**626 (513-761)**	**12.5 (10.2-15.2)**	**0.29 (0.23-0.36)**	**57.8 (46.5-72.0)**	**26,071 (23,187-28,951)**	**516.2 (459.1-573.3)**	**0.71 (0.63-0.79)**	**140.6 (125.2-156.5)**
Argentina	27,446 (24,571-30,353)	62.8 (56.2-69.5)	1.88 (1.74-2.03)	42.9 (39.9-46.4)	523 (422-648)	14.6 (11.8-18.1)	0.23 (0.18-0.28)	63.7 (51.3-79.3)	20,581 (18,143-23,242)	623.2 (549.4-703.7)	0.50 (0.44-0.57)	152.7 (134.4-172.5)
Chile	4,976 (4,028-6,057)	27.4 (22.2-33.3)	0.51 (0.47-0.55)	28.1 (26.1-30.4)	73 (55-95)	6.1 (4.6-7.9)	0.05 (0.04-0.06)	39.9 (30.8-51.9)	4,185 (3,358-5,120)	301.6 (242.0-369.0)	0.15 (0.14-0.17)	111.5 (99.0-123.2)
Uruguay	1,600 (1,418-1,793)	46.5 (41.2-52.1)	0.16 (0.14-0.17)	45.4 (41.8-48.8)	30 (18-45)	12.5 (7.7-18.8)	0.01 (0.01-0.02)	59.6 (47.6-72.9)	1,306 (1,139-1,487)	363.1 (316.7-413.5)	0.05 (0.04-0.06)	141.9 (124.2-160.8)
**Central Europe, Eastern Europe, and Central Asia**	**97,153 (86,019-110,400)**	**23.4 (20.7-26.6)**	**27.14 (25.20-29.20)**	**65.3 (60.6-70.3)**	**17,025 (13,377-22,085)**	**60.4 (47.4-78.3)**	**3.02 (2.41-3.71)**	**107.1 (85.4-131.7)**	**31,483 (28,367-34,849)**	**85.2 (76.7-94.3)**	**6.32 (5.59-7.09)**	**171.1 (151.1-191.9)**
**Eastern Europe**	**46,126 (36,064-58,694)**	**21.8 (17.1-27.8)**	**17.18 (15.84-18.62)**	**81.2 (74.9-88.0)**	**1,791 (1,454-2,219)**	**13.8 (11.2-17.1)**	**1.46 (1.15-1.83)**	**112.6 (88.5-141.5)**	**11,363 (9,178-14,151)**	**54.8 (44.3-68.3)**	**4.21 (3.67-4.78)**	**203.0 (177.0-230.7)**
Belarus	895 (720-1,088)	9.4 (7.5-11.4)	0.70 (0.63-0.77)	73.1 (66.4-80.3)	26 (16-40)	4.5 (2.8-7.0)	0.08 (0.06-0.10)	139.6 (108.2-182.1)	261 (214-316)	27.7 (22.7-33.5)	0.16 (0.14-0.18)	170.0 (148.2-194.6)
Estonia	159 (136-188)	12.0 (10.3-14.3)	0.10 (0.09-0.11)	77.5 (71.2-85.3)	4 (2-6)	5.6 (3.5-8.1)	0.01 (0.01-0.01)	113.1 (86.2-146.6)	67 (56-78)	36.0 (30.2-42.1)	0.04 (0.03-0.04)	191.7 (167.7-220.1)
Latvia	328 (282-377)	16.6 (14.2-19.0)	0.15 (0.14-0.16)	76.0 (70.0-82.4)	6 (4-10)	6.1 (3.6-9.9)	0.01 (0.01-0.01)	108.9 (84.2-136.5)	134 (114-157)	45.5 (38.8-53.4)	0.05 (0.04-0.06)	174.2 (151.6-198.8)

Location	All Ages				Children under-5				Adults over-70			
	**Deaths (95% UI)**	**Deaths per 1000 (95% UI)**	**Millions of cases (95% UI)**	**Incidence per 1000 (95% UI)**	**Deaths (95% UI)**	**Deaths per 1000 (95% UI)**	**Millions of cases (95% UI)**	**Incidence per 1000 (95% UI)**	**Deaths (95% UI)**	**Deaths per 1000 (95% UI)**	**Millions of cases (95% UI)**	**Incidence per 1000 (95% UI)**
Lithuania	545 (487-611)	18.8 (16.8-21.1)	0.26 (0.24-0.28)	88.4 (81.7-96.0)	11 (8-14)	7.1 (5.0-9.5)	0.02 (0.01-0.02)	127.4 (98.4-159.1)	256 (222-293)	61.1 (53.1-69.9)	0.10 (0.09-0.11)	238.7 (210.3-270.4)
Moldova	858 (739-990)	21.1 (18.2-24.3)	0.33 (0.30-0.36)	81.3 (74.5-89.0)	88 (55-132)	39.1 (24.5-58.8)	0.03 (0.02-0.04)	131.9 (101.8-165.0)	120 (101-141)	42.7 (36.0-50.4)	0.06 (0.05-0.07)	212.3 (180.1-243.4)
Russia	37,613 (27,719-50,026)	25.8 (19.0-34.3)	12.46 (11.41-13.64)	85.3 (78.1-93.4)	1,473 (1,166-1,853)	15.7 (12.4-19.8)	1.04 (0.82-1.30)	110.7 (87.0-138.8)	9,090 (6,997-11,767)	68.3 (52.6-88.5)	2.97 (2.58-3.42)	223.5 (193.8-257.1)
Ukraine	5,728 (4,395-7,417)	12.6 (9.6-16.3)	3.18 (2.93-3.45)	69.8 (64.2-75.6)	183 (88-341)	7.4 (3.6-13.9)	0.27 (0.21-0.34)	111.2 (85.5-139.4)	1,436 (1,155-1,795)	27.1 (21.8-33.9)	0.83 (0.72-0.94)	155.9 (135.6-178.1)
**Central Europe**	**26,325 (24,435-28,353)**	**22.8 (21.1-24.5)**	**4.79 (4.48-5.13)**	**41.4 (38.7-44.4)**	**795 (674-939)**	**14.2 (12.1-16.8)**	**0.54 (0.43-0.67)**	**96.7 (76.9-119.7)**	**17,366 (15,787-19,073)**	**134.3 (122.1-147.5)**	**1.56 (1.42-1.73)**	**120.6 (109.6-133.4)**
Albania	408 (320-565)	14.1 (11.1-19.6)	0.18 (0.17-0.20)	63.1 (57.6-69.8)	76 (42-121)	40.9 (22.8-65.3)	0.04 (0.03-0.05)	203.6 (159.1-262.8)	250 (194-344)	101.5 (78.6-139.4)	0.04 (0.04-0.05)	178.2 (155.6-205.9)
Bosnia and Herzegovina	350 (270-551)	9.2 (7.1-14.5)	0.17 (0.16-0.19)	45.5 (41.2-49.7)	6 (4-8)	3.5 (2.4-4.7)	0.02 (0.02-0.03)	136.1 (105.2-170.8)	238 (181-384)	61.4 (46.7-99.2)	0.05 (0.04-0.06)	127.5 (108.7-146.4)
Bulgaria	1,613 (1,376-1,867)	22.3 (19.0-25.8)	0.35 (0.32-0.38)	48.5 (44.5-52.9)	76 (44-121)	22.7 (13.1-36.4)	0.04 (0.03-0.05)	121.5 (97.0-151.1)	978 (824-1,143)	100.5 (84.6-117.4)	0.12 (0.10-0.13)	121.0 (104.8-138.3)
Croatia	675 (573-780)	16.0 (13.6-18.5)	0.15 (0.14-0.16)	34.9 (32.7-37.4)	4 (3-5)	1.9 (1.4-2.5)	0.01 (0.01-0.02)	71.1 (59.0-85.3)	533 (442-626)	95.3 (79.1-111.9)	0.06 (0.06-0.07)	111.6 (101.1-125.8)
Czech Republic	2,887 (2,566-3,277)	27.2 (24.1-30.8)	0.44 (0.40-0.48)	41.4 (37.7-45.4)	15 (11-21)	2.8 (1.9-3.8)	0.04 (0.03-0.05)	67.9 (51.9-88.7)	2,159 (1,881-2,474)	171.7 (149.6-196.8)	0.17 (0.15-0.19)	136.0 (119.0-155.0)
Hungary	969 (835-1,088)	9.8 (8.4-11.0)	0.37 (0.34-0.41)	37.6 (34.3-40.9)	26 (17-38)	5.8 (3.7-8.3)	0.05 (0.04-0.07)	108.4 (81.3-145.3)	639 (550-728)	51.6 (44.5-58.9)	0.11 (0.10-0.13)	90.4 (79.5-103.1)
Macedonia	149 (122-222)	7.2 (5.9-10.7)	0.10 (0.09-0.11)	49.6 (44.9-54.9)	15 (9-23)	13.0 (7.9-20.1)	0.02 (0.01-0.02)	149.3 (116.1-188.7)	90 (69-149)	52.8 (40.4-87.6)	0.02 (0.02-0.03)	130.2 (111.7-148.8)
Montenegro	56 (47-70)	8.9 (7.5-11.2)	0.04 (0.04-0.05)	65.5 (58.9-72.9)	2 (1-3)	6.0 (3.8-9.1)	0.01 (0.01-0.01)	204.0 (155.7-262.0)	35 (28-46)	60.8 (49.1-79.9)	0.01 (0.01-0.01)	157.0 (137.4-177.6)

Location	All Ages				Children under-5				Adults over-70			
	Deaths (95% UI)	Deaths per 1000 (95% UI)	Millions of cases (95% UI)	Incidence per 1000 (95% UI)	Deaths (95% UI)	Deaths per 1000 (95% UI)	Millions of cases (95% UI)	Incidence per 1000 (95% UI)	Deaths (95% UI)	Deaths per 1000 (95% UI)	Millions of cases (95% UI)	Incidence per 1000 (95% UI)
Poland	10,252 (9,081-11,579)	26.5 (23.5-30.0)	1.33 (1.23-1.45)	34.4 (31.7-37.4)	68 (40-112)	3.6 (2.1-6.0)	0.12 (0.09-0.16)	65.4 (50.5-83.2)	7,241 (6,201-8,352)	183.0 (156.7-211.1)	0.47 (0.41-0.52)	118.0 (104.0-131.9)
Romania	5,388 (4,787-6,021)	27.8 (24.7-31.1)	1.03 (0.96-1.12)	53.4 (49.4-57.7)	446 (355-565)	50.8 (40.4-64.3)	0.12 (0.10-0.14)	133.2 (108.5-162.6)	2,626 (2,258-3,004)	113.0 (97.2-129.3)	0.30 (0.26-0.34)	129.9 (113.3-147.6)
Serbia	1,208 (968-1,391)	13.8 (11.0-15.8)	0.29 (0.27-0.32)	33.2 (30.6-36.1)	32 (24-41)	7.6 (5.7-10.0)	0.04 (0.03-0.05)	93.1 (74.0-114.3)	799 (664-973)	81.6 (67.8-99.3)	0.09 (0.08-0.10)	92.0 (79.3-103.8)
Slovakia	1,744 (1,394-2,081)	32.0 (25.6-38.1)	0.24 (0.22-0.26)	43.7 (40.5-47.5)	28 (19-42)	9.8 (6.5-14.5)	0.03 (0.02-0.04)	99.0 (78.1-122.8)	1,201 (943-1,438)	235.4 (184.8-281.9)	0.08 (0.07-0.09)	147.2 (130.7-166.8)
Slovenia	627 (533-731)	30.3 (25.8-35.4)	0.08 (0.08-0.09)	41.0 (38.0-44.8)	1 (1-2)	1.2 (0.9-1.6)	0.01 (0.01-0.01)	78.6 (59.8-102.5)	576 (484-677)	210.6 (177.2-247.7)	0.04 (0.04-0.04)	142.7 (128.1-159.8)
**Central Asia**	**24,702 (20,925-29,866)**	**28.0 (23.7-33.8)**	**5.17 (4.77-5.59)**	**58.5 (54.1-63.3)**	**14,439 (10,821-19,609)**	**149.7 (112.2-203.3)**	**1.02 (0.81-1.24)**	**105.7 (84.0-128.2)**	**2,753 (2,488-3,027)**	**82.9 (74.9-91.2)**	**0.56 (0.50-0.62)**	**168.5 (150.3-188.3)**
Armenia	424 (372-485)	13.9 (12.2-15.9)	0.19 (0.17-0.21)	62.4 (57.2-67.8)	121 (84-168)	54.8 (38.0-75.9)	0.02 (0.02-0.02)	87.2 (68.4-106.9)	191 (162-224)	83.7 (71.2-98.5)	0.05 (0.04-0.05)	206.2 (177.9-234.1)
Azerbaijan	2,713 (1,994-3,709)	27.6 (20.3-37.7)	0.60 (0.55-0.66)	61.2 (55.9-66.7)	1,754 (1,083-2,692)	183.3 (113.2-281.3)	0.12 (0.09-0.14)	120.9 (94.7-148.4)	340 (232-440)	84.4 (57.7-109.4)	0.08 (0.07-0.09)	198.5 (168.4-231.8)
Georgia	502 (428-583)	12.4 (10.6-14.4)	0.20 (0.19-0.21)	49.2 (45.9-53.1)	68 (45-100)	20.3 (13.5-30.0)	0.02 (0.02-0.03)	62.9 (49.7-77.2)	251 (209-294)	61.8 (51.4-72.3)	0.06 (0.05-0.07)	146.7 (128.8-165.4)
Kazakhstan	3,134 (2,625-3,705)	17.5 (14.7-20.7)	0.96 (0.88-1.05)	54.0 (49.5-58.7)	633 (413-925)	32.3 (21.1-47.2)	0.14 (0.11-0.17)	71.7 (55.6-88.0)	693 (571-832)	85.9 (70.8-103.1)	0.13 (0.11-0.15)	158.3 (138.1-182.6)
Kyrgyzstan	1,250 (1,028-1,497)	20.9 (17.2-25.0)	0.39 (0.35-0.42)	64.5 (58.8-70.6)	897 (682-1,136)	120.0 (91.2-152.0)	0.09 (0.07-0.11)	123.4 (99.3-148.9)	91 (79-105)	55.0 (47.8-63.2)	0.03 (0.03-0.04)	197.8 (169.6-228.2)
Mongolia	600 (453-811)	19.8 (14.9-26.7)	0.15 (0.14-0.17)	50.9 (46.2-55.9)	380 (256-536)	102.0 (68.7-143.9)	0.04 (0.03-0.05)	110.6 (87.2-134.8)	45 (36-63)	57.2 (45.7-80.5)	0.01 (0.01-0.01)	145.6 (124.7-167.3)
Tajikistan	4,221 (3,039-5,823)	49.1 (35.4-67.8)	0.61 (0.55-0.68)	71.4 (64.5-79.3)	2,718 (1,589-4,248)	238.7 (139.6-373.1)	0.14 (0.11-0.17)	119.8 (94.1-148.8)	378 (289-468)	217.7 (166.1-269.4)	0.04 (0.04-0.05)	251.8 (214.6-289.4)

	All Ages				Children under-5				Adults over-70			
**Location**	**Deaths (95% UI)**	**Deaths per 1000 (95% UI)**	**Millions of cases (95% UI)**	**Incidence per 1000 (95% UI)**	**Deaths (95% UI)**	**Deaths per 1000 (95% UI)**	**Millions of cases (95% UI)**	**Incidence per 1000 (95% UI)**	**Deaths (95% UI)**	**Deaths per 1000 (95% UI)**	**Millions of cases (95% UI)**	**Incidence per 1000 (95% UI)**
Turkmenistan	1,580 (1,127-2,179)	28.8 (20.6-39.8)	0.25 (0.23-0.28)	46.0 (41.8-50.7)	1,190 (737-1,797)	206.1 (127.6-311.1)	0.06 (0.05-0.08)	111.5 (87.2-137.3)	61 (53-71)	44.0 (37.8-50.7)	0.02 (0.01-0.02)	110.8 (97.8-127.7)
Uzbekistan	10,277 (7,087-15,016)	33.8 (23.3-49.3)	1.81 (1.66-1.97)	59.3 (54.5-64.6)	6,678 (3,599-11,576)	199.9 (107.7-346.5)	0.39 (0.31-0.48)	116.4 (91.4-144.0)	702 (603-812)	76.5 (65.7-88.5)	0.14 (0.12-0.16)	154.3 (135.2-175.5)
**Latin America and Caribbean**	**160,842 (150,906-170,365)**	**27.9 (26.2-29.5)**	**23.60 (22.05-25.23)**	**40.9 (38.2-43.8)**	**21,838 (19,982-24,428)**	**44.0 (40.3-49.2)**	**4.71 (3.82-5.70)**	**94.9 (77.0-114.8)**	**89,805 (81,557-97,556)**	**326.2 (296.2-354.3)**	**6.45 (5.91-7.05)**	**234.4 (214.6-256.0)**
**Central Latin America**	**43,191 (40,602-46,405)**	**17.0 (15.9-18.2)**	**5.30 (4.89-5.75)**	**20.8 (19.2-22.6)**	**9,162 (8,326-10,403)**	**40.1 (36.4-45.5)**	**1.32 (1.06-1.64)**	**57.8 (46.4-71.7)**	**19,219 (17,262-21,205)**	**175.3 (157.5-193.5)**	**1.22 (1.10-1.34)**	**111.2 (100.6-122.1)**
Colombia	6,045 (5,376-6,817)	12.5 (11.1-14.1)	1.17 (1.06-1.28)	24.0 (21.9-26.3)	874 (651-1,174)	24.5 (18.2-32.9)	0.25 (0.19-0.32)	70.2 (54.2-88.9)	3,119 (2,638-3,634)	143.1 (121.1-166.8)	0.28 (0.25-0.32)	128.7 (113.8-145.3)
Costa Rica	407 (357-463)	8.5 (7.4-9.6)	0.18 (0.16-0.20)	37.5 (33.9-41.9)	32 (19-51)	10.4 (6.1-16.8)	0.04 (0.03-0.04)	115.1 (87.3-147.6)	241 (200-286)	83.8 (69.6-99.4)	0.04 (0.04-0.05)	149.5 (130.6-169.5)
El Salvador	1,994 (1,419-2,372)	32.4 (23.0-38.5)	0.24 (0.22-0.26)	39.2 (36.0-42.9)	151 (98-227)	28.3 (18.3-42.4)	0.05 (0.04-0.06)	97.2 (75.6-121.5)	1,219 (783-1,534)	347.5 (223.1-437.2)	0.07 (0.06-0.08)	209.1 (182.6-236.9)
Guatemala	8,884 (7,469-10,424)	53.8 (45.2-63.1)	0.74 (0.68-0.82)	45.1 (40.9-49.9)	2,705 (2,248-3,257)	137.2 (114.0-165.2)	0.28 (0.22-0.34)	140.4 (113.2-171.9)	3,464 (2,756-4,219)	658.7 (524.2-802.4)	0.13 (0.11-0.14)	240.9 (215.5-269.9)
Honduras	879 (603-1,363)	10.6 (7.2-16.4)	0.28 (0.24-0.32)	33.4 (29.3-38.1)	328 (223-469)	34.7 (23.6-49.5)	0.11 (0.09-0.14)	117.2 (90.5-150.6)	307 (169-607)	115.6 (63.5-228.3)	0.04 (0.04-0.05)	157.8 (134.1-183.3)
Mexico	18,599 (17,374-19,952)	14.5 (13.5-15.5)	1.74 (1.61-1.88)	13.5 (12.5-14.6)	3,613 (3,239-4,170)	31.0 (27.7-35.7)	0.35 (0.28-0.43)	30.2 (23.9-37.3)	8,053 (7,141-9,019)	142.5 (126.4-159.6)	0.45 (0.41-0.49)	79.9 (72.7-87.4)
Nicaragua	831 (702-1,006)	13.5 (11.4-16.3)	0.12 (0.10-0.13)	18.7 (16.8-21.1)	309 (238-409)	50.8 (39.0-67.1)	0.04 (0.03-0.05)	61.7 (47.2-78.2)	291 (224-382)	132.8 (102.3-174.2)	0.02 (0.02-0.02)	94.1 (82.8-106.0)
Panama	787 (686-907)	19.8 (17.3-22.9)	0.16 (0.15-0.18)	40.8 (36.8-45.5)	154 (102-235)	44.4 (29.4-67.7)	0.04 (0.03-0.05)	124.8 (98.5-155.8)	415 (352-496)	200.0 (169.3-238.6)	0.04 (0.04-0.05)	198.3 (174.2-224.0)
Venezuela	4,765 (4,161-5,498)	15.2 (13.2-17.5)	0.67 (0.61-0.73)	21.2 (19.3-23.2)	995 (799-1,242)	34.5 (27.7-43.0)	0.16 (0.13-0.20)	56.3 (44.1-70.2)	2,110 (1,756-2,552)	165.6 (137.9-200.3)	0.14 (0.12-0.16)	110.1 (97.1-122.3)

Location	All Ages				Children under-5				Adults over-70			
	Deaths (95% UI)	Deaths per 1000 (95% UI)	Millions of cases (95% UI)	Incidence per 1000 (95% UI)	Deaths (95% UI)	Deaths per 1000 (95% UI)	Millions of cases (95% UI)	Incidence per 1000 (95% UI)	Deaths (95% UI)	Deaths per 1000 (95% UI)	Millions of cases (95% UI)	Incidence per 1000 (95% UI)
**Andean Latin America**	**28,653 (24,397-32,571)**	**47.9 (40.7-54.4)**	**3.76 (3.50-4.03)**	**62.8 (58.4-67.4)**	**4,813 (3,939-5,793)**	**72.2 (59.1-86.9)**	**0.77 (0.62-0.93)**	**115.5 (93.8-140.1)**	**17,032 (13,894-19,794)**	**636.5 (519.2-739.7)**	**1.09 (0.98-1.19)**	**406.5 (365.1-446.1)**
Bolivia	6,678 (5,374-8,272)	60.3 (48.6-74.7)	0.80 (0.73-0.88)	72.3 (65.6-79.5)	1,962 (1,382-2,706)	143.4 (101.0-197.7)	0.19 (0.15-0.24)	142.3 (110.7-177.1)	3,377 (2,415-4,467)	695.3 (497.3-919.7)	0.22 (0.19-0.25)	455.4 (398.6-515.9)
Ecuador	4,402 (3,954-4,854)	26.6 (23.9-29.3)	0.63 (0.58-0.67)	37.8 (35.1-40.7)	930 (698-1,232)	52.0 (39.0-68.9)	0.14 (0.11-0.17)	77.5 (62.5-93.9)	2,301 (1,996-2,652)	308.7 (267.8-355.7)	0.17 (0.15-0.19)	225.0 (204.0-249.0)
Peru	17,572 (13,521-21,033)	54.5 (41.9-65.2)	2.33 (2.17-2.51)	72.3 (67.2-77.7)	1,921 (1,393-2,562)	54.8 (39.7-73.1)	0.44 (0.35-0.52)	124.4 (101.3-149.5)	11,354 (8,450-13,754)	785.8 (584.8-951.9)	0.70 (0.62-0.77)	483.8 (431.2-536.1)
**Caribbean**	**16,895 (15,166-18,887)**	**36.9 (33.2-41.3)**	**2.81 (2.60-3.03)**	**61.4 (56.9-66.3)**	**3,128 (2,046-4,702)**	**78.4 (51.3-117.9)**	**0.60 (0.48-0.74)**	**150.1 (119.3-185.9)**	**9,871 (8,822-10,866)**	**344.3 (307.6-378.9)**	**0.83 (0.74-0.92)**	**288.9 (258.3-320.1)**
Antigua and Barbuda	25 (22-28)	27.3 (24.0-31.1)	0.01 (0.00-0.01)	58.2 (53.2-63.7)	2 (1-2)	25.6 (14.9-40.0)	0.00 (0.00-0.00)	133.1 (103.6-165.1)	16 (13-18)	333.1 (281.9-390.2)	0.00 (0.00-0.00)	286.2 (251.7-325.7)
The Bahamas	125 (109-144)	31.5 (27.4-36.3)	0.02 (0.02-0.03)	59.1 (54.0-64.3)	8 (4-18)	25.1 (11.0-53.4)	0.00 (0.00-0.01)	127.5 (97.0-162.0)	73 (61-86)	344.5 (289.6-406.5)	0.01 (0.01-0.01)	274.1 (241.4-309.9)
Barbados	171 (151-192)	60.4 (53.3-67.9)	0.03 (0.03-0.03)	101.1 (92.7-110.2)	3 (2-7)	23.2 (10.3-45.1)	0.00 (0.00-0.00)	179.1 (137.9-228.2)	135 (117-154)	486.7 (422.1-558.5)	0.01 (0.01-0.01)	423.5 (376.7-474.2)
Belize	109 (93-128)	29.2 (24.9-34.3)	0.02 (0.02-0.02)	54.7 (49.4-60.5)	15 (7-31)	32.5 (14.0-64.9)	0.01 (0.00-0.01)	116.6 (91.4-145.3)	51 (44-59)	612.8 (521.0-707.4)	0.00 (0.00-0.00)	336.3 (295.8-382.7)
Bermuda	14 (12-17)	20.2 (16.9-23.7)	0.00 (0.00-0.00)	57.6 (52.4-63.0)	0 (0-0)	4.9 (2.8-8.1)	0.00 (0.00-0.00)	120.3 (93.3-152.3)	11 (9-14)	259.3 (212.5-311.4)	0.00 (0.00-0.00)	286.6 (252.2-323.2)
Cuba	5,697 (5,072-6,399)	49.9 (44.5-56.1)	0.71 (0.65-0.77)	62.1 (56.6-67.5)	60 (46-74)	10.1 (7.7-12.4)	0.05 (0.04-0.06)	78.2 (62.4-96.4)	4,677 (4,096-5,342)	410.1 (359.2-468.4)	0.34 (0.30-0.39)	300.5 (264.4-339.1)
Dominica	26 (22-30)	34.8 (30.3-40.1)	0.00 (0.00-0.00)	57.8 (52.7-63.1)	2 (1-3)	36.4 (21.0-59.4)	0.00 (0.00-0.00)	114.8 (89.3-146.0)	17 (15-20)	379.8 (321.7-447.8)	0.00 (0.00-0.00)	276.4 (241.7-310.0)
Dominican Republic	1,979 (1,473-2,362)	18.8 (14.0-22.4)	0.55 (0.50-0.60)	52.0 (47.3-57.0)	466 (302-713)	49.5 (32.1-75.7)	0.14 (0.11-0.17)	146.0 (116.6-176.4)	1,036 (742-1,270)	216.1 (154.9-265.0)	0.12 (0.10-0.13)	244.0 (211.9-276.2)

Location	All Ages				Children under-5				Adults over-70			
	**Deaths (95% UI)**	**Deaths per 1000 (95% UI)**	**Millions of cases (95% UI)**	**Incidence per 1000 (95% UI)**	**Deaths (95% UI)**	**Deaths per 1000 (95% UI)**	**Millions of cases (95% UI)**	**Incidence per 1000 (95% UI)**	**Deaths (95% UI)**	**Deaths per 1000 (95% UI)**	**Millions of cases (95% UI)**	**Incidence per 1000 (95% UI)**
Grenada	61 (53-69)	57.2 (50.3-64.6)	0.01 (0.01-0.01)	69.8 (64.1-76.1)	3 (2-6)	36.0 (18.6-66.3)	0.00 (0.00-0.00)	137.3 (106.5-171.6)	39 (34-45)	732.6 (629.2-838.6)	0.00 (0.00-0.00)	377.6 (336.5-424.9)
Guyana	236 (205-268)	30.9 (26.8-35.0)	0.03 (0.03-0.04)	45.1 (41.6-49.1)	23 (16-31)	34.4 (23.9-46.8)	0.01 (0.00-0.01)	86.1 (68.4-104.9)	92 (77-107)	421.9 (353.9-490.0)	0.01 (0.00-0.01)	242.6 (210.5-275.5)
Haiti	5,421 (4,149-7,076)	48.7 (37.3-63.6)	0.74 (0.66-0.83)	66.4 (59.3-74.7)	2,415 (1,347-3,989)	159.0 (88.6-262.5)	0.30 (0.24-0.38)	198.1 (155.7-247.6)	1,572 (1,213-1,994)	484.7 (374.0-614.9)	0.09 (0.08-0.11)	281.0 (243.2-326.8)
Jamaica	707 (569-914)	24.6 (19.8-31.8)	0.16 (0.14-0.17)	54.2 (49.5-59.4)	68 (34-115)	24.4 (12.3-41.5)	0.03 (0.03-0.04)	122.9 (95.6-156.6)	490 (388-645)	272.4 (215.7-358.1)	0.05 (0.04-0.05)	257.3 (224.9-291.1)
Puerto Rico	1,716 (1,483-1,990)	46.7 (40.4-54.2)	0.29 (0.26-0.32)	78.5 (71.7-85.9)	15 (11-21)	7.4 (5.4-9.8)	0.02 (0.02-0.03)	111.2 (82.3-144.7)	1,302 (1,096-1,536)	351.2 (295.6-414.4)	0.13 (0.11-0.14)	341.5 (300.5-383.3)
Saint Lucia	46 (41-51)	25.3 (22.5-28.1)	0.01 (0.01-0.01)	66.2 (60.9-72.2)	2 (1-4)	21.4 (9.8-41.0)	0.00 (0.00-0.00)	143.2 (108.7-189.1)	29 (25-33)	248.9 (213.3-284.2)	0.00 (0.00-0.00)	306.6 (268.3-347.2)
Saint Vincent and the Grenadines	39 (35-44)	35.6 (31.8-40.3)	0.01 (0.01-0.01)	57.9 (53.1-63.2)	2 (1-4)	26.8 (15.3-46.1)	0.00 (0.00-0.00)	112.5 (86.0-141.8)	24 (21-28)	444.0 (386.6-514.0)	0.00 (0.00-0.00)	299.1 (265.4-341.8)
Suriname	183 (152-212)	33.4 (27.8-38.9)	0.03 (0.02-0.03)	48.1 (44.1-52.5)	26 (14-45)	56.1 (30.8-97.2)	0.00 (0.00-0.01)	103.8 (79.8-131.1)	102 (81-122)	412.6 (328.5-492.0)	0.01 (0.01-0.01)	249.5 (220.7-281.7)
Trinidad and Tobago	301 (266-341)	22.5 (19.8-25.4)	0.08 (0.07-0.08)	56.1 (51.2-60.8)	15 (5-34)	21.8 (7.6-49.3)	0.01 (0.01-0.01)	137.5 (104.7-176.2)	175 (151-202)	213.8 (183.8-245.9)	0.02 (0.02-0.02)	236.0 (206.7-268.2)
Virgin Islands, U.S.	39 (32-47)	37.2 (30.6-44.7)	0.01 (0.01-0.01)	74.2 (67.6-81.3)	0 (0-1)	6.6 (4.4-9.5)	0.00 (0.00-0.00)	121.8 (91.3-160.0)	30 (24-36)	238.0 (194.8-289.8)	0.00 (0.00-0.00)	257.2 (223.8-291.7)
**Tropical Latin America**	**71,338 (66,753-75,856)**	**33.0 (30.8-35.0)**	**11.74 (10.99-12.54)**	**54.2 (50.7-57.9)**	**4,639 (4,197-5,173)**	**28.8 (26.1-32.1)**	**2.02 (1.64-2.44)**	**125.3 (101.8-151.5)**	**43,198 (39,301-46,840)**	**391.7 (356.3-424.7)**	**3.32 (3.03-3.61)**	**301.0 (274.8-327.7)**
Brazil	69,962 (65,395-74,351)	33.3 (31.2-35.4)	11.10 (10.39-11.85)	52.9 (49.5-56.5)	4,461 (4,018-4,966)	28.8 (26.0-32.1)	1.88 (1.53-2.26)	121.5 (99.0-146.3)	42,435 (38,545-46,019)	394.2 (358.1-427.5)	3.21 (2.93-3.50)	298.2 (272.4-325.1)
Paraguay	1,376 (1,148-1,652)	20.6 (17.2-24.7)	0.64 (0.58-0.72)	96.5 (86.8-107.4)	178 (119-256)	28.3 (18.9-40.6)	0.14 (0.11-0.18)	219.6 (170.6-279.3)	762 (599-942)	288.5 (226.8-356.7)	0.11 (0.10-0.13)	417.2 (361.8-475.3)

Location	All Ages				Children under-5				Adults over-70			
	Deaths (95% UI)	Deaths per 1000 (95% UI)	Millions of cases (95% UI)	Incidence per 1000 (95% UI)	Deaths (95% UI)	Deaths per 1000 (95% UI)	Millions of cases (95% UI)	Incidence per 1000 (95% UI)	Deaths (95% UI)	Deaths per 1000 (95% UI)	Millions of cases (95% UI)	Incidence per 1000 (95% UI)
**Southeast Asia, East Asia, and Oceania**	**380,060 (328,336-412,923)**	**18.2 (15.8-19.8)**	**75.11 (69.18-81.50)**	**36.1 (33.2-39.1)**	**60,156 (53,483-67,970)**	**48.9 (43.5-55.2)**	**14.82 (11.75-18.29)**	**120.4 (95.5-148.6)**	**214,284 (179,802-237,500)**	**190.3 (159.6-210.9)**	**14.73 (13.26-16.29)**	**130.8 (117.7-144.6)**
**East Asia**	**171,297 (148,361-203,061)**	**12.1 (10.5-14.3)**	**44.22 (40.74-48.08)**	**31.2 (28.7-33.9)**	**17,022 (14,554-19,437)**	**26.3 (22.5-30.1)**	**6.94 (5.43-8.64)**	**107.4 (84.0-133.6)**	**115,743 (97,715-137,327)**	**132.3 (111.7-157.0)**	**10.15 (9.15-11.23)**	**116.0 (104.7-128.4)**
China	156,620 (138,086-189,571)	11.5 (10.1-13.9)	42.33 (39.03-46.04)	31.0 (28.5-33.7)	14,847 (12,657-17,040)	24.5 (20.9-28.1)	6.46 (5.07-8.02)	106.5 (83.6-132.3)	106,026 (91,066-129,136)	126.4 (108.6-154.0)	9.70 (8.76-10.72)	115.6 (104.4-127.8)
North Korea	5,769 (4,484-7,912)	21.8 (16.9-29.9)	1.22 (1.08-1.38)	46.1 (40.9-52.2)	2,148 (1,181-3,544)	72.6 (39.9-119.8)	0.44 (0.33-0.56)	147.2 (112.1-188.5)	2,453 (1,778-3,582)	156.9 (113.7-229.2)	0.20 (0.17-0.24)	129.2 (110.1-151.2)
Taiwan	8,908 (4,346-11,470)	37.5 (18.3-48.3)	0.68 (0.61-0.75)	28.5 (25.8-31.5)	27 (20-36)	2.7 (1.9-3.5)	0.04 (0.03-0.06)	44.2 (33.4-57.6)	7,264 (3,528-9,407)	358.4 (174.1-464.1)	0.25 (0.21-0.29)	123.3 (105.9-142.6)
**Southeast Asia**	**201,790 (170,920-218,582)**	**30.8 (26.1-33.4)**	**30.04 (27.56-32.83)**	**45.9 (42.1-50.1)**	**40,181 (34,696-46,626)**	**70.5 (60.8-81.8)**	**7.63 (6.10-9.41)**	**133.9 (107.0-165.0)**	**97,141 (80,361-107,598)**	**389.8 (322.4-431.7)**	**4.51 (4.02-5.00)**	**181.0 (161.5-200.4)**
Cambodia	9,858 (8,480-11,429)	61.8 (53.2-71.7)	0.98 (0.90-1.07)	61.3 (56.3-66.9)	3,974 (3,025-5,018)	211.0 (160.6-266.4)	0.32 (0.27-0.38)	172.4 (142.6-203.6)	2,702 (2,191-3,338)	697.9 (566.1-862.4)	0.10 (0.09-0.11)	253.0 (220.2-290.2)
Indonesia	38,140 (33,582-42,030)	14.8 (13.0-16.3)	11.39 (10.22-12.79)	44.2 (39.6-49.6)	12,802 (11,046-15,168)	56.3 (48.6-66.7)	4.01 (3.17-5.01)	176.5 (139.3-220.4)	12,974 (10,582-14,966)	158.3 (129.1-182.6)	1.08 (0.95-1.21)	131.6 (116.2-147.4)
Laos	5,125 (3,118-8,041)	71.1 (43.2-111.5)	0.29 (0.25-0.32)	39.7 (34.7-44.8)	3,859 (1,829-6,835)	341.4 (161.8-604.7)	0.12 (0.09-0.15)	105.2 (81.1-132.4)	590 (471-767)	370.3 (295.7-481.4)	0.02 (0.02-0.03)	143.7 (122.9-164.0)
Malaysia	14,522 (8,746-17,536)	47.2 (28.4-57.0)	1.55 (1.42-1.68)	50.3 (46.1-54.8)	244 (173-341)	9.5 (6.8-13.3)	0.20 (0.16-0.25)	79.8 (61.1-99.3)	8,096 (5,105-10,027)	752.6 (474.6-932.1)	0.29 (0.25-0.33)	269.9 (235.8-309.7)
Maldives	21 (16-26)	5.8 (4.5-7.2)	0.01 (0.01-0.02)	38.8 (34.4-43.5)	2 (1-2)	5.6 (3.6-7.9)	0.00 (0.00-0.00)	91.1 (69.1-117.7)	14 (10-18)	111.0 (81.7-144.0)	0.00 (0.00-0.00)	164.9 (141.4-191.8)
Mauritius	257 (221-294)	20.2 (17.4-23.1)	0.05 (0.04-0.05)	38.5 (35.3-42.0)	11 (8-15)	17.3 (12.8-23.1)	0.01 (0.00-0.01)	77.3 (60.9-96.4)	148 (124-175)	196.8 (165.0-232.0)	0.01 (0.01-0.01)	132.9 (115.8-150.4)
Myanmar	15,057 (11,972-19,387)	27.6 (22.0-35.6)	1.75 (1.59-1.93)	32.1 (29.2-35.5)	5,882 (3,355-10,007)	126.3 (72.0-214.9)	0.45 (0.35-0.57)	96.5 (74.4-121.9)	3,881 (2,978-5,767)	218.6 (167.7-324.9)	0.21 (0.18-0.23)	115.5 (98.9-132.3)

Location	All Ages				Children under-5				Adults over-70			
	Deaths (95% UI)	Deaths per 1000 (95% UI)	Millions of cases (95% UI)	Incidence per 1000 (95% UI)	Deaths (95% UI)	Deaths per 1000 (95% UI)	Millions of cases (95% UI)	Incidence per 1000 (95% UI)	Deaths (95% UI)	Deaths per 1000 (95% UI)	Millions of cases (95% UI)	Incidence per 1000 (95% UI)
Philippines	60,747 (54,003-67,853)	59.3 (52.7-66.2)	5.13 (4.74-5.57)	50.1 (46.2-54.4)	9,709 (7,738-11,930)	83.9 (66.9-103.1)	1.25 (1.01-1.52)	108.5 (87.5-131.6)	32,839 (28,352-37,644)	1,188.5 (1,026.1-1,362.4)	0.73 (0.65-0.81)	264.7 (235.4-294.9)
Sri Lanka	4,155 (3,236-5,570)	20.1 (15.6-26.9)	1.15 (1.06-1.26)	55.7 (51.2-61.0)	112 (66-182)	7.4 (4.4-12.1)	0.15 (0.11-0.18)	98.2 (75.6-122.0)	2,194 (1,568-3,490)	186.6 (133.4-296.9)	0.23 (0.20-0.27)	199.4 (172.8-228.2)
Seychelles	60 (49-69)	61.3 (50.3-71.5)	0.01 (0.01-0.01)	57.8 (53.3-63.0)	2 (2-3)	29.5 (22.5-38.1)	0.00 (0.00-0.00)	109.4 (86.0-134.8)	38 (30-45)	805.3 (641.4-943.5)	0.00 (0.00-0.00)	258.5 (229.2-292.2)
Thailand	34,760 (17,858-42,966)	51.5 (26.5-63.7)	3.92 (3.57-4.28)	58.1 (53.0-63.4)	331 (215-475)	10.7 (6.9-15.4)	0.29 (0.22-0.36)	92.9 (72.3-117.2)	22,848 (10,889-29,264)	481.3 (229.4-616.4)	1.11 (0.97-1.27)	234.8 (203.4-266.6)
Timor-Leste	395 (283-556)	34.1 (24.5-47.9)	0.05 (0.04-0.06)	43.7 (38.5-49.7)	211 (115-366)	127.7 (69.7-221.1)	0.02 (0.01-0.02)	110.7 (83.9-141.2)	97 (70-138)	278.0 (201.4-395.6)	0.01 (0.00-0.01)	154.5 (131.0-182.8)
Vietnam	18,694 (15,931-23,274)	19.8 (16.9-24.7)	3.72 (3.38-4.08)	39.4 (35.9-43.3)	3,041 (2,097-4,233)	40.3 (27.8-56.0)	0.80 (0.63-0.99)	105.7 (84.0-131.7)	10,719 (8,638-14,882)	240.7 (194.0-334.2)	0.71 (0.62-0.80)	158.6 (138.5-179.7)
**Oceania**	**6,551 (4,941-8,959)**	**58.5 (44.1-79.9)**	**0.85 (0.77-0.94)**	**75.8 (68.3-83.8)**	**2,907 (1,640-4,925)**	**205.7 (116.1-348.6)**	**0.24 (0.19-0.31)**	**171.5 (132.2-217.9)**	**1,129 (887-1,467)**	**452.9 (355.9-588.5)**	**0.07 (0.06-0.08)**	**295.0 (252.2-337.3)**
American Samoa	13 (10-16)	15.8 (12.9-19.6)	0.01 (0.00-0.01)	65.5 (58.6-73.3)	1 (1-2)	16.3 (11.3-22.8)	0.00 (0.00-0.00)	116.3 (92.1-144.4)	4 (3-5)	251.7 (194.4-328.2)	0.00 (0.00-0.00)	255.6 (218.9-296.0)
Federated States of Micronesia	37 (26-51)	36.4 (25.7-50.0)	0.01 (0.01-0.01)	72.9 (66.1-80.7)	4 (2-8)	45.1 (22.5-78.4)	0.00 (0.00-0.00)	149.4 (114.8-190.1)	13 (9-17)	488.8 (334.8-654.9)	0.00 (0.00-0.00)	314.8 (268.6-369.5)
Fiji	320 (241-426)	37.1 (27.9-49.4)	0.07 (0.06-0.07)	76.7 (69.9-84.0)	60 (32-101)	117.8 (63.9-198.7)	0.01 (0.01-0.01)	191.9 (146.8-247.3)	93 (66-132)	301.7 (214.1-427.9)	0.01 (0.01-0.01)	275.6 (234.6-322.0)
Guam	54 (45-66)	31.0 (25.9-37.7)	0.01 (0.01-0.02)	81.4 (74.4-89.0)	6 (4-8)	33.0 (21.9-46.8)	0.00 (0.00-0.00)	139.6 (108.9-175.7)	27 (22-35)	286.6 (229.6-365.0)	0.00 (0.00-0.00)	293.2 (251.2-336.6)
Kiribati	45 (34-59)	40.0 (30.1-52.3)	0.01 (0.01-0.01)	75.4 (67.7-84.1)	13 (6-25)	98.6 (45.4-185.7)	0.00 (0.00-0.00)	180.1 (138.3-233.6)	10 (8-14)	407.4 (296.6-528.3)	0.00 (0.00-0.00)	279.1 (234.3-329.1)
Marshall Islands	21 (16-29)	28.7 (21.1-39.0)	0.01 (0.01-0.01)	76.3 (68.1-86.0)	6 (3-12)	63.1 (28.9-118.0)	0.00 (0.00-0.00)	179.4 (137.1-229.2)	4 (3-6)	298.2 (213.0-410.1)	0.00 (0.00-0.00)	269.3 (228.6-316.6)

Location	All Ages				Children under-5				Adults over-70			
	Deaths (95% UI)	Deaths per 1000 (95% UI)	Millions of cases (95% UI)	Incidence per 1000 (95% UI)	Deaths (95% UI)	Deaths per 1000 (95% UI)	Millions of cases (95% UI)	Incidence per 1000 (95% UI)	Deaths (95% UI)	Deaths per 1000 (95% UI)	Millions of cases (95% UI)	Incidence per 1000 (95% UI)
Northern Mariana Islands	10 (8-13)	8.2 (6.5-10.4)	0.01 (0.01-0.01)	61.5 (54.9-68.9)	1 (0-1)	4.0 (1.9-7.5)	0.00 (0.00-0.00)	140.8 (107.6-183.7)	2 (2-3)	277.3 (217.6-354.5)	0.00 (0.00-0.00)	289.2 (248.3-334.2)
Papua New Guinea	5,560 (3,988-7,839)	70.8 (50.8-99.8)	0.59 (0.52-0.65)	74.6 (66.6-83.3)	2,694 (1,434-4,691)	250.0 (133.1-435.4)	0.19 (0.14-0.24)	173.1 (132.6-219.4)	825 (593-1,110)	627.5 (450.9-844.7)	0.04 (0.03-0.04)	290.8 (244.9-336.2)
Samoa	65 (48-91)	32.6 (24.4-45.8)	0.02 (0.02-0.02)	86.5 (77.4-97.1)	6 (2-12)	20.2 (8.5-43.1)	0.00 (0.00-0.01)	169.6 (128.8-222.3)	32 (23-48)	467.1 (334.9-693.8)	0.00 (0.00-0.00)	333.4 (284.0-386.2)
Solomon Islands	247 (189-314)	41.3 (31.6-52.6)	0.05 (0.04-0.05)	76.6 (69.2-86.0)	64 (36-105)	76.4 (42.7-125.9)	0.01 (0.01-0.02)	163.2 (126.9-205.7)	63 (48-80)	518.6 (391.6-662.2)	0.00 (0.00-0.00)	319.3 (270.7-368.5)
Tonga	39 (32-47)	36.1 (29.5-43.3)	0.01 (0.01-0.01)	83.5 (74.6-93.3)	5 (3-10)	38.9 (19.3-72.0)	0.00 (0.00-0.00)	166.1 (124.7-219.0)	21 (16-26)	479.7 (368.8-591.0)	0.00 (0.00-0.00)	345.6 (295.1-403.5)
Vanuatu	139 (109-182)	50.3 (39.4-65.8)	0.02 (0.02-0.02)	74.2 (66.2-83.6)	48 (26-83)	118.2 (64.1-205.3)	0.01 (0.00-0.01)	157.0 (119.5-201.1)	34 (27-44)	474.6 (371.4-610.7)	0.00 (0.00-0.00)	283.6 (236.1-332.9)
**North Africa and Middle East**	**106,326 (93,766-120,871)**	**18.5 (16.3-21.0)**	**32.49 (29.41-35.79)**	**56.5 (51.2-62.3)**	**39,687 (30,652-50,193)**	**62.8 (48.5-79.4)**	**8.42 (6.67-10.43)**	**133.2 (105.6-165.1)**	**33,118 (28,155-40,078)**	**187.8 (159.6-227.2)**	**4.35 (3.81-4.89)**	**246.6 (216.3-277.4)**
Afghanistan	20,137 (14,336-27,229)	60.3 (42.9-81.5)	2.63 (2.31-2.97)	78.7 (69.1-88.9)	14,608 (8,711-21,802)	294.7 (175.7-439.8)	1.12 (0.88-1.38)	225.4 (177.9-279.3)	1,620 (1,183-2,039)	357.9 (261.3-450.6)	0.13 (0.11-0.16)	291.7 (252.4-342.7)
Algeria	6,584 (4,542-8,457)	16.3 (11.3-21.0)	2.31 (2.09-2.55)	57.4 (51.9-63.3)	705 (362-1,240)	15.5 (8.0-27.3)	0.50 (0.39-0.63)	110.0 (85.0-139.1)	3,877 (2,421-5,360)	242.0 (151.2-334.6)	0.45 (0.38-0.53)	281.4 (238.7-331.4)
Bahrain	88 (67-109)	6.3 (4.8-7.8)	0.06 (0.05-0.06)	41.4 (37.5-45.7)	9 (7-13)	9.4 (6.8-13.0)	0.01 (0.01-0.01)	87.0 (67.8-110.0)	35 (27-45)	174.1 (133.4-221.7)	0.01 (0.00-0.01)	254.2 (218.2-295.1)
Egypt	21,515 (16,929-27,848)	23.5 (18.5-30.4)	6.21 (5.57-6.93)	67.7 (60.8-75.5)	7,567 (4,744-11,422)	69.4 (43.5-104.7)	1.66 (1.29-2.05)	152.3 (118.6-187.8)	4,917 (3,032-9,553)	173.3 (106.8-336.7)	0.68 (0.57-0.80)	240.1 (201.8-280.2)
Iran	8,676 (6,870-10,869)	10.7 (8.4-13.4)	4.08 (3.68-4.51)	50.1 (45.3-55.5)	1,383 (539-3,038)	17.1 (6.7-37.7)	0.86 (0.65-1.12)	106.2 (81.0-138.4)	4,237 (3,365-5,346)	164.7 (130.8-207.8)	0.62 (0.54-0.71)	240.6 (208.5-274.7)
Iraq	6,958 (4,633-9,604)	17.7 (11.8-24.4)	2.52 (2.22-2.85)	63.9 (56.4-72.4)	4,004 (1,772-6,588)	52.0 (23.0-85.5)	1.09 (0.85-1.37)	142.1 (110.9-177.5)	924 (633-1,527)	127.9 (87.7-211.6)	0.16 (0.14-0.19)	225.5 (192.0-259.5)

Location	All Ages				Children under-5				Adults over-70			
	Deaths (95% UI)	Deaths per 1000 (95% UI)	Millions of cases (95% UI)	Incidence per 1000 (95% UI)	Deaths (95% UI)	Deaths per 1000 (95% UI)	Millions of cases (95% UI)	Incidence per 1000 (95% UI)	Deaths (95% UI)	Deaths per 1000 (95% UI)	Millions of cases (95% UI)	Incidence per 1000 (95% UI)
Jordan	846 (682-1,031)	11.0 (8.8-13.4)	0.44 (0.39-0.49)	57.0 (51.1-63.1)	273 (198-368)	28.5 (20.6-38.3)	0.12 (0.09-0.15)	124.9 (97.5-154.9)	307 (236-399)	162.3 (124.5-211.0)	0.05 (0.04-0.06)	258.5 (223.1-302.7)
Kuwait	342 (270-427)	8.7 (6.8-10.9)	0.18 (0.16-0.20)	45.1 (40.9-49.8)	32 (21-47)	11.3 (7.3-16.5)	0.03 (0.02-0.03)	90.5 (70.3-114.6)	161 (123-205)	382.9 (293.3-486.6)	0.02 (0.01-0.02)	370.1 (321.5-422.9)
Lebanon	358 (259-487)	6.1 (4.4-8.4)	0.36 (0.32-0.40)	61.6 (55.3-68.5)	19 (9-33)	5.9 (2.9-10.1)	0.04 (0.03-0.05)	128.0 (97.6-164.6)	254 (169-356)	75.9 (50.6-106.4)	0.08 (0.07-0.10)	247.4 (208.6-287.7)
Libya	763 (516-996)	12.4 (8.4-16.2)	0.42 (0.37-0.46)	67.6 (60.6-75.1)	42 (19-75)	9.1 (4.1-16.2)	0.06 (0.05-0.08)	133.8 (100.6-175.8)	421 (278-569)	229.2 (151.5-309.6)	0.06 (0.05-0.07)	347.5 (297.4-405.1)
Morocco	6,202 (4,345-7,802)	18.4 (12.9-23.2)	1.66 (1.52-1.82)	49.4 (45.0-54.0)	752 (467-1,150)	32.8 (20.4-50.2)	0.21 (0.16-0.27)	91.6 (69.7-117.5)	3,463 (2,232-4,715)	243.3 (156.8-331.2)	0.36 (0.31-0.42)	256.3 (220.5-295.3)
Palestine	803 (639-1,159)	15.6 (12.4-22.4)	0.38 (0.34-0.43)	74.2 (65.6-83.7)	149 (73-260)	13.7 (6.7-23.9)	0.15 (0.11-0.18)	135.2 (105.4-169.6)	338 (239-578)	401.5 (283.5-686.1)	0.03 (0.02-0.03)	337.5 (293.2-384.0)
Oman	474 (372-565)	10.1 (7.9-12.0)	0.26 (0.23-0.29)	55.4 (49.8-61.1)	36 (24-52)	8.2 (5.4-11.8)	0.04 (0.03-0.05)	95.6 (73.6-120.2)	198 (148-248)	266.3 (198.8-332.8)	0.03 (0.03-0.03)	402.3 (350.0-456.2)
Qatar	58 (41-77)	2.5 (1.8-3.4)	0.06 (0.06-0.07)	27.2 (24.3-30.2)	7 (4-11)	5.7 (3.2-9.2)	0.01 (0.01-0.01)	74.7 (56.5-95.4)	12 (8-18)	71.0 (46.5-100.9)	0.00 (0.00-0.00)	164.8 (141.3-188.6)
Saudi Arabia	4,611 (2,934-5,646)	14.6 (9.3-17.9)	1.52 (1.40-1.66)	48.3 (44.3-52.6)	43 (28-64)	1.7 (1.1-2.6)	0.12 (0.09-0.16)	48.5 (36.3-64.2)	2,755 (1,712-3,476)	487.2 (302.8-614.7)	0.27 (0.24-0.30)	477.1 (419.8-534.5)
Sudan	11,155 (8,021-16,070)	28.4 (20.4-40.9)	1.91 (1.71-2.13)	48.6 (43.5-54.1)	5,912 (2,885-10,870)	141.0 (68.8-259.2)	0.58 (0.45-0.73)	138.5 (108.1-174.6)	2,565 (1,604-3,643)	312.9 (195.7-444.4)	0.20 (0.16-0.23)	238.6 (200.5-279.1)
Syria	1,515 (1,158-1,871)	8.3 (6.4-10.3)	1.00 (0.89-1.12)	55.0 (48.8-61.8)	326 (179-581)	18.4 (10.1-32.9)	0.24 (0.18-0.30)	133.6 (102.2-168.3)	504 (383-759)	105.7 (80.3-159.2)	0.11 (0.09-0.12)	223.0 (188.4-257.3)
Tunisia	2,247 (1,408-3,158)	20.0 (12.6-28.2)	0.80 (0.73-0.88)	71.1 (64.9-78.1)	74 (44-116)	8.9 (5.3-13.9)	0.11 (0.08-0.14)	131.9 (101.6-169.7)	1,551 (925-2,247)	269.8 (160.9-390.9)	0.20 (0.17-0.23)	345.0 (296.2-403.0)
Turkey	6,311 (5,118-8,102)	8.0 (6.5-10.2)	3.29 (3.01-3.57)	41.5 (38.0-45.0)	763 (435-1,258)	12.4 (7.1-20.4)	0.47 (0.36-0.59)	76.1 (58.4-96.0)	3,487 (2,609-4,979)	83.7 (62.6-119.5)	0.75 (0.65-0.84)	178.9 (155.3-202.1)

	All Ages				Children under-5				Adults over-70			
**Location**	**Deaths (95% UI)**	**Deaths per 1000 (95% UI)**	**Millions of cases (95% UI)**	**Incidence per 1000 (95% UI)**	**Deaths (95% UI)**	**Deaths per 1000 (95% UI)**	**Millions of cases (95% UI)**	**Incidence per 1000 (95% UI)**	**Deaths (95% UI)**	**Deaths per 1000 (95% UI)**	**Millions of cases (95% UI)**	**Incidence per 1000 (95% UI)**
United Arab Emirates	448 (331-618)	4.7 (3.4-6.4)	0.27 (0.24-0.30)	27.9 (24.8-30.9)	23 (10-47)	2.9 (1.3-5.7)	0.06 (0.04-0.07)	68.4 (51.9-87.5)	118 (55-166)	232.6 (108.0-327.7)	0.01 (0.01-0.01)	214.5 (181.1-248.0)
Yemen	6,234 (4,267-8,929)	22.2 (15.2-31.7)	2.11 (1.87-2.39)	74.8 (66.5-85.0)	2,958 (1,091-5,607)	63.9 (23.6-121.2)	0.94 (0.75-1.19)	204.2 (161.8-257.5)	1,372 (895-1,967)	326.2 (212.7-467.4)	0.13 (0.12-0.15)	318.5 (273.7-366.6)
**South Asia**	**589,653 (496,203-642,836)**	**34.7 (29.2-37.8)**	**82.97 (78.21-88.05)**	**48.8 (46.0-51.8)**	**199,513 (175,850-223,300)**	**129.9 (114.5-145.4)**	**18.76 (15.58-22.04)**	**122.1 (101.4-143.5)**	**236,883 (173,779-269,243)**	**408.8 (299.9-464.6)**	**13.37 (12.46-14.35)**	**230.7 (214.9-247.7)**
Bangladesh	33,611 (28,207-39,898)	20.8 (17.4-24.6)	5.23 (4.73-5.76)	32.3 (29.2-35.6)	16,747 (11,715-22,663)	116.9 (81.8-158.2)	1.87 (1.53-2.26)	130.3 (106.5-157.5)	10,495 (8,565-14,308)	196.8 (160.6-268.3)	0.58 (0.50-0.65)	107.9 (93.2-122.2)
Bhutan	213 (161-267)	26.6 (20.1-33.4)	0.04 (0.03-0.04)	46.6 (41.2-53.0)	76 (51-108)	97.8 (65.2-138.8)	0.01 (0.01-0.02)	163.0 (125.2-207.6)	88 (61-118)	337.9 (232.9-454.6)	0.00 (0.00-0.01)	181.2 (152.2-209.9)
India	495,511 (410,365-540,787)	37.7 (31.2-41.1)	68.21 (64.66-71.73)	51.8 (49.1-54.5)	149,826 (132,370-167,643)	133.6 (118.0-149.5)	12.43 (10.43-14.44)	110.8 (93.0-128.7)	210,843 (150,450-240,558)	456.4 (325.7-520.7)	12.05 (11.23-12.92)	260.8 (243.0-279.6)
Nepal	10,926 (8,195-13,663)	36.3 (27.2-45.4)	1.18 (1.06-1.31)	39.3 (35.3-43.6)	4,750 (3,260-6,767)	119.0 (81.7-169.6)	0.51 (0.41-0.62)	128.2 (103.1-156.5)	3,711 (2,546-4,786)	383.3 (263.0-494.3)	0.13 (0.11-0.14)	130.7 (112.0-149.1)
Pakistan	49,393 (40,404-59,640)	25.9 (21.2-31.2)	8.31 (7.36-9.40)	43.5 (38.6-49.2)	28,114 (20,168-37,952)	121.9 (87.4-164.5)	3.94 (3.14-4.82)	170.7 (136.3-208.7)	11,747 (9,285-14,684)	216.4 (171.0-270.5)	0.61 (0.53-0.69)	112.5 (97.9-127.6)
**Sub-Saharan Africa**	**650,639 (582,183-720,960)**	**66.4 (59.4-73.6)**	**57.02 (52.29-62.28)**	**58.2 (53.4-63.5)**	**312,417 (266,256-361,152)**	**199.5 (170.0-230.6)**	**15.76 (12.72-19.44)**	**100.6 (81.3-124.2)**	**138,099 (116,652-157,256)**	**768.7 (649.3-875.3)**	**4.12 (3.63-4.66)**	**229.3 (201.8-259.1)**
**Southern Sub-Saharan Africa**	**47,384 (41,130-54,257)**	**61.6 (53.4-70.5)**	**5.52 (5.11-5.97)**	**71.7 (66.4-77.5)**	**10,819 (8,731-13,304)**	**125.7 (101.4-154.5)**	**0.86 (0.69-1.05)**	**99.6 (79.8-121.7)**	**14,170 (11,840-16,383)**	**579.0 (483.8-669.4)**	**0.55 (0.49-0.61)**	**224.2 (198.7-250.2)**
Botswana	925 (493-1,351)	40.2 (21.5-58.8)	0.13 (0.12-0.14)	57.1 (52.0-62.8)	66 (34-112)	24.8 (12.8-42.5)	0.02 (0.02-0.03)	76.6 (58.7-98.5)	274 (159-395)	542.1 (314.1-781.9)	0.01 (0.01-0.01)	194.9 (166.6-223.6)
Lesotho	1,684 (1,179-2,261)	78.9 (55.2-105.9)	0.15 (0.13-0.16)	69.3 (62.9-75.8)	525 (346-755)	203.5 (134.1-292.5)	0.03 (0.02-0.04)	113.0 (89.2-140.1)	435 (269-605)	795.6 (492.3-1,107.2)	0.01 (0.01-0.01)	226.5 (190.4-264.9)
Namibia	1,232 (887-1,584)	49.3 (35.5-63.4)	0.16 (0.15-0.18)	65.3 (59.6-71.9)	313 (182-499)	94.2 (54.6-150.0)	0.04 (0.03-0.04)	107.7 (84.6-133.0)	336 (227-450)	627.5 (423.7-838.6)	0.01 (0.01-0.01)	210.6 (180.3-242.4)

Location	All Ages				Children under-5				Adults over-70			
	**Deaths (95% UI)**	**Deaths per 1000 (95% UI)**	**Millions of cases (95% UI)**	**Incidence per 1000 (95% UI)**	**Deaths (95% UI)**	**Deaths per 1000 (95% UI)**	**Millions of cases (95% UI)**	**Incidence per 1000 (95% UI)**	**Deaths (95% UI)**	**Deaths per 1000 (95% UI)**	**Millions of cases (95% UI)**	**Incidence per 1000 (95% UI)**
South Africa	30,730 (25,960-34,380)	58.3 (49.2-65.2)	3.99 (3.71-4.30)	75.7 (70.3-81.6)	5,360 (3,830-7,411)	107.0 (76.4-147.9)	0.49 (0.39-0.61)	97.6 (78.1-120.8)	9,631 (7,991-10,883)	494.2 (410.1-558.5)	0.44 (0.39-0.48)	223.4 (198.4-248.2)
Swaziland	900 (650-1,169)	67.3 (48.6-87.3)	0.08 (0.08-0.09)	62.3 (56.3-68.8)	398 (259-577)	190.8 (124.1-276.5)	0.02 (0.02-0.03)	106.4 (84.2-132.1)	169 (105-239)	605.9 (375.9-859.0)	0.01 (0.00-0.01)	188.4 (159.6-217.7)
Zimbabwe	11,914 (8,092-15,166)	74.7 (50.7-95.1)	1.00 (0.91-1.11)	62.8 (57.0-69.8)	4,156 (2,870-5,618)	164.1 (113.3-221.8)	0.26 (0.21-0.32)	102.9 (83.0-125.8)	3,325 (2,074-4,646)	1,065.5 (664.7-1,488.9)	0.07 (0.06-0.09)	239.3 (202.2-278.2)
**Western Sub-Saharan Africa**	**273,944 (236,313-315,218)**	**68.8 (59.3-79.2)**	**20.43 (18.74-22.25)**	**51.3 (47.0-55.9)**	**138,330 (110,473-170,088)**	**214.1 (171.0-263.2)**	**4.88 (3.94-5.98)**	**75.5 (61.0-92.6)**	**54,205 (44,239-64,416)**	**859.3 (701.3-1,021.1)**	**1.52 (1.32-1.73)**	**241.3 (210.0-273.7)**
Benin	7,777 (6,418-9,325)	68.6 (56.6-82.2)	0.60 (0.55-0.66)	52.8 (48.2-58.0)	3,466 (2,435-4,859)	181.4 (127.4-254.3)	0.13 (0.10-0.17)	69.8 (54.8-87.9)	1,696 (1,350-2,082)	911.8 (725.8-1,119.3)	0.05 (0.04-0.05)	247.9 (214.2-283.1)
Burkina Faso	16,350 (12,524-20,747)	87.8 (67.2-111.4)	0.95 (0.86-1.05)	51.1 (46.4-56.2)	10,223 (6,467-14,590)	320.0 (202.4-456.7)	0.25 (0.20-0.31)	77.8 (61.5-97.0)	2,169 (1,671-2,715)	859.3 (661.9-1,075.7)	0.06 (0.05-0.07)	232.5 (196.7-266.8)
Cameroon	20,298 (15,936-25,556)	84.6 (66.4-106.5)	1.34 (1.23-1.47)	56.0 (51.2-61.4)	9,908 (6,600-13,985)	255.5 (170.2-360.7)	0.36 (0.29-0.44)	92.0 (73.9-112.7)	4,899 (3,654-6,283)	1,055.5 (787.4-1,353.8)	0.12 (0.10-0.14)	262.2 (225.5-304.8)
Cape Verde	241 (196-277)	43.9 (35.8-50.6)	0.03 (0.03-0.03)	51.5 (47.3-55.8)	24 (17-34)	32.5 (23.2-44.8)	0.00 (0.00-0.01)	57.3 (44.3-72.0)	143 (109-170)	786.0 (600.2-935.5)	0.00 (0.00-0.01)	254.9 (222.8-292.2)
Chad	16,527 (13,065-21,400)	114.5 (90.5-148.3)	0.93 (0.84-1.03)	64.2 (57.9-71.5)	11,238 (8,058-15,219)	425.4 (305.0-576.2)	0.32 (0.26-0.40)	122.8 (97.9-151.9)	2,149 (1,652-2,774)	1,029.9 (791.6-1,329.4)	0.06 (0.05-0.07)	270.7 (229.3-318.5)
Cote d'Ivoire	18,565 (14,445-23,401)	80.5 (62.7-101.5)	1.25 (1.15-1.37)	54.4 (49.7-59.4)	8,015 (5,196-11,708)	225.2 (146.0-329.0)	0.28 (0.22-0.34)	77.7 (61.8-96.2)	3,933 (2,916-5,001)	1,003.8 (744.4-1,276.6)	0.10 (0.09-0.11)	254.5 (217.0-290.6)
The Gambia	1,063 (893-1,263)	52.0 (43.7-61.7)	0.11 (0.10-0.12)	54.7 (49.9-59.9)	479 (340-641)	130.5 (92.8-174.8)	0.03 (0.02-0.04)	82.3 (65.3-100.8)	243 (192-297)	848.9 (672.5-1,036.8)	0.01 (0.01-0.01)	259.5 (226.3-301.4)
Ghana	16,576 (14,011-19,645)	58.7 (49.6-69.6)	1.58 (1.45-1.72)	55.9 (51.5-60.8)	3,502 (2,331-4,991)	80.6 (53.6-114.8)	0.30 (0.24-0.37)	68.6 (54.8-84.1)	5,585 (4,422-6,841)	1,021.3 (808.7-1,251.1)	0.15 (0.13-0.17)	271.0 (238.2-311.8)
Guinea	12,523 (10,199-15,417)	97.1 (79.1-119.6)	0.83 (0.76-0.92)	64.1 (58.6-71.1)	6,594 (4,552-9,005)	324.1 (223.7-442.5)	0.23 (0.18-0.29)	112.0 (87.6-142.3)	2,445 (1,927-3,045)	1,084.6 (854.6-1,350.6)	0.06 (0.05-0.07)	280.3 (242.1-324.2)

Location	All Ages				Children under-5				Adults over-70			
	**Deaths (95% UI)**	**Deaths per 1000 (95% UI)**	**Millions of cases (95% UI)**	**Incidence per 1000 (95% UI)**	**Deaths (95% UI)**	**Deaths per 1000 (95% UI)**	**Millions of cases (95% UI)**	**Incidence per 1000 (95% UI)**	**Deaths (95% UI)**	**Deaths per 1000 (95% UI)**	**Millions of cases (95% UI)**	**Incidence per 1000 (95% UI)**
Guinea-Bissau	1,516 (1,158-1,833)	79.7 (60.9-96.3)	0.11 (0.10-0.11)	55.2 (50.4-60.2)	479 (325-649)	154.4 (104.9-209.3)	0.02 (0.02-0.03)	75.9 (60.3-95.0)	383 (289-480)	1,145.2 (863.7-1,434.1)	0.01 (0.01-0.01)	248.0 (213.7-287.4)
Liberia	2,412 (1,925-2,942)	52.2 (41.7-63.7)	0.27 (0.25-0.30)	59.0 (53.7-65.2)	849 (569-1,215)	119.0 (79.9-170.5)	0.07 (0.05-0.08)	91.6 (70.2-116.5)	671 (497-848)	829.2 (614.3-1,047.5)	0.02 (0.02-0.03)	270.6 (233.2-314.6)
Mali	7,464 (5,662-9,866)	41.6 (31.5-54.9)	0.68 (0.61-0.76)	38.1 (34.0-42.5)	5,219 (3,557-7,535)	165.7 (112.9-239.2)	0.21 (0.17-0.27)	67.8 (52.6-85.9)	835 (595-1,150)	313.7 (223.5-431.9)	0.04 (0.04-0.05)	153.9 (131.9-176.7)
Mauritania	1,962 (1,561-2,497)	48.2 (38.3-61.3)	0.20 (0.19-0.22)	50.0 (45.6-54.6)	711 (499-991)	138.3 (97.0-192.6)	0.04 (0.03-0.05)	71.9 (57.0-90.4)	582 (425-762)	743.7 (543.3-973.5)	0.02 (0.02-0.02)	234.6 (200.6-267.9)
Niger	18,652 (14,634-23,838)	92.9 (72.9-118.7)	1.17 (1.06-1.30)	58.4 (52.9-64.7)	11,612 (7,930-16,633)	315.2 (215.3-451.5)	0.34 (0.28-0.42)	93.4 (75.0-114.0)	2,578 (1,966-3,322)	881.5 (672.4-1,136.1)	0.08 (0.06-0.09)	256.6 (218.8-299.9)
Nigeria	112,867 (86,159-148,355)	61.1 (46.6-80.3)	8.61 (7.79-9.47)	46.6 (42.2-51.2)	57,446 (39,276-82,081)	194.5 (133.0-277.9)	1.83 (1.45-2.26)	61.9 (49.2-76.6)	21,784 (15,491-32,249)	793.2 (564.1-1,174.2)	0.63 (0.53-0.73)	228.7 (192.2-267.1)
Sao Tome and Principe	108 (76-138)	54.4 (38.3-69.6)	0.01 (0.01-0.01)	62.7 (57.2-69.3)	29 (20-41)	86.6 (59.4-123.6)	0.00 (0.00-0.00)	85.9 (66.4-108.9)	45 (29-60)	1,077.5 (687.6-1,445.2)	0.00 (0.00-0.00)	301.2 (261.2-347.6)
Senegal	8,227 (6,794-10,063)	53.2 (44.0-65.1)	0.90 (0.83-0.98)	58.4 (53.8-63.6)	3,338 (2,353-4,492)	131.3 (92.6-176.7)	0.26 (0.21-0.31)	101.5 (83.4-122.3)	2,081 (1,533-2,888)	754.3 (555.5-1,046.8)	0.07 (0.06-0.08)	239.5 (203.1-279.6)
Sierra Leone	6,373 (5,271-7,758)	96.2 (79.6-117.1)	0.44 (0.40-0.49)	66.9 (61.0-73.8)	3,640 (2,660-4,950)	350.1 (255.8-476.0)	0.12 (0.09-0.15)	114.4 (89.8-141.7)	911 (716-1,152)	982.2 (772.1-1,241.9)	0.03 (0.02-0.03)	281.0 (237.6-324.7)
Togo	4,445 (3,637-5,356)	60.0 (49.1-72.2)	0.41 (0.37-0.44)	54.7 (50.3-59.8)	1,559 (1,053-2,227)	142.5 (96.3-203.6)	0.09 (0.07-0.11)	78.7 (62.1-99.4)	1,074 (831-1,345)	929.3 (718.7-1,164.0)	0.03 (0.03-0.03)	258.6 (219.8-301.0)
**Eastern Sub-Saharan Africa**	**251,054 (224,563-279,640)**	**64.9 (58.0-72.2)**	**23.82 (21.75-26.14)**	**61.5 (56.2-67.5)**	**121,120 (103,742-138,312)**	**193.6 (165.8-221.1)**	**7.61 (6.11-9.31)**	**121.6 (97.6-148.9)**	**55,332 (47,039-64,979)**	**770.0 (654.6-904.2)**	**1.59 (1.40-1.79)**	**221.5 (194.8-249.4)**
Burundi	9,212 (6,981-11,883)	79.6 (60.3-102.7)	0.82 (0.73-0.91)	70.8 (62.7-78.9)	5,063 (3,097-7,639)	236.3 (144.6-356.6)	0.31 (0.24-0.39)	144.6 (112.5-182.4)	1,499 (1,008-1,919)	893.3 (600.6-1,143.4)	0.04 (0.03-0.05)	233.3 (198.8-273.1)
Comoros	415 (334-507)	53.3 (42.9-65.1)	0.05 (0.05-0.06)	64.1 (58.3-70.7)	160 (103-240)	172.8 (111.2-258.7)	0.01 (0.01-0.01)	114.3 (88.7-146.5)	97 (76-124)	722.0 (561.7-919.0)	0.00 (0.00-0.00)	229.2 (196.5-265.8)

	All Ages				Children under-5				Adults over-70			
**Location**	**Deaths (95% UI)**	**Deaths per 1000 (95% UI)**	**Millions of cases (95% UI)**	**Incidence per 1000 (95% UI)**	**Deaths (95% UI)**	**Deaths per 1000 (95% UI)**	**Millions of cases (95% UI)**	**Incidence per 1000 (95% UI)**	**Deaths (95% UI)**	**Deaths per 1000 (95% UI)**	**Millions of cases (95% UI)**	**Incidence per 1000 (95% UI)**
Djibouti	649 (488-837)	67.0 (50.3-86.4)	0.07 (0.06-0.07)	68.5 (60.9-76.5)	316 (190-506)	183.4 (110.3-293.2)	0.02 (0.02-0.03)	124.2 (94.6-159.1)	148 (108-197)	656.3 (478.0-870.1)	0.01 (0.00-0.01)	223.2 (191.2-257.2)
Eritrea	3,101 (2,281-3,983)	58.1 (42.8-74.7)	0.33 (0.30-0.37)	62.3 (56.2-69.1)	1,404 (871-2,065)	176.6 (109.5-259.6)	0.10 (0.07-0.12)	120.1 (92.3-154.3)	583 (368-773)	755.5 (476.9-1,001.9)	0.02 (0.01-0.02)	217.9 (185.0-249.2)
Ethiopia	55,573 (46,200-64,889)	54.3 (45.2-63.4)	6.33 (5.78-6.90)	61.8 (56.5-67.5)	20,030 (14,254-26,277)	131.1 (93.3-171.9)	1.91 (1.55-2.30)	125.2 (101.6-150.8)	15,516 (12,366-19,680)	725.4 (578.1-920.0)	0.46 (0.40-0.53)	214.8 (185.8-247.5)
Kenya	23,237 (19,088-27,109)	49.9 (41.0-58.2)	2.27 (2.05-2.51)	48.8 (43.9-54.0)	11,205 (9,321-13,104)	170.4 (141.8-199.3)	0.61 (0.47-0.77)	93.1 (72.2-117.2)	5,460 (3,920-7,020)	704.7 (505.9-906.0)	0.17 (0.14-0.19)	214.7 (184.5-244.3)
Madagascar	19,510 (15,446-24,350)	78.2 (61.9-97.6)	1.78 (1.60-1.98)	71.4 (64.3-79.3)	10,032 (6,780-14,132)	258.0 (174.3-363.4)	0.53 (0.42-0.67)	137.2 (107.2-172.2)	3,195 (2,332-4,198)	758.5 (553.6-996.5)	0.09 (0.08-0.10)	217.8 (186.8-249.2)
Malawi	13,322 (10,676-16,515)	74.2 (59.5-92.0)	1.18 (1.05-1.32)	65.7 (58.8-73.4)	7,172 (4,944-10,138)	224.0 (154.4-316.7)	0.44 (0.34-0.55)	136.4 (107.4-171.2)	2,910 (2,220-3,647)	792.7 (604.6-993.2)	0.09 (0.07-0.10)	236.8 (202.5-275.9)
Mozambique	18,215 (14,624-22,403)	63.2 (50.8-77.8)	1.55 (1.40-1.72)	53.9 (48.7-59.7)	10,143 (7,132-13,975)	204.2 (143.6-281.4)	0.51 (0.40-0.64)	102.5 (80.1-128.0)	3,432 (2,613-4,406)	594.3 (452.6-763.0)	0.11 (0.09-0.12)	189.2 (162.5-215.5)
Rwanda	7,194 (5,632-8,783)	59.6 (46.6-72.7)	0.74 (0.67-0.81)	61.0 (55.4-66.8)	3,579 (2,440-4,935)	191.2 (130.3-263.6)	0.22 (0.18-0.27)	118.1 (96.9-142.3)	1,507 (1,137-1,917)	761.0 (574.0-967.9)	0.05 (0.04-0.05)	231.4 (199.2-266.5)
Somalia	10,311 (7,910-13,305)	99.3 (76.1-128.1)	0.65 (0.59-0.72)	62.9 (56.9-69.2)	5,501 (3,394-8,087)	417.2 (257.4-613.2)	0.15 (0.12-0.19)	116.0 (91.3-144.5)	1,800 (1,410-2,310)	997.1 (781.2-1,279.8)	0.04 (0.04-0.05)	228.8 (194.3-265.1)
South Sudan	15,163 (10,976-20,819)	111.6 (80.8-153.2)	1.01 (0.89-1.14)	74.1 (65.8-84.2)	10,189 (6,545-14,869)	356.2 (228.8-519.8)	0.39 (0.31-0.51)	138.0 (106.8-178.3)	2,148 (1,536-3,008)	814.7 (582.7-1,140.9)	0.06 (0.05-0.07)	238.9 (202.7-278.3)
Tanzania	38,831 (31,383-46,778)	71.2 (57.6-85.8)	3.25 (2.96-3.57)	59.7 (54.3-65.5)	18,786 (12,827-25,487)	208.7 (142.5-283.1)	1.00 (0.81-1.21)	110.9 (89.5-134.8)	9,214 (7,002-11,586)	855.6 (650.2-1,075.9)	0.25 (0.21-0.28)	228.2 (194.4-263.1)
Uganda	23,050 (18,486-28,012)	57.1 (45.8-69.4)	2.83 (2.51-3.17)	70.2 (62.2-78.6)	11,205 (7,977-14,750)	149.1 (106.1-196.2)	1.12 (0.87-1.41)	148.6 (115.9-187.2)	4,941 (3,669-6,229)	820.2 (609.1-1,034.1)	0.15 (0.13-0.17)	247.3 (210.7-284.2)
Zambia	13,272 (10,758-16,203)	79.7 (64.6-97.3)	0.94 (0.85-1.03)	56.4 (51.3-61.7)	6,334 (4,456-8,697)	222.8 (156.8-306.0)	0.28 (0.22-0.34)	98.0 (78.6-120.1)	2,881 (2,195-3,699)	967.1 (736.6-1,241.5)	0.07 (0.06-0.08)	227.2 (198.2-262.7)

Location	All Ages				Children under-5				Adults over-70			
	Deaths (95% UI)	Deaths per 1000 (95% UI)	Millions of cases (95% UI)	Incidence per 1000 (95% UI)	Deaths (95% UI)	Deaths per 1000 (95% UI)	Millions of cases (95% UI)	Incidence per 1000 (95% UI)	Deaths (95% UI)	Deaths per 1000 (95% UI)	Millions of cases (95% UI)	Incidence per 1000 (95% UI)
**Central Sub-Saharan Africa**	**78,087 (61,096-99,630)**	**66.3 (51.9-84.6)**	**7.25 (6.54-8.05)**	**61.6 (55.5-68.4)**	**42,084 (27,228-62,102)**	**202.4 (130.9-298.6)**	**2.41 (1.93-3.00)**	**116.1 (92.9-144.4)**	**14,338 (11,285-18,207)**	**708.5 (557.7-899.7)**	**0.46 (0.39-0.52)**	**225.8 (193.0-258.6)**
Angola	14,298 (10,663-19,181)	55.2 (41.2-74.1)	1.63 (1.44-1.83)	62.8 (55.6-70.8)	7,853 (4,780-12,235)	160.1 (97.4-249.4)	0.58 (0.45-0.74)	119.0 (90.8-150.9)	2,247 (1,685-2,947)	651.8 (488.6-854.8)	0.08 (0.07-0.09)	223.2 (189.1-258.6)
Central African Republic	6,028 (4,490-7,994)	120.2 (89.5-159.3)	0.34 (0.31-0.38)	68.3 (61.6-75.9)	3,413 (2,225-5,103)	460.0 (299.9-687.7)	0.11 (0.09-0.14)	152.8 (120.8-190.7)	1,069 (728-1,437)	911.9 (621.1-1,225.6)	0.03 (0.02-0.03)	229.8 (196.1-267.2)
Congo	2,493 (1,828-3,241)	52.9 (38.8-68.8)	0.24 (0.22-0.27)	51.6 (46.5-56.9)	928 (563-1,492)	124.5 (75.6-200.2)	0.07 (0.06-0.09)	97.4 (77.4-122.9)	714 (487-954)	670.5 (457.4-896.8)	0.02 (0.02-0.02)	193.6 (164.4-223.7)
Democratic Republic of the Congo	53,979 (38,815-73,976)	67.9 (48.8-93.0)	4.89 (4.45-5.43)	61.5 (55.9-68.2)	29,575 (15,798-49,420)	210.4 (112.4-351.6)	1.61 (1.30-2.00)	114.6 (92.5-142.0)	9,794 (7,353-13,152)	709.0 (532.3-952.0)	0.32 (0.27-0.37)	228.7 (192.7-264.9)
Equatorial Guinea	315 (206-489)	37.6 (24.6-58.4)	0.05 (0.04-0.05)	53.8 (48.3-59.7)	86 (47-146)	86.9 (47.4-147.6)	0.01 (0.01-0.01)	96.2 (73.3-124.5)	93 (56-154)	618.0 (371.5-1,016.8)	0.00 (0.00-0.00)	224.5 (189.4-261.7)
Gabon	973 (759-1,191)	55.1 (43.0-67.5)	0.10 (0.09-0.11)	57.8 (52.6-63.6)	228 (141-352)	93.3 (57.6-143.6)	0.02 (0.02-0.03)	97.1 (75.6-121.3)	421 (309-540)	717.4 (526.0-920.5)	0.01 (0.01-0.02)	222.3 (189.1-256.7)

Appendix Figure 12. Clustered bar chart of LRI aetiologies in 2016

The bars indicate the mortality rate per 100,000 for each aetiology, summarised by age groups.

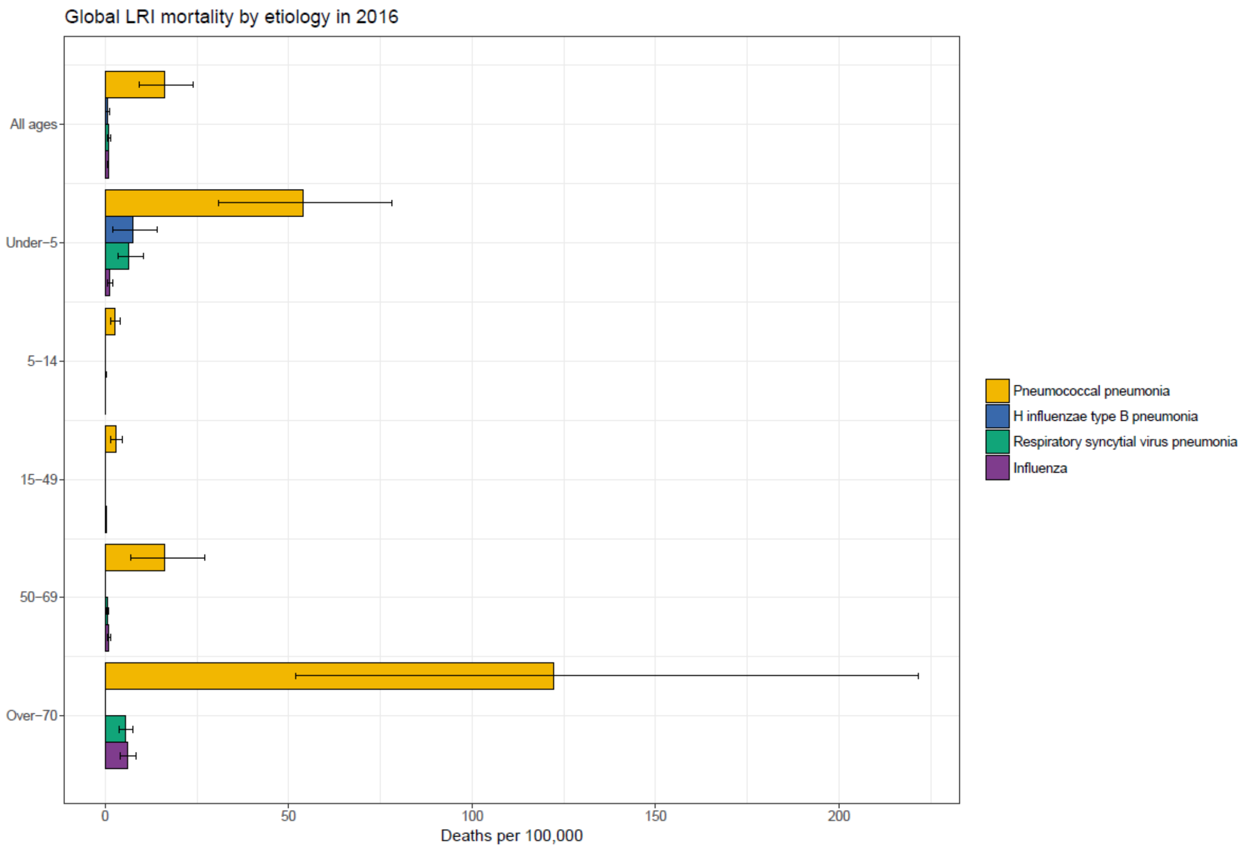

Summary of risk factors

Risk factors in the GBD 2016 study are described in detail in a separate manuscript. In general, risk factor attribution is analogous to the aetiology attribution. The population-level exposure to a risk factor was modeled using either DisMod-MR 2.1 or space-time Gaussian process regression to produce age, sex, location, year specific exposure prevalence. Risk factors were also associated with lower respiratory infections using a relative risk of LRI given exposure. This manuscript used changes in the population attributable fraction of LRI deaths due to these risk factors in the decomposition analysis and the analysis of the number needed to treat. The Table below shows the population attributable fraction for the risk factors associated with LRI among children under-5 in 2000 and 2016. The table is ordered by the attributable fraction in 2016.

Appendix Table 13. The population attributable fraction for risk factors associated with LRI deaths, globally, among children under-5 in 2000 and 2016.

Risk factor	Attributable fraction in 2000	Attributable fraction in 2016
Child wasting	58.3%<br>(42.5 to 67.1)	57.1%<br>(40.1 to 66.7)
Household air pollution from solid fuels	43.9%<br>(33.8 to 53.4)	38.3%<br>(29.2 to 47.7)
Ambient particulate matter pollution	30.3%<br>(23.7 to 37.3)	33.3%<br>(26.3 to 40.5)
Antibiotics for LRI	31.6%<br>(15.4-45.4)	30.2%<br>(14.4-43.8)
Low pneumococcal conjugate vaccine coverage	34.3%<br>(29.9-38.4)	19.4%<br>(16.6-22.2)
Child stunting	16.1%<br>(1.9 to 37.3)	13.5%<br>(1.2 to 33.7)
Low Hib vaccine coverage	35.6%<br>(18.8-49.1)	11.8%<br>(5.3-18.4)
Child underweight	15.2%<br>(10.4 to 25.4)	11.5%<br>(7.8 to 20.5)
No access to handwashing facility	10.6%<br>(6.9 to 14.1)	10.9%<br>(7.1 to 14.4)
Non-exclusive breastfeeding	8.7%<br>(5.7 to 11.8)	8.5%<br>(5.7 to 11.5)
Secondhand smoke	10.6%<br>(5.8 to 16.1)	8.2%<br>(4.4 to 12.7)
Zinc deficiency	2.10%<br>(0 to 8.2)	1.6%<br>(0 to 6.6)

Appendix Table 14. The percent change in under-5 LRI mortality attributable to change in risk factors between 2000 and 2016 by GBD region and country. Numbers in parentheses indicate the number of deaths averted, if negative, or attributable to, if positive, changes in the risk factor attribution between 2000 and 2016.

Location	Childhood stunting	Childhood underweight	Childhood wasting	Hand-washing	Breast-feeding	Second-hand smoking	Zinc deficiency	Household air pollution	Ambient particulate matter pollution	Antibiotic treatment	Hib vaccine	Pneumococcal vaccine
Global	-2.82% (-40130)	-3.69% (-52461)	-10.27% (-146136)	-0.67% (-9468)	-0.1% (-1373)	-0.88% (-12506)	-0.38% (-5348)	-9.56% (-135999)	3.7% (52571)	-1.94% (-27627)	-5.86% (-83318)	-7.24% (-103062)
Southeast Asia, East Asia, and Oceania	-2.42% (-5681)	-2.47% (-5811)	-10.26% (-24110)	-0.48% (-1128)	-0.01% (-19)	-1.59% (-3739)	-0.3% (-707)	-10.17% (-23908)	0.48% (1139)	-1.83% (-4308)	-5.63% (-13234)	-1.58% (-3706)
East Asia	-2.17% (-2413)	-1.74% (-1940)	-11.48% (-12771)	-0.87% (-965)	0.44% (488)	-1.64% (-1819)	-0.12% (-132)	-11.99% (-13332)	0.92% (1024)	-1.7% (-1892)	-6.01% (-6687)	-3.68% (-4094)
China	-2.15% (-2298)	-1.72% (-1844)	-11.53% (-12343)	-0.9% (-966)	0.48% (515)	-1.63% (-1743)	-0.13% (-135)	-12.3% (-13172)	0.86% (926)	-1.7% (-1818)	-5.87% (-6288)	-4.32% (-4622)
North Korea	-2.78% (-114)	-2.34% (-96)	-10.36% (-424)	0.04% (2)	-0.66% (-27)	-1.78% (-73)	0.08% (3)	-3.86% (-158)	2.41% (99)	-1.77% (-72)	-6.81% (-279)	0% (0)
Taiwan	-0.63% (0)	-0.35% (0)	-7.1% (-4)	-0.09% (0)	0.09% (0)	-4.63% (-3)	0.23% (0)	-3.34% (-2)	-0.28% (0)	-1.54% (-1)	-1.91% (-1)	-34.2% (-20)
Southeast Asia	-2.67% (-3227)	-3.16% (-3821)	-9.19% (-11111)	-0.12% (-140)	-0.42% (-506)	-1.59% (-1925)	-0.48% (-574)	-8.49% (-10265)	0.13% (162)	-1.98% (-2391)	-5.49% (-6640)	-0.73% (-886)
Cambodia	-3.95% (-457)	-4.63% (-535)	-9.22% (-1066)	-1.31% (-152)	-2.64% (-305)	-2.22% (-257)	-0.44% (-51)	-10.69% (-1236)	0.61% (70)	-6.01% (-696)	-7.43% (-859)	-14.89% (-1722)
Indonesia	-1.64% (-720)	-1.9% (-838)	-1.6% (-704)	0.25% (111)	-0.09% (-38)	-1.41% (-620)	-0.2% (-88)	-9.49% (-4173)	-0.15% (-66)	-1.79% (-788)	-4.53% (-1994)	1.88% (828)
Laos	-7.01% (-515)	-7.7% (-566)	-23.27% (-1711)	0.09% (6)	-0.94% (-69)	-1.33% (-98)	-0.33% (-24)	-6.74% (-495)	0.39% (29)	-0.45% (-33)	-6.08% (-447)	-10.58% (-778)
Malaysia	-0.66% (-3)	-1.16% (-6)	-11.33% (-54)	-0.04% (0)	-0.36% (-2)	-0.95% (-5)	-0.11% (-1)	-1.62% (-8)	1.1% (5)	-1.52% (-7)	-9.28% (-45)	-16.99% (-82)
Maldives	-4.34% (-1)	-4.72% (-1)	-16.02% (-2)	-1.05% (0)	0.06% (0)	-1.74% (0)	-1.29% (0)	-16.81% (-2)	-1.14% (0)	-2.12% (0)	-10.33% (-1)	1.99% (0)
Mauritius	-0.87% (0)	-1.27% (0)	-12.48% (-3)	0.06% (0)	-0.28% (0)	-0.63% (0)	-0.12% (0)	-2.33% (-1)	-2.49% (-1)	-1.42% (0)	-7.99% (-2)	-16.78% (-4)
Myanmar	-2.92% (-823)	-3.05% (-858)	-13.63% (-3840)	-0.33% (-94)	0.04% (10)	-2.7% (-760)	-0.95% (-268)	-10.24% (-2885)	1.4% (394)	-2.12% (-597)	-7.8% (-2198)	0.39% (110)
Philippines	-1.56% (-292)	-3.11% (-581)	-9.29% (-1734)	0.03% (5)	-0.92% (-172)	-0.79% (-147)	-0.53% (-100)	1.7% (318)	-1.17% (-218)	0.24% (44)	-6.25% (-1167)	-7.37% (-1376)
Sri Lanka	-2.44% (-11)	-2.87% (-13)	-10.22% (-45)	-1.01% (-4)	-0.4% (-2)	-1.93% (-9)	-0.27% (-1)	-10.62% (-47)	-2.14% (-9)	-1.25% (-6)	-6.51% (-29)	0% (0)
Seychelles	0% (0)	-0.57% (0)	-0.75% (0)	0.04% (0)	-0.61% (0)	-1.47% (0)	-0.25% (0)	-1.85% (0)	-3.77% (0)	-2.04% (0)	-10.56% (0)	1.79% (0)
Thailand	-0.01% (0)	0.38% (8)	-0.09% (-2)	-0.07% (-1)	0.14% (3)	-0.74% (-16)	-0.04% (-1)	-9.73% (-204)	-0.25% (-5)	-2.14% (-45)	-3.79% (-80)	-14.59% (-306)
Timor-Leste	-7.07% (-31)	-8.57% (-38)	-12.52% (-55)	0.16% (1)	0.65% (3)	-2.43% (-11)	-0.6% (-3)	-10.79% (-48)	-2.01% (-9)	-1.58% (-7)	-6.49% (-29)	2.42% (11)
Vietnam	-4.92% (-374)	-5.18% (-394)	-24.91% (-1894)	-0.15% (-11)	0.87% (66)	-0.05% (-4)	-0.5% (-38)	-19.52% (-1485)	-0.37% (-28)	-3.36% (-256)	-4.72% (-359)	1.56% (118)

Location	Childhood stunting	Childhood underweight	Childhood wasting	Hand-washing	Breast-feeding	Second-hand smoking	Zinc deficiency	Household air pollution	Ambient particulate matter pollution	Antibiotic treatment	Hib vaccine	Pneumococcal vaccine
Oceania	-1.35% (-38)	-1.63% (-46)	-7.55% (-213)	-0.83% (-23)	-0.01% (0)	0.27% (8)	-0.02% (0)	-10.49% (-296)	-1.71% (-48)	-0.8% (-23)	-5.74% (-162)	-10.27% (-290)
American Samoa	-0.68% (0)	-0.7% (0)	-5.42% (0)	-0.35% (0)	0.01% (0)	-1.39% (0)	0.01% (0)	-5.36% (0)	-0.51% (0)	-0.45% (0)	-5.82% (0)	0% (0)
Federated States of Micronesia	-0.83% (0)	-0.87% (0)	-5.39% (-1)	-0.74% (0)	0.09% (0)	-0.92% (0)	0% (0)	-10.51% (-1)	-0.48% (0)	-0.7% (0)	-0.17% (0)	-7.61% (-1)
Fiji	-0.45% (0)	-0.96% (-1)	-12.46% (-9)	-0.41% (0)	0.16% (0)	-2.31% (-2)	0.01% (0)	-14.25% (-10)	-1.09% (-1)	-0.68% (0)	-0.89% (-1)	-29.8% (-21)
Guam	-0.22% (0)	-0.32% (0)	-1.2% (0)	0% (0)	0.45% (0)	0.09% (0)	-0.08% (0)	-0.33% (0)	0.45% (0)	-0.66% (0)	-6.25% (0)	-31.65% (-2)
Kiribati	-2.41% (-1)	-1.73% (0)	-11.08% (-2)	-1.35% (0)	-0.07% (0)	0.28% (0)	0.02% (0)	-8.17% (-2)	-0.38% (0)	-0.86% (0)	-6.9% (-2)	-11.39% (-3)
Marshall Islands	-1.07% (0)	-1.22% (0)	-7.03% (-1)	-0.94% (0)	-0.01% (0)	-2.01% (0)	-0.05% (0)	-8.81% (-1)	0.05% (0)	-0.76% (0)	-0.66% (0)	-3.11% (0)
Northern Mariana Islands	-0.15% (0)	-0.3% (0)	-1.45% (0)	0% (0)	-0.37% (0)	-1.84% (0)	-0.85% (0)	-3.43% (0)	0.18% (0)	-0.47% (0)	0% (0)	0% (0)
Papua New Guinea	-1.36% (-35)	-1.68% (-43)	-7.33% (-188)	-0.82% (-21)	-0.02% (0)	0.36% (9)	-0.01% (0)	-10.49% (-268)	-1.85% (-47)	-0.8% (-20)	-5.92% (-151)	-9.54% (-244)
Samoa	-0.29% (0)	-0.05% (0)	-0.08% (0)	-0.48% (0)	0.1% (0)	-1.61% (0)	-0.15% (0)	-10.34% (-1)	-0.48% (0)	-0.4% (0)	-7.9% (-1)	1.91% (0)
Solomon Islands	-2.33% (-1)	-2.24% (-1)	-11.33% (-7)	-1.11% (-1)	-0.14% (0)	1.53% (1)	-0.38% (0)	-7.97% (-5)	1.54% (1)	-1.2% (-1)	-8.75% (-6)	-22.4% (-14)
Tonga	-1.06% (0)	-0.64% (0)	-9.15% (-1)	-0.99% (0)	0.09% (0)	-1.34% (0)	-0.03% (0)	-15.86% (-1)	-1.02% (0)	-0.82% (0)	-10.07% (-1)	-0.01% (0)
Vanuatu	-0.72% (0)	-0.28% (0)	-8.1% (-4)	-1.49% (-1)	-0.07% (0)	-0.69% (0)	-0.04% (0)	-10.65% (-6)	-1.45% (-1)	-0.75% (0)	-6.74% (-4)	2.41% (1)
Central Europe, Eastern Europe, and Central Asia	-3.5% (-1253)	-2.47% (-884)	-13.15% (-4709)	-1.16% (-414)	-0.11% (-38)	-0.33% (-119)	-0.63% (-226)	-6.81% (-2439)	1.88% (674)	-1.13% (-406)	-6.5% (-2329)	-5.12% (-1835)
Central Asia	-3.95% (-1143)	-2.93% (-848)	-14.33% (-4145)	-1.35% (-390)	-0.2% (-57)	0.06% (17)	-0.75% (-217)	-7.6% (-2198)	2.42% (700)	-0.99% (-285)	-6.91% (-1998)	-4.31% (-1248)
Armenia	0.14% (0)	0.22% (1)	1.32% (3)	-0.49% (-1)	0.91% (2)	-0.34% (-1)	-0.75% (-2)	-8.01% (-21)	4.87% (12)	1.56% (4)	-6.14% (-16)	-15.23% (-39)
Azerbaijan	-2.78% (-96)	-3.5% (-120)	-10.68% (-368)	-1.68% (-58)	1.23% (42)	5.04% (173)	-2.03% (-70)	-8.55% (-294)	5.03% (173)	-1% (-34)	-6.49% (-223)	-10.97% (-378)
Georgia	-0.91% (-5)	-0.37% (-2)	-6.26% (-34)	-0.78% (-4)	0.35% (2)	-0.39% (-2)	-0.39% (-2)	-3.54% (-19)	2.6% (14)	-0.59% (-3)	-5.1% (-27)	-11.8% (-63)
Kazakhstan	-1.93% (-48)	-2% (-50)	-15.67% (-393)	-0.7% (-17)	-0.11% (-3)	1.17% (29)	0.08% (2)	-4% (-100)	1.81% (45)	-1.21% (-30)	-6.34% (-159)	-12.65% (-317)
Kyrgyzstan	-3.41% (-56)	-1.3% (-21)	-11.53% (-191)	-1.18% (-20)	-1.94% (-32)	-0.38% (-6)	-0.26% (-4)	-9.84% (-163)	2.27% (37)	-1.43% (-24)	-9.18% (-152)	1.44% (24)
Mongolia	-5.25% (-62)	-4.79% (-57)	-30.84% (-367)	-0.92% (-11)	0.94% (11)	0.73% (9)	-0.13% (-2)	-9% (-107)	5.91% (70)	-1.78% (-21)	-7.77% (-92)	0.74% (9)
Tajikistan	-4.12% (-160)	-3.7% (-144)	3.88% (151)	-1.17% (-45)	-0.65% (-25)	-4.27% (-165)	-0.99% (-38)	-18.73% (-726)	-0.28% (-11)	-1.41% (-54)	-6.78% (-263)	1.6% (62)

Location	Childhood stunting	Childhood underweight	Childhood wasting	Hand-washing	Breast-feeding	Second-hand smoking	Zinc deficiency	Household air pollution	Ambient particulate matter pollution	Antibiotic treatment	Hib vaccine	Pneumococcal vaccine
Turkmenistan	-3.94% (-134)	-2.23% (-75)	-16.4% (-556)	-1.58% (-53)	0.25% (8)	-0.01% (0)	-0.87% (-30)	-0.55% (-19)	1.07% (36)	-0.98% (-33)	-5.68% (-192)	0% (0)
Uzbekistan	-4.83% (-583)	-3.13% (-378)	-19.79% (-2391)	-1.48% (-179)	-0.52% (-63)	-0.17% (-20)	-0.59% (-72)	-6.2% (-749)	2.66% (322)	-0.73% (-89)	-8.62% (-1041)	-22.09% (-2668)
Central Europe	-1.47% (-42)	-0.66% (-19)	-4.61% (-132)	-0.33% (-9)	-0.08% (-2)	-1.99% (-57)	-0.02% (-1)	-6.3% (-180)	-0.77% (-22)	-1.82% (-52)	-5.91% (-169)	-3.41% (-97)
Albania	-4.2% (-18)	-2.68% (-12)	-7.26% (-32)	-0.39% (-2)	-0.06% (0)	0.28% (1)	-0.1% (0)	-12.04% (-53)	0.17% (1)	-2.14% (-9)	-4.77% (-21)	-12.81% (-56)
Bosnia and Herzegovina	0.04% (0)	0.04% (0)	2.66% (1)	-0.13% (0)	0.26% (0)	1.36% (0)	0.27% (0)	-9.41% (-2)	0.45% (0)	-3.07% (-1)	-9.1% (-2)	1.67% (0)
Bulgaria	-1.03% (-2)	-0.62% (-1)	-6.23% (-15)	-0.15% (0)	0.38% (1)	-1.63% (-4)	-0.08% (0)	-2.35% (-6)	-1.6% (-4)	-1.67% (-4)	-8.74% (-21)	-21.96% (-52)
Croatia	-0.76% (0)	-0.21% (0)	-6.36% (-1)	-0.1% (0)	0.1% (0)	-2.43% (0)	-0.3% (0)	-2.68% (0)	0.88% (0)	-1.67% (0)	-9.03% (-2)	1.76% (0)
Czech Republic	-0.27% (0)	-0.08% (0)	-4.03% (-1)	-0.08% (0)	0.42% (0)	0.82% (0)	-0.05% (0)	-0.62% (0)	-1.25% (0)	-2.43% (-1)	-28.42% (-7)	-70.89% (-17)
Hungary	-0.34% (0)	-0.13% (0)	-3.51% (-2)	-0.09% (0)	0.18% (0)	-2.93% (-2)	0.02% (0)	-2.64% (-2)	0.15% (0)	-1.97% (-1)	-0.02% (0)	-57.43% (-34)
Macedonia	0.03% (0)	0.23% (0)	-0.77% (0)	-0.09% (0)	-0.36% (0)	-1.03% (0)	-0.17% (0)	-8.81% (-3)	-1.69% (-1)	0.37% (0)	-4.99% (-2)	1.22% (0)
Montenegro	0.51% (0)	0.31% (0)	0.08% (0)	-0.21% (0)	-0.08% (0)	-0.74% (0)	-0.04% (0)	-0.39% (0)	0.89% (0)	-10.24% (-1)	-6.59% (-1)	1.45% (0)
Poland	-1.15% (-2)	-0.35% (-1)	-9.47% (-16)	-0.12% (0)	0.32% (1)	-2.48% (-4)	0.09% (0)	-2.1% (-4)	-0.38% (-1)	-2.22% (-4)	-7.58% (-13)	-20.15% (-34)
Romania	-1.13% (-19)	-0.4% (-7)	-3.78% (-64)	-0.4% (-7)	-0.2% (-3)	-2.93% (-49)	0% (0)	-6.15% (-104)	-1.03% (-17)	-1.55% (-26)	-5.59% (-94)	1.2% (20)
Serbia	1.21% (1)	1.88% (2)	4.95% (5)	-0.26% (0)	-0.04% (0)	1.25% (1)	-0.13% (0)	-7.71% (-7)	-0.14% (0)	-3.69% (-3)	-10.17% (-9)	2% (2)
Slovakia	-1.03% (-1)	-0.27% (0)	-8.87% (-6)	-0.09% (0)	-0.27% (0)	0.91% (1)	-0.07% (0)	-0.67% (0)	-0.81% (-1)	-2.1% (-1)	-1.28% (-1)	-33.42% (-21)
Slovenia	-0.9% (0)	-0.21% (0)	-7.6% (0)	-0.05% (0)	-0.39% (0)	-2.53% (0)	0.09% (0)	-2.43% (0)	0.62% (0)	-1.85% (0)	-0.37% (0)	-23.03% (-1)
Eastern Europe	-1.68% (-68)	-0.43% (-17)	-10.77% (-433)	-0.38% (-15)	0.54% (22)	-1.97% (-79)	-0.22% (-9)	-1.53% (-61)	-0.11% (-4)	-1.71% (-69)	-3.2% (-129)	-12.97% (-521)
Belarus	-0.74% (-1)	-0.36% (-1)	-6.26% (-9)	-0.57% (-1)	0.24% (0)	-0.77% (-1)	0.08% (0)	-1.19% (-2)	-0.25% (0)	-2.05% (-3)	-4.13% (-6)	-12.46% (-17)
Estonia	-1.41% (0)	-0.18% (0)	-7.52% (-1)	-0.2% (0)	0.57% (0)	1.4% (0)	0.35% (0)	-2.47% (0)	-1.91% (0)	-1.53% (0)	-6.21% (-1)	-12.54% (-2)
Latvia	-1.35% (0)	-0.2% (0)	-7.01% (-1)	-0.26% (0)	0.2% (0)	0.12% (0)	-0.22% (0)	-1.62% (0)	-2.3% (0)	-1.74% (0)	-0.36% (0)	-14.93% (-3)
Lithuania	-1.54% (-1)	-0.23% (0)	-8.49% (-3)	-0.24% (0)	0.7% (0)	-1.47% (0)	0.2% (0)	0.13% (0)	-1.04% (0)	-1.62% (-1)	-8% (-3)	-18.79% (-6)
Moldova	-1.42% (-4)	-0.59% (-2)	-10.76% (-34)	-1.03% (-3)	-0.05% (0)	-2.78% (-9)	-0.1% (0)	-14.39% (-45)	-0.91% (-3)	-1.28% (-4)	-5.14% (-16)	-6.6% (-21)
Russia	-1.72% (-50)	-0.44% (-13)	-12.21% (-357)	-0.34% (-10)	0.62% (18)	-2.31% (-67)	-0.26% (-8)	-0.13% (-4)	0.09% (3)	-1.97% (-58)	-3.28% (-96)	-16.04% (-469)

Location	Childhood stunting	Childhood underweight	Childhood wasting	Hand-washing	Breast-feeding	Second-hand smoking	Zinc deficiency	Household air pollution	Ambient particulate matter pollution	Antibiotic treatment	Hib vaccine	Pneumococcal vaccine
Ukraine	-1.84% (-11)	-0.33% (-2)	-4.94% (-29)	-0.2% (-1)	0.51% (3)	-0.29% (-2)	-0.15% (-1)	-1.79% (-10)	-0.45% (-3)	-0.53% (-3)	-2.01% (-12)	0.97% (6)
High-income	-0.38% (-13)	-0.39% (-14)	-5.49% (-193)	-0.2% (-7)	-0.23% (-8)	-2.19% (-77)	-0.02% (-1)	-0.91% (-32)	-0.77% (-27)	-0.31% (-11)	-1.27% (-45)	-25.89% (-909)
High-income Asia Pacific	0.21% (1)	-0.15% (-1)	-1.01% (-5)	-0.05% (0)	0.13% (1)	-1.41% (-7)	-0.06% (0)	-0.02% (0)	0.87% (4)	-0.38% (-2)	-8.48% (-42)	-27.49% (-137)
Brunei	-0.89% (0)	-1.51% (0)	-10.82% (0)	-0.13% (0)	-0.11% (0)	-3% (0)	0.01% (0)	-0.19% (0)	1.03% (0)	-0.55% (0)	-13.32% (-1)	-0.01% (0)
Japan	0.32% (1)	-0.16% (-1)	-0.65% (-2)	-0.04% (0)	-0.04% (0)	0.36% (1)	-0.06% (0)	-0.01% (0)	0.5% (2)	-0.39% (-1)	-8.62% (-27)	-30.37% (-95)
South Korea	0.09% (0)	-0.06% (0)	-0.92% (-2)	-0.08% (0)	0.46% (1)	-4.56% (-7)	-0.1% (0)	-0.01% (0)	1.25% (2)	-0.35% (-1)	-8.01% (-13)	-19.51% (-32)
Singapore	-0.28% (0)	-0.32% (0)	-5.47% (-1)	-0.11% (0)	0.14% (0)	-2.8% (-1)	0.34% (0)	-0.07% (0)	3.48% (1)	-0.4% (0)	-8.12% (-2)	-21.5% (-4)
Australasia	-0.28% (0)	-0.08% (0)	-2.07% (-1)	-0.06% (0)	0.75% (0)	0.42% (0)	-0.11% (0)	-0.1% (0)	-0.89% (0)	-0.5% (0)	-0.73% (0)	-43.73% (-23)
Australia	-0.29% (0)	-0.08% (0)	-2.15% (-1)	-0.06% (0)	0.84% (0)	0.24% (0)	-0.15% (0)	-0.1% (0)	-0.87% (0)	-0.49% (0)	-0.64% (0)	-40.75% (-17)
New Zealand	-0.25% (0)	-0.07% (0)	-1.8% (0)	-0.06% (0)	0.42% (0)	1.08% (0)	0.01% (0)	-0.09% (0)	-0.98% (0)	-0.54% (0)	-1.35% (0)	-62.07% (-7)
Western Europe	-0.14% (-1)	-0.04% (0)	-1.9% (-13)	-0.03% (0)	-0.11% (-1)	-1.42% (-10)	0% (0)	-0.47% (-3)	-0.7% (-5)	-0.47% (-3)	-0.89% (-6)	-29.94% (-207)
Andorra	-0.07% (0)	-0.03% (0)	-1.63% (0)	-0.02% (0)	0.01% (0)	-1.38% (0)	0.07% (0)	-0.42% (0)	-0.22% (0)	-0.57% (0)	-1.2% (0)	-40.78% (0)
Austria	-0.07% (0)	-0.02% (0)	-1.49% (0)	-0.03% (0)	0.05% (0)	-0.39% (0)	-0.1% (0)	-0.43% (0)	-0.21% (0)	-0.45% (0)	-1.59% (0)	-27.48% (-3)
Belgium	-0.06% (0)	-0.01% (0)	-1.28% (0)	-0.02% (0)	0.11% (0)	-2.12% (0)	-0.08% (0)	-0.16% (0)	-0.67% (0)	-0.57% (0)	-2.72% (0)	-41.4% (-6)
Cyprus	-0.12% (0)	-0.05% (0)	-2.33% (0)	-0.03% (0)	-0.01% (0)	-0.78% (0)	-0.03% (0)	-0.39% (0)	1.7% (0)	-0.38% (0)	-11.23% (0)	-35.61% (-1)
Denmark	-0.05% (0)	-0.02% (0)	-1.14% (0)	-0.02% (0)	0.16% (0)	-1.89% (0)	0% (0)	-0.09% (0)	-0.6% (0)	-0.42% (0)	-0.12% (0)	-38.05% (-3)
England	-0.21% (0)	-0.05% (0)	-1.99% (-4)	-0.03% (0)	-0.24% (0)	-0.62% (-1)	-0.08% (0)	-0.44% (-1)	-1.23% (-2)	#VALUE!	#VALUE!	#VALUE!
Finland	-0.24% (0)	-0.07% (0)	-2.22% (0)	-0.04% (0)	-0.12% (0)	-0.59% (0)	-0.11% (0)	-0.52% (0)	-0.84% (0)	-0.44% (0)	#VALUE!	-29.09% (-3)
France	-0.05% (0)	-0.02% (0)	-1.3% (0)	-0.02% (0)	0.14% (0)	0.99% (0)	0.07% (0)	-0.16% (0)	-0.79% (0)	-0.34% (0)	-0.23% (0)	-25.45% (-2)
Germany	-0.08% (0)	-0.02% (0)	-1.72% (-1)	-0.02% (0)	-0.11% (0)	-3.7% (-3)	-0.06% (0)	-0.26% (0)	-0.62% (0)	-0.47% (0)	-0.45% (0)	-23.6% (-18)
Greece	-0.05% (0)	-0.06% (0)	-1.52% (-1)	-0.02% (0)	0.13% (0)	-1.7% (-1)	0.21% (0)	-0.35% (0)	-0.49% (0)	-0.49% (0)	-2.51% (-2)	-47.26% (-34)
Iceland	-0.12% (0)	-0.04% (0)	-2.39% (-1)	-0.02% (0)	-0.18% (0)	-4.55% (-1)	0.07% (0)	-0.47% (0)	-0.59% (0)	-0.43% (0)	-1.65% (0)	-20.8% (-5)
Ireland	-0.08% (0)	-0.04% (0)	-1.74% (0)	-0.02% (0)	0.22% (0)	-2.74% (0)	0.88% (0)	-0.17% (0)	-0.7% (0)	-0.38% (0)	0.61% (0)	-42.21% (0)

Location	Childhood stunting	Childhood underweight	Childhood wasting	Hand-washing	Breast-feeding	Second-hand smoking	Zinc deficiency	Household air pollution	Ambient particulate matter pollution	Antibiotic treatment	Hib vaccine	Pneumococcal vaccine
Israel	-0.11% (0)	-0.03% (0)	-2.28% (0)	-0.03% (0)	-0.29% (0)	-3.76% (0)	-0.04% (0)	-0.33% (0)	-0.58% (0)	-0.54% (0)	-2.34% (0)	-48.38% (-6)
Italy	-0.09% (0)	-0.02% (0)	-1.68% (0)	-0.03% (0)	0.04% (0)	-1.23% (0)	-0.15% (0)	-0.27% (0)	1.25% (0)	-0.42% (0)	-0.27% (0)	-35.46% (-9)
Luxembourg	-0.07% (0)	-0.03% (0)	-1.33% (-1)	-0.03% (0)	-0.17% (0)	-2.48% (-2)	0.12% (0)	-0.36% (0)	0.01% (0)	-0.48% (0)	-2.9% (-2)	-37.87% (-26)
Malta	-0.05% (0)	-0.02% (0)	-1.11% (0)	-0.02% (0)	0.31% (0)	-1.3% (0)	-0.13% (0)	-0.21% (0)	-0.58% (0)	-0.38% (0)	-0.43% (0)	-37.38% (0)
Netherlands	-0.1% (0)	-0.03% (0)	-1.94% (0)	-0.03% (0)	0.27% (0)	-2.2% (0)	0.18% (0)	-0.53% (0)	-0.92% (0)	-0.71% (0)	-2.29% (0)	-44.58% (-1)
Northern Ireland	-0.07% (0)	-0.03% (0)	-1.66% (0)	-0.02% (0)	0.02% (0)	-0.52% (0)	-0.01% (0)	-0.15% (0)	-0.36% (0)	-0.41% (0)	-0.07% (0)	-42.75% (-12)
Norway	-0.07% (0)	0% (0)	-1.6% (0)	-0.02% (0)	-0.3% (0)	-0.45% (0)	-0.09% (0)	-0.27% (0)	-0.62% (0)	-0.39% (0)	#VALUE!	-46.05% (-4)
Portugal	-0.06% (0)	-0.03% (0)	-1.36% (0)	-0.02% (0)	0.27% (0)	-0.55% (0)	0.4% (0)	-0.27% (0)	-1.55% (0)	-0.39% (0)	-0.49% (0)	-37.05% (-3)
Scotland	-0.12% (0)	-0.04% (0)	-2.25% (-1)	-0.05% (0)	0% (0)	-1.05% (0)	0.19% (0)	-1.62% (-1)	-0.17% (0)	-0.39% (0)	0.07% (0)	-24.99% (-11)
Spain	-0.09% (0)	-0.01% (0)	-2.33% (0)	-0.03% (0)	-0.39% (0)	-0.67% (0)	0.15% (0)	-0.76% (0)	-0.7% (0)	-0.41% (0)	#VALUE!	-41.67% (-5)
Sweden	-0.11% (0)	-0.07% (0)	-2.86% (-1)	-0.03% (0)	0.02% (0)	-1.9% (-1)	0.04% (0)	-0.84% (0)	-0.11% (0)	-0.53% (0)	-0.57% (0)	-26.95% (-13)
Switzerland	-0.04% (0)	0.02% (0)	-0.58% (0)	-0.02% (0)	0.31% (0)	-1.95% (0)	0.34% (0)	-0.23% (0)	-1.02% (0)	-0.49% (0)	0.11% (0)	-74.36% (-7)
Wales	-0.04% (0)	-0.02% (0)	-0.91% (0)	-0.02% (0)	0.41% (0)	-1.58% (0)	-0.01% (0)	-0.38% (0)	-0.6% (0)	-0.45% (0)	-0.71% (0)	-20.68% (-2)
Southern Latin America	-0.11% (0)	-0.03% (0)	-2.42% (0)	-0.04% (0)	-0.5% (0)	-0.09% (0)	-0.01% (0)	-0.45% (0)	-0.81% (0)	-0.43% (0)	#VALUE!	-11.57% (-1)
Argentina	-0.79% (-10)	-0.74% (-10)	-9.6% (-127)	-0.47% (-6)	0.21% (3)	-1.9% (-25)	-0.02% (0)	-2.22% (-29)	-0.87% (-11)	-0.11% (-1)	-0.72% (-10)	-17.63% (-234)
Chile	-0.99% (-10)	-0.97% (-9)	-12.63% (-123)	-0.57% (-6)	0.19% (2)	-2.32% (-23)	-0.01% (0)	-1.9% (-18)	-1.07% (-10)	-0.08% (-1)	-0.8% (-8)	-16.87% (-165)
Uruguay	-0.14% (0)	0% (0)	-0.11% (0)	-0.2% (-1)	0.32% (1)	-0.34% (-1)	-0.07% (0)	-3.9% (-10)	0.01% (0)	-0.22% (-1)	-0.05% (0)	-22.57% (-60)
High-income North America	-0.49% (0)	-0.38% (0)	-4.41% (-4)	-0.17% (0)	0.13% (0)	-1.92% (-2)	0.1% (0)	-0.66% (-1)	-1.25% (-1)	-0.07% (0)	-0.55% (0)	-21.49% (-18)
Canada	-0.36% (-3)	-0.37% (-4)	-5.64% (-53)	-0.05% (0)	-1.19% (-11)	-3.87% (-37)	0% (0)	-0.02% (0)	-1.56% (-15)	-0.44% (-4)	-0.19% (-2)	-31.35% (-296)
Greenland	-0.33% (0)	-0.17% (0)	-4.32% (-2)	-0.04% (0)	-0.46% (0)	-1.21% (-1)	0.14% (0)	-0.04% (0)	-0.3% (0)	-0.5% (0)	-0.85% (0)	-70.24% (-33)
United States	-0.51% (0)	-0.35% (0)	-5.92% (0)	-0.07% (0)	0.42% (0)	-1.02% (0)	-0.24% (0)	-0.61% (0)	-2.16% (0)	-0.51% (0)	-2.13% (0)	-56.53% (0)
Latin America and Caribbean	-0.36% (-3)	-0.38% (-3)	-5.71% (-51)	-0.05% (0)	-1.22% (-11)	-4.01% (-36)	-0.01% (0)	-0.02% (0)	-1.63% (-15)	-0.43% (-4)	-0.18% (-2)	-30.73% (-275)
Caribbean	-2.26% (-1247)	-1.45% (-800)	-9.87% (-5448)	-0.91% (-501)	-0.25% (-138)	-0.89% (-492)	-0.21% (-114)	-7.66% (-4229)	-3.49% (-1930)	-1.63% (-899)	-2.54% (-1403)	-12.67% (-6998)

Location	Childhood stunting	Childhood underweight	Childhood wasting	Hand-washing	Breast-feeding	Second-hand smoking	Zinc deficiency	Household air pollution	Ambient particulate matter pollution	Antibiotic treatment	Hib vaccine	Pneumococcal vaccine
Antigua and Barbuda	-1.58% (-96)	-2.1% (-128)	-12.07% (-736)	0.04% (2)	-1.06% (-65)	-0.64% (-39)	-1.23% (-75)	-5.56% (-339)	-3.63% (-221)	-4.28% (-261)	-6.37% (-389)	1.19% (72)
The Bahamas	-0.3% (0)	-0.35% (0)	-4.01% (0)	-0.68% (0)	-0.01% (0)	-0.84% (0)	-0.17% (0)	-1.24% (0)	-1.24% (0)	-1.67% (0)	-0.31% (0)	-2.69% (0)
Barbados	0.01% (0)	-0.18% (0)	-1.71% (0)	-0.1% (0)	-0.11% (0)	0.2% (0)	0.11% (0)	-1.57% (0)	-4.96% (0)	-2.07% (0)	-0.94% (0)	-24.31% (-2)
Belize	-0.19% (0)	-0.21% (0)	-1.39% (0)	-0.15% (0)	-0.25% (0)	-0.7% (0)	-0.04% (0)	-0.09% (0)	-1.64% (0)	-1.67% (0)	-0.64% (0)	-24.49% (-1)
Bermuda	-1.58% (0)	0.15% (0)	-0.8% (0)	0.05% (0)	0.13% (0)	-1.56% (0)	-0.17% (0)	-4.39% (-1)	-5.51% (-1)	-3.72% (-1)	-5.86% (-1)	1.89% (0)
Cuba	-0.23% (0)	-0.36% (0)	-3.94% (0)	-0.12% (0)	0.15% (0)	-1.46% (0)	0.69% (0)	-1.88% (0)	-1.02% (0)	-2.21% (0)	0% (0)	0% (0)
Dominica	0.99% (1)	0.05% (0)	0.58% (1)	-0.75% (-1)	-0.39% (-1)	-2.06% (-3)	0.18% (0)	-1.91% (-2)	-3.01% (-4)	0.34% (0)	-1.04% (-1)	-0.42% (-1)
Dominican Republic	-0.45% (0)	-0.53% (0)	-5.12% (0)	-1.24% (0)	0.01% (0)	-0.64% (0)	-0.09% (0)	-9.94% (0)	-0.98% (0)	-2.73% (0)	-8.06% (0)	-22.9% (0)
Grenada	-0.83% (-7)	-0.57% (-4)	-5.57% (-44)	0.15% (1)	0.81% (6)	-2.77% (-22)	-0.92% (-7)	-5.04% (-39)	-3.21% (-25)	-0.84% (-7)	-6.3% (-49)	-2.9% (-23)
Guyana	-0.94% (0)	-0.95% (0)	-8.23% (0)	-1.31% (0)	0.07% (0)	-1.68% (0)	-0.17% (0)	-5.03% (0)	-1.04% (0)	-2.38% (0)	-1.08% (0)	-0.35% (0)
Haiti	0.22% (0)	-1.01% (-1)	-11.94% (-6)	0.4% (0)	-0.06% (0)	-0.6% (0)	-0.11% (0)	-3.94% (-2)	-3.49% (-2)	-1.34% (-1)	-5.9% (-3)	-15.8% (-8)
Jamaica	-1.83% (-88)	-2.48% (-120)	-13.65% (-661)	0.07% (3)	-1.46% (-71)	-0.23% (-11)	-1.4% (-68)	-5.86% (-284)	-3.74% (-181)	-5.16% (-250)	-6.43% (-312)	2.31% (112)
Puerto Rico	-1.17% (-1)	-1.26% (-1)	-12.27% (-13)	-0.66% (-1)	-0.54% (-1)	-1.42% (-2)	-0.06% (0)	-5.52% (-6)	-3.87% (-4)	-1.72% (-2)	-7.91% (-8)	1.58% (2)
Saint Lucia	-0.05% (0)	-0.1% (0)	-1.31% (-1)	-0.06% (0)	0.05% (0)	0.08% (0)	-0.01% (0)	-0.04% (0)	-2.2% (-1)	-1.67% (-1)	-5.3% (-2)	-20.66% (-10)
Saint Vincent and the Grenadines	-0.44% (0)	-0.56% (0)	-8.05% (0)	-0.2% (0)	0% (0)	-1.36% (0)	-0.05% (0)	-6.56% (0)	-1.41% (0)	-1.87% (0)	-6.42% (0)	0% (0)
Suriname	-0.53% (0)	-0.62% (0)	-5.28% (0)	-0.89% (0)	0.04% (0)	-0.4% (0)	-0.14% (0)	-14.09% (-1)	-1.15% (0)	-1.7% (0)	-7.32% (0)	-0.01% (0)
Trinidad and Tobago	-1.59% (-1)	-2.02% (-1)	-17.49% (-9)	-0.88% (0)	0.68% (0)	-0.68% (0)	-0.24% (0)	-5.99% (-3)	-3.5% (-2)	0.63% (0)	-6.47% (-3)	-1.22% (-1)
Virgin Islands, U.S.	-0.3% (0)	-0.43% (0)	-5.83% (-2)	-0.69% (0)	0.56% (0)	-2.47% (-1)	-0.39% (0)	-0.29% (0)	-1.63% (0)	-0.91% (0)	-1.03% (0)	-13.78% (-4)
Andean Latin America	-0.04% (0)	-0.1% (0)	-1.22% (0)	-0.06% (0)	-0.07% (0)	0.9% (0)	0.06% (0)	-0.38% (0)	-1.18% (0)	-1.39% (0)	-1.79% (0)	-8.11% (0)
Bolivia	-2.68% (-324)	-1.31% (-158)	-6.49% (-784)	-1.26% (-152)	-0.36% (-44)	-1.13% (-136)	-0.3% (-36)	-9% (-1087)	-4.13% (-499)	-2.4% (-290)	-3.17% (-383)	-14.7% (-1776)
Ecuador	-3.38% (-176)	-1.5% (-78)	-6.9% (-359)	-1.29% (-67)	-0.36% (-19)	-0.99% (-52)	-0.28% (-15)	-10.96% (-570)	-4.12% (-214)	-4.81% (-250)	-1.57% (-82)	-12.28% (-638)
Peru	-2.53% (-49)	-2.41% (-47)	-14.27% (-276)	-1.21% (-23)	-0.06% (-1)	-0.86% (-17)	0% (0)	-6.53% (-126)	-3.34% (-64)	-0.16% (-3)	-6.33% (-122)	-15.17% (-293)
Central Latin America	-2.02% (-100)	-0.68% (-34)	-3.03% (-150)	-1.24% (-62)	-0.48% (-24)	-1.38% (-68)	-0.42% (-21)	-7.9% (-391)	-4.46% (-221)	-0.74% (-37)	-2.88% (-143)	-17.19% (-850)

Location	Childhood stunting	Childhood underweight	Childhood wasting	Hand-washing	Breast-feeding	Second-hand smoking	Zinc deficiency	Household air pollution	Ambient particulate matter pollution	Antibiotic treatment	Hib vaccine	Pneumococcal vaccine
Colombia	-1.99% (-465)	-1.54% (-360)	-7.47% (-1746)	-0.77% (-181)	-0.09% (-21)	-1.14% (-266)	-0.05% (-11)	-7.62% (-1781)	-4.22% (-987)	-1.32% (-309)	-2.33% (-544)	-15.22% (-3558)
Costa Rica	-1.25% (-32)	-0.82% (-21)	-6.77% (-174)	-0.65% (-17)	-0.56% (-14)	-3% (-77)	-0.06% (-1)	-4.09% (-105)	-1.84% (-47)	-0.35% (-9)	-1.02% (-26)	-16.62% (-427)
El Salvador	-0.53% (0)	-0.2% (0)	-4.11% (-4)	-0.56% (0)	0.13% (0)	-1.29% (-1)	-0.04% (0)	-4.13% (-4)	-1.89% (-2)	-0.3% (0)	-0.52% (0)	-13.13% (-12)
Guatemala	-3.95% (-27)	-1.04% (-7)	-4.75% (-33)	-0.97% (-7)	-0.42% (-3)	-0.35% (-2)	-0.05% (0)	-11.67% (-80)	-3.09% (-21)	-0.8% (-6)	-4.48% (-31)	-13.72% (-94)
Honduras	-2.74% (-175)	-3.09% (-197)	-11.01% (-702)	-1.31% (-83)	-0.25% (-16)	-1.17% (-74)	-0.08% (-5)	-12.09% (-771)	-4.52% (-288)	-1.57% (-100)	-6.77% (-431)	-9.12% (-581)
Mexico	-3.22% (-22)	-2.58% (-18)	-8.91% (-61)	-0.59% (-4)	0.56% (4)	-0.77% (-5)	-0.11% (-1)	-10.86% (-74)	-3.66% (-25)	-0.84% (-6)	-0.3% (-2)	-23.89% (-163)
Nicaragua	-1.6% (-173)	-0.86% (-93)	-6% (-651)	-0.45% (-49)	0.1% (11)	-0.64% (-69)	0.03% (3)	-5.6% (-607)	-4.73% (-513)	-1.45% (-157)	-0.46% (-50)	-17.5% (-1898)
Panama	-1.3% (-12)	-1.6% (-14)	-9.72% (-87)	-1.32% (-12)	-0.15% (-1)	-0.89% (-8)	-0.25% (-2)	-12.25% (-109)	-4.92% (-44)	-1.41% (-13)	-1.2% (-11)	-18.76% (-167)
Venezuela	-3.22% (-5)	-1.61% (-2)	-9.71% (-15)	-0.75% (-1)	0.19% (0)	-2.87% (-4)	-0.38% (-1)	-8% (-12)	-2.05% (-3)	-1.05% (-2)	1.32% (2)	-8.2% (-13)
Tropical Latin America	-1.74% (-19)	-0.58% (-6)	-1.91% (-21)	-0.67% (-7)	-0.14% (-2)	-2.27% (-25)	-0.29% (-3)	-1.67% (-18)	-4.04% (-44)	-1.55% (-17)	-1.38% (-15)	-13.2% (-143)
Brazil	-2.65% (-358)	-1.12% (-152)	-16.04% (-2165)	-1.25% (-169)	-0.06% (-8)	-0.36% (-49)	0.06% (8)	-7.48% (-1009)	-1.6% (-216)	-0.27% (-36)	-0.8% (-108)	-15.17% (-2048)
Paraguay	-2.66% (-345)	-1.11% (-143)	-16.2% (-2102)	-1.2% (-155)	-0.03% (-4)	-0.35% (-46)	0.06% (7)	-7.29% (-946)	-1.67% (-217)	-0.27% (-35)	-0.73% (-95)	-15.44% (-2003)
North Africa and Middle East	-2.42% (-13)	-1.6% (-8)	-12.09% (-63)	-2.71% (-14)	-0.67% (-4)	-0.54% (-3)	0.11% (1)	-12.06% (-63)	0.2% (1)	-0.3% (-2)	-2.77% (-14)	-7.72% (-40)
North Africa and Middle East	-3.22% (-2540)	-2.88% (-2276)	-6.84% (-5398)	-0.43% (-342)	0.05% (38)	-1.56% (-1235)	-0.47% (-373)	-7.88% (-6217)	5.4% (4264)	0.59% (467)	-6.38% (-5033)	-7.45% (-5880)
Afghanistan	-3.22% (-2538)	-2.88% (-2274)	-6.84% (-5394)	-0.43% (-342)	0.05% (38)	-1.56% (-1234)	-0.47% (-373)	-7.88% (-6213)	5.4% (4261)	0.59% (467)	-6.38% (-5030)	-7.45% (-5876)
Algeria	-4.34% (-787)	-4.94% (-897)	-5.43% (-986)	-0.35% (-63)	0.25% (45)	0.33% (60)	-0.25% (-45)	-14.27% (-2591)	2.81% (510)	-2.38% (-431)	-6.2% (-1124)	-6.2% (-1125)
Bahrain	-6.57% (-164)	-7.85% (-196)	-32.17% (-804)	-0.67% (-17)	0.57% (14)	0.06% (2)	-0.62% (-16)	-2.27% (-57)	1.53% (38)	-0.52% (-13)	-7.77% (-194)	1.64% (41)
Egypt	-2.79% (0)	-0.98% (0)	-21.55% (-3)	-0.01% (0)	0.22% (0)	-1.22% (0)	-0.03% (0)	-3.1% (0)	5.89% (1)	-0.55% (0)	-0.62% (0)	-23.85% (-3)
Iran	-1.57% (-309)	-1.09% (-215)	1.16% (229)	-0.63% (-124)	0.28% (55)	-3.66% (-722)	0.19% (37)	-1.17% (-230)	10.45% (2061)	8.9% (1755)	-9.28% (-1830)	1.9% (375)
Iraq	-2.49% (-106)	-1.74% (-74)	-13.01% (-553)	-0.01% (0)	0.41% (18)	-0.1% (-4)	-0.17% (-7)	-0.87% (-37)	0.16% (7)	-0.61% (-26)	-7% (-298)	0% (0)
Jordan	0.73% (33)	-0.98% (-45)	-10.1% (-457)	0.51% (23)	0.71% (32)	-0.03% (-1)	-1.67% (-76)	-3.03% (-137)	8.89% (402)	-1.72% (-78)	-5.32% (-241)	-0.5% (-23)
Kuwait	-1.27% (-5)	-1.16% (-5)	-12.11% (-50)	-0.08% (0)	0.14% (1)	-4.4% (-18)	-0.32% (-1)	-0.09% (0)	4.1% (17)	-0.67% (-3)	-8.62% (-35)	1.64% (7)
Lebanon	-1.28% (0)	-0.26% (0)	-11.65% (-4)	-0.21% (0)	-0.2% (0)	-2.06% (-1)	0.02% (0)	-2.24% (-1)	13.78% (5)	-1.3% (0)	-0.97% (0)	-72.49% (-24)

Location	Childhood stunting	Childhood underweight	Childhood wasting	Hand-washing	Breast-feeding	Second-hand smoking	Zinc deficiency	Household air pollution	Ambient particulate matter pollution	Antibiotic treatment	Hib vaccine	Pneumococcal vaccine
Libya	-2.62% (-1)	-1.1% (-1)	-8.76% (-4)	-0.4% (0)	0.29% (0)	-4.63% (-2)	0.17% (0)	-0.01% (0)	3.97% (2)	-1.58% (-1)	-8.36% (-4)	-21.47% (-10)
Morocco	-1.17% (-2)	-0.26% (-1)	-1.41% (-3)	-0.22% (0)	-0.28% (-1)	-1.2% (-2)	0.2% (0)	-0.98% (-2)	2.18% (4)	-0.59% (-1)	-6.79% (-13)	-18.97% (-37)
Palestine	-1.34% (-46)	-1.46% (-50)	-9.35% (-320)	-1.01% (-35)	0.61% (21)	-1.68% (-58)	-0.02% (-1)	-5.9% (-202)	1.64% (56)	-0.97% (-33)	-5.86% (-201)	-12.43% (-426)
Oman	-0.81% (-3)	-1.47% (-5)	-14.13% (-44)	1.79% (6)	-0.16% (-1)	-1.73% (-5)	0.06% (0)	-1.59% (-5)	1.37% (4)	-3.65% (-11)	-4.34% (-13)	-19.11% (-59)
Qatar	-1.49% (-2)	-2.04% (-2)	-13.07% (-13)	-3.21% (-3)	0.19% (0)	-3.1% (-3)	-0.29% (0)	-13.22% (-13)	2.84% (3)	-0.26% (0)	-8.13% (-8)	-23.59% (-24)
Saudi Arabia	-1.53% (0)	-0.67% (0)	-8.47% (0)	-0.03% (0)	-0.68% (0)	-2.76% (0)	0.27% (0)	-0.02% (0)	11.33% (1)	-0.73% (0)	-0.72% (0)	-42.35% (-2)
Sudan	-2.3% (-9)	-1.86% (-7)	-13.71% (-51)	-1.03% (-4)	-0.12% (0)	-2.29% (-9)	0.06% (0)	-6.98% (-26)	8.84% (33)	-0.3% (-1)	-6.27% (-24)	-16.5% (-62)
Syria	-7.06% (-666)	-6.07% (-573)	-18.68% (-1763)	-0.67% (-63)	-0.96% (-90)	-0.1% (-10)	-1.36% (-128)	-17.49% (-1651)	5.94% (560)	-4.92% (-464)	-4.92% (-464)	-9.72% (-918)
Tunisia	-3.82% (-26)	-1.12% (-8)	1.72% (12)	-0.19% (-1)	0.1% (1)	-2.58% (-17)	-0.52% (-4)	-0.64% (-4)	4.47% (30)	-0.27% (-2)	-4.37% (-30)	1.68% (11)
Turkey	-1.87% (-5)	-0.32% (-1)	-6.93% (-18)	-0.04% (0)	1.06% (3)	-2.07% (-6)	-0.32% (-1)	-2.96% (-8)	-0.14% (0)	-0.53% (-1)	-8.93% (-24)	1.54% (4)
United Arab Emirates	-0.94% (-74)	-1.16% (-92)	-9.68% (-768)	-0.64% (-51)	-0.9% (-71)	-3.59% (-284)	-0.09% (-7)	-3.79% (-301)	2.4% (191)	-0.11% (-9)	-4.94% (-392)	-13.77% (-1092)
Yemen	-2.18% (-1)	0.09% (0)	-22.16% (-6)	-0.35% (0)	-0.47% (0)	-2.46% (-1)	-0.92% (0)	-2.11% (-1)	9.8% (3)	-0.32% (0)	-1.2% (0)	-41.25% (-11)
South Asia	-5.63% (-365)	-1.61% (-104)	3.27% (212)	-0.13% (-8)	0.18% (12)	-2.35% (-152)	-1.94% (-125)	-14.62% (-947)	5.17% (335)	-3.29% (-213)	-5.62% (-364)	-13.97% (-905)
South Asia	-2.03% (-11036)	-4% (-21729)	-8.9% (-48289)	-1.23% (-6676)	0.09% (506)	-0.55% (-2975)	-0.34% (-1866)	-12.43% (-67431)	5% (27122)	-3.11% (-16860)	-4.79% (-26017)	-3.25% (-17658)
Bangladesh	-2.03% (-11036)	-4% (-21729)	-8.9% (-48289)	-1.23% (-6676)	0.09% (506)	-0.55% (-2975)	-0.34% (-1866)	-12.43% (-67431)	5% (27122)	-3.11% (-16860)	-4.79% (-26017)	-3.25% (-17658)
Bhutan	-3.56% (-2261)	-4.45% (-2821)	-9.41% (-5971)	-0.38% (-242)	-1.29% (-819)	-0.23% (-148)	-0.29% (-186)	-7.52% (-4772)	7.33% (4654)	-3.01% (-1913)	-6.44% (-4085)	-19.3% (-12245)
India	-4.89% (-18)	-1.85% (-7)	-0.47% (-2)	-0.55% (-2)	0.09% (0)	-0.77% (-3)	-0.16% (-1)	-16.18% (-60)	4.39% (16)	-2.44% (-9)	-6.16% (-23)	1.25% (5)
Nepal	-1.64% (-6799)	-4.41% (-18228)	-10.28% (-42533)	-1.39% (-5739)	0.32% (1318)	-0.52% (-2172)	-0.34% (-1401)	-13.42% (-55537)	5.01% (20749)	-3.43% (-14209)	-4.32% (-17870)	1.49% (6171)
Pakistan	-4.13% (-638)	-4.29% (-663)	-11.8% (-1825)	-2% (-309)	0.07% (10)	-0.46% (-71)	-0.1% (-15)	-8.05% (-1244)	5.79% (896)	-3.98% (-615)	-5.64% (-872)	-14.56% (-2250)
Sub-Saharan Africa	-2.66% (-1319)	-0.02% (-10)	4.11% (2042)	-0.77% (-383)	-0.01% (-4)	-1.17% (-581)	-0.53% (-263)	-11.73% (-5819)	1.63% (807)	-0.23% (-114)	-8.5% (-4215)	-39.74% (-19718)
Central Sub-Saharan Africa	-3.9% (-18365)	-4.44% (-20950)	-12.3% (-57998)	-0.08% (-400)	-0.36% (-1715)	-0.82% (-3868)	-0.44% (-2061)	-6.74% (-31753)	4.52% (21323)	-1.19% (-5608)	-6.93% (-32653)	-10.89% (-51316)
Angola	-4.49% (-2387)	-6.65% (-3531)	-25.89% (-13749)	-0.02% (-10)	-1.05% (-558)	-1.29% (-687)	-2.26% (-1199)	-10.23% (-5434)	1.57% (834)	-1.56% (-831)	-7.22% (-3834)	-17.4% (-9242)
Central African Republic	-8.12% (-1041)	-7.47% (-957)	-21.22% (-2722)	-0.39% (-50)	0.12% (15)	-0.7% (-90)	-4.25% (-544)	-27.72% (-3555)	-1.88% (-241)	-1.95% (-250)	-6.78% (-869)	-7.27% (-932)

Location	Childhood stunting	Childhood underweight	Childhood wasting	Hand-washing	Breast-feeding	Second-hand smoking	Zinc deficiency	Household air pollution	Ambient particulate matter pollution	Antibiotic treatment	Hib vaccine	Pneumococcal vaccine
Congo	-4.96% (-179)	-0.97% (-35)	-4.21% (-152)	0.01% (0)	-2.54% (-92)	-1.42% (-51)	-0.1% (-4)	-2.99% (-108)	5.82% (210)	-0.29% (-11)	-6.33% (-228)	-16.33% (-590)
Democratic Republic of the Congo	-2.43% (-38)	-0.99% (-16)	-3.48% (-55)	-0.14% (-2)	0.17% (3)	-0.26% (-4)	-0.5% (-8)	-17.14% (-270)	-1.78% (-28)	-3.52% (-56)	-9% (-142)	-15.3% (-241)
Equatorial Guinea	-3.2% (-1105)	-7.26% (-2505)	-31.1% (-10727)	0.14% (50)	-1.42% (-491)	-1.56% (-537)	-1.86% (-640)	-4.08% (-1408)	2.52% (870)	-1.46% (-503)	-7.9% (-2726)	-32.75% (-11298)
Gabon	-5.77% (-17)	-4.03% (-12)	-17.92% (-53)	-1% (-3)	1.47% (4)	0.4% (1)	-0.78% (-2)	-15.36% (-46)	4.53% (13)	-2.07% (-6)	-2.6% (-8)	1.25% (4)
Eastern Sub-Saharan Africa	-2.05% (-6)	-2.01% (-6)	-13.5% (-40)	-1.73% (-5)	0.63% (2)	-2.04% (-6)	-0.1% (0)	-15.84% (-47)	3.12% (9)	-1.87% (-6)	-10.06% (-30)	3.47% (10)
Burundi	-4.18% (-8591)	-5.03% (-10329)	-11.97% (-24587)	-0.2% (-402)	-0.3% (-622)	-0.93% (-1903)	-0.2% (-404)	-4.98% (-10230)	1.92% (3941)	-1.51% (-3108)	-7.27% (-14934)	-15.99% (-32850)
Comoros	-6.11% (-281)	-6.71% (-309)	-19.61% (-903)	-0.28% (-13)	-0.33% (-15)	-2.92% (-134)	2.19% (101)	-5.05% (-232)	-0.27% (-13)	-6.16% (-283)	-13.22% (-608)	-41.03% (-1889)
Djibouti	-6.76% (-19)	-5.05% (-14)	-8.06% (-23)	-1.38% (-4)	-0.25% (-1)	-2.52% (-7)	0.11% (0)	-12.34% (-35)	-1.27% (-4)	-0.6% (-2)	-4.94% (-14)	1.77% (5)
Eritrea	-1.8% (-12)	-3.17% (-22)	-8.1% (-55)	-1.07% (-7)	0.14% (1)	-3.43% (-23)	-0.73% (-5)	-9.55% (-65)	6.17% (42)	0.92% (6)	-4.22% (-29)	-9.32% (-63)
Ethiopia	-5.32% (-110)	-7.2% (-148)	-13.94% (-287)	-0.41% (-8)	-0.32% (-7)	-1.26% (-26)	0.14% (3)	-19.13% (-394)	3.04% (63)	-0.41% (-8)	-10.37% (-213)	-29.54% (-608)
Kenya	-1.67% (-932)	-5.26% (-2939)	-10.89% (-6085)	-0.21% (-119)	0.6% (336)	-0.22% (-124)	0.07% (40)	-5.08% (-2841)	5.27% (2947)	-1.95% (-1090)	-5.49% (-3071)	-9.23% (-5158)
Madagascar	-4.06% (-726)	-2.45% (-439)	-10.15% (-1817)	-0.4% (-72)	-0.33% (-60)	-1.18% (-212)	-0.08% (-14)	-3.85% (-689)	-0.09% (-16)	-1.17% (-209)	-7.79% (-1394)	-21.51% (-3850)
Malawi	-2.49% (-283)	-1.9% (-217)	36.03% (4101)	0.12% (13)	-0.24% (-28)	-2.35% (-267)	0.01% (1)	-3.24% (-368)	1.51% (172)	1.85% (211)	-8.2% (-934)	-21.21% (-2414)
Mozambique	-6.93% (-992)	-5.42% (-776)	-26.04% (-3728)	0.84% (121)	-2.01% (-287)	-1.07% (-153)	-0.11% (-16)	-3.78% (-541)	-0.43% (-61)	-1.36% (-194)	-8.23% (-1179)	-21.05% (-3013)
Rwanda	-6.26% (-982)	-7.41% (-1163)	-23.39% (-3670)	-0.56% (-88)	0.13% (20)	-0.63% (-99)	-1.62% (-255)	-6.48% (-1017)	-1.13% (-178)	-1.11% (-174)	-6.88% (-1080)	-12.94% (-2030)
Somalia	-4.84% (-437)	-6.48% (-584)	-27.48% (-2477)	-0.29% (-26)	-0.35% (-32)	-0.31% (-28)	-1.26% (-113)	-5.63% (-508)	-0.25% (-23)	-5.37% (-484)	-7.95% (-717)	-22.05% (-1988)
South Sudan	-0.98% (-65)	-3.85% (-255)	-7.47% (-494)	-0.65% (-43)	-0.05% (-3)	-1.3% (-86)	0.15% (10)	-5.17% (-341)	1.28% (84)	0.19% (12)	-4.35% (-287)	2.31% (152)
Tanzania	-8.21% (-655)	-7.04% (-562)	-13.81% (-1101)	0.04% (3)	-0.86% (-69)	-1.44% (-114)	-0.06% (-4)	-5.65% (-450)	3.54% (282)	-0.4% (-32)	-3.55% (-283)	2.29% (182)
Uganda	-4.59% (-1514)	-4.69% (-1548)	-13.32% (-4391)	-0.12% (-40)	-0.91% (-300)	-0.65% (-216)	-0.21% (-69)	-5.23% (-1725)	-0.07% (-24)	-0.73% (-240)	-8.07% (-2661)	-18.89% (-6228)
Zambia	-5.77% (-821)	-5.52% (-785)	-15.22% (-2164)	-0.77% (-110)	0.28% (40)	-1.72% (-244)	-0.31% (-44)	-6.01% (-855)	4.82% (685)	-2.57% (-366)	-7.88% (-1120)	-12.09% (-1718)
Southern Sub-Saharan Africa	-6.41% (-762)	-4.8% (-570)	-12.56% (-1493)	-0.06% (-8)	-1.83% (-218)	-1.42% (-169)	-0.32% (-38)	-1.42% (-169)	-0.14% (-16)	-2.15% (-255)	-7.42% (-882)	-15.42% (-1834)
Botswana	-2.38% (-452)	-1.26% (-239)	-9.8% (-1859)	0.65% (124)	-0.38% (-71)	-2.02% (-384)	-0.02% (-3)	-8.68% (-1646)	-1.34% (-255)	-1.8% (-341)	-5.09% (-966)	-25.77% (-4888)

Location	Childhood stunting	Childhood underweight	Childhood wasting	Hand-washing	Breast-feeding	Second-hand smoking	Zinc deficiency	Household air pollution	Ambient particulate matter pollution	Antibiotic treatment	Hib vaccine	Pneumococcal vaccine
Lesotho	-1.42% (-4)	-1.05% (-3)	-3.78% (-11)	-0.63% (-2)	0.02% (0)	-0.6% (-2)	-0.14% (0)	-12.78% (-39)	0.38% (1)	-1.55% (-5)	-11.03% (-33)	-20.54% (-62)
Namibia	-9.08% (-69)	-2.88% (-22)	-19.23% (-147)	-0.11% (-1)	-1.19% (-9)	-2.57% (-20)	-0.28% (-2)	-10.59% (-81)	-0.5% (-4)	-0.7% (-5)	-7.64% (-58)	-21.75% (-166)
South Africa	-2.78% (-14)	-3.13% (-16)	-11.55% (-60)	-1.48% (-8)	-1.37% (-7)	-1.87% (-10)	-0.05% (0)	-12.09% (-63)	-0.65% (-3)	-6.01% (-31)	-10.47% (-55)	-26.6% (-139)
Swaziland	-2.06% (-245)	-1.55% (-184)	-9.93% (-1183)	0.39% (46)	-0.1% (-12)	-2.27% (-270)	-0.03% (-3)	-9.76% (-1163)	-1.87% (-223)	-1.74% (-207)	-1.04% (-124)	-23.01% (-2742)
Zimbabwe	-4.43% (-24)	-2.49% (-14)	-8.85% (-49)	-1.6% (-9)	-1.15% (-6)	-1.62% (-9)	-0.02% (0)	-21.57% (-118)	-2% (-11)	-1.61% (-9)	-10.19% (-56)	-22.24% (-122)
Western Sub-Saharan Africa	-1.91% (-94)	0.02% (1)	-8.31% (-408)	1.97% (97)	-0.76% (-37)	-1.5% (-74)	0.06% (3)	-3.7% (-182)	-0.3% (-15)	-1.71% (-84)	-17.12% (-841)	-39.79% (-1955)
Benin	-3.58% (-6930)	-3.53% (-6846)	-9.18% (-17788)	-0.06% (-111)	-0.24% (-463)	-0.46% (-893)	-0.23% (-455)	-7.45% (-14435)	8.67% (16798)	-0.68% (-1327)	-6.76% (-13091)	-4.95% (-9596)
Burkina Faso	-1.22% (-72)	-3.51% (-206)	-19.02% (-1116)	-0.38% (-22)	-0.49% (-29)	-1.39% (-82)	-0.25% (-14)	-5.68% (-333)	9.97% (585)	-1.05% (-62)	-9.76% (-573)	-14.03% (-823)
Cameroon	-5.92% (-772)	-9.12% (-1188)	-25.65% (-3343)	-0.53% (-69)	-0.66% (-86)	-0.74% (-96)	0.4% (52)	-7.66% (-998)	11.07% (1443)	-2.17% (-283)	-7.94% (-1035)	-21.4% (-2789)
Cape Verde	-2.64% (-262)	-2.39% (-237)	-8.62% (-855)	-0.24% (-24)	-0.56% (-56)	-1.08% (-107)	-0.52% (-51)	-10.21% (-1013)	8.07% (800)	-0.05% (-5)	-7.84% (-777)	-18.51% (-1835)
Chad	-2.24% (-2)	-1.96% (-2)	-15.65% (-16)	-2.05% (-2)	-1.05% (-1)	-0.91% (-1)	-0.15% (0)	-10.87% (-11)	4.4% (4)	-0.86% (-1)	-8.33% (-8)	1.59% (2)
Cote d'Ivoire	-2.13% (-207)	-2.7% (-261)	-14.4% (-1395)	0.85% (83)	0.5% (48)	-0.25% (-25)	0.66% (64)	-4.6% (-446)	9.59% (928)	-0.68% (-66)	-4.57% (-442)	2.71% (262)
The Gambia	-0.83% (-79)	-0.15% (-14)	0.2% (19)	-0.2% (-19)	-0.63% (-59)	-0.03% (-3)	-0.42% (-40)	-5.75% (-545)	12.2% (1157)	-2.75% (-261)	-9.55% (-905)	-1.85% (-175)
Ghana	-0.78% (-5)	-0.58% (-3)	-2.23% (-13)	-0.48% (-3)	-1.04% (-6)	-2.74% (-16)	-0.56% (-3)	-3.96% (-24)	7.3% (43)	-3.12% (-19)	-1.99% (-12)	-33.25% (-198)
Guinea	-3.29% (-177)	-4.35% (-234)	-21.58% (-1161)	0.96% (52)	-1.07% (-58)	-0.65% (-35)	-0.84% (-45)	-10.7% (-576)	8.11% (436)	-0.22% (-12)	-8.04% (-432)	-17.1% (-920)
Guinea-Bissau	-2.45% (-249)	-4.57% (-464)	-17.37% (-1764)	-0.22% (-23)	-0.3% (-31)	-1.06% (-108)	-0.1% (-10)	-3.35% (-340)	7.41% (753)	-0.71% (-72)	-4.56% (-463)	2.21% (225)
Liberia	-4.07% (-36)	-3.36% (-30)	-17.74% (-156)	0.04% (0)	-0.04% (0)	0.46% (4)	-0.8% (-7)	-4.06% (-36)	6.22% (55)	-0.09% (-1)	-7.27% (-64)	-16.32% (-143)
Mali	-6.75% (-148)	-4.07% (-89)	-10.3% (-226)	0.01% (0)	-1.79% (-39)	-0.82% (-18)	-0.85% (-19)	-4.45% (-97)	5.14% (113)	-0.42% (-9)	-5.62% (-123)	-9.44% (-207)
Mauritania	-5.7% (-420)	-7.28% (-537)	-26.54% (-1958)	-0.1% (-7)	-0.56% (-42)	-0.03% (-3)	0.66% (49)	-3.39% (-250)	9.19% (678)	-1.41% (-104)	-6.23% (-460)	-11.56% (-853)
Niger	-4.57% (-46)	-3.63% (-36)	-12.24% (-122)	-1.23% (-12)	-0.09% (-1)	-1.87% (-19)	0.28% (3)	-16.77% (-168)	6.34% (63)	-0.54% (-5)	-5.66% (-57)	-7.93% (-79)
Nigeria	-4.28% (-796)	-5.58% (-1036)	-6.58% (-1223)	-0.63% (-118)	-0.97% (-181)	-0.04% (-7)	-0.04% (-8)	-1.73% (-321)	8.81% (1636)	0.26% (48)	-8.09% (-1502)	-3.1% (-575)
Sao Tome and Principe	-3.96% (-3445)	-2.31% (-2008)	-2.49% (-2164)	0.03% (25)	0.36% (314)	-0.18% (-154)	-0.3% (-262)	-9.82% (-8546)	8.12% (7073)	-0.54% (-472)	-6.16% (-5366)	0.46% (399)
Senegal	-5.97% (-4)	-0.9% (-1)	-7.03% (-4)	-1% (-1)	0.92% (1)	-0.52% (0)	-0.27% (0)	-18.04% (-11)	-0.31% (0)	-1.03% (-1)	-6.98% (-4)	-18.58% (-12)

Location	Childhood stunting	Childhood underweight	Childhood wasting	Hand-washing	Breast-feeding	Second-hand smoking	Zinc deficiency	Household air pollution	Ambient particulate matter pollution	Antibiotic treatment	Hib vaccine	Pneumococcal vaccine
Sierra Leone	-2.82% (-135)	-3.06% (-147)	-18.15% (-871)	0.38% (18)	-1.68% (-81)	-1.51% (-73)	-0.1% (-5)	-6.18% (-297)	7.15% (343)	1.01% (49)	-8.64% (-415)	-19.25% (-924)
Togo	0.45% (22)	-4.59% (-221)	-19.22% (-924)	0.26% (13)	-1.78% (-86)	-1.7% (-82)	-3.12% (-150)	-4.87% (-234)	9.26% (445)	-0.35% (-17)	-9.18% (-441)	-21.09% (-1014)

Appendix Table 15. The number needed to treat to prevent one under-5 death due to LRI in 2016

The number needed to treat is the effect number of children that would need to be reached in an intervention program that removes exposure to the risk factor to prevent one death. For example, for every 1697 children that are no longer wasted (less than 2 standard deviation below the global mean), one death due to LRI would be averted. Numbers in parentheses are 95% uncertainty intervals.

Location	Childhood wasting	Household air pollution	Ambient particulate matter pollution	Antibiotic treatment	Pneumococcal conjugate vaccine	Childhood stunted	Hib vaccine	Childhood under-weight	Hand-washing	Breast-feeding	Second-hand smoking
Global	1697 (1384-2439)	2527 (1999-3410)	2907 (2348-3736)	3204 (2002-7470)	4980 (4233-6059)	7157 (2788-87779)	8175 (5229-18492)	8433 (4690-12470)	8898 (6696-13743)	11417 (8353-17576)	11795 (7486-22946)
Southeast Asia, East Asia, and Oceania	3671 (2898-5733)	6996 (5196-9842)	9033 (6512-13239)	6728 (4113-15856)	10070 (8279-12547)	17693 (6341-266394)	19844 (12629-43795)	23579 (12288-37950)	40769 (28848-66769)	21695 (16077-31456)	13328 (8863-25398)
China	10764 (7544-22728)	18599 (13600-26412)	12880 (10061-17287)	13515 (8117-33401)	38371 (30880-49045)	60062 (19065-3081115)	120806 (72792-290841)	119836 (57116-234506)	146918 (104165-242513)	34582 (26624-47094)	21163 (14965-34950)
North Korea	2517 (1425-5126)	3081 (1764-5848)	5447 (3080-10660)	4494 (1876-17200)	4009 (2396-7509)	10379 (3502-364846)	126952 (53223-482571)	14481 (6853-34077)	45410 (23864-111894)	16152 (8869-34315)	6681 (3560-14115)
Taiwan	89382 (53842-210895)	1745145 (758296-5813768)	160585 (107562-273274)	124580 (65618-369045)	779813 (506350-1199469)	1016117 (291169-57553736)	2954311 (1042067-15062811)	2126354 (883267-5557544)	2423218 (1315916-5213355)	311312 (209064-518785)	218816 (144237-374177)
Cambodia	811 (589-1279)	1106 (791-1668)	2280 (1509-3971)	1682 (903-4519)	4708 (3312-7078)	4204 (1471-42770)	8739 (4629-26036)	4738 (2539-8328)	8024 (4965-14485)	7766 (4848-13606)	3214 (2010-6354)
Indonesia	2532 (1995-3470)	9289 (6546-13715)	12560 (7070-28259)	5938 (3462-14131)	5173 (4138-6441)	12444 (4775-156887)	7858 (5096-16865)	15899 (8365-25064)	33372 (23838-52590)	18829 (12734-30992)	12813 (7669-29656)
Laos	474 (261-1139)	630 (338-1388)	1343 (690-3108)	865 (344-3647)	2811 (1485-6392)	2012 (708-35241)	2702 (1183-9094)	2548 (1143-6607)	3867 (1941-9759)	2910 (1470-6744)	2192 (1006-6608)
Malaysia	16062 (10961-26843)	2522324 (817467-10258318)	66693 (38213-138789)	34761 (16921-103024)	30715 (21850-44976)	124750 (39774-1997875)	1048377 (415613-4700305)	131886 (66302-249855)	942312 (475131-2141718)	105573 (62872-192032)	78648 (46673-161098)
Maldives	29649 (19020-54678)	495203 (195278-1796338)	82645 (47368-176424)	59183 (28567-195716)	52690 (36791-83498)	169078 (57770-6202356)	502563 (219417-1737477)	183216 (86479-404856)	344287 (191193-725435)	287451 (166550-594856)	99343 (57488-194506)
Mauritius	9064 (6184-14403)	394183 (137663-1750958)	43309 (23384-110590)	19031 (9743-54434)	35038 (25355-50665)	99240 (28324-9174541)	633743 (240144-2348535)	109965 (50151-240670)	613139 (304880-1344216)	52753 (34101-97191)	40084 (25318-76899)
Myanmar	1379 (791-2845)	1957 (1131-3700)	2528 (1461-4723)	2545 (1042-9605)	3184 (1845-5664)	7774 (2578-131330)	26577 (10790-96128)	8808 (4108-20773)	10315 (5425-23594)	7593 (3934-15955)	7100 (3720-18165)
Philippines	1990 (1465-3347)	3893 (2606-6086)	6080 (3974-10697)	3657 (2084-9541)	9063 (6486-13090)	8359 (3098-80319)	11417 (6361-29659)	10776 (5420-20783)	26139 (15522-51691)	17546 (12359-27743)	8365 (5099-17033)
Sri Lanka	19687 (11518-35858)	47342 (26963-99422)	64370 (37828-127231)	44305 (18938-159454)	39246 (23937-69193)	163944 (50031-2905446)	9363323 (2777976-63989171)	135674 (58763-322057)	414054 (195118-1002172)	193208 (100012-448790)	148924 (78494-386944)

Location	Childhood wasting	Household air pollution	Ambient particulate matter pollution	Antibiotic treatment	Pneumococcal conjugate vaccine	Childhood stunted	Hib vaccine	Childhood under-weight	Hand-washing	Breast-feeding	Second-hand smoking
Seychelles	8111 (5442-14855)	427117 (155410-1905623)	25413 (12159-146880)	11184 (5994-31547)	9898 (7571-13392)	74413 (21599-2910169)	2039212 (611176-12355089)	134336 (63495-281992)	234795 (128804-533351)	33723 (22312-58344)	25784 (15697-50428)
Thailand	16249 (10328-30304)	117441 (57764-307714)	47601 (28166-91985)	32223 (15280-104933)	27304 (18636-43389)	117310 (37570-5497410)	24854 (15058-52398)	133408 (64634-321594)	172356 (98747-359385)	70230 (45324-124817)	73286 (42231-156367)
Timor-Leste	1061 (619-2004)	1790 (1037-3506)	5390 (2846-12815)	2381 (957-9035)	2281 (1295-4311)	4336 (1557-61350)	4599 (2248-12558)	5234 (2487-11367)	13502 (7065-32638)	10004 (5170-24582)	7391 (3482-25426)
Vietnam	4800 (3121-8856)	11388 (6916-21002)	11660 (7303-20522)	8353 (4130-25800)	7246 (5180-10929)	28139 (9138-576849)	23806 (12566-65505)	37029 (17807-75877)	43578 (26032-82638)	23775 (14672-45706)	18433 (10788-38867)
American Samoa	11963 (7513-23258)	73713 (37481-168040)	601197 (82327-Inf)	19631 (9710-58499)	17928 (12500-26407)	124080 (35456-8123238)	16311 (10213-33380)	248665 (110084-647618)	308864 (174601-681388)	62798 (39069-119246)	85573 (41526-298222)
Federated States of Micronesia	4333 (2290-10338)	12365 (6514-30952)	31290 (10985-345994)	7050 (2817-29349)	27018 (13892-66633)	34578 (10082-1605261)	16760 (7710-52671)	64856 (27570-202954)	71299 (33202-184188)	28356 (13817-73244)	28863 (13507-92023)
Fiji	1508 (807-3237)	7043 (3419-16766)	13830 (5227-61786)	2705 (1111-10222)	15340 (7593-35456)	21453 (5868-1718103)	13670 (6134-45952)	24622 (10027-64789)	41996 (20091-107610)	10061 (5227-22110)	8956 (4482-24163)
Guam	6305 (3910-12785)	79228 (39136-203626)	41314 (14407-Inf)	9674 (4723-30044)	8796 (6014-13796)	81668 (22811-6031115)	8037 (5083-17288)	164238 (68469-477560)	189109 (99863-431290)	27930 (16676-56209)	57953 (25453-258228)
Kiribati	1598 (799-3982)	2269 (1167-5037)	77159 (11805-Inf)	3176 (1170-14142)	20807 (9075-63089)	10363 (2912-429632)	23413 (9369-99285)	22672 (9335-65491)	17045 (7799-46467)	13772 (6753-35416)	5600 (2768-14183)
Marshall Islands	2996 (1510-7807)	10752 (5215-28090)	18055 (5080-Inf)	5004 (1859-22568)	8713 (4525-20697)	22204 (6740-1032995)	7407 (3371-22452)	40381 (17391-134612)	43849 (19504-121526)	19508 (9328-51708)	15243 (6807-42026)
Northern Mariana Islands	57942 (28059-163569)	355435 (152786-1059157)	239255 (86936-3157886)	78908 (29501-350056)	72307 (37287-158139)	612996 (162689-27585498)	65735 (32973-177944)	1231497 (497867-4511470)	1439701 (646012-4242030)	283128 (130834-800388)	238409 (97013-962129)
Papua New Guinea	695 (377-1486)	904 (504-1851)	3333 (1567-9600)	1203 (484-4591)	1878 (1067-4029)	2339 (887-27724)	2361 (1182-7365)	3212 (1539-7633)	4914 (2549-11498)	5586 (2821-14729)	2326 (1163-6051)
Samoa	12169 (5266-40354)	18034 (8269-48126)	424704 (67856-Inf)	15544 (5063-75782)	14488 (6838-36049)	107054 (27174-8559311)	18761 (8425-57116)	226207 (82790-753791)	204573 (81129-699034)	66784 (28158-210140)	25061 (10853-69354)
Solomon Islands	2573 (1486-5838)	2883 (1700-5637)	18630 (7724-76875)	4075 (1719-14919)	7589 (4075-15525)	12863 (4201-200755)	15215 (7208-49712)	23103 (10742-56192)	29469 (15967-71981)	21014 (10985-50972)	9254 (5012-20725)
Tonga	4933 (2500-12086)	11833 (5782-29707)	203840 (35104-Inf)	8195 (3071-34043)	7502 (4047-15409)	56934 (16736-2807697)	986605 (286152-5780211)	140459 (53329-431432)	75114 (37349-199300)	32231 (15774-82306)	26991 (12012-96936)

Location	Childhood wasting	Household air pollution	Ambient particulate matter pollution	Antibiotic treatment	Pneumococcal conjugate vaccine	Childhood stunted	Hib vaccine	Childhood under-weight	Hand-washing	Breast-feeding	Second-hand smoking
Vanuatu	1608 (845-3915)	2061 (1136-4251)	11549 (4658-53176)	2626 (1051-9954)	2471 (1379-4557)	8850 (2868-158343)	4677 (2380-14384)	13756 (6124-35907)	13039 (6735-30035)	9747 (4928-23169)	13719 (5818-57326)
Central Europe, Eastern Europe, and Central Asia	3288 (2324-5487)	15829 (8750-30680)	6432 (4395-10149)	5639 (2957-15322)	7347 (5454-10044)	20864 (6824-533869)	39078 (21792-94569)	43692 (21116-81742)	56683 (35701-101174)	15564 (10330-25981)	12853 (8405-22663)
Armenia	3734 (2362-7108)	20234 (8104-91304)	8667 (5562-16144)	5404 (2743-15673)	11493 (7760-17090)	21086 (7114-571089)	81677 (35438-276607)	41631 (17778-97236)	73584 (41289-146863)	15811 (9862-27448)	7638 (4934-13055)
Azerbaijan	1073 (641-2178)	6398 (2245-36417)	2256 (1374-4163)	1746 (785-5815)	3979 (2362-7330)	7290 (2302-136111)	6383 (2678-24388)	13878 (5983-36810)	21578 (10425-50442)	4147 (2418-7935)	2358 (1456-4472)
Georgia	11760 (6902-28745)	21864 (11588-68334)	27671 (16147-55993)	17677 (7949-61352)	19939 (13215-30899)	74865 (23120-3299979)	63087 (30031-185475)	257304 (111766-671121)	248144 (131558-596318)	49708 (31944-91811)	28664 (17198-57618)
Kazakhstan	6406 (4032-12855)	37873 (14358-193097)	18933 (11040-37366)	9671 (4599-30974)	28207 (17916-46924)	67097 (19558-2550702)	82397 (33852-319948)	120862 (53020-280842)	130589 (65679-292539)	29934 (18026-57486)	13572 (8298-24317)
Kyrgyzstan	1675 (1112-3519)	4764 (2719-9691)	5271 (3087-11043)	2766 (1521-7532)	2884 (2175-3940)	11536 (3560-580120)	8251 (4321-22990)	27873 (12930-61143)	33187 (20611-62203)	11027 (6912-19936)	9519 (4982-28351)
Mongolia	2500 (1470-6240)	3303 (2002-6152)	4285 (2643-7223)	3410 (1637-10842)	3485 (2407-5294)	13402 (4228-365131)	24850 (10759-85530)	31085 (14094-73256)	17479 (10263-34099)	12551 (7600-23019)	8038 (4339-18707)
Tajikistan	657 (389-1210)	1837 (927-5155)	1249 (761-2265)	1275 (574-4433)	1220 (768-2179)	3245 (1172-66019)	9104 (4140-31541)	4841 (2249-10782)	8831 (4874-19278)	4622 (2521-10111)	7004 (3890-14507)
Turkmenistan	840 (510-1647)	194618 (48949-2164524)	1847 (1112-3303)	1588 (730-5330)	1419 (919-2279)	7322 (2133-237401)	7888 (3578-25446)	11473 (5206-26247)	17262 (8439-40980)	4777 (2702-9727)	2840 (1627-5327)
Uzbekistan	1034 (578-2343)	5742 (1803-35066)	1700 (927-3444)	1640 (653-6454)	2489 (1407-4814)	6784 (2006-183258)	177296 (54700-1015264)	17546 (7217-48048)	17567 (8368-47751)	4681 (2421-11108)	5425 (2717-14389)
Albania	4113 (2496-7738)	14670 (7908-31369)	18172 (9091-47945)	9362 (3781-39183)	1777581 (662555-6469628)	20794 (7345-196586)	1554943 (575046-8134310)	45262 (20001-112149)	160464 (75325-389776)	19966 (11808-38731)	11120 (6508-22084)
Bosnia and Herzegovina	59977 (39075-106448)	141420 (87636-244460)	104752 (70536-164379)	115378 (55094-413393)	84040 (60066-121059)	470085 (147721-10448059)	770273 (340693-2353230)	783551 (393130-1702060)	3496986 (1956098-7510777)	196961 (126911-338509)	133860 (84174-247768)
Bulgaria	8931 (4882-22471)	79206 (39476-194224)	20834 (11616-41630)	15921 (6477-66758)	169442 (83697-426877)	72163 (22278-2452013)	110139 (45904-365735)	123340 (51606-306505)	493175 (242708-1161235)	26085 (15461-49225)	17383 (10033-32742)
Croatia	114001 (73974-229096)	1020131 (583077-1993362)	296466 (181227-589017)	188001 (94850-609581)	150100 (113859-207069)	1102338 (319887-39744883)	2355568 (1035921-7308869)	2487572 (1121728-5588215)	6060492 (3276148-13601311)	300001 (208473-452551)	246926 (164169-433180)

Location	Childhood wasting	Household air pollution	Ambient particulate matter pollution	Antibiotic treatment	Pneumococcal conjugate vaccine	Childhood stunted	Hib vaccine	Childhood under-weight	Hand-washing	Breast-feeding	Second-hand smoking
Czech Republic	87678 (54428-199024)	3634548 (1837499-8564343)	210672 (122789-445694)	131186 (62383-447860)	104559 (75886-153032)	1750182 (454513-237714794)	5234076 (1860534-25309222)	3099022 (1349323-8187192)	4952383 (2534232-11643855)	268310 (176445-444209)	189681 (121544-352435)
Hungary	52843 (29787-146571)	283964 (149255-648516)	84149 (50989-161694)	62392 (28580-226931)	986797 (572738-1887237)	693532 (184035-127326246)	3938016 (1528336-16513624)	1445022 (588464-4060025)	2253957 (1139617-5032716)	90784 (58871-151052)	91726 (55279-174420)
Macedonia	14867 (8354-33909)	52544 (30215-102234)	31465 (18466-60847)	29421 (12533-120827)	22361 (14161-37993)	145462 (42068-5050371)	164448 (70412-569184)	333843 (143836-857842)	827675 (385921-2142532)	57745 (33883-109457)	31557 (18574-61745)
Montenegro	30633 (18152-67754)	88453 (51848-171263)	93098 (52386-207638)	97957 (39768-383243)	48196 (31180-81638)	211653 (67632-4766185)	462751 (210451-1519700)	646887 (289710-1558086)	1571629 (794986-3925272)	123501 (76684-231697)	66162 (39550-121212)
Poland	57576 (32680-139712)	880118 (426935-2107904)	129687 (68963-280751)	100644 (39792-395789)	80141 (48700-141620)	566776 (146716-35657355)	3801098 (1503514-15527640)	1337130 (554503-3398892)	3266536 (1524746-8729052)	214782 (126867-403695)	159150 (81027-364128)
Romania	4304 (2870-8890)	24895 (14699-47555)	11614 (7137-24403)	7208 (3719-21754)	5725 (4361-7705)	26200 (8492-475336)	76813 (32309-309852)	91046 (39859-224661)	179872 (95915-388168)	12599 (9056-18979)	10472 (6860-17910)
Serbia	22470 (15511-37667)	80015 (51598-133092)	78065 (46677-157823)	54068 (26254-170687)	38025 (27779-52878)	214525 (65767-9559019)	604439 (261837-1961205)	245386 (114484-484022)	1824178 (1045855-3627967)	96862 (65693-149286)	54713 (36929-91527)
Slovakia	20624 (12731-43054)	662153 (337210-1532232)	57029 (31540-116353)	37348 (16404-125125)	1507821 (557400-5885892)	214036 (62746-6317968)	1245807 (503875-5003210)	488761 (219109-1186903)	1399335 (737115-3274280)	83804 (52141-143082)	47175 (27905-85650)
Slovenia	184063 (114111-389105)	2821588 (1459088-6103188)	513369 (296647-1116370)	301537 (143015-951517)	2822713 (1569701-5493072)	1870992 (542061-77791559)	4236935 (1908963-15273232)	4331691 (1823216-10226968)	12006592 (5996229-27835795)	638148 (428187-1059173)	414592 (263970-714924)
Belarus	56554 (32721-132349)	3100275 (932271-24378875)	129762 (74529-286423)	75296 (33825-253863)	64025 (40255-104150)	672471 (177135-169145395)	2665313 (451264-36827005)	1703048 (707554-4373858)	1597933 (811027-3870042)	200001 (123463-370392)	107670 (63893-213064)
Estonia	47003 (27453-117330)	273647 (86248-1958189)	441361 (134377-3933105)	61373 (28876-199850)	52245 (35033-83888)	502381 (149130-21616529)	634290 (275145-2209817)	1594435 (692921-4370483)	1489698 (781231-3465243)	145818 (89204-262880)	90780 (55799-164675)
Latvia	56231 (30157-141961)	331249 (90908-2905009)	120441 (57270-352887)	55794 (23845-200171)	406548 (214834-886254)	558548 (156181-43130437)	512380 (222138-1736167)	1743232 (649830-5357805)	1244615 (602113-3030400)	163334 (93694-323997)	92910 (50513-215683)
Lithuania	39932 (24895-94658)	1240738 (299696-12742987)	92688 (54477-205745)	48315 (24988-144477)	227251 (130229-443013)	426822 (119965-21672204)	443767 (182909-1425664)	1319925 (570452-3323435)	1075750 (584569-2405242)	101557 (69776-161556)	86062 (54523-163764)
Moldova	9591 (5736-22469)	70134 (28607-228772)	15186 (8764-31181)	8854 (4034-30288)	24660 (14445-49776)	88722 (25710-2788456)	59491 (24809-214183)	205335 (88642-518770)	85647 (46374-186550)	27724 (16311-49293)	15517 (9159-29897)
Russia	17827 (11514-41115)	1854054 (474371-19499101)	45400 (26081-101847)	21832 (11836-57718)	60273 (40349-101181)	112451 (34601-2725714)	59338 (25679-178767)	770318 (318767-2275417)	427069 (231997-832551)	55282 (39023-83446)	33588 (21757-61433)

Location	Childhood wasting	Household air pollution	Ambient particulate matter pollution	Antibiotic treatment	Pneumococcal conjugate vaccine	Childhood stunted	Hib vaccine	Childhood under-weight	Hand-washing	Breast-feeding	Second-hand smoking
Ukraine	25973 (12552-64875)	544704 (208437-2461542)	81278 (38156-198400)	46507 (17070-202661)	39179 (20249-84339)	139707 (43358-3726973)	66184 (28798-205856)	673303 (271530-1994707)	643421 (268825-1820796)	176736 (87403-429613)	71778 (34680-174940)
High-income	106332 (67410-258762)	4765256 (3106269-7770337)	270335 (143691-710037)	100284 (62750-227543)	767088 (608603-1003194)	1036494 (292919-99048370)	1016647 (592279-2497767)	2366971 (1032286-5167610)	3032497 (2169816-4878324)	255290 (200044-345332)	210718 (147245-349666)
Brunei	21855 (14326-49034)	20729357 (12574978-37966366)	183904 (65837-1668657)	28994 (15945-76533)	25516 (19518-33772)	157067 (48541-10331648)	739125 (311991-2583322)	229581 (105392-444625)	761356 (520478-1329294)	56317 (40587-81867)	45122 (30099-72850)
Japan	103287 (67813-260744)	222074366 (147419059-354856059)	296521 (161310-770470)	120584 (75181-283351)	7369276 (5831270-9644391)	1028651 (292543-88286028)	8355012 (4778674-21127001)	2516582 (1073504-5796522)	3220038 (2311411-5274589)	374750 (291070-510856)	195659 (137708-306705)
South Korea	149318 (81054-386284)	856259205 (477242577-1696475718)	221826 (136865-399821)	167780 (81519-520043)	3904353 (2246823-6808536)	1681896 (461231-1202592470)	7573374 (3307448-24357853)	4538515 (1895901-11945320)	4466380 (2703352-8099084)	460945 (293439-774417)	268691 (164710-488428)
Singapore	39251 (25416-81184)	97652477 (58298231-188458977)	85763 (52871-175412)	58533 (31077-168440)	395188 (265015-612438)	663842 (179368-Inf)	862501 (425465-2263103)	804413 (349036-1803076)	1571206 (1056079-2720289)	129315 (91651-204475)	72155 (49107-117148)
Australia	205318 (111614-587553)	31778818 (18330722-64468170)	1245598 (415392-11729460)	180402 (100718-469086)	2951181 (1508627-6290534)	2093429 (576121-Inf)	2644349 (1216291-8577055)	6231757 (2473734-17703544)	4866871 (3311796-8011345)	461927 (323243-695592)	333111 (219538-533654)
New Zealand	151488 (83587-458548)	13594770 (7642227-27785828)	937491 (300010-12233901)	106934 (58999-292188)	981343 (571218-1849038)	1489727 (376866-Inf)	853344 (406439-2601188)	4394708 (1838690-12621107)	2794980 (1857608-4712187)	327238 (225266-540369)	224715 (144528-383114)
Andorra	126392 (57319-520135)	8793098 (4161062-20044711)	348683 (145654-1408543)	116342 (44143-449176)	1008445 (467531-2362396)	2643201 (620514-Inf)	3289548 (1291935-14320559)	3668677 (1321271-11945434)	5218834 (2621389-11498148)	765380 (444966-1480989)	259563 (123062-635345)
Austria	386554 (202549-1207524)	16604762 (9682443-30753044)	682064 (363009-1705742)	311668 (152780-925576)	275025 (191661-396781)	7933759 (1999639-Inf)	2583529 (1075961-10382331)	11005749 (4233957-31508519)	13479451 (8513972-24446622)	596778 (393263-976916)	571782 (344698-1082925)
Belgium	206590 (111399-636964)	25247591 (15197652-47456276)	371107 (213268-862023)	181155 (97886-496680)	2187503 (1343900-3988173)	4336895 (1110812-Inf)	11249940 (5177765-38632898)	5959876 (2423929-16371933)	8024006 (5325621-13655823)	397183 (278260-606896)	295779 (193971-499676)
Cyprus	272221 (145030-888001)	18103248 (10985069-33884721)	428756 (246981-958718)	227481 (111287-686255)	1725485 (1009030-3514131)	5426841 (1311789-Inf)	6045360 (2885121-17722215)	7516447 (3012266-22063063)	9800901 (6116413-18110291)	571157 (354624-980503)	315372 (197933-567757)
Denmark	231167 (126602-714927)	33569220 (19856557-65492831)	588253 (273321-2065015)	186545 (97562-534370)	1985791 (1078033-4247312)	5020158 (1231696-Inf)	1858849 (853809-5765816)	6903155 (2794703-18189091)	8499514 (5471461-14488558)	586694 (403329-920376)	390504 (249104-688096)
England	151313 (89971-426537)	8240477 (5513170-12756730)	251462 (133583-693580)	NA (NA-NA)	1050773 (869228-1305599)	1067349 (288532-14945339799)	1323257 (788569-3229178)	2254799 (1043770-4699201)	4092479 (2940423-6508697)	208002 (167336-270383)	236003 (155170-434709)
Finland	1039833 (559856-3469451)	83052093 (45818830-164517424)	4908519 (1668337-46505268)	734569 (368822-2136597)	7943016 (4357273-16053274)	21579688 (5228072-Inf)	41986927 (17012536-159387170)	29307135 (12013295-80269564)	32840775 (20034240-60033464)	2220153 (1390032-3803384)	1988230 (1134983-4010960)

Location	Childhood wasting	Household air pollution	Ambient particulate matter pollution	Antibiotic treatment	Pneumococcal conjugate vaccine	Childhood stunted	Hib vaccine	Childhood under-weight	Hand-washing	Breast-feeding	Second-hand smoking
France	336607 (181130-981696)	32413515 (19117528-59467868)	774801 (388445-2200869)	284739 (149231-798960)	2267435 (1320863-4114983)	6600112 (1634117-Inf)	10619853 (4753870-35831946)	9089441 (3885280-25559360)	12304819 (8017259-21700691)	586521 (395922-915705)	537035 (334926-1022785)
Germany	341627 (188327-1105049)	14626730 (8855873-28459625)	643283 (341669-1647737)	267919 (140153-753897)	421836 (300184-669024)	7453287 (1801778-Inf)	7122721 (3299790-21294370)	8099672 (3314689-22144114)	13235477 (8642335-23556173)	847970 (585178-1320280)	487026 (312558-836343)
Greece	126532 (63015-410191)	10180832 (5495717-19472345)	329211 (151653-1089112)	115025 (51351-342998)	653571 (361276-1272190)	2357396 (590203-Inf)	2868934 (1362584-8465636)	3313647 (1343296-9529216)	5047207 (2970920-9097066)	257338 (147556-431831)	157648 (96190-300291)
Iceland	235260 (130115-748546)	23665805 (13935970-47326310)	972891 (376860-5403205)	193534 (99911-570405)	1564315 (884196-3086742)	4880081 (1168707-Inf)	1787629 (811424-6004320)	6861518 (2777666-18168118)	8782246 (5642755-15006637)	684441 (467563-1081873)	608802 (360431-1377563)
Ireland	321326 (174251-1019782)	19898958 (11666137-37335630)	893109 (388416-3421972)	246838 (122501-731307)	2106166 (1301895-3703720)	6537373 (1637238-Inf)	3227915 (1536137-10381235)	8958302 (3806656-24730723)	10785461 (6740548-19329029)	591329 (380707-994869)	510386 (312751-924359)
Israel	212951 (115748-662047)	12466490 (7540358-21808783)	316153 (185150-651220)	174954 (93784-468413)	2130000 (1067720-4687136)	3568878 (889708-Inf)	2197551 (912774-6979970)	5145967 (2161996-13575431)	6951005 (4545292-11851296)	457864 (317401-684524)	298027 (202654-488740)
Italy	300354 (157962-964077)	17889526 (10328263-35234159)	541439 (282593-1395212)	254765 (124164-809120)	1750831 (962910-3288527)	5901856 (1485237-Inf)	2957650 (1375903-9202753)	8281291 (3643903-23302491)	10323510 (6307028-18962780)	663763 (432689-1168692)	513496 (304775-1014866)
Luxembourg	402849 (215966-1276439)	35174933 (20409467-64791381)	652333 (370141-1521581)	319891 (159896-925600)	4063431 (2429351-7359473)	8715522 (2209012-Inf)	81811387 (34542659-294026241)	12156630 (5154446-34435848)	14714371 (9619409-25622445)	847013 (549812-1373647)	464790 (303295-753491)
Malta	127937 (70066-383783)	5304017 (3086354-9713002)	250352 (124701-843007)	94119 (50269-268874)	83047 (61320-114944)	2362145 (586571-Inf)	1973359 (745409-8505390)	3305212 (1353415-9639457)	3995014 (2652881-7155419)	258436 (175258-407856)	135811 (85908-221255)
Netherlands	300062 (162335-884984)	41027078 (24011649-78887862)	523370 (284789-1308674)	245054 (129383-684175)	3653846 (1918710-7533323)	6394949 (1579638-Inf)	3632789 (1695820-11139288)	8692976 (3491918-25312542)	11055871 (7151583-19235969)	595809 (411879-938077)	446041 (287610-781017)
Northern Ireland	121810 (62277-423070)	10300621 (5776965-20543797)	329551 (138895-1380292)	NA (NA-NA)	3796858 (1561151-17542198)	2662874 (650679-Inf)	6903266 (2468487-271568662)	3464730 (1430305-10595740)	4118601 (2462951-8280008)	205560 (130900-363682)	244584 (137198-568936)
Norway	370275 (205876-1107794)	25242897 (15079968-49326462)	1279791 (530581-6232159)	286932 (149287-833736)	2887610 (1710725-5255532)	8067885 (1981556-Inf)	3531468 (1692263-11776615)	11259486 (4659256-31670470)	13030388 (8430921-22471531)	1013822 (668658-1730445)	590005 (373002-1056486)
Portugal	155868 (88527-472336)	3664560 (2234089-6566535)	431159 (192826-1692577)	124172 (66511-330549)	701119 (417921-1293554)	2605732 (670512-Inf)	4606107 (2024447-16562142)	3762519 (1601672-9274707)	4893016 (3308590-8467432)	305047 (212920-457149)	230156 (151321-394039)
Scotland	152581 (84411-414699)	7453065 (4420592-14075000)	517751 (238947-1949429)	NA (NA-NA)	5757472 (2153298-30486423)	3499135 (887029-Inf)	7244303 (2317882-106446285)	5263311 (2036204-15263562)	6514546 (4042074-12013333)	324049 (213976-505747)	401336 (230184-867287)
Spain	250784 (143984-712124)	11001360 (6414767-20493361)	758453 (351199-2744353)	230873 (117483-673058)	1247567 (725141-2386948)	6059352 (1526627-Inf)	5518241 (2356171-17944924)	7947294 (3095308-21337059)	9988780 (6514763-18218344)	577898 (383314-935596)	326122 (210919-541058)

Location	Childhood wasting	Household air pollution	Ambient particulate matter pollution	Antibiotic treatment	Pneumococcal conjugate vaccine	Childhood stunted	Hib vaccine	Childhood under-weight	Hand-washing	Breast-feeding	Second-hand smoking
Sweden	328381 (171210-1028595)	10349538 (5890643-20232960)	2823471 (810296-46830715)	276693 (138853-857575)	6544930 (3431427-13352161)	6528832 (1662764-Inf)	8675530 (3177510-29499284)	8931679 (3722287-22813632)	11876505 (7442894-21746502)	899510 (594747-1559312)	825641 (496871-1649475)
Switzerland	300386 (153354-938574)	11455424 (6515249-23368377)	725500 (332959-2548331)	234579 (115499-702894)	577011 (367140-986277)	6400301 (1587299-Inf)	3176535 (1435579-10239733)	8795240 (3508110-24070132)	10421035 (6419048-18660635)	701854 (470827-1133734)	439683 (273517-807031)
Wales	162578 (86263-517560)	10608736 (6277948-19072939)	398080 (199212-1343763)	NA (NA-NA)	3477340 (1520314-13831562)	3544724 (901755-Inf)	4167957 (1404286-29364379)	4470841 (1789078-12392294)	5504382 (3599279-9586139)	259019 (178104-397907)	296284 (187927-554281)
Argentina	15494 (10043-34614)	487688 (305401-861697)	52330 (29051-129311)	22449 (12598-57011)	171953 (106624-303665)	145176 (43441-9703005)	222164 (107667-674556)	359733 (163899-822592)	434307 (302211-714155)	66182 (45231-109340)	41176 (28010-69072)
Chile	104493 (49521-431196)	623886 (358288-1156236)	87562 (55172-165802)	54349 (29250-151640)	252259 (148491-448993)	860086 (225490-891586471)	269982 (134359-748593)	3572008 (1281377-17570569)	1205979 (784750-2100401)	175140 (116068-285957)	129248 (75266-302062)
Uruguay	23498 (12285-67895)	646836 (346163-1354566)	74037 (35924-246827)	26582 (12264-92703)	452673 (211116-1083834)	125591 (35559-4078521)	382249 (158524-1435579)	313148 (135636-789174)	561857 (327883-1042771)	80730 (49335-145186)	44160 (25900-81514)
Canada	236770 (117124-863927)	69350744 (42330488-134335002)	751276 (312063-4047157)	155504 (83969-419533)	434947 (290120-705858)	2281752 (587185-Inf)	575697 (297182-1541827)	5791133 (2220701-18835377)	6921604 (4591795-11511854)	405358 (278059-625586)	254191 (165036-414316)
Greenland	54441 (25998-215710)	1302379 (785401-2486337)	714345 (156632-33861065)	36770 (18349-110844)	448120 (223168-1067853)	382197 (97013-Inf)	392102 (173339-1323376)	1028089 (407197-3298342)	1494400 (946581-2665821)	90624 (58236-158679)	87622 (48315-200577)
United States	171527 (98555-521980)	26658212 (17661453-42844185)	381850 (174487-1451886)	103706 (65509-235450)	1019493 (810201-1330867)	1520526 (393790-Inf)	827172 (487422-2024816)	3322673 (1456326-6900797)	5125652 (3595974-8234443)	231072 (183565-304180)	308682 (201889-563173)
Latin America and Caribbean	5665 (3959-11536)	14661 (10392-21853)	13305 (8853-22623)	7408 (4582-16899)	25024 (18801-33930)	27162 (9523-651305)	42497 (24867-100603)	57115 (26941-101105)	40342 (29519-64373)	23184 (17093-34820)	26956 (18222-46628)
Antigua and Barbuda	10512 (5816-26688)	365213 (160343-1062804)	27860 (13327-112875)	13694 (5982-52198)	11394 (7414-19801)	88322 (24133-4239030)	2154644 (725003-11973906)	177744 (77730-441514)	85443 (49273-161979)	31513 (18238-63126)	37965 (21850-74760)
The Bahamas	10093 (4358-30804)	194186 (75289-745824)	34378 (13001-155881)	13923 (4474-66237)	194823 (64487-831255)	88940 (21830-5039204)	347921 (123882-1466865)	176229 (62996-544382)	101493 (43899-265039)	29228 (12483-76264)	53785 (23314-148432)
Barbados	9057 (4334-24141)	9709993 (3739210-36305802)	26990 (11008-132616)	26691 (7618-167337)	118258 (51792-346361)	103239 (28562-2315460)	111160 (41242-446822)	149208 (62362-458524)	97508 (43818-270850)	32756 (15393-84049)	64153 (28279-171302)
Belize	7761 (3641-24162)	31521 (14436-94885)	17788 (7681-52218)	11726 (3819-57095)	8977 (4466-21026)	50882 (14250-1607179)	36199 (14784-137359)	99631 (41272-299897)	52197 (25375-135856)	23750 (11321-60628)	51915 (22298-160507)
Bermuda	51910 (26632-160721)	753237 (320398-2322328)	208428 (84202-1204538)	71207 (29282-276202)	59618 (35684-105539)	454426 (129677-23915666)	53851 (29283-125999)	925729 (374598-2563379)	501353 (279307-1032776)	208786 (122495-410297)	216844 (115218-487184)

Location	Childhood wasting	Household air pollution	Ambient particulate matter pollution	Antibiotic treatment	Pneumococcal conjugate vaccine	Childhood stunted	Hib vaccine	Childhood under-weight	Hand-washing	Breast-feeding	Second-hand smoking
Cuba	28104 (18862-62517)	686820 (349409-1661991)	66511 (38804-138480)	36181 (19261-102173)	28910 (22714-38670)	203076 (59577-4070901)	652950 (286719-2206313)	422672 (194129-869921)	262680 (177523-440290)	102861 (70572-161891)	54139 (36563-88090)
Dominica	9306 (4923-23073)	60353 (27265-174857)	18367 (9223-57002)	9669 (3989-34960)	7965 (4820-13782)	76536 (21268-4125314)	326440 (133706-1195640)	142954 (55255-378337)	51300 (29847-108494)	19254 (10846-38296)	39001 (21229-94618)
Dominican Republic	5475 (3119-13127)	29017 (15916-63537)	10437 (6016-20020)	6907 (3091-22539)	8591 (4962-18080)	39795 (11390-2717411)	15292 (8177-40437)	68803 (32549-152217)	18826 (11479-34246)	16238 (9754-30580)	39657 (21270-87041)
Grenada	7750 (3723-20949)	142989 (58161-477020)	17305 (7515-69592)	9760 (3579-39816)	8097 (4380-16067)	57125 (15877-3762817)	313774 (119795-1332114)	112096 (46026-309350)	63033 (30169-148180)	26026 (13577-57088)	37112 (16799-90044)
Guyana	5753 (3810-10716)	45794 (24083-94054)	17275 (9766-37037)	9617 (4861-28868)	144039 (77498-308944)	42354 (13169-1567750)	127884 (55743-415941)	49946 (23737-102691)	30891 (20292-54292)	28757 (18193-53944)	32618 (18532-66423)
Haiti	1151 (635-2578)	1326 (745-2623)	3247 (1744-7612)	2129 (884-7825)	1834 (1102-3348)	6515 (2224-143740)	5855 (2727-18611)	8384 (3685-19380)	4873 (2868-9653)	6784 (3631-15044)	17190 (8292-49908)
Jamaica	11232 (5437-33564)	61337 (28403-163324)	29526 (13806-82227)	14128 (5650-57702)	11937 (6847-23218)	88724 (26027-4559230)	348021 (130428-1292536)	172373 (74171-498854)	55014 (29662-120460)	38972 (20191-87638)	35469 (19045-79762)
Puerto Rico	43033 (26717-104495)	18495270 (8579821-57441789)	81200 (38383-535685)	47863 (24081-147414)	378733 (157858-953497)	470605 (135424-33634758)	504365 (221046-1805352)	964915 (412024-2484276)	472191 (296029-870996)	95722 (64553-159975)	291132 (158759-741762)
Saint Lucia	13962 (6586-36016)	175589 (72810-523255)	30451 (13841-114083)	16188 (5629-80888)	13589 (7132-29927)	242529 (64516-257266423)	804807 (262810-3457302)	257882 (111557-822509)	129269 (65120-328971)	41771 (21540-96267)	53717 (25975-139119)
Saint Vincent and the Grenadines	10075 (5243-25129)	92108 (39047-272566)	24292 (11740-81912)	13138 (5105-50363)	10848 (6302-19899)	74383 (21632-2800552)	2531012 (836619-14546467)	137290 (64318-345914)	74067 (40610-157190)	28333 (16256-55787)	53524 (26813-124787)
Suriname	3803 (2072-9071)	21216 (10364-52758)	10081 (5194-25058)	5629 (2281-21221)	8875 (4167-36388)	32966 (8626-2010162)	69531 (29840-243114)	45920 (18875-117541)	30462 (16196-67616)	12080 (6487-26095)	12173 (6457-25872)
Trinidad and Tobago	14039 (5650-47610)	1366623 (470210-5928644)	30400 (11507-113897)	17229 (5013-106389)	29764 (12390-89873)	450803 (113171-Inf)	27156 (10385-102363)	263955 (100945-977475)	108148 (43712-338941)	40983 (17123-130664)	53692 (21152-225459)
Virgin Islands, U.S.	56362 (30818-146350)	2159114 (877923-6594065)	97311 (45121-604269)	53201 (25056-182021)	43960 (29600-66649)	605166 (168480-24893992)	40359 (25187-86958)	1224925 (518128-3412887)	431212 (260943-793292)	95489 (56587-174929)	224764 (131671-456805)
Bolivia	1875 (1138-4368)	5036 (2915-10756)	3719 (2273-6833)	2610 (1291-8060)	10726 (5782-23936)	7838 (2453-487258)	13305 (6558-40091)	20759 (9579-46987)	14011 (8807-24752)	9336 (5719-18070)	14556 (7777-38347)
Ecuador	4740 (2925-11096)	54644 (25597-143339)	15672 (8063-42110)	6320 (3300-17000)	78111 (40769-171130)	20054 (6609-293868)	70268 (32683-223522)	57358 (26914-115426)	40975 (26257-70950)	27027 (16925-50458)	36275 (21169-69861)

Location	Childhood wasting	Household air pollution	Ambient particulate matter pollution	Antibiotic treatment	Pneumococcal conjugate vaccine	Childhood stunted	Hib vaccine	Childhood under-weight	Hand-washing	Breast-feeding	Second-hand smoking
Peru	5901 (3407-16001)	10949 (6752-19216)	8719 (5512-14744)	5746 (3013-16193)	36710 (21134-67920)	18418 (6200-372354)	31818 (15948-96438)	49224 (22746-108176)	36839 (23182-64537)	36073 (22587-68386)	32500 (18392-73127)
Colombia	13847 (7864-36997)	45220 (24926-95248)	26421 (15010-57151)	12471 (6533-34253)	221066 (114643-466203)	65499 (19144-4557211)	205140 (95960-696902)	142131 (62022-295477)	98412 (62539-171209)	43409 (27473-75001)	45791 (27987-90983)
Costa Rica	24710 (13214-65239)	241457 (115649-562052)	58044 (30689-138030)	34487 (14346-128808)	502203 (237485-1175590)	231754 (62182-175025079)	483326 (203195-1719111)	699046 (300449-2040480)	239205 (132049-493194)	93157 (52159-186584)	109876 (60967-229460)
El Salvador	8521 (4888-20343)	32713 (16281-81617)	14069 (8555-24209)	12128 (5573-39940)	83580 (46676-169029)	44509 (12785-3641083)	71885 (34904-223639)	87458 (40650-205877)	72192 (43656-137995)	32071 (19401-62135)	48415 (26831-108659)
Guatemala	2424 (1454-6577)	2610 (1727-4408)	3214 (2285-5102)	2335 (1348-5939)	18578 (11522-34730)	4208 (1652-41408)	37196 (16706-115717)	9389 (4834-16951)	12094 (8379-20121)	10331 (6832-17579)	14111 (8286-31386)
Honduras	11313 (5991-29389)	9945 (6128-17564)	12495 (7994-21689)	9140 (4485-27096)	114372 (63489-244937)	38225 (12490-1162880)	94672 (44424-278904)	76091 (34844-174421)	66266 (41001-124770)	28178 (17635-52942)	25083 (15538-48138)
Mexico	7690 (5371-15727)	37115 (25177-57772)	19411 (12336-37104)	10978 (6504-26044)	43589 (34331-56485)	47917 (15705-1460455)	41270 (24961-98508)	118006 (53262-226764)	99610 (70541-162569)	22332 (16649-32960)	33581 (21823-65837)
Nicaragua	6046 (3769-15406)	7494 (4730-14095)	10543 (6768-19579)	6711 (3430-17992)	371330 (159699-1129143)	25454 (8253-464720)	375015 (149158-1513698)	63074 (27916-133378)	39578 (26173-69241)	17455 (11535-30099)	23552 (14299-51480)
Panama	6719 (3547-18976)	29129 (14870-74819)	17287 (8738-44569)	8332 (3647-26610)	25826 (15526-48036)	26002 (8424-1549506)	23747 (11758-64193)	81709 (35015-191616)	69649 (39983-131744)	22417 (12593-44304)	36156 (21145-65336)
Venezuela	6610 (4669-11451)	433156 (187073-1321187)	13542 (9050-22693)	9884 (5426-26970)	13033 (9120-20295)	58460 (18094-1601349)	24277 (13551-59861)	135848 (63558-282258)	83039 (56908-142432)	22792 (15795-37161)	32037 (19375-66969)
Brazil	7490 (5344-15500)	89172 (56710-141614)	34163 (17863-88982)	10772 (6776-24597)	197960 (140492-283885)	76344 (22331-5164168)	207390 (112194-534261)	145475 (65685-294339)	72112 (52222-114819)	32639 (24626-46638)	22579 (16155-33817)
Paraguay	9555 (5421-24387)	18447 (10737-35281)	17782 (10359-33615)	10936 (5270-33485)	38684 (23302-71481)	65623 (19436-5101419)	33143 (16633-101184)	164310 (70727-432710)	77215 (48674-138757)	32655 (19466-61818)	32165 (18726-63882)
North Africa and Middle East	2716 (1992-4508)	6384 (4364-10626)	4317 (3188-6076)	5125 (2794-13974)	8440 (6451-11412)	10748 (4246-111558)	8914 (5617-19216)	14337 (7733-25841)	21631 (14189-37630)	20209 (13421-32997)	17031 (10484-35228)
Afghanistan	570 (359-1124)	713 (463-1266)	961 (616-1687)	1150 (523-4256)	2179 (1395-3976)	1638 (682-14392)	1894 (1040-4879)	2375 (1219-4998)	3248 (1996-6292)	4944 (2722-9558)	3607 (1967-8821)
Algeria	11465 (6175-26632)	2118184 (916577-6202403)	25266 (13714-53702)	20589 (8032-85766)	18877 (10542-37629)	96943 (28357-4944108)	1378843 (549340-5492677)	198331 (78161-541124)	216388 (116561-482692)	63293 (33230-147156)	45495 (24560-106375)

Location	Childhood wasting	Household air pollution	Ambient particulate matter pollution	Antibiotic treatment	Pneumococcal conjugate vaccine	Childhood stunted	Hib vaccine	Childhood under-weight	Hand-washing	Breast-feeding	Second-hand smoking
Bahrain	22811 (13945-48225)	1168680 (603849-2840153)	26809 (18392-41109)	35909 (17608-109513)	4357503 (2001227-11753454)	382932 (105155-Inf)	3669876 (1546127-12355591)	294861 (134914-666515)	369665 (236947-656679)	103239 (67278-172123)	93455 (57141-170997)
Egypt	2910 (1798-5336)	657400 (314784-1741241)	3142 (1965-5380)	4488 (2070-14524)	4196 (2671-6750)	15458 (5007-279313)	4135 (2428-9358)	33280 (14866-70515)	57035 (33045-112194)	16778 (9554-35028)	12041 (6472-27607)
Iran	11325 (4881-37319)	1672834 (640732-5754324)	18772 (8198-50065)	19451 (6066-113103)	17009 (7532-43456)	120765 (34946-6558604)	3771866 (1286359-18009936)	167772 (61660-646516)	239408 (105196-676203)	70126 (29204-216161)	46270 (20227-144236)
Iraq	3596 (2008-8887)	112016 (51174-349804)	5234 (3132-12379)	5874 (2492-27563)	12924 (6758-34780)	16606 (5652-302379)	13798 (6857-44531)	23447 (10746-64663)	94830 (53941-235929)	18557 (9999-47469)	15227 (8139-40169)
Jordan	8243 (5122-17196)	16112753 (8139025-39564566)	13065 (8790-20614)	13157 (6410-40087)	10234 (7343-14878)	70045 (20403-3942082)	1067421 (487211-3543595)	154652 (69573-348632)	126569 (81693-227752)	29607 (19285-53313)	24911 (14602-51764)
Kuwait	26125 (14206-69005)	5167555 (2460107-12916937)	19949 (13038-33062)	29902 (14018-100044)	3339634 (1451362-9534173)	342873 (92242-93229596)	3382330 (1509103-11267629)	618302 (262256-1624284)	388314 (238826-738001)	68727 (42076-119653)	59660 (35130-120862)
Lebanon	33753 (18055-82449)	688930727 (294975252-2053764375)	68906 (38500-150518)	57633 (22871-244442)	733500 (194670-3631261)	238958 (70842-7715357)	1854663 (697384-7459515)	538377 (225598-1557584)	399250 (217594-982124)	134796 (73336-306076)	95368 (50792-213858)
Libya	20263 (10580-50549)	6673084 (2821897-21218377)	31688 (17742-71864)	36570 (14306-177344)	1055631 (447820-3476910)	118895 (38706-5005648)	892294 (337505-3539998)	348680 (156396-1049272)	475396 (251185-1205250)	172086 (89509-436004)	132433 (61323-436305)
Morocco	7897 (4665-18070)	138349 (69177-347307)	14614 (8832-28500)	9601 (4353-31683)	123498 (60244-308306)	44578 (14038-941433)	170601 (75584-495090)	115065 (53351-297108)	69603 (41503-132947)	28449 (16164-57154)	30729 (17390-71562)
Palestine	16716 (7936-48466)	1008165 (438344-3206449)	43395 (21320-117561)	25928 (9951-107162)	21450 (12052-45313)	120322 (34838-7853916)	19366 (9758-49345)	301924 (130118-869192)	125604 (67466-299471)	102048 (51378-300348)	78308 (35824-257897)
Oman	20851 (13267-37847)	1271520 (613147-3143315)	32114 (21197-52779)	41287 (19354-131238)	6565540 (2158799-26618544)	191434 (57370-10487652)	12349628 (5321566-48233231)	206002 (96591-435037)	354532 (215736-664327)	143866 (85683-284502)	118066 (69336-238836)
Qatar	36519 (19550-88530)	165007053 (75704857-437590436)	36239 (21571-65747)	59522 (25129-209763)	1393673 (697391-3338359)	342983 (95961-23088614)	1334187 (570260-4474630)	565146 (229359-1557685)	692207 (392631-1410599)	143017 (77850-283214)	195603 (101348-480296)
Saudi Arabia	102254 (62474-223454)	12603548 (7107121-22516870)	116619 (75146-188150)	194242 (89491-632657)	4869196 (2915405-8705931)	1060000 (318012-49544809)	5343794 (2774480-14336502)	1739553 (798359-4118961)	1551700 (941022-2970482)	927571 (594261-1647443)	842964 (405917-2657590)
Sudan	978 (517-2216)	1928 (1019-4374)	1895 (1044-4063)	2552 (930-11477)	7361 (3782-17079)	4816 (1705-66463)	6318 (2789-20583)	4549 (2124-11040)	6016 (3063-13406)	12780 (5975-36959)	234826 (29462-Inf)
Syria	7737 (4123-17283)	6071200 (2584261-16674036)	18987 (10156-36853)	16686 (6541-61918)	16064 (8872-30474)	37888 (13075-816069)	42562 (19055-122763)	85164 (35906-215170)	185682 (98447-419069)	146502 (79852-314914)	56967 (27838-167346)

Location	Childhood wasting	Household air pollution	Ambient particulate matter pollution	Antibiotic treatment	Pneumococcal conjugate vaccine	Childhood stunted	Hib vaccine	Childhood under-weight	Hand-washing	Breast-feeding	Second-hand smoking
Tunisia	24515 (13531-56961)	4214982 (1958164-10651443)	43488 (25719-82202)	38321 (16480-132609)	33040 (21007-55717)	190382 (58270-4998534)	1333451 (520680-5543109)	442154 (199159-1133521)	307773 (173638-602730)	115692 (65517-220282)	64457 (36603-131064)
Turkey	22446 (11964-60749)	833767 (383056-2265057)	30068 (17182-58008)	24511 (10397-87053)	172117 (94719-365750)	133967 (36812-8418419)	160867 (73393-506550)	460739 (191177-1232975)	191552 (107401-411139)	98235 (53649-214304)	41710 (23122-85517)
United Arab Emirates	54574 (25431-150120)	9816861 (3748899-31369405)	79992 (40554-191657)	116946 (40333-565608)	2282015 (892663-7644014)	502206 (135799-37917195)	2071169 (742534-8663737)	866091 (322951-2933749)	1240398 (581696-3120470)	374676 (171786-942716)	364664 (173466-985391)
Yemen	2138 (1143-5627)	7056 (3494-20669)	4375 (2248-11467)	5050 (1843-29001)	11713 (5673-32176)	8987 (3061-125527)	10183 (4339-35697)	7737 (3700-23282)	17069 (8302-48156)	14484 (6939-44760)	15886 (7017-58878)
South Asia	1387 (1160-1871)	2188 (1693-2991)	1935 (1569-2477)	2788 (1698-6775)	2671 (2273-3212)	5422 (2243-59956)	8672 (5333-20004)	5162 (3049-7488)	8466 (6289-13401)	9022 (6484-13985)	7353 (4704-14272)
Bangladesh	1623 (1135-2509)	1993 (1353-3104)	1987 (1406-3063)	2959 (1494-9328)	8969 (6184-13764)	8349 (2985-232829)	26241 (12883-75583)	7651 (3899-14035)	7195 (4960-11968)	12250 (7300-23664)	12007 (5632-48634)
Bhutan	2046 (1377-3611)	5432 (3207-10337)	3096 (2094-4994)	3734 (1798-11654)	2975 (2062-4511)	9961 (3443-327448)	52169 (22692-181061)	14521 (6849-33142)	28610 (17384-52551)	11797 (7006-24523)	10456 (6356-19984)
India	1356 (1129-1862)	2196 (1691-3016)	1882 (1526-2408)	2801 (1696-6807)	2174 (1852-2606)	4994 (2133-48100)	8931 (5384-21122)	4918 (2953-7158)	8268 (6048-13020)	8628 (6202-13128)	7187 (4612-13848)
Nepal	1674 (1125-2646)	2063 (1352-3422)	2188 (1471-3452)	2960 (1410-9514)	6323 (4258-9851)	9178 (3148-156376)	15615 (7747-46031)	8390 (4160-16294)	11031 (7224-20138)	15114 (8782-29605)	12578 (6041-43764)
Pakistan	1372 (956-2045)	2308 (1552-3687)	2150 (1502-3211)	2647 (1370-7664)	6280 (4038-10250)	6211 (2326-124267)	5300 (2948-12636)	5013 (2719-9091)	10369 (6919-17943)	8911 (5662-15861)	6120 (3727-11989)
Sub-Saharan Africa	837 (656-1233)	1064 (839-1452)	1544 (1213-2079)	1544 (931-3715)	3575 (2844-4669)	3566 (1362-45002)	3530 (2248-8012)	4603 (2487-7236)	3465 (2612-5415)	6177 (4308-9983)	10146 (6212-21447)
Angola	1051 (627-2081)	2045 (1084-5422)	2500 (1524-4413)	1908 (857-6448)	6692 (3754-12949)	4707 (1602-46793)	6441 (3135-19507)	6523 (3178-14597)	4386 (2579-8791)	5746 (3181-11524)	8727 (4918-19010)
Central African Republic	374 (228-700)	414 (269-707)	623 (397-1029)	631 (299-1955)	1061 (687-1728)	1488 (545-15468)	937 (504-2197)	1920 (1010-3900)	1522 (954-2676)	2808 (1527-6395)	4266 (2312-10096)
Congo	1381 (802-2652)	2249 (1238-4824)	2552 (1460-4517)	3243 (1312-12056)	8028 (4308-15356)	8645 (2842-306018)	6682 (3250-18973)	11394 (5371-25106)	5936 (3469-11023)	9524 (5289-19186)	13612 (7352-34469)
Democratic Republic of the Congo	773 (445-1573)	907 (538-1783)	1475 (869-2807)	1437 (605-5507)	4015 (2184-8387)	3058 (1060-37409)	3589 (1780-10344)	4517 (2129-10027)	3100 (1766-6320)	6552 (3378-15203)	9620 (4528-29120)

Location	Childhood wasting	Household air pollution	Ambient particulate matter pollution	Antibiotic treatment	Pneumococcal conjugate vaccine	Childhood stunted	Hib vaccine	Childhood under-weight	Hand-washing	Breast-feeding	Second-hand smoking
Equatorial Guinea	2855 (1451-6813)	3455 (1762-13231)	3327 (1858-6822)	3615 (1493-13411)	3337 (1979-6068)	14751 (5009-605182)	3970 (2088-10008)	40502 (17166-101866)	8623 (4905-18188)	9869 (5355-21326)	17425 (9358-39266)
Gabon	2463 (1462-5537)	11295 (4511-45557)	3680 (2218-6147)	3523 (1579-12095)	3119 (2033-5291)	14642 (4846-548178)	8564 (4532-22453)	26196 (12323-60657)	9821 (5671-20806)	8546 (4973-16356)	16937 (8851-46451)
Burundi	780 (478-1512)	790 (503-1349)	1448 (893-2573)	1422 (650-5182)	12716 (6837-29872)	2372 (924-18600)	10328 (4498-31377)	3205 (1629-6758)	2836 (1746-5293)	8648 (4489-18331)	9259 (5042-22726)
Comoros	1030 (619-1928)	1358 (845-2433)	3440 (1854-8227)	1779 (832-5575)	1682 (1111-2725)	5155 (1967-170396)	4138 (2213-12017)	6589 (3147-15124)	4402 (2572-8116)	5400 (3107-10773)	11507 (5546-36717)
Djibouti	745 (463-1287)	3403 (1251-29578)	1556 (932-2853)	1790 (756-6656)	4448 (2603-8540)	4247 (1592-47168)	3831 (1901-10529)	4844 (2408-11177)	4210 (2343-8525)	4656 (2729-9778)	4876 (2537-12341)
Eritrea	875 (562-1581)	1749 (905-9718)	1852 (1190-3116)	1702 (831-5569)	14982 (8117-30942)	4537 (1624-56166)	50566 (20419-196667)	4691 (2217-10202)	3953 (2408-7319)	12250 (6764-28437)	8499 (4730-17874)
Ethiopia	1211 (868-1923)	1473 (1054-2258)	2523 (1764-3896)	2401 (1265-6836)	4015 (2777-6255)	4218 (1745-30394)	3626 (2154-7933)	5665 (3044-10545)	4953 (3395-8304)	10741 (6849-19450)	44356 (20728-166933)
Kenya	1285 (947-2201)	1324 (1021-1793)	3917 (2354-8139)	1803 (1082-4480)	11394 (8351-16805)	6239 (2147-149000)	9766 (5590-24567)	9842 (4959-16893)	4035 (2946-6294)	5811 (3982-10031)	8817 (5677-16251)
Madagascar	562 (373-897)	747 (513-1170)	2135 (1342-4129)	1159 (583-3480)	5162 (3035-9691)	2331 (953-19964)	4430 (2223-12649)	2753 (1365-5736)	2690 (1684-4677)	6103 (3502-11980)	8554 (4388-26731)
Malawi	930 (575-2111)	863 (584-1309)	2048 (1310-3491)	1377 (680-4239)	14395 (8708-25820)	3081 (1150-37636)	12130 (5803-32577)	5891 (2967-11715)	2926 (1869-5052)	8192 (4873-16617)	21846 (8796-167341)
Mozambique	879 (572-1679)	947 (657-1515)	2766 (1679-5490)	1549 (778-4532)	5559 (3470-9586)	3815 (1354-38609)	4773 (2611-12902)	6006 (3013-11725)	3458 (2248-5918)	5180 (3120-9286)	7240 (4094-16252)
Rwanda	1106 (679-2321)	1023 (711-1627)	1664 (1125-2661)	1858 (909-5814)	34516 (18594-74800)	3757 (1397-55355)	28626 (13362-82994)	9361 (4454-19622)	3385 (2202-5953)	16456 (9436-32064)	10126 (5901-21604)
Somalia	352 (222-623)	451 (294-762)	1240 (715-2501)	743 (354-2473)	700 (459-1151)	1710 (607-16040)	777 (455-1760)	1911 (931-4236)	1683 (1060-3113)	2124 (1278-4010)	3850 (2085-9710)
South Sudan	397 (267-646)	503 (340-833)	924 (591-1571)	839 (405-2599)	819 (547-1309)	1952 (749-26420)	956 (548-2250)	1884 (952-3954)	1862 (1165-3249)	3176 (1890-6502)	8937 (4068-31111)
Tanzania	850 (561-1548)	975 (674-1538)	2543 (1590-4850)	1488 (761-4385)	10759 (6307-21369)	3686 (1370-45503)	9983 (4864-30028)	6187 (2998-12804)	3276 (2209-5709)	6639 (4054-13133)	5667 (3512-11394)

Location	Childhood wasting	Household air pollution	Ambient particulate matter pollution	Antibiotic treatment	Pneumococcal conjugate vaccine	Childhood stunted	Hib vaccine	Childhood under-weight	Hand-washing	Breast-feeding	Second-hand smoking
Uganda	1141 (769-2029)	1303 (931-1951)	1776 (1266-2654)	2269 (1166-7476)	7858 (4740-14044)	5304 (1818-112970)	7959 (4021-23569)	7979 (3810-15447)	4640 (3075-7831)	10936 (6774-20806)	15177 (8136-40588)
Zambia	797 (521-1431)	959 (661-1528)	1939 (1270-3229)	1435 (726-4304)	8178 (5158-14298)	2760 (1081-29439)	7636 (3831-20298)	5784 (2839-11001)	3193 (2105-5393)	6021 (3535-12882)	9399 (4716-31117)
Botswana	8506 (4420-19944)	23060 (11115-57920)	21053 (10643-48636)	12981 (5283-54359)	302831 (109202-1003504)	39751 (12480-2701245)	600086 (224578-2548240)	78993 (33625-211928)	40695 (20924-98832)	51463 (25948-127965)	33159 (16697-82678)
Lesotho	1089 (659-2231)	1445 (913-2498)	2402 (1458-4379)	1473 (730-4540)	5174 (2911-9426)	4062 (1442-67604)	5565 (2780-14470)	8676 (4140-18545)	3181 (2022-5897)	5892 (3573-11323)	3293 (2009-6162)
Namibia	1778 (1041-3497)	3610 (2016-6987)	5143 (2845-10905)	3818 (1636-14727)	11143 (5786-23932)	11445 (3714-323103)	12929 (6137-39979)	14878 (6532-34399)	11467 (6104-25889)	12128 (6568-26729)	22026 (9966-78848)
South Africa	1818 (1170-3508)	8582 (5281-15358)	3805 (2507-6229)	3028 (1506-8958)	15522 (9827-25426)	8979 (3111-108867)	13415 (7187-33456)	17917 (8388-34275)	9985 (6366-17860)	7121 (4780-11792)	8538 (5169-16399)
Swaziland	1302 (733-3264)	2133 (1297-4155)	2683 (1649-5113)	1610 (777-5170)	16176 (7370-38574)	4221 (1460-172778)	21283 (8857-74075)	11908 (5458-27979)	4979 (3097-10074)	5450 (3301-10381)	11560 (6810-26851)
Zimbabwe	1288 (807-2589)	1629 (1080-2707)	3058 (1862-5724)	1940 (1008-5854)	19505 (11653-36459)	5589 (1901-187734)	18114 (9046-53059)	8966 (4469-18158)	4342 (2854-7806)	6487 (3961-12587)	8790 (4983-19638)
Benin	947 (648-1681)	1101 (794-1753)	1374 (952-2103)	1699 (844-4956)	5910 (3725-10450)	4199 (1529-46454)	5215 (2829-13945)	5580 (2607-11330)	3666 (2387-6386)	7821 (4771-14985)	11940 (6846-23989)
Burkina Faso	491 (324-826)	602 (393-1025)	740 (492-1238)	959 (467-3118)	6126 (3382-12476)	2335 (863-31496)	5132 (2443-15381)	2472 (1252-4955)	2165 (1354-4140)	3760 (2067-8561)	5552 (2844-16716)
Cameroon	706 (452-1404)	913 (574-1550)	838 (568-1343)	1146 (575-3451)	5444 (3177-10134)	3012 (1073-25184)	4648 (2341-13392)	4286 (2154-8365)	2746 (1715-4875)	4537 (2589-9401)	7336 (4027-17143)
Cape Verde	5787 (3792-11680)	16060 (7565-63414)	8736 (5885-13580)	9415 (4839-27680)	8964 (6424-13125)	48658 (14625-904162)	291279 (121146-1055240)	79299 (36537-181804)	28647 (17626-54206)	33695 (20240-62170)	81670 (40476-252721)
Chad	335 (232-509)	446 (314-689)	603 (429-911)	696 (364-1959)	689 (499-987)	1541 (602-15894)	916 (539-2200)	1552 (805-2845)	1585 (1037-2611)	2486 (1455-4831)	10008 (4521-44580)
Cote d'Ivoire	768 (485-1398)	1005 (642-1680)	1354 (863-2273)	1395 (664-4384)	3495 (2019-6753)	3850 (1482-53501)	5259 (2696-15072)	5124 (2481-10856)	3235 (1941-5804)	4546 (2586-10684)	4678 (2643-9597)
The Gambia	1253 (872-2061)	1488 (1079-2337)	2011 (1362-3169)	2693 (1344-8065)	13039 (8231-23018)	7687 (2625-165117)	10778 (5376-30430)	7770 (4023-14953)	5157 (3382-8463)	13864 (7699-29803)	7159 (4302-14584)

Location	Childhood wasting	Household air pollution	Ambient particulate matter pollution	Antibiotic treatment	Pneumococcal conjugate vaccine	Childhood stunted	Hib vaccine	Childhood under-weight	Hand-washing	Breast-feeding	Second-hand smoking
Ghana	2286 (1430-4342)	2993 (2000-4894)	3800 (2494-5924)	3627 (1785-10939)	34028 (19611-66897)	16116 (5067-630568)	28417 (13050-82467)	20167 (9821-44147)	8252 (5311-14235)	21124 (11411-51492)	44624 (25963-94823)
Guinea	519 (354-904)	585 (407-886)	1054 (712-1661)	923 (475-2736)	902 (650-1325)	2504 (883-29577)	1599 (912-4128)	3364 (1621-6626)	2086 (1372-3625)	3734 (2150-8210)	11019 (4640-61000)
Guinea-Bissau	1162 (757-2158)	1246 (865-1977)	1993 (1313-3294)	1888 (980-5597)	5233 (3126-9707)	5291 (1811-52426)	7329 (3786-20061)	6859 (3577-13491)	4406 (2931-7766)	8866 (5251-19205)	20683 (9076-90615)
Liberia	1486 (957-2663)	1593 (1061-2500)	5441 (3126-12824)	2546 (1234-7651)	6022 (3804-10108)	6943 (2487-62286)	5502 (2939-14255)	9493 (4374-20087)	5331 (3446-9452)	12072 (6990-23352)	19488 (10610-47197)
Mali	991 (640-1716)	1116 (738-1785)	1517 (1012-2464)	1893 (907-5660)	4332 (2742-7637)	5998 (2048-83929)	3735 (2005-10062)	5652 (2812-10966)	4145 (2613-7107)	9036 (5196-19118)	14151 (7558-40477)
Mauritania	1215 (819-1999)	2163 (1356-3987)	1647 (1137-2602)	2134 (1084-6250)	5237 (3446-8616)	7269 (2527-154612)	4745 (2720-11989)	6605 (3508-12489)	6234 (3868-11400)	7178 (4421-13816)	9237 (5192-20620)
Niger	476 (313-776)	572 (376-889)	639 (424-964)	937 (461-2789)	2557 (1553-4573)	1802 (703-17221)	2974 (1508-8872)	1762 (1005-3462)	2329 (1457-4043)	4551 (2551-10682)	8103 (4397-19226)
Nigeria	835 (544-1467)	1271 (817-2160)	1196 (818-1870)	1530 (750-4482)	2448 (1623-3882)	3537 (1354-59644)	2131 (1238-5160)	4080 (2097-7874)	3577 (2303-6311)	5748 (3445-10984)	12809 (7713-26616)
Sao Tome and Principe	2257 (1402-4399)	3261 (1924-7480)	8363 (4246-24881)	3816 (1837-11671)	27586 (15630-51765)	13113 (4452-787021)	23043 (11117-71380)	18572 (8849-41007)	10514 (6415-19154)	14926 (8647-30795)	89549 (43013-293675)
Senegal	1350 (897-2473)	1807 (1230-2997)	2261 (1534-3393)	2362 (1227-6945)	15055 (9685-25235)	8183 (2620-286575)	13323 (6840-35455)	8595 (4124-17752)	6195 (4063-10871)	10079 (5526-22080)	15554 (8061-38437)
Sierra Leone	468 (319-800)	553 (388-829)	1044 (720-1669)	843 (437-2303)	3179 (2009-5315)	1841 (717-18268)	2704 (1425-7750)	2588 (1297-4989)	1950 (1299-3309)	3144 (1946-5691)	4335 (2191-13606)
Togo	1277 (806-2338)	1436 (948-2256)	1848 (1242-2872)	2123 (1044-6448)	7639 (4875-13034)	7712 (2535-141621)	7278 (3830-19496)	8262 (4021-16707)	4852 (3095-8611)	13530 (7634-28474)	15354 (8871-32199)
